# Global patterns and trends in heart failure burden under chronic respiratory disease cause categories among adults aged 55 years and older: A systematic analysis based on the GBD 2021 Study

**DOI:** 10.1371/journal.pone.0353177

**Published:** 2026-07-21

**Authors:** Yinqin Liu, Anran Xu, Chen Zhang, ZhenYi Li, Shaobin Li, Hong Fang

**Affiliations:** 1 Longhua Clinical Medical College, Shanghai University of Traditional Chinese Medicine, China; 2 Longhua Hospital Shanghai University of Traditional Chinese Medicine, China; Universitas Syiah Kuala, INDONESIA

## Abstract

**Background:**

Chronic respiratory diseases (CRDs) and heart failure (HF) impose substantial global burdens. However, the long-term population-level burden of HF impairment under CRD cause categories remains insufficiently characterized within the GBD framework.

**Methods:**

Using GBD 2021 data, we estimated the prevalence and years lived with disability (YLDs) of HF impairment under CRD cause categories among adults aged ≥55 years from 1990 to 2021. CRD cause categories included chronic obstructive pulmonary disease (COPD), interstitial lung disease and pulmonary sarcoidosis (ILD&PS), and pneumoconiosis (PC). Cases, rates, relative changes, and EAPCs were used as primary descriptive measures. Joinpoint regression, age–period–cohort analysis, inequality indices, and BAPC-based projections were used as secondary analyses to describe trend timing, age–cohort patterns, socioeconomic distribution, and historical-trend extensions through 2032.

**Results:**

From 1990 to 2021, the global prevalent cases and YLDs of HF impairment under CRD cause categories increased markedly, while prevalence and YLD rates changed only modestly overall. The burden rose sharply with age, especially after age 75, and was generally higher in males. Temporal trends differed across periods. Patterns varied across SDI strata and differed by CRD subtype. Model-based extensions of historical patterns suggested that this burden pattern may remain substantial through 2032.

**Conclusion:**

The burden of HF impairment under CRD cause categories showed marked population-level variation across age, sex, SDI strata, and CRD subtypes. These findings may help inform burden monitoring, public health planning, resource allocation, and long-term service planning for older adults.

Chronic respiratory diseases (CRDs) are a group of diseases that affect the airways and lungs, including chronic obstructive pulmonary disease (COPD), interstitial lung disease and pulmonary sarcoidosis (ILD&PS), occupational lung diseases (such as pneumoconiosis, PC) and pulmonary hypertension. CRDs are often characterized by long-term persistence, progressive symptoms, and the need for ongoing management. Recurrent exacerbations and inadequate disease control may contribute to functional decline, hospitalizations, and increased mortality risk. According to statistics, CRDs were the third leading cause of death globally in 2019, resulting in approximately 4 million deaths and a global prevalence of 454.6 million cases, placing immense pressure and economic burden on healthcare systems worldwide [1].

Heart failure (HF), a complex syndrome characterized by dyspnea and fluid retention, has seen a global prevalence surge of 106.3% over the past three decades, exceeding 50 million cases by 2019 [2]. This burden is profoundly age-dependent: prevalence and healthcare utilization escalate precipitously with advancing age. For instance, U.S. data (2021–2023) indicate that HF prevalence in octogenarians is nearly double that of the 60–79 age group [3]. Surveillance studies further identify HF as the primary driver of hospitalization and adverse outcomes for individuals aged ≥65 years [4–6]. Ultimately, while age-standardized incidence may be stabilizing in some developed nations [7], the absolute caseload continues to intensify due to demographic aging, solidifying HF as a formidable global public health challenge.

CRDs and HF are prevalent conditions in aging populations, sharing clinical features such as recurrent exacerbations, functional limitation, and high hospitalization rates. In addition to shared risk factors such as smoking and cardiometabolic comorbidities, clinical and epidemiological evidence suggests that chronic respiratory impairment and cardiac dysfunction may intersect through several cardiopulmonary pathways [8,9]. CRD-associated chronic hypoxemia and pulmonary vascular remodeling can increase pulmonary vascular resistance, thereby elevating right ventricular afterload and predisposing susceptible individuals to right-sided cardiac dysfunction and HF decompensation [10,11]. Systemic inflammation and oxidative stress arising from chronic airway inflammation may also extend beyond local pulmonary effects and have been linked to endothelial dysfunction, atherosclerosis progression, and myocardial remodeling [9,12]. In CRDs, particularly COPD, pulmonary hyperinflation and altered intrathoracic pressure can impair venous return and ventricular filling, reduce stroke volume, and exacerbate dyspnea and exercise intolerance, thereby complicating the clinical assessment of HF [13]. Acute respiratory exacerbations, often triggered by infection, may further induce sympathetic activation and increased metabolic demand and have been temporally associated with acute cardiovascular events and HF-related hospitalizations, especially in frail older adults [14]. These overlapping clinical features and cardiopulmonary pathways highlight the relevance of examining the population-level burden of HF impairment under CRD cause categories among older adults.

Although CRDs and HF have each been widely examined in previous GBD studies, the long-term HF burden under CRD cause categories in older adults remains insufficiently characterized. To address this gap, we used GBD 2021 data to assess the global, regional, and national patterns of this burden among adults aged ≥55 years from 1990 to 2021, focusing on differences by CRD subtype, socioeconomic development level, and model-based trend extensions. These findings may provide a population-level basis for burden monitoring and public health planning in ageing populations.

## Methods

### Data source and extraction

This study was based on a secondary analysis of the publicly available GBD 2021 database, with data obtained through the Global Health Data Exchange (GHDx) (accessed November 28, 2024). We extracted estimates from 1990 to 2021 at the global, regional, and national levels, including five SDI quintiles and 21 GBD regions. In the GBD framework, heart failure was specified as the impairment of interest, and CRDs and their major subtypes—COPD, ILD&PS, and PC—were specified as causes. The primary outcomes were GBD-reported prevalence and years lived with disability (YLDs), and both number and rate metrics were extracted for each outcome. We restricted the analysis to adults aged ≥55 years to focus on a broad older-adult period, from late midlife to advanced older ages, when cardiopulmonary burden becomes more common [15]. Similar age cutoffs have been used in previous public health and GBD-based studies of older adults [16,17]. With this restriction, cases and YLDs were counted only within this age group, and crude rates were calculated using the corresponding population as the denominator. Because this age group covers a wide age range, crude rates may also be influenced by the age structure within this group. We therefore conducted age-stratified analyses to describe differences across older age groups. Counts and non-age-standardized rates were used for descriptive comparisons, whereas age-standardized rates were used for temporal trend analyses and model-based estimation of future trends to reduce the influence of changes in age structure. No missing values were identified in the extracted analytical dataset, and thus no imputation or complete-case analysis was required. Because this study was based on aggregated GBD estimates rather than original individual-level patient data, a conventional participant recruitment process was not applicable. Institutional review board approval was not required because all data were publicly available and de-identified. Both the raw data and code scripts are available on the Open Science Framework (OSF). For supplementary contextual comparison, corresponding GBD 2023 estimates were also extracted for the same outcomes, age range, CRD cause categories, and selected stratifications.

### Definitions

In GBD 2021, causes are organized within a hierarchical cause list. CRDs are classified as a Level 2 cause and comprise three Level 3 causes: COPD, ILD&PS, and PC. PC further includes silicosis, asbestosis, coal workers’ pneumoconiosis, and other pneumoconioses [18]. YLDs quantify non-fatal health loss and are calculated as prevalence multiplied by a disability weight [19]. In the GHDx results interface, HF is available as an impairment category of non-fatal health loss. Accordingly, the present analysis estimated the prevalence and YLDs of HF impairment allocated to CRD cause categories in the GBD framework. Therefore, these estimates should be interpreted as modeled population-level burden estimates within the GBD framework, rather than as clinically confirmed CRD–HF comorbidity or evidence of individual-level causal relationships.

### SDI

The Socio-demographic Index (SDI) is a composite measure of population and socioeconomic development, reflecting the link between social development and health outcomes. It combines per capita income, educational attainment for individuals aged 15 + , and fertility rates for women aged 15–24 to rank countries and regions into five SDI quintiles: High, High-middle, Middle, Low-middle, and Low SDI [20].

### Statistical analysis

We first summarized GBD-reported prevalent cases, YLDs, and corresponding rates in 1990 and 2021, and calculated relative changes from 1990 to 2021. Long-term changes in prevalence and YLD rates were quantified using the estimated annual percentage change (EAPC), which was derived by fitting a linear regression to the natural logarithm of each annual estimate [21]. This approach provides a concise summary of long-term temporal trends and has been widely used in chronic disease and global burden trend analyses [22]. EAPC estimates were interpreted as increasing, decreasing, or non-significant according to whether the 95% CI was above, below, or crossed zero. The burden measures and EAPC estimates served as the principal descriptive outputs for assessing burden magnitude and long-term direction of change.

Additional analyses were then used to examine specific features of these patterns. Joinpoint regression was applied to the GBD-reported prevalence and YLD rates to describe segment-specific changes in temporal trends. Segment-specific annual percent change (APC) and average annual percent change (AAPC) were estimated using the U.S. National Cancer Institute’s Joinpoint software, with 95% confidence intervals obtained via Monte Carlo permutation tests. Age–period–cohort analysis was used to examine age-, period-, and cohort-related patterns among adults aged ≥55 years [23,24]. Age was divided into nine groups from 55–59 years to ≥95 years, and the study period was divided into six periods from 1992–1996–2017–2021, excluding 1990–1991. The analysis was conducted using the NCI online APC tool, with 55–59 years and 2002–2006 as the reference groups. Sensitivity analyses using alternative reference groups were performed by comparing net drift and local drift estimates [25].

Cross-country inequalities were assessed using the Slope Index of Inequality (SII) and the Concentration Index (CI), with countries ranked by SDI. SII measured absolute rate inequality across the SDI distribution, whereas CI measured the population-weighted concentration of burden. Negative SII or CI values indicated higher rates or burden concentration in lower-SDI settings, while positive values indicated the opposite pattern {Jiang, 2023 #23} [26,27]. The two indices were interpreted as complementary measures of rate-based and population-weighted inequality.

To extend historical age-standardized trends of HF burden under CRD cause categories from 2022 to 2032, we used a Bayesian age–period–cohort (BAPC) model based on historical GBD 2021 estimates, IHME population forecasts, and the WHO 2000–2025 standard population [28,29]. The model generated extrapolated estimates of prevalent cases, YLDs, age-standardized prevalence rates, and age-standardized YLD rates up to 2032. Nordpred was used as a supplementary modelling comparison to examine whether the broad direction of change was consistent across approaches [30]. GBD 2023 estimates were used only for supplementary comparison with the GBD 2021-based results, focusing on whether broad burden patterns and detailed stratified trends differed between the two datasets. Data cleaning, statistical calculations, and visualization were performed using R software version 4.4.1, with graphical visualization generated using the ggplot2 package. Final figure refinement and layout optimization were completed in Adobe Illustrator version 2025 to improve visual clarity.

## Results

### Total CRDs

**Fig 1:**
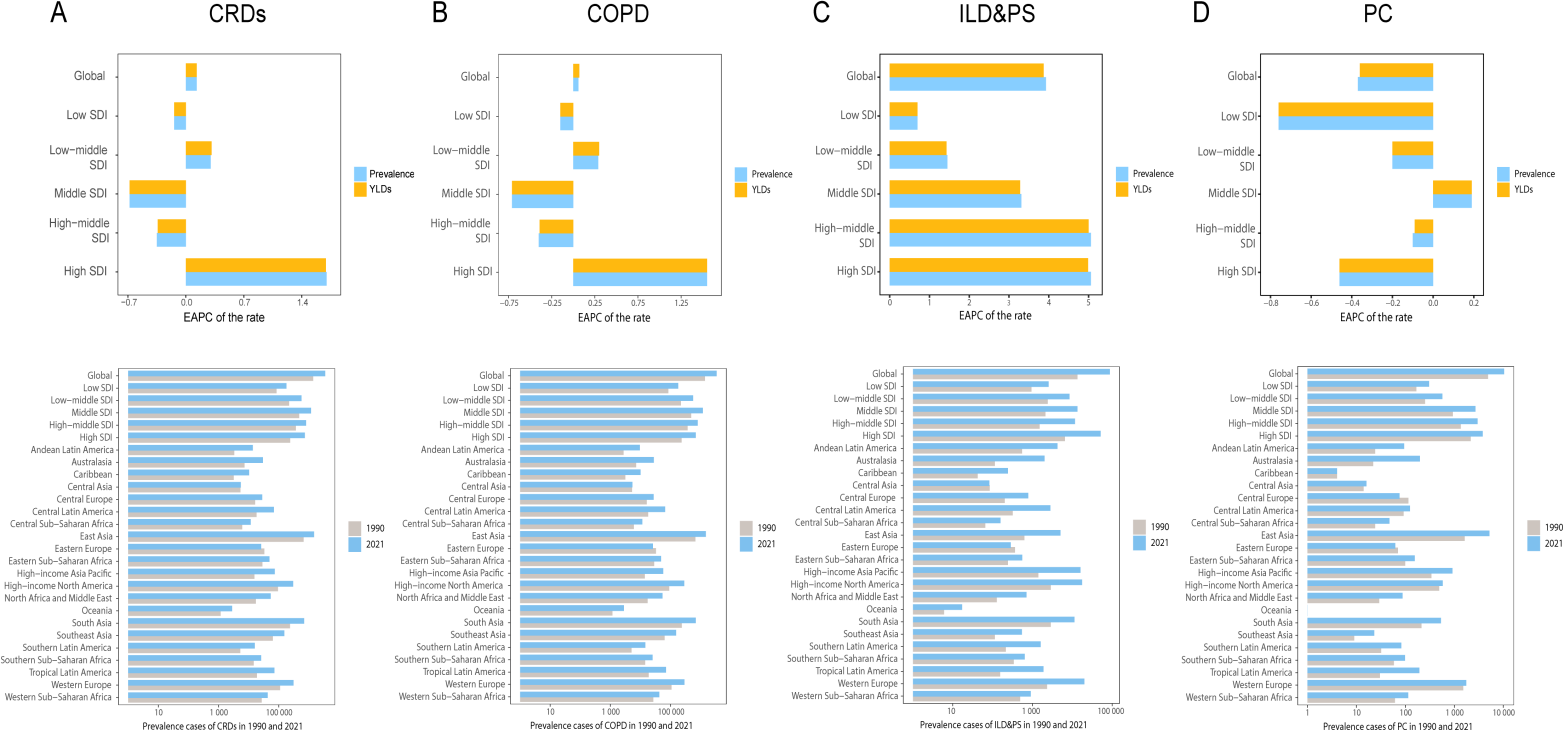
Prevalent cases in 1990 and 2021, and EAPCs of prevalence and YLD rates from 1990 to 2021 for HF impairment under CRD cause categories overall and for COPD, ILD&PS, and PC among adults aged ≥55 years. (A) CRDs; (B) COPD; (C) ILD&PS; (D) PC.

From 1990 to 2021, GBD 2021 estimates showed substantial increases in the global prevalent cases and YLDs of HF impairment under CRD cause categories among adults aged ≥55 years, each rising by approximately 150% (Table 1; Fig 1A; Supplementary Table S1 in S1 File). Based on EAPC, the long-term average change in rates was modest but positive for both the prevalence rate and YLD rate (both EAPC = 0.13). This modest global average masked large between-region contrasts: High-income Asia Pacific had the fastest rate increases (EAPC = 2.75 for prevalence rate; 2.72 for YLD rate), whereas Eastern Europe showed the steepest declines (both EAPCs = −2.27) (Table 1; Fig 1A; Supplementary Table S1 in S1 File). By SDI, the Middle SDI quintile carried the highest burden in 2021, while rate trajectories diverged over time (Middle SDI decreasing: EAPC = −0.68; High SDI increasing most rapidly: EAPC = 1.70) (Table 1; Fig 1A).

**Table 1:** Prevalent cases, prevalence rates, and EAPC trends of HF impairment under CRD cause categories from 1990 to 2021 among adults aged ≥55 years.

Location	Prevalent cases			Prevalence rates		
	1990(95% UI)	2021(95% UI)	Relative change, 1990–2021 (95% UI)	1990, per 100,000 (95% UI)	2021, per100,000(95% UI)	EAPC (95% CI)
Global	1397709 (1045163-1847182)	3489347 (2534499-4681296)	1.5 (1.42-1.53)	208.17 (155.66-275.11)	234.82 (170.56-315.03)	0.13 (0-0.25)
Low SDI	84618 (58368-123151)	180894 (124445-263660)	1.14 (1.13-1.14)	226.81 (156.45-330.09)	220.45 (151.66-321.31)	−0.14 (−0.25–-0.04)
Low-middle SDI	221311 (168268-291682)	569809 (416448-775902)	1.57 (1.47-1.66)	219.55 (166.93-289.36)	236.35 (172.74-321.84)	0.3 (0.2-0.41)
Middle SDI	480798 (361571-631584)	1184563 (855209-1610755)	1.46 (1.37-1.55)	277.02 (208.33-363.9)	252.11 (182.01-342.82)	−0.68 (−0.81–-0.54)
High-middle SDI	370570 (277385-483319)	813207 (592746-1104138)	1.19 (1.14-1.28)	214.79 (160.78-280.14)	234.57 (170.98-318.49)	−0.35 (−0.59–-0.12)
High SDI	239751 (173430-327573)	739402 (563304-958761)	2.08 (1.93-2.25)	128.58 (93.01-175.68)	214.31 (163.27-277.89)	1.7 (1.59-1.81)
Andean Latin America	3305 (2561-4243)	13812 (10685-17770)	3.18 (3.17-3.19)	98.49 (76.31-126.43)	139.43 (107.86-179.38)	1.66 (1.45-1.88)
Australasia	7295 (5352-9697)	29773 (23750-37012)	3.08 (2.82-3.44)	185.18 (135.85-246.16)	337.02 (268.83-418.96)	1.7 (1.51-1.88)
Caribbean	3171 (2364-4176)	10296 (7815-13382)	2.25 (2.2-2.31)	73.58 (54.85-96.89)	111.2 (84.41-144.53)	1.43 (1.17-1.69)
Central Asia	5361 (3726-7405)	5453 (3663-7664)	0.02 (−0.02-0.03)	67.03 (46.59-92.58)	37.48 (25.18-52.67)	−1.86 (−1.98–-1.74)
Central Europe	16372 (11443-22827)	28148 (20392-37349)	0.72 (0.64-0.78)	61.73 (43.15-86.08)	76.02 (55.07-100.87)	0.87 (0.78-0.97)
Central Latin America	18364 (13853-23606)	68677 (51012-90556)	2.74 (2.68-2.84)	135.33 (102.08-173.95)	160.59 (119.28-211.75)	0.5 (0.37-0.64)
Central Sub-Saharan Africa	6112 (3557-9867)	11735 (6999-19102)	0.92 (0.92-0.97)	162.54 (94.59-262.39)	130.05 (77.56-211.69)	−0.68 (−0.93–-0.44)
East Asia	670871 (497850-875778)	1497158 (1073194-2038066)	1.23 (1.16-1.33)	450.39 (334.23-587.96)	381.81 (273.69-519.75)	−1.25 (−1.5–-1)
Eastern Europe	33017 (22500-46869)	26002 (17023-38137)	−0.21 (−0.24–-0.19)	67.53 (46.02-95.86)	41.89 (27.42-61.43)	−2.27 (−2.6–-1.95)
Eastern Sub-Saharan Africa	28615 (17406-43578)	49118 (30235-75816)	0.72 (0.72-0.74)	235.21 (143.08-358.21)	181.67 (111.82-280.41)	−0.98 (−1.09–-0.87)
High-income Asia Pacific	15619 (9578-22808)	73406 (51283-102842)	3.7 (3.51-4.35)	44.67 (27.39-65.22)	104.12 (72.74-145.87)	2.75 (2.66-2.83)
High-income North America	93652 (63880-130176)	302486 (218169-415386)	2.23 (2.19-2.42)	161.67 (110.27-224.72)	268.79 (193.87-369.12)	1.61 (1.37-1.85)
North Africa and Middle East	17361 (13405-22171)	54109 (41733-69685)	2.12 (2.11-2.14)	61.42 (47.43-78.44)	70.98 (54.74-91.41)	0.85 (0.7-1)
Oceania	1173 (875-1532)	2822 (2124-3680)	1.41 (1.41-1.43)	243.86 (181.87-318.43)	228.67 (172.11-298.18)	−0.42 (−0.52–-0.31)
South Asia	237533 (178064-312255)	695280 (497270-961777)	1.93 (1.79-2.08)	250.19 (187.55-328.89)	280.02 (200.27-387.35)	0.41 (0.31-0.51)
Southeast Asia	63517 (48650-83390)	153056 (116782-200798)	1.41 (1.4-1.41)	150.01 (114.9-196.95)	133.61 (101.94-175.29)	−0.48 (−0.64–-0.33)
Southern Latin America	5254 (3673-7025)	16088 (11049-22054)	2.06 (2.01-2.14)	66.32 (46.37-88.68)	109.32 (75.08-149.86)	1.76 (1.54-1.97)
Southern Sub-Saharan Africa	14636 (9349-21462)	25985 (16445-38838)	0.78 (0.76-0.81)	330.77 (211.3-485.05)	266.91 (168.93-398.94)	−0.69 (−0.94–-0.43)
Tropical Latin America	18789 (13725-25196)	72089 (51266-100903)	2.84 (2.74-3)	124.09 (90.65-166.41)	162.74 (115.73-227.78)	0.79 (0.69-0.89)
Western Europe	110718 (79645-148437)	310805 (238458-394697)	1.81 (1.66-1.99)	114.01 (82.01-152.85)	208.41 (159.89-264.66)	2.34 (2.19-2.49)
Western Sub-Saharan Africa	26974 (16451-41539)	43048 (25747-67687)	0.6 (0.57-0.63)	186.86 (113.96-287.76)	133.93 (80.1-210.58)	−1.1 (−1.13–-1.07)

**Fig 2:**
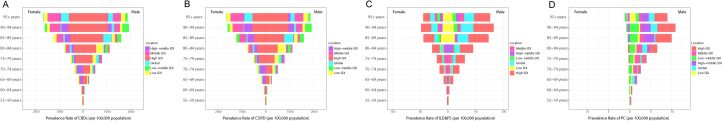
Sex- and age-specific prevalence rates of HF impairment under CRD cause categories overall and for COPD, ILD&PS, and PC in 2021 among adults aged ≥55 years. (A) CRDs; (B) COPD; (C) ILD&PS; (D) PC.

**Fig 3:**
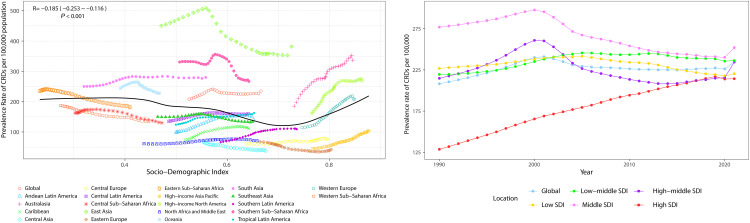
Associations between SDI and prevalence rates of HF impairment under CRD cause categories across 21 GBD regions, and temporal trends in prevalence rates from 1990 to 2021 among adults aged ≥55 years.

**Fig 4:**
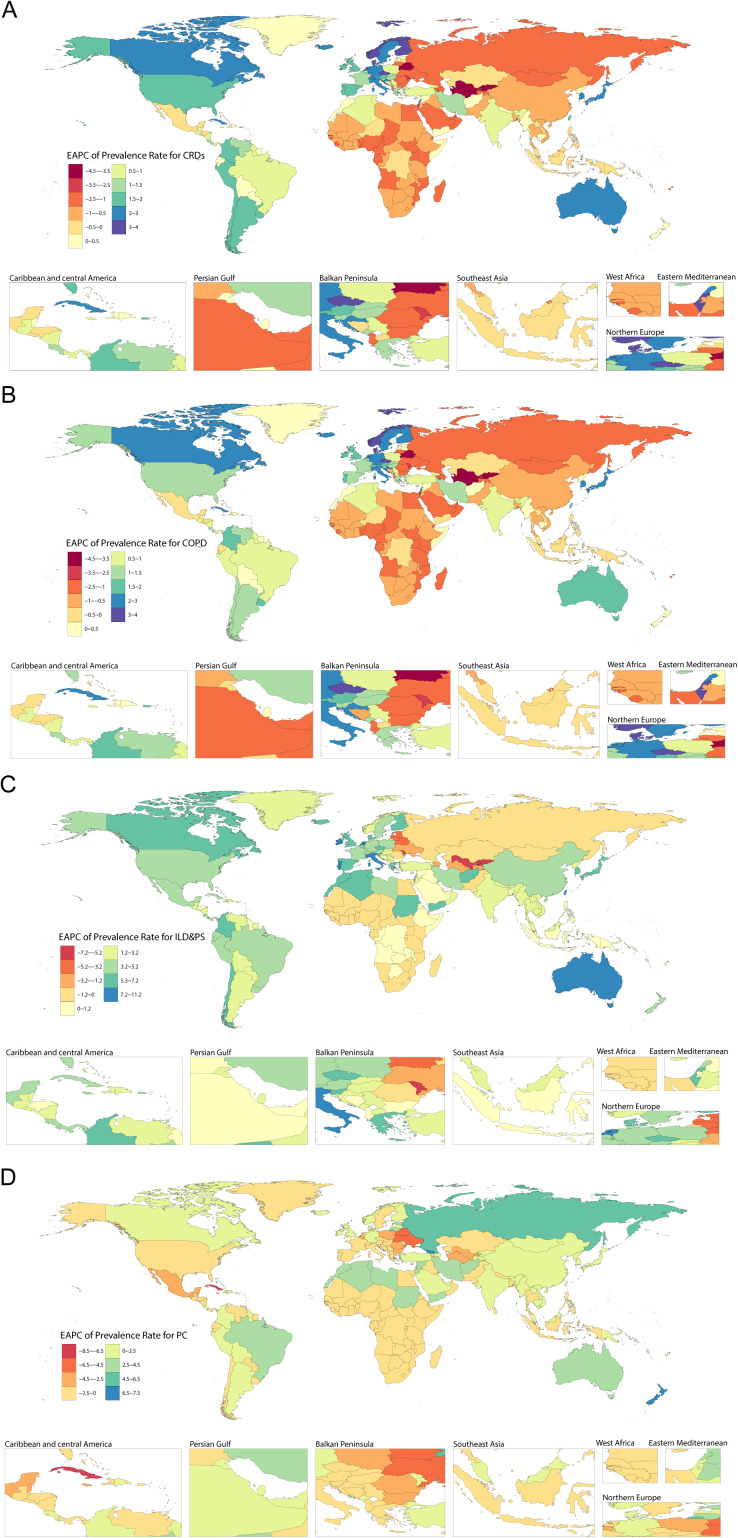
EAPCs of prevalence rates for HF impairment under CRD cause categories overall and for COPD, ILD&PS, and PC across 204 countries and territories from 1990 to 2021 among adults aged ≥55 years. (A) CRDs; (B) COPD; (C) ILD&PS; (D) PC.

These average trends occurred against a pronounced age–sex gradient and non-linear development pattern. In 2021, prevalence and YLD rates rose steeply with age—especially from age 75 years onward—and were generally higher in males, with the sex gap widening in the oldest age groups (Fig 2A; S1A Fig). Regionally, rates tended to decline with increasing SDI across low-to-middle SDI settings (SDI ~ 0.4–0.6) but rose again when SDI exceeded ~0.7 and peaked in high-SDI regions (Fig 3; S3A Fig). Country-level patterns were consistent with this heterogeneity: > 90% of countries experienced increases in absolute burden, whereas rate changes were split (52.0% increasing vs 48.0% decreasing) (Fig 4A; S4A Fig; Supplementary Tables S8–S9 in S1 File).

**Fig 5:**
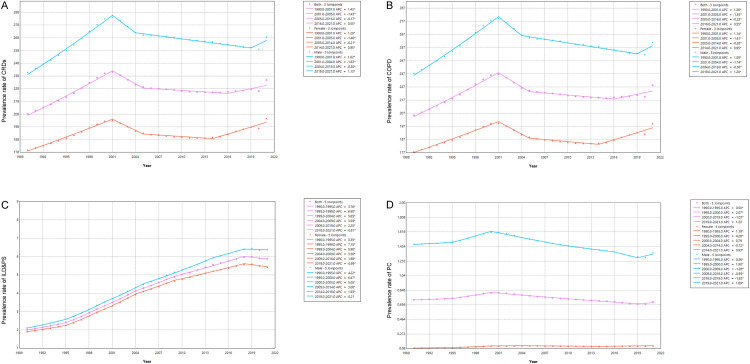
Joinpoint regression analysis of temporal trends in prevalence rates for HF impairment under CRD cause categories overall and for COPD, ILD&PS, and PC from 1990 to 2021 among adults aged ≥55 years. (A) CRDs; (B) COPD; (C) ILD&PS; (D) PC.

**Fig 6:**
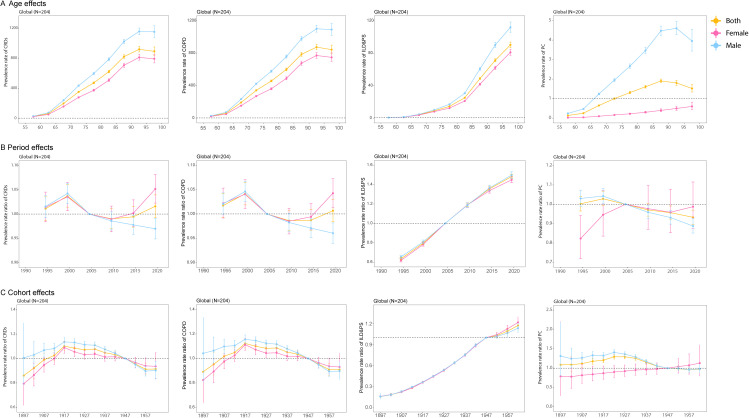
Age, period, and cohort effects on prevalence rates of HF impairment under CRD cause categories overall and for COPD, ILD&PS, and PC among adults aged ≥55 years. (A) Age effects; (B) Period effects; (C) Cohort effects.

To further characterize the temporal structure behind the long-term EAPC estimates, Joinpoint regression indicated phase-specific changes in rates. The prevalence rate increased during 1990–2001, then plateaued or declined during 2001–2016, and increased again after 2016 (AAPC = 0.35%, 95% CI: 0.22–0.47) (Fig 5A; Supplementary Table S16 in S1 File). For the YLD rate, the most pronounced recent increase occurred during 2019–2021 (APC = 1.76%) (S5A Fig; Supplementary Table S17 in S1 File). The A–P–C results also highlighted the strong age gradient observed in the descriptive burden estimates. Although the global net drift of prevalence was close to null (−0.06% per year), the age effect increased sharply in the oldest groups, particularly among adults aged ≥80 years, consistent with the cross-sectional pattern in 2021 (Fig 6A; S6A Fig; Supplementary Tables S24–S26 in S1 File). Period effects showed only limited elevation around the late 1990s/early 2000s, whereas cohort effects peaked in earlier birth cohorts and declined in later cohorts (Fig 6B–6C; S6B–S6C Fig; Supplementary Tables S27–S28 in S1 File). SDI-stratified results further indicated sustained increases in high-SDI settings but declines in middle and low SDI quintiles (Supplementary Tables S24–S30 in S1 File).

**Fig 7:**
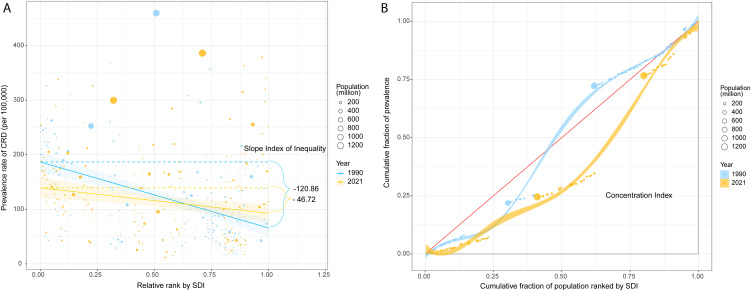
Health inequality in the prevalence burden of HF impairment under CRD cause categories worldwide from 1990 to 2021 among adults aged ≥55 years. (A) Slope index of inequality; (B) Concentration index.

**Fig 8:**
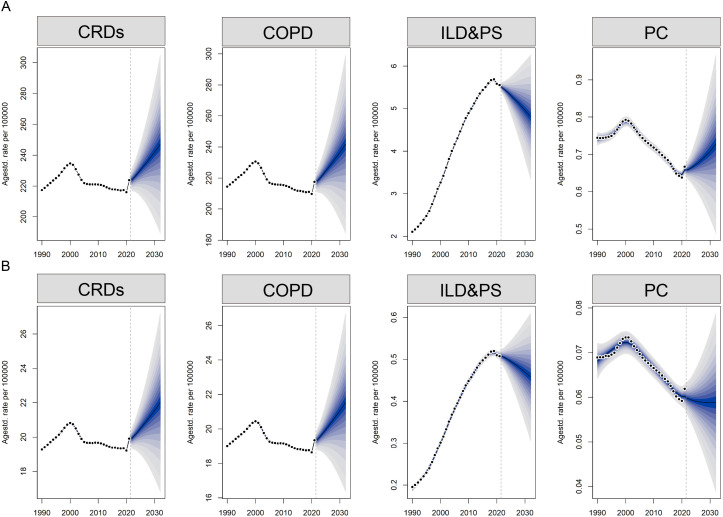
Observed and projected trends in age-standardized prevalence rates and age-standardized YLD rates of HF impairment under CRD cause categories overall and for COPD, ILD&PS, and PC globally from 1990 to 2032 among adults aged ≥55 years. (A) Age-standardized prevalence rate; (B) Age-standardized YLD rate. The black line represents the global estimate, and shaded areas indicate 95% UI.

Inequality analyses based on SDI ranking showed that absolute inequality in crude prevalence rates decreased between 1990 and 2021, as indicated by the SII becoming less negative from −120.86 to −46.72 (Fig 7A). In parallel, the concentration curve/CI suggested that prevalent cases in 2021 remained relatively concentrated toward higher-SDI settings (Fig 7B), reflecting differences between rate-based absolute inequality and population-weighted case distribution. When historical age-standardized trends were extended to 2032 using the BAPC model, the corresponding 2032 estimates for ASPR and age-standardized YLD rate were 247.35 and 22.02 per 100,000, respectively. Nordpred showed a broadly consistent direction of change (Fig 8A–8B; Supplementary Tables S31–S33 in S1 File).

### COPD

Within the COPD cause category, global prevalent cases and YLDs of HF impairment increased substantially among adults aged ≥55 years from 1990 to 2021, each rising by approximately 146% (Fig 1B; Supplementary Tables S2–S3 in S1 File). However, rate-based growth was comparatively modest: EAPC estimates were 0.06 for the prevalence rate and 0.07 for the YLD rate, indicating that the long-term rate increase in the COPD subgroup was weaker than that observed for total CRDs, despite the marked rise in counts. Regional trends remained heterogeneous: High-income Asia Pacific showed the fastest increases (EAPC = 2.28 and 2.26), whereas Eastern Europe showed the clearest declines (EAPC = −2.27 and −2.26) (Fig 1B; Supplementary Tables S2–S3 in S1 File).

In 2021, the COPD subgroup showed a steep age gradient in both prevalence and YLD rates, with higher rates in males and the highest burden observed among adults aged ≥75 years (Fig 2B; S1B Fig). By SDI, the Middle SDI quintile carried the largest burden in 2021, but trajectories diverged: the Middle SDI quintile declined over time (EAPC = −0.71), while the High SDI quintile increased most clearly (EAPC = 1.55) (S2A and S3B Fig; Supplementary Tables S2–S3 in S1 File). At the country level, increases in absolute burden were widespread (93.6% of countries), yet rate increases were less common (observed in 46.6% of countries), indicating that increases in absolute burden were not always accompanied by increases in rates; Luxembourg showed the fastest growth in rates (EAPCs 4.57 and 4.55) (Fig 4B; S4B Fig; Supplementary Tables S10–S11 in S1 File).

Joinpoint regression indicated that the weak long-term rate increase for COPD was not linear. Prevalence rates increased during 1990–2001, plateaued or declined during 2001–2016, and rose again after 2016 (overall AAPC = 0.29%, females 0.32%) (Fig 5B; Supplementary Table S18 in S1 File). YLD rates showed a comparable segmented pattern, with an overall AAPC of 0.33% and the sharpest recent increase during 2019–2021 (APC = 1.87%) (S5B Fig; Supplementary Table S19 in S1 File). A–P–C analysis showed a contrast between the overall rate trajectory and age-specific changes: although the net drift was slightly negative (−0.13% per year), local drift remained positive among adults aged ≥80 years, despite declining among those aged 55–79 years (Fig 6A; S6A Fig; Supplementary Tables S24–S26, S29–S30 in S1 File). Period effects were elevated around 1997–2001, and cohort risks generally declined after 1947, although a rising tendency persisted in more recent female cohorts (Fig 6B–6C; S6B–S6C Fig; Supplementary Tables S27–S28 in S1 File).

Inequality analyses showed a narrowing absolute gap in crude prevalence rates from 1990 to 2021, with the SII changing from approximately −120.73 to −51.15 (S7A Fig). Despite this narrowing, COPD retained a clearly negative SII in 2021, indicating higher crude rates in lower-SDI settings. Meanwhile, the concentration curve/CI suggested that prevalent cases remained relatively concentrated toward higher-SDI settings (S7B Fig). In the BAPC analysis, the 2032 estimates for COPD were 243.91 per 100,000 for ASPR and 21.54 per 100,000 for the age-standardized YLD rate (Fig 8A–8B; Supplementary Tables S31–S32 in S1 File).

### ILD&PS

Among the CRD subtypes, the ILD&PS subgroup showed the largest increase in estimated HF impairment burden from 1990 to 2021: global prevalent cases and YLDs rose by more than 500%, and both rates increased rapidly (EAPC = 3.93 for prevalence rate; 3.87 for YLD rate) (Fig 1C; Supplementary Tables S4–S5 in S1 File). Unlike total CRDs and COPD, where the long-term average rate changes were modest, ILD&PS displayed a pronounced and sustained rate escalation. Regional trends varied substantially, with Australasia showing the fastest growth (EAPC = 7.16 and 7.03) and Eastern Europe showing the steepest decline (EAPC = −3.09 for both rates) (Fig 1C; Supplementary Tables S4–S5 in S1 File).

Unlike CRDs overall and COPD, the ILD&PS-specific burden was concentrated in high-development settings. In 2021, both counts and rates peaked in the High SDI quintile, and rates were positively correlated with SDI (S2B Fig; S3C Fig; Supplementary Tables S4–S5 in S1 File). Country-level contrasts were also sharper than those observed for COPD: Montenegro showed the largest increase (and the fastest rate growth; EAPC = 12.48 and 12.47), whereas Mauritania was the only country with a notable decline in absolute burden (Fig 4C; S4C Fig; Supplementary Tables S12–S13 in S1 File). Age–sex patterns followed the expected gradient (higher burden at older ages and generally higher in males); however, at the oldest ages (≥90 years), female burden tended to approach or exceed males in some high-SDI settings (Fig 2C; S1C Fig).

The time pattern for ILD&PS differed from that observed for CRDs overall and COPD. Although the long-term EAPC showed a pronounced increase, Joinpoint analysis indicated that the rise softened in the most recent period, with a decline after 2018 for both prevalence and YLD rates (prevalence AAPC = 3.54%; YLD rate AAPC = 3.49%) (Fig 5C; S5C Fig; Supplementary Tables S20–S21 in S1 File). In the A–P–C analysis, ILD&PS showed continued growth in prevalence, with an overall net drift of 3.49% per year. Period risks increased after 2002, and cohort risks rose among individuals born after 1947, particularly in higher-SDI settings (Fig 6; S6 Fig; Supplementary Tables S24–S30 in S1 File). Inequality analyses showed that ILD&PS differed by SDI in both crude rates and case distribution. For absolute inequality, the SII increased from −0.14 in 1990 to 6.19 in 2021, indicating that crude prevalence rates were higher in higher-SDI settings by 2021 (S7A Fig). For relative, population-weighted inequality, the CI showed that prevalent cases in 2021 were distributed more toward higher-SDI countries (S7B Fig). For ILD&PS, the modelled 2032 values moved in different directions across the two age-standardized indicators, with ASPR declining to 4.80 per 100,000 and the age-standardized YLD rate increasing to 0.46 per 100,000 (Fig 8A-8B; Supplementary Tables S31–S32 in S1 File).

### PC

From 1990 to 2021, the PC subgroup showed a distinct count–rate divergence among adults aged ≥55 years: prevalent cases and YLDs increased by 113%, whereas prevalence and YLD rates declined (EAPC = −0.37 and −0.36, respectively) (Fig 1D; Supplementary Tables S6–S7 in S1 File). This pattern contrasted with CRDs overall, COPD, and ILD&PS, where rate changes were more stable or increasing. Regional and country-level results reinforced this PC-specific pattern. Central Europe showed the sharpest rate decline (EAPC = −3.67), whereas Australasia showed the most notable rate increases (EAPC = 4.96 for prevalence; 4.95 for YLDs) (Fig 1D; Supplementary Tables S6–S7 in S1 File). At the country level, the largest rise in absolute burden occurred in the Maldives (+251.9%), while Qatar showed the most substantial decrease (−86%). For rates, declines predominated globally, occurring in 61.8% of countries (Fig 4D; S4D Fig; Supplementary Tables S14–S15 in S1 File).

Joinpoint analysis added temporal detail to the overall declining rate trend. Rates decreased overall (AAPC = −0.17 for prevalence; −0.16 for YLDs), with a brief rise during 1995–2000, a prolonged decline during 2000–2019, and a slight rebound in YLD rates during 2019–2021 (Fig 5D; S5D Fig; Supplementary Tables S22–S23 in S1 File). Sex-specific results showed a different pattern among females, whose prevalence and YLD rates increased throughout the study period (both AAPCs = 0.95%), despite the overall decline (Supplementary Tables S22–S23 in S1 File). The A–P–C results were in line with the overall downward rate tendency for PC. The net drift was −0.35% per year, period risk peaked around 1997–2001, and cohort risks generally declined after 1947 (Fig 6; S6 Fig; Supplementary Tables S24–S30 in S1 File).

Inequality patterns were less pronounced than those observed for the other CRD subtypes. The SII changed only slightly from 0.02 in 1990 to 0.15 in 2021, suggesting limited change in absolute inequality in crude prevalence rates. The CI showed only a mild population-weighted concentration of prevalent cases toward higher-SDI settings (S7A–S7B Fig). In the model-based extension of historical age-standardized trends, the 2032 estimates for PC were 0.73 per 100,000 for ASPR (+10.4%) and 0.06 per 100,000 for the age-standardized YLD rate (−2.2%) (Fig 8A–8B; Supplementary Tables S31–S32 in S1 File).

### Comparison with GBD 2023 estimates

To contextualize the GBD 2021-based findings, we performed a supplementary comparison using updated GBD 2023 estimates. Broad burden patterns were highly consistent across both datasets, including the longitudinal increase in absolute burden, the steep age-related rise among adults aged ≥55 years, and the persistent heterogeneity across CRD cause categories. Minor variations were noted in some specific estimates, particularly in 2021 rate values and SDI-stratified EAPCs. Overall, the supplementary comparison supported the stability of the broad descriptive findings, while indicating that detailed stratified trend estimates were more sensitive to the dataset used (Supplementary Tables S34–S37 in S1 File).

## Discussion

This study examined the global, regional, and national population-level burden of HF impairment under CRD cause categories among adults aged ≥55 years from 1990 to 2021. Evaluated through basic burden metrics and long-term EAPC trends, the findings showed a substantial increase in absolute burden, alongside modest global changes in rates. The data also indicated a strong age gradient concentrated in older age groups, as well as marked heterogeneity across regions, countries, sexes, SDI strata, and CRD subtypes. COPD accounted for the largest share of the burden, whereas ILD&PS and pneumoconiosis showed distinct geographic and temporal patterns. Together, these findings highlight an older-adult burden pattern that differs clearly by age, socioeconomic context, and CRD subtype.

The substantial rise in absolute burden occurred alongside relatively modest long-term changes in rates. This pattern is closely linked to population ageing within the ≥ 55-year group. As the number of older adults increases, especially in the more advanced age groups, prevalent cases and YLDs can rise markedly even when prevalence and YLD rates change only modestly. United Nations projections also indicate that population ageing will continue in the coming decades [31]. As additional background context, longer average life expectancy, a growing proportion of older adults living with multiple chronic diseases, and more comprehensive case reporting systems may further contextualize the increase in case numbers [32–35]. In this setting, counts reflect the absolute volume of health loss, whereas rates describe the burden level within the population being measured. Age-standardized rates provide a more comparable view of temporal and between-population patterns because they reduce the influence of differences in age structure [36,37].

Regional and SDI differences suggest that population ageing alone does not fully account for the heterogeneity observed across settings. In low- and middle-SDI settings, the burden may be viewed alongside continued exposure to tobacco smoke, household and ambient air pollution, and occupational dusts, as well as more limited access to prevention and long-term disease management [1,38]. In high-SDI settings, longer survival with chronic cardiopulmonary disease, residual exposure accumulated earlier in life, and better recognition of chronic cardiopulmonary conditions may be linked to higher recorded burden [39]. Overall, the uneven patterns across SDI levels should be understood in relation to ageing, exposure profiles, and health-system context.

From the perspective of SDI-ranked inequality, the main implication is that a narrowing rate gap should not be interpreted as evidence that the overall burden became lighter or more evenly distributed. In this study, the narrowing of rate-based inequality occurred alongside a continued concentration of prevalent burden toward higher-SDI settings. This pattern is particularly relevant in older populations, where the absolute number of affected individuals is strongly shaped by population ageing, longer survival with chronic disease, and the size of the population at risk. Therefore, lower-SDI settings may still show higher crude rates, while higher-SDI settings account for a larger share of population-weighted prevalent burden. The finding does not indicate inconsistency between inequality measures, but rather shows that rate differences and burden concentration reflect different dimensions of the same SDI-ranked burden pattern. The subtype findings further indicate that this pattern should not be generalized across all CRD categories. ILD&PS showed a clearer shift toward higher-SDI settings, whereas pneumoconiosis displayed a weaker and more mixed inequality pattern. These differences suggest that the overall CRD estimate masks important subtype-specific variation. Interpretation of inequality in GBD-modeled HF impairment under CRD cause categories should therefore consider rate differences, population-weighted burden concentration, absolute case burden, and subtype composition together.

Age- and sex-stratified patterns provide further context for interpreting the burden. The burden increased with age and was highest among adults aged ≥75 years, showing that the burden became more pronounced from age 75 onward. This finding is consistent with previous evidence that chronic disease burden is greater in later life [40]. Rather than reflecting individual clinical diagnoses, this age gradient may be viewed alongside the gradual decline in physiological function and long-term cumulative health loss associated with ageing. Men generally had a higher burden than women, which may be linked to smoking, occupational exposures, and other related risk factors [1,41]. However, this sex difference should be considered together with age structure. In some settings, older women also show a relatively high burden. This pattern may be associated with their longer life expectancy and a larger proportion of women reaching advanced age [42,43]. This heavy concentration of burden at advanced ages highlights why the age-stratified results are so important. Although the overall ≥55-year estimates provide a useful summary of the broad older-adult period, they average out the internal differences within this group. Specifically, the overall rate masks the relatively lower burden among adults aged 55–74 years and the pronounced spike from age 75 onward. Therefore, age-specific results should always be considered together with the overall estimates when interpreting the burden in older adults.

Turning to the temporal dimension, the late-stage patterns differed across CRD subtypes. For CRDs overall and COPD, YLD rates increased during 2019–2021, whereas ILD&PS rates declined from 2018 to 2021. These changes occurred during a period when healthcare access, routine follow-up, diagnosis, and chronic disease management were widely affected [44]. This background may be relevant because COPD often requires continuous management and rehabilitation, while ILD&PS relies more on imaging and specialist assessment [45,46].

The A–P–C results provide a longer-term view of these trends. For CRDs overall, as well as the COPD and PC subgroups, later birth cohorts showed lower risks. This pattern aligns with gradual improvements in disease prevention and routine care, such as the wider use of clinical guidelines [47]. For PC, it may also reflect changes in workplace protection and dust exposure, although earlier occupational exposures remain important because work-related lung damage often develops over many years [41,45,48]. In contrast, ILD&PS showed higher cohort-related estimates in later-born groups. This pattern may be partly related to the slow course of fibrotic lung disease and improved diagnostic recognition over time [49,50]. When the observed historical patterns were extended to 2032, the burden of HF impairment under CRD cause categories among adults aged ≥55 years did not show a clear decline. This was in line with the main finding from 1990 to 2021: the number of cases and YLDs increased substantially, whereas changes in rates were relatively small. As the older population continues to grow, even stable or slowly changing rates may still translate into a considerable care burden. These results suggest that HF impairment under CRD cause categories may remain an important issue for burden monitoring, service planning, and long-term care in older adults. However, the projections should be understood mainly as an extension of past trends, rather than as precise estimates of the future burden.

The supplementary comparison with GBD 2023 provides important context for interpreting the GBD 2021-based findings. Differences between GBD versions are expected because GBD updates may include new data sources, revised modelling strategies, and changes in disease classification. In this study, the main burden patterns were relatively stable between the two GBD versions, whereas small rate changes and narrowly stratified estimates were more likely to vary after database updates. Therefore, the main value of this study lies in identifying large-scale burden patterns, SDI-related differences, and subgroup heterogeneity, rather than in providing exact numerical estimates. Detailed stratified trends should consequently be interpreted with caution.

Ultimately, these findings suggest that the burden of HF impairment within CRD cause categories in older adults should be understood as a population-level disability burden within the GBD framework. When monitoring this burden, it is more practical to look at the number of cases, prevalence rates, and disability as a whole, as each paints a different part of the clinical picture. For planning services, the rising absolute number of patients underscores the need for ongoing support—including chronic care, rehabilitation, and disability management—as our populations age. Practically, these results suggest that resource allocation should be focused on the specific groups or settings bearing the heaviest load, rather than just the global average.We hope this evidence provides a clearer map for health systems to prioritize services and meet these evolving clinical needs.

### Limitations

This study has several limitations. First, GBD estimates depend on the availability and quality of source data and on modelling assumptions across countries and regions. In settings with sparse data or incomplete surveillance systems, estimates may have greater uncertainty, and updates to GBD data inputs or modelling methods may affect some stratified results. Second, differences in diagnostic capacity, healthcare access, and reporting standards may lead to under-ascertainment or misclassification of HF and CRDs, thereby influencing prevalence and YLD estimates. Third, the analysis was restricted to adults aged ≥55 years. Although this restriction allowed us to focus on older adults, the estimates are not directly comparable with all-age studies, and crude rates may still be affected by the age structure within the ≥ 55-year population. Fourth, this study used aggregated population-level estimates and could not account for individual-level factors. Therefore, the findings should be interpreted as population-level epidemiological patterns rather than individual-level clinical relationships or causal effects. Finally, the BAPC projections were extensions of historical age-period-cohort patterns rather than deterministic forecasts. Their precision may decrease over a ten-year horizon, and future changes in population structure, diagnostic access, healthcare practice, environmental exposures, or GBD modelling strategies may lead to trends that differ from the projected estimates.

### Conclusion

In summary, using GBD 2021 data, this study described the burden of HF impairment under CRD cause categories among adults aged ≥55 years from 1990 to 2021. The burden varied across regions, SDI strata, age groups, sex, and CRD subtypes. Within the GBD framework, these findings may help inform burden monitoring, public health planning, resource allocation, and long-term service planning for older adults.

## Supporting information

S1 Fig Sex- and age-specific YLD rates of HF impairment under CRD cause categories overall and for COPD, ILD&PS, and PC in 2021 among adults aged ≥55 years. (A) CRDs; (B) COPD; (C) ILD&PS; (D) PC.
(TIF)

S2 Fig Associations between SDI and prevalence rates of HF impairment under the COPD, ILD&PS, and PC cause categories across 21 GBD regions, and temporal trends in prevalence rates from 1990 to 2021 among adults aged ≥55 years. (A) COPD; (B) ILD&PS; (C) PC.
(TIF)

S3 Fig Associations between SDI and YLD rates of HF impairment under CRD cause categories overall and for COPD, ILD&PS, and PC across 21 GBD regions, and temporal trends in YLD rates from 1990 to 2021 among adults aged ≥55 years. (A) CRDs; (B) COPD; (C) ILD&PS; (D) PC.
(TIF)

S4 Fig Percentage change in prevalent cases of HF impairment under CRD cause categories overall and for COPD, ILD&PS, and PC across 204 countries and territories from 1990 to 2021 among adults aged ≥55 years. (A) CRDs; (B) COPD; (C) ILD&PS; (D) PC.
(TIF)

S5 Fig Joinpoint regression analysis of temporal trends in YLD rates for HF impairment under CRD cause categories overall and for COPD, ILD&PS, and PC from 1990 to 2021 among adults aged ≥55 years. (A) CRDs; (B) COPD; (C) ILD&PS; (D) PC.
(TIF)

S6 Fig Age, period, and cohort effects on YLD rates of HF impairment under CRD cause categories overall and for COPD, ILD&PS, and PC among adults aged ≥55 years. (A) Age effects; (B) Period effects; (C) Cohort effects.
(TIF)

S7 Fig Health inequality in the prevalence burden of HF impairment under the COPD, ILD&PS, and PC cause categories worldwide from 1990 to 2021 among adults aged ≥55 years. (A) Slope index of inequality; (B) Concentration index.
(TIF)

S1 File This file contains all supplementary tables, including Tables S1–S37. (DOCX)

## Abbreviations

CRDs: Chronic respiratory diseases
COPD: Chronic obstructive pulmonary disease
ILD&PS: Interstitial lung disease and pulmonary sarcoidosis
PC: Pneumoconiosis
HF: Heart failure
YLDs: Years lived with disability
GBD: Global Burden of Disease
GHDx: Global Health Data Exchange
SDI: Socio-demographic Index
EAPC: Estimated Annual Percentage Change
APC: Annual Percent Change
AAPC: Average Annual Percent Change
A–P–C: Age–Period–Cohort
SII: Slope Index of Inequality
CI: Concentration Index
BAPC: Bayesian age–period–cohort model
WHO: World Health Organization
ASPR: Age-standardized prevalence rate.
