# Supplementary figures and images for: Global patterns and trends in heart failure burden under chronic respiratory disease cause categories among adults aged 55 years and older: A systematic analysis based on the GBD 2021 Study
Source: PLoS One. 2026 Jul 21;21(7):e0353177. doi: 10.1371/journal.pone.0353177 (PMC13387532; doi:10.1371/journal.pone.0353177)

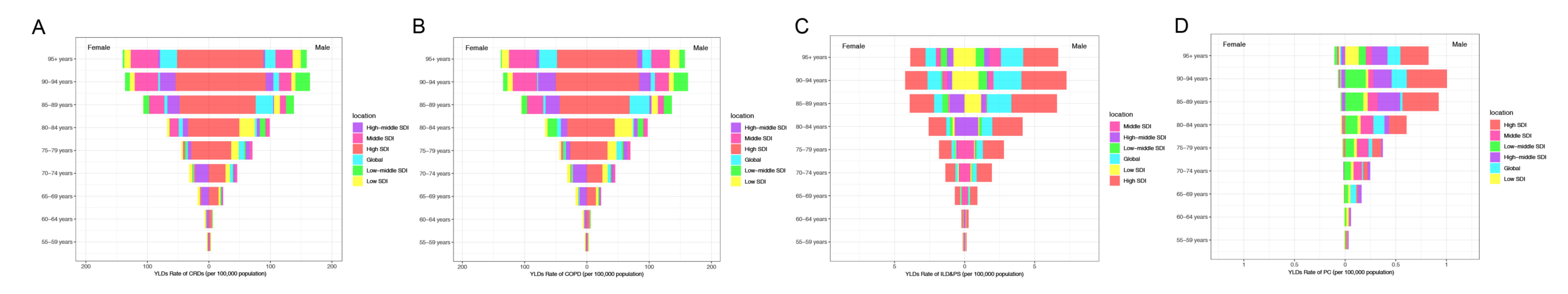

Supplement: S1 Fig — (A) CRDs; (B) COPD; (C) ILD&PS; (D) PC. (TIF) [file pone.0353177.s001.tif]

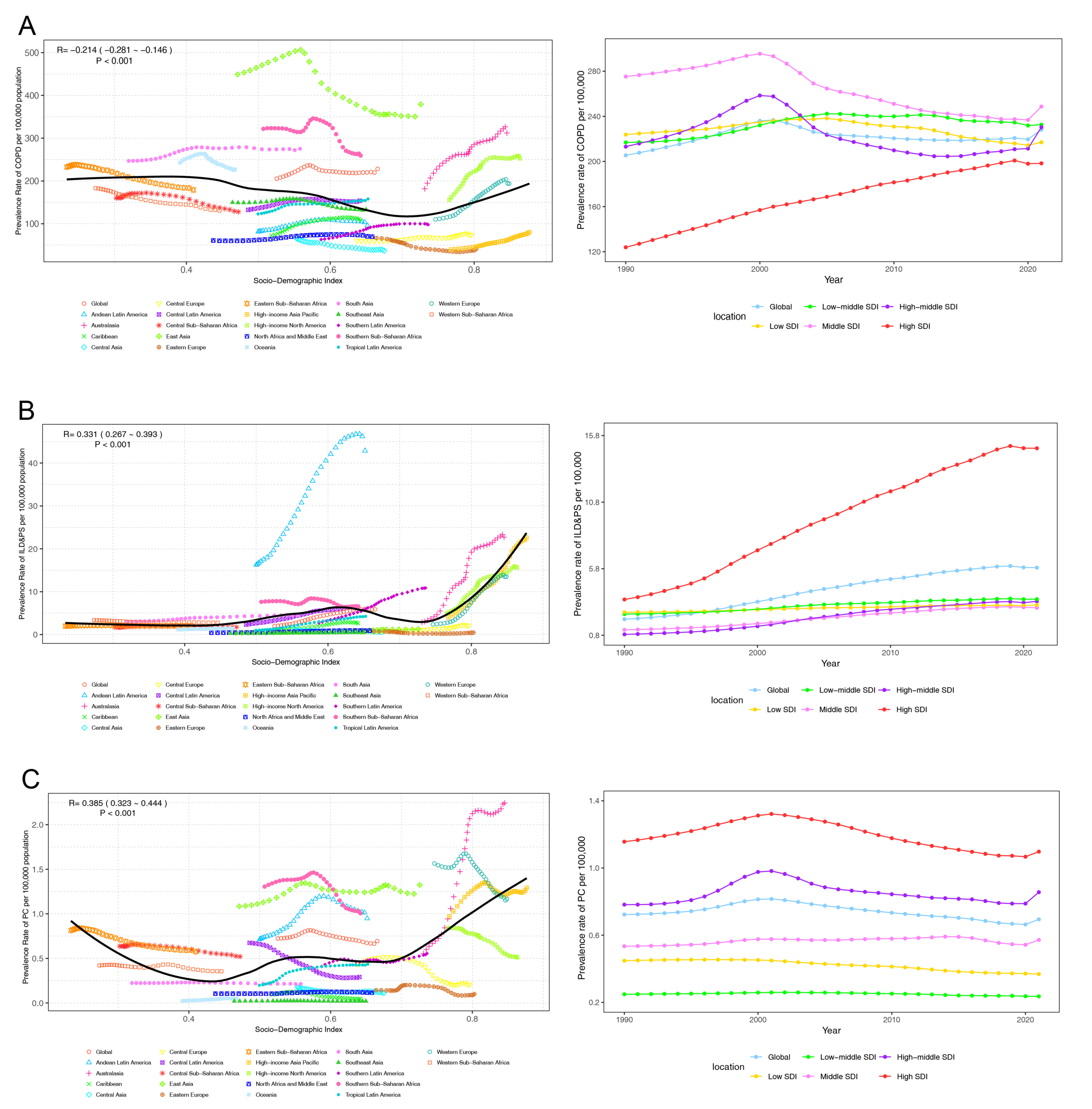

Supplement: S2 Fig — (A) COPD; (B) ILD&PS; (C) PC. (TIF) [file pone.0353177.s002.tif]

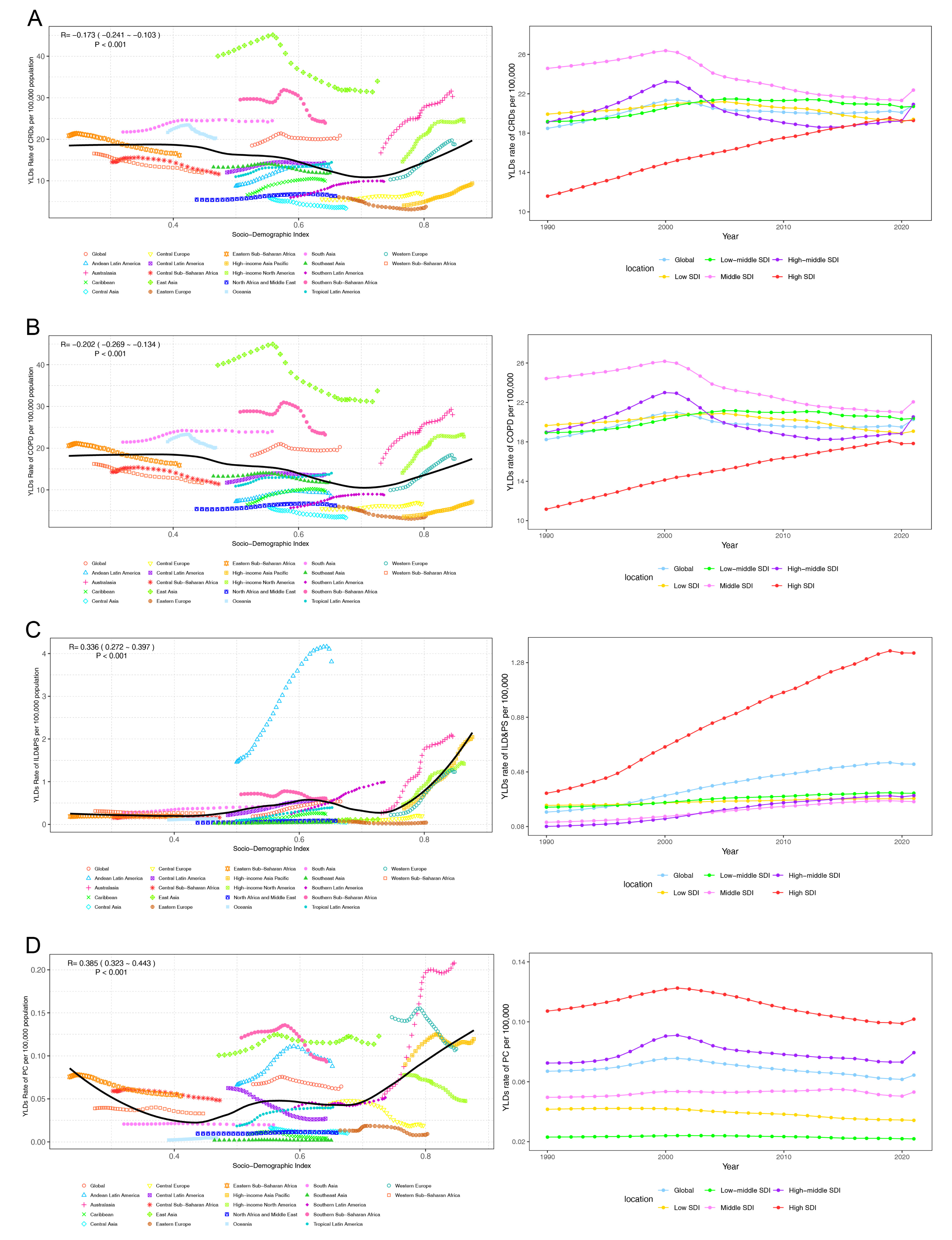

Supplement: S3 Fig — (A) CRDs; (B) COPD; (C) ILD&PS; (D) PC. (TIF) [file pone.0353177.s003.tif]

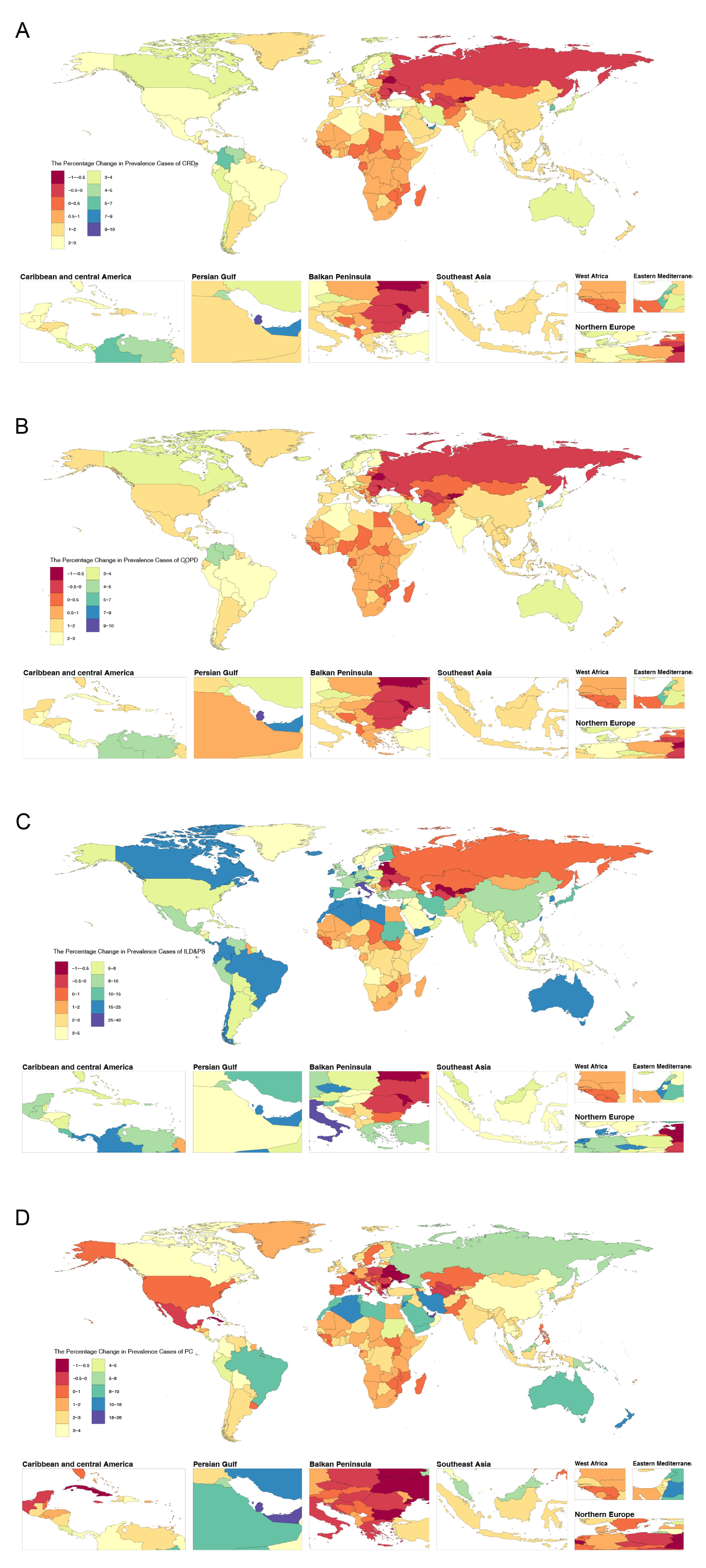

Supplement: S4 Fig — (A) CRDs; (B) COPD; (C) ILD&PS; (D) PC. (TIF) [file pone.0353177.s004.tif]

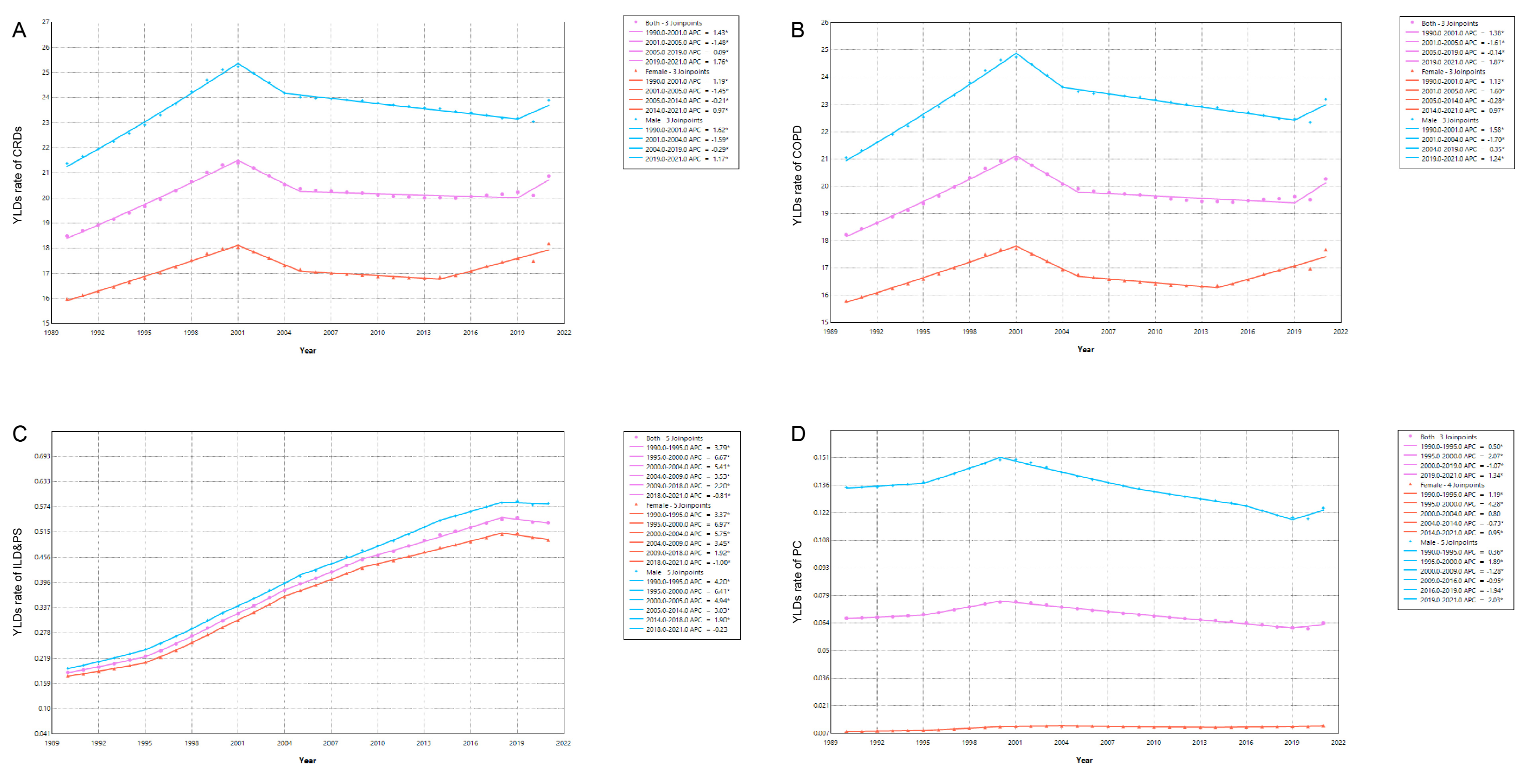

Supplement: S5 Fig — (A) CRDs; (B) COPD; (C) ILD&PS; (D) PC. (TIF) [file pone.0353177.s005.tif]

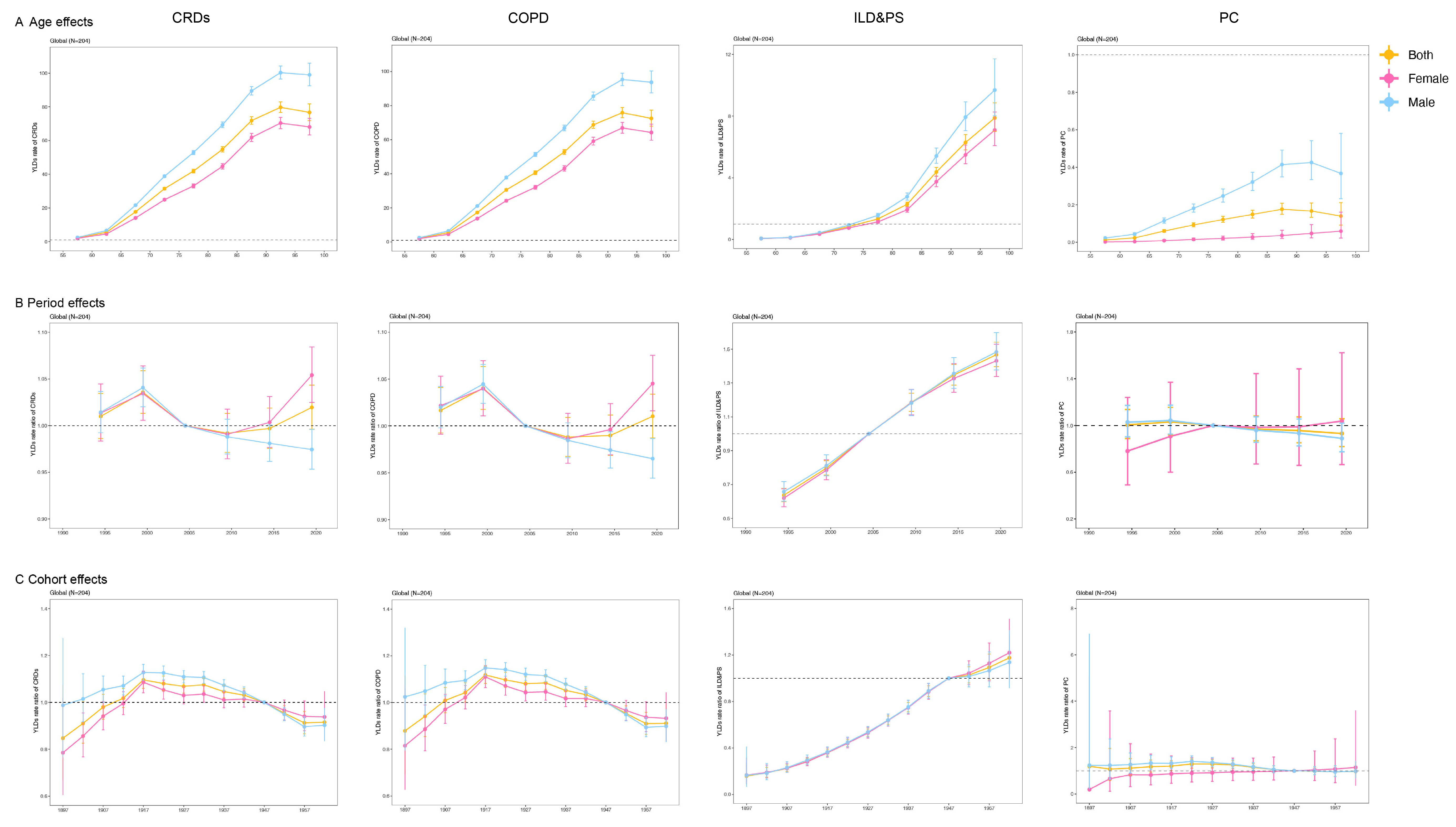

Supplement: S6 Fig — (A) Age effects; (B) Period effects; (C) Cohort effects. (TIF) [file pone.0353177.s006.tif]

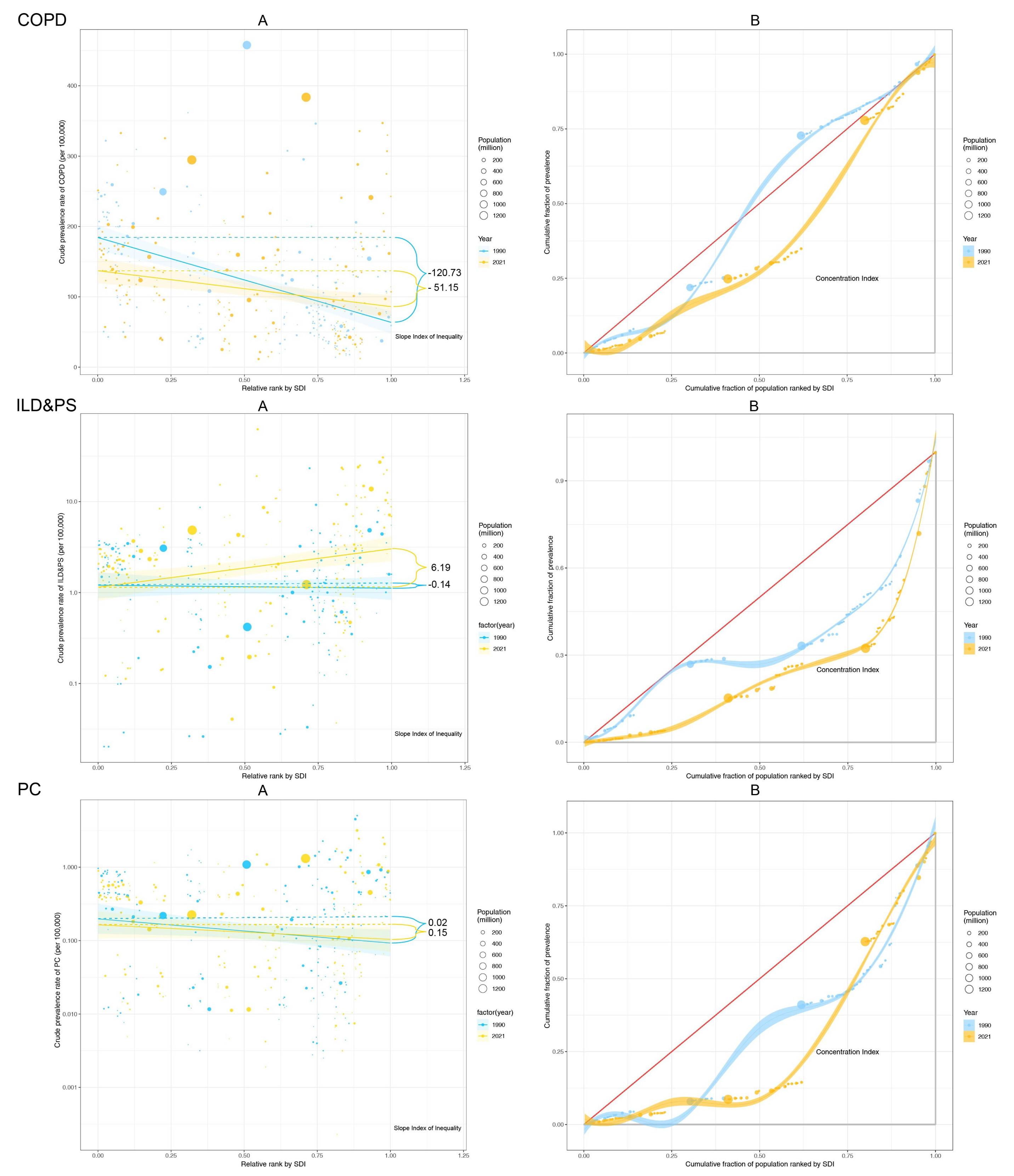

Supplement: S7 Fig — (A) Slope index of inequality; (B) Concentration index. (TIF) [file pone.0353177.s007.tif]
