# Supplementary material for: Global patterns and trends in heart failure burden under chronic respiratory disease cause categories among adults aged 55 years and older: A systematic analysis based on the GBD 2021 Study
Source: PLoS One. 2026 Jul 21;21(7):e0353177. doi: 10.1371/journal.pone.0353177 (PMC13387532; doi:10.1371/journal.pone.0353177)
Supplement: S1 File — (DOCX) [file pone.0353177.s008.docx]

**S1 Appendix. Supplementary tables**

**Table S1. YLDs, YLD rates, and EAPC trends of HF impairment under CRD cause categories from 1990 to 2021 among adults aged ≥55 years.**

| **Location** | **YLDs** | | | **YLD rates** | | |
| --- | --- | --- | --- | --- | --- | --- |
|  | **1990 (95% UI)** | **2021 (95% UI)** | **Relative change, 1990–2021(95% UI)** | **1990, per 100,000 (95% UI)** | **2021, per 100,000 (95% UI)** | **EAPC (95% CI)** |
| Global | 124104 (76528-183525) | 310171 (188619-469506) | 1.5 (1.46-1.56) | 18.48 (11.4-27.33) | 20.87 (12.69-31.6) | 0.13 (0.01-0.25) |
| Low SDI | 7435 (4444-11543) | 15918 (9373-25230) | 1.14 (1.11-1.19) | 19.93 (11.91-30.94) | 19.4 (11.42-30.75) | -0.14 (-0.24--0.04) |
| Low-middle SDI | 19331 (12036-29095) | 49952 (30345-77503) | 1.58 (1.52-1.66) | 19.18 (11.94-28.86) | 20.72 (12.59-32.15) | 0.31 (0.21-0.41) |
| Middle SDI | 42662 (26841-61797) | 105118 (63863-157836) | 1.46 (1.38-1.55) | 24.58 (15.46-35.61) | 22.37 (13.59-33.59) | -0.68 (-0.81--0.54) |
| High-middle SDI | 32997 (20385-47916) | 72511 (44137-109975) | 1.2 (1.17-1.3) | 19.13 (11.82-27.77) | 20.92 (12.73-31.72) | -0.34 (-0.58--0.11) |
| High SDI | 21620 (12891-32674) | 66541 (41145-100578) | 2.08 (2.19-2.08) | 11.59 (6.91-17.52) | 19.29 (11.93-29.15) | 1.69 (1.58-1.8) |
| Andean Latin America | 293 (178-420) | 1226 (739-1786) | 3.18 (3.15-3.25) | 8.72 (5.3-12.51) | 12.38 (7.46-18.03) | 1.66 (1.45-1.88) |
| Australasia | 658 (379-1000) | 2676 (1706-3696) | 3.07 (3.5-2.7) | 16.71 (9.62-25.38) | 30.29 (19.31-41.84) | 1.68 (1.49-1.86) |
| Caribbean | 284 (164-426) | 920 (553-1365) | 2.24 (2.37-2.2) | 6.6 (3.8-9.89) | 9.94 (5.98-14.74) | 1.42 (1.16-1.68) |
| Central Asia | 479 (274-733) | 490 (276-766) | 0.02 (0.01-0.05) | 5.99 (3.43-9.16) | 3.37 (1.9-5.27) | -1.84 (-1.96--1.72) |
| Central Europe | 1476 (842-2260) | 2538 (1499-3915) | 0.72 (0.78-0.73) | 5.57 (3.18-8.52) | 6.85 (4.05-10.57) | 0.87 (0.78-0.97) |
| Central Latin America | 1629 (1037-2360) | 6115 (3799-9121) | 2.75 (2.66-2.86) | 12.01 (7.65-17.39) | 14.3 (8.88-21.33) | 0.51 (0.37-0.65) |
| Central Sub-Saharan Africa | 545 (288-930) | 1048 (551-1785) | 0.92 (0.91-0.92) | 14.51 (7.66-24.73) | 11.61 (6.1-19.78) | -0.68 (-0.92--0.44) |
| East Asia | 59617 (37271-85566) | 133204 (79122-200897) | 1.23 (1.12-1.35) | 40.02 (25.02-57.44) | 33.97 (20.18-51.23) | -1.24 (-1.49--0.99) |
| Eastern Europe | 2959 (1676-4702) | 2339 (1276-3827) | -0.21 (-0.24--0.19) | 6.05 (3.43-9.62) | 3.77 (2.06-6.17) | -2.27 (-2.59--1.95) |
| Eastern Sub-Saharan Africa | 2536 (1382-4263) | 4362 (2382-7196) | 0.72 (0.72-0.69) | 20.84 (11.36-35.04) | 16.13 (8.81-26.61) | -0.97 (-1.07--0.86) |
| High-income Asia Pacific | 1415 (766-2358) | 6591 (3850-10436) | 3.66 (4.03-3.43) | 4.05 (2.19-6.74) | 9.35 (5.46-14.8) | 2.72 (2.63-2.81) |
| High-income North America | 8465 (4854-13376) | 27275 (16473-44363) | 2.22 (2.39-2.32) | 14.61 (8.38-23.09) | 24.24 (14.64-39.42) | 1.6 (1.36-1.84) |
| North Africa and Middle East | 1548 (949-2266) | 4836 (2898-7053) | 2.12 (2.05-2.11) | 5.48 (3.36-8.02) | 6.34 (3.8-9.25) | 0.86 (0.71-1.01) |
| Oceania | 104 (62-153) | 250 (151-365) | 1.4 (1.44-1.39) | 21.65 (12.91-31.9) | 20.28 (12.27-29.61) | -0.42 (-0.51--0.32) |
| South Asia | 20643 (12612-31325) | 60731 (36177-94367) | 1.94 (1.87-2.01) | 21.74 (13.28-32.99) | 24.46 (14.57-38.01) | 0.42 (0.33-0.52) |
| Southeast Asia | 5628 (3534-8343) | 13574 (8425-20234) | 1.41 (1.38-1.43) | 13.29 (8.35-19.7) | 11.85 (7.35-17.66) | -0.48 (-0.64--0.32) |
| Southern Latin America | 473 (274-711) | 1445 (838-2191) | 2.05 (2.06-2.08) | 5.96 (3.46-8.98) | 9.82 (5.7-14.89) | 1.75 (1.53-1.96) |
| Southern Sub-Saharan Africa | 1306 (740-2137) | 2328 (1285-3781) | 0.78 (0.74-0.77) | 29.52 (16.71-48.29) | 23.92 (13.2-38.83) | -0.67 (-0.93--0.41) |
| Tropical Latin America | 1664 (1005-2524) | 6398 (3793-10014) | 2.84 (2.77-2.97) | 10.99 (6.64-16.67) | 14.44 (8.56-22.61) | 0.79 (0.69-0.89) |
| Western Europe | 9989 (6028-14870) | 27983 (17582-40337) | 1.8 (1.92-1.71) | 10.29 (6.21-15.31) | 18.76 (11.79-27.05) | 2.34 (2.19-2.49) |
| Western Sub-Saharan Africa | 2392 (1306-4023) | 3838 (2090-6408) | 0.6 (0.6-0.59) | 16.57 (9.05-27.87) | 11.94 (6.5-19.94) | -1.08 (-1.12--1.05) |

**Table S2. Prevalent cases, prevalence rates, and EAPC trends of HF impairment under the COPD cause category from 1990 to 2021 among adults aged ≥55 years.**

| **Location** | **Prevalent cases** | | |  | **Prevalence rates** | | |  |
| --- | --- | --- | --- | --- | --- | --- | --- | --- |
|  | **1990 Cases (95% UI)** | **2021 Cases (95% UI)** | **Relative change, 1990– 2021(95% UI)** |  | **1990, per 100,000 (95% UI)** | **2021, per 100,000 (95% UI)** | **EAPC (95% CI)** |  |
| Global | 1379374 (1031197-1821883) | 3391662 (2461233-4556712) | 1.46 (1.39-1.5) |  | 205.44 (153.58-271.35) | 228.24 (165.63-306.65) | 0.06 (-0.06-0.19) |  |
| Low SDI | 83508 (57560-121667) | 178066 (122397-259666) | 1.13 (1.13-1.13) |  | 223.83 (154.28-326.11) | 217 (149.16-316.44) | -0.15 (-0.26--0.05) |  |
| Low-middle SDI | 218648 (166148-288240) | 560770 (409784-763794) | 1.56 (1.47-1.65) |  | 216.91 (164.83-285.95) | 232.6 (169.98-316.82) | 0.29 (0.18-0.39) |  |
| Middle SDI | 477768 (359223-627625) | 1168396 (843206-1589467) | 1.45 (1.35-1.53) |  | 275.27 (206.97-361.62) | 248.67 (179.46-338.29) | -0.71 (-0.85--0.57) |  |
| High-middle SDI | 367718 (275332-479233) | 798619 (581912-1084550) | 1.17 (1.11-1.26) |  | 213.14 (159.59-277.78) | 230.36 (167.85-312.84) | -0.4 (-0.64--0.16) |  |
| High SDI | 231082 (167298-315667) | 684383 (522199-886269) | 1.96 (2.12-1.81) |  | 123.93 (89.72-169.29) | 198.36 (151.36-256.88) | 1.55 (1.45-1.65) |  |
| Andean Latin America | 2736 (2104-3533) | 9472 (7218-12243) | 2.46 (2.43-2.47) |  | 81.53 (62.7-105.28) | 95.62 (72.86-123.58) | 0.93 (0.71-1.14) |  |
| Australasia | 7160 (5250-9520) | 27568 (22004-34280) | 2.85 (3.19-2.6) |  | 181.76 (133.27-241.65) | 312.06 (249.07-388.03) | 1.47 (1.29-1.65) |  |
| Caribbean | 3125 (2329-4114) | 10050 (7639-13043) | 2.22 (2.28-2.17) |  | 72.51 (54.04-95.47) | 108.54 (82.51-140.88) | 1.39 (1.12-1.65) |  |
| Central Asia | 5263 (3658-7269) | 5355 (3600-7529) | 0.02 (-0.02-0.04) |  | 65.8 (45.74-90.89) | 36.8 (24.74-51.74) | -1.87 (-1.99--1.75) |  |
| Central Europe | 16055 (11221-22397) | 27287 (19766-36211) | 0.7 (0.76-0.62) |  | 60.54 (42.31-84.45) | 73.69 (53.38-97.79) | 0.84 (0.75-0.94) |  |
| Central Latin America | 17956 (13552-23080) | 65753 (48833-86574) | 2.66 (2.6-2.75) |  | 132.32 (99.86-170.08) | 153.75 (114.19-202.44) | 0.43 (0.29-0.57) |  |
| Central Sub-Saharan Africa | 6023 (3506-9740) | 11531 (6872-18783) | 0.91 (0.96-0.93) |  | 160.18 (93.22-259.02) | 127.79 (76.16-208.15) | -0.69 (-0.94--0.45) |  |
| East Asia | 668632 (496250-872751) | 1486920 (1065954-2023589) | 1.22 (1.15-1.32) |  | 448.89 (333.16-585.92) | 379.2 (271.84-516.06) | -1.26 (-1.51--1.01) |  |
| Eastern Europe | 32582 (22198-46261) | 25657 (16803-37635) | -0.21 (-0.24--0.19) |  | 66.64 (45.4-94.62) | 41.33 (27.07-60.62) | -2.27 (-2.59--1.95) |  |
| Eastern Sub-Saharan Africa | 28274 (17192-43106) | 48411 (29781-74776) | 0.71 (0.73-0.73) |  | 232.41 (141.32-354.32) | 179.05 (110.15-276.56) | -0.98 (-1.09--0.88) |  |
| High-income Asia Pacific | 13874 (8477-20403) | 56555 (39993-78894) | 3.08 (3.72-2.87) |  | 39.67 (24.24-58.35) | 80.22 (56.73-111.9) | 2.28 (2.23-2.34) |  |
| High-income North America | 90302 (61570-125584) | 284370 (204814-390704) | 2.15 (2.33-2.11) |  | 155.89 (106.29-216.79) | 252.7 (182-347.19) | 1.52 (1.28-1.76) |  |
| North Africa and Middle East | 17206 (13283-21974) | 53320 (41108-68693) | 2.1 (2.09-2.13) |  | 60.88 (47-77.74) | 69.94 (53.92-90.11) | 0.83 (0.68-0.98) |  |
| Oceania | 1168 (871-1525) | 2804 (2111-3656) | 1.4 (1.42-1.4) |  | 242.69 (181-316.93) | 227.22 (171.03-296.26) | -0.42 (-0.53--0.32) |  |
| South Asia | 234454 (175743-308223) | 683475 (488714-945444) | 1.92 (1.78-2.07) |  | 246.94 (185.1-324.64) | 275.27 (196.83-380.77) | 0.39 (0.3-0.49) |  |
| Southeast Asia | 63395 (48556-83229) | 152492 (116338-200049) | 1.41 (1.4-1.4) |  | 149.72 (114.68-196.57) | 133.12 (101.56-174.63) | -0.49 (-0.65--0.33) |  |
| Southern Latin America | 5008 (3497-6706) | 14408 (9853-19749) | 1.88 (1.82-1.94) |  | 63.22 (44.14-84.65) | 97.9 (66.95-134.2) | 1.56 (1.34-1.78) |  |
| Southern Sub-Saharan Africa | 14240 (9076-20925) | 25250 (15957-37872) | 0.77 (0.76-0.81) |  | 321.83 (205.11-472.9) | 259.37 (163.9-389.02) | -0.69 (-0.95--0.43) |  |
| Tropical Latin America | 18606 (13592-24953) | 70009 (49734-98031) | 2.76 (2.66-2.93) |  | 122.88 (89.77-164.8) | 158.04 (112.27-221.3) | 0.71 (0.61-0.82) |  |
| Western Europe | 106889 (76874-143311) | 288941 (221490-367620) | 1.7 (1.88-1.57) |  | 110.07 (79.16-147.57) | 193.75 (148.52-246.5) | 2.19 (2.06-2.33) |  |
| Western Sub-Saharan Africa | 26427 (16088-40815) | 42036 (25056-66396) | 0.59 (0.56-0.63) |  | 183.07 (111.45-282.74) | 130.78 (77.95-206.56) | -1.11 (-1.14--1.07) |  |

**Table S3. YLDs, YLD rates, and EAPC trends of HF impairment under the COPD cause category from 1990 to 2021 among adults aged ≥55 years.**

| **Location** | **YLDs** | |  | **YLD rates** | |  |
| --- | --- | --- | --- | --- | --- | --- |
|  | **1990 (95% UI)** | **2021 (95% UI)** | **Relative change, 1990–2021(95% UI)** | **1990, per 100,000 (95% UI)** | **2021, per 100,000 (95% UI)** | **EAPC (95% CI)** |
| Global | 122406 (75541-180860) | 301242 (183173-455536) | 1.46 (1.42-1.52) | 18.23 (11.25-26.94) | 20.27 (12.33-30.66) | 0.07 (-0.06-0.19) |
| Low SDI | 7332 (4377-11384) | 15655 (9209-24825) | 1.14 (1.1-1.18) | 19.65 (11.73-30.51) | 19.08 (11.22-30.25) | -0.15 (-0.25--0.05) |
| Low-middle SDI | 19084 (11893-28728) | 49118 (29826-76210) | 1.57 (1.51-1.65) | 18.93 (11.8-28.5) | 20.37 (12.37-31.61) | 0.3 (0.2-0.4) |
| Middle SDI | 42383 (26662-61361) | 103637 (62914-155618) | 1.45 (1.36-1.54) | 24.42 (15.36-35.35) | 22.06 (13.39-33.12) | -0.71 (-0.84--0.57) |
| High-middle SDI | 32733 (20234-47503) | 71173 (43295-107983) | 1.17 (1.14-1.27) | 18.97 (11.73-27.53) | 20.53 (12.49-31.15) | -0.39 (-0.63--0.15) |
| High SDI | 20817 (12434-31427) | 61531 (38064-92795) | 1.96 (2.06-1.95) | 11.16 (6.67-16.85) | 17.83 (11.03-26.9) | 1.55 (1.44-1.65) |
| Andean Latin America | 242 (146-347) | 840 (523-1240) | 2.47 (2.58-2.57) | 7.2 (4.35-10.35) | 8.48 (5.28-12.51) | 0.93 (0.72-1.14) |
| Australasia | 646 (371-981) | 2475 (1574-3411) | 2.83 (3.24-2.48) | 16.39 (9.42-24.89) | 28.02 (17.81-38.62) | 1.45 (1.27-1.63) |
| Caribbean | 280 (161-420) | 898 (539-1329) | 2.21 (2.35-2.16) | 6.5 (3.74-9.74) | 9.7 (5.82-14.36) | 1.37 (1.11-1.64) |
| Central Asia | 470 (269-719) | 481 (271-753) | 0.02 (0.01-0.05) | 5.87 (3.36-8.99) | 3.31 (1.86-5.17) | -1.85 (-1.97--1.73) |
| Central Europe | 1447 (826-2216) | 2458 (1453-3790) | 0.7 (0.76-0.71) | 5.46 (3.11-8.35) | 6.64 (3.92-10.23) | 0.84 (0.74-0.93) |
| Central Latin America | 1592 (1015-2300) | 5846 (3632-8717) | 2.67 (2.58-2.79) | 11.73 (7.48-16.95) | 13.67 (8.49-20.38) | 0.43 (0.29-0.58) |
| Central Sub-Saharan Africa | 537 (284-916) | 1029 (540-1753) | 0.92 (0.9-0.91) | 14.29 (7.54-24.35) | 11.4 (5.99-19.43) | -0.69 (-0.93--0.45) |
| East Asia | 59409 (37146-85252) | 132254 (78615-199411) | 1.23 (1.12-1.34) | 39.88 (24.94-57.23) | 33.73 (20.05-50.85) | -1.26 (-1.5--1.01) |
| Eastern Europe | 2918 (1654-4639) | 2307 (1258-3776) | -0.21 (-0.24--0.19) | 5.97 (3.38-9.49) | 3.72 (2.03-6.08) | -2.26 (-2.58--1.95) |
| Eastern Sub-Saharan Africa | 2504 (1365-4213) | 4297 (2344-7093) | 0.72 (0.72-0.68) | 20.58 (11.22-34.63) | 15.89 (8.67-26.23) | -0.97 (-1.08--0.87) |
| High-income Asia Pacific | 1254 (674-2086) | 5065 (2978-7983) | 3.04 (3.42-2.83) | 3.59 (1.93-5.97) | 7.18 (4.22-11.32) | 2.26 (2.2-2.31) |
| High-income North America | 8154 (4676-12888) | 25628 (15488-41714) | 2.14 (2.31-2.24) | 14.08 (8.07-22.25) | 22.77 (13.76-37.07) | 1.51 (1.28-1.74) |
| North Africa and Middle East | 1533 (940-2245) | 4763 (2855-6942) | 2.11 (2.04-2.09) | 5.42 (3.33-7.94) | 6.25 (3.75-9.11) | 0.84 (0.68-0.99) |
| Oceania | 104 (62-153) | 249 (150-363) | 1.39 (1.42-1.37) | 21.54 (12.84-31.74) | 20.14 (12.19-29.41) | -0.42 (-0.52--0.32) |
| South Asia | 20358 (12435-30896) | 59642 (35578-92691) | 1.93 (1.86-2) | 21.44 (13.1-32.54) | 24.02 (14.33-37.33) | 0.41 (0.31-0.51) |
| Southeast Asia | 5617 (3527-8326) | 13522 (8394-20158) | 1.41 (1.38-1.42) | 13.27 (8.33-19.66) | 11.8 (7.33-17.6) | -0.48 (-0.64--0.32) |
| Southern Latin America | 450 (260-677) | 1291 (751-1965) | 1.87 (1.89-1.9) | 5.68 (3.29-8.55) | 8.78 (5.1-13.36) | 1.55 (1.33-1.77) |
| Southern Sub-Saharan Africa | 1270 (718-2084) | 2260 (1246-3679) | 0.78 (0.74-0.77) | 28.7 (16.23-47.11) | 23.22 (12.8-37.79) | -0.68 (-0.93--0.41) |
| Tropical Latin America | 1647 (995-2497) | 6206 (3679-9719) | 2.77 (2.7-2.89) | 10.87 (6.57-16.49) | 14.01 (8.31-21.94) | 0.71 (0.61-0.81) |
| Western Europe | 9634 (5815-14351) | 25987 (16251-37277) | 1.7 (1.79-1.6) | 9.92 (5.99-14.78) | 17.43 (10.9-25) | 2.19 (2.05-2.33) |
| Western Sub-Saharan Africa | 2341 (1275-3946) | 3744 (2033-6279) | 0.6 (0.59-0.59) | 16.22 (8.83-27.33) | 11.65 (6.32-19.54) | -1.09 (-1.13--1.06) |

**Table S4. Prevalent cases, prevalence rates, and EAPC trends of HF impairment under the ILD&PS cause category from 1990 to 2021 among adults aged ≥55 years.**

| **Location** | **Prevalent cases** | | | **Relative change, 1990–2021(95% UI)** | **Prevalence rates** | | **EAPC (95% CI)** |
| --- | --- | --- | --- | --- | --- | --- | --- |
|  | **1990  (95% UI)** | | **2021  (95% UI)** |  | **1990, per 100,000 (95% UI)** | **2021, per 100,000 (95% UI)** |  |
| Global | 13479 (9946-18241) | 87369 (65773-114641) | | 5.48 (5.61-5.28) | 2.01 (1.48-2.72) | 5.88 (4.43-7.71) | 3.93 (3.6-4.26) |
| Low SDI | 943 (656-1304) | 2525 (1741-3579) | | 1.68 (1.65-1.74) | 2.53 (1.76-3.5) | 3.08 (2.12-4.36) | 0.7 (0.65-0.75) |
| Low-middle SDI | 2412 (1838-3143) | 8470 (6210-11463) | | 2.51 (2.38-2.65) | 2.39 (1.82-3.12) | 3.51 (2.58-4.75) | 1.45 (1.35-1.55) |
| Middle SDI | 2102 (1575-2729) | 13480 (10134-18078) | | 5.41 (5.43-5.62) | 1.21 (0.91-1.57) | 2.87 (2.16-3.85) | 3.31 (3.1-3.52) |
| High-middle SDI | 1503 (1100-2022) | 11621 (8970-14811) | | 6.73 (7.15-6.32) | 0.87 (0.64-1.17) | 3.35 (2.59-4.27) | 5.06 (4.63-5.48) |
| High SDI | 6512 (4503-9075) | 51232 (37647-67592) | | 6.87 (7.36-6.45) | 3.49 (2.41-4.87) | 14.85 (10.91-19.59) | 5.06 (4.65-5.48) |
| Andean Latin America | 545 (429-684) | 4246 (3337-5428) | | 6.79 (6.78-6.94) | 16.25 (12.77-20.38) | 42.86 (33.69-54.79) | 4.12 (3.8-4.44) |
| Australasia | 113 (84-151) | 2007 (1575-2533) | | 16.76 (17.75-15.77) | 2.87 (2.13-3.83) | 22.72 (17.83-28.68) | 7.16 (6.2-8.12) |
| Caribbean | 42 (31-56) | 242 (181-321) | | 4.76 (4.84-4.73) | 0.98 (0.73-1.31) | 2.61 (1.95-3.47) | 4.08 (3.69-4.46) |
| Central Asia | 84 (59-117) | 82 (56-114) | | -0.02 (-0.05--0.03) | 1.05 (0.74-1.46) | 0.56 (0.38-0.78) | -1.42 (-1.66--1.17) |
| Central Europe | 201 (140-282) | 784 (576-1057) | | 2.9 (3.11-2.75) | 0.76 (0.53-1.06) | 2.12 (1.56-2.85) | 3.86 (3.61-4.1) |
| Central Latin America | 317 (237-423) | 2799 (2037-3787) | | 7.83 (7.59-7.95) | 2.33 (1.75-3.12) | 6.54 (4.76-8.86) | 3.55 (3.3-3.8) |
| Central Sub-Saharan Africa | 65 (38-104) | 157 (93-251) | | 1.42 (1.45-1.41) | 1.72 (1-2.75) | 1.75 (1.04-2.79) | 0.11 (0-0.23) |
| East Asia | 625 (444-868) | 5050 (3547-7446) | | 7.08 (6.99-7.58) | 0.42 (0.3-0.58) | 1.29 (0.9-1.9) | 3.96 (3.76-4.16) |
| Eastern Europe | 364 (255-509) | 284 (182-426) | | -0.22 (-0.29--0.16) | 0.75 (0.52-1.04) | 0.46 (0.29-0.69) | -3.09 (-4.28--1.88) |
| Eastern Sub-Saharan Africa | 242 (148-363) | 552 (339-839) | | 1.28 (1.29-1.31) | 1.99 (1.22-2.98) | 2.04 (1.25-3.1) | 0.03 (-0.04-0.1) |
| High-income Asia Pacific | 1405 (866-2022) | 15941 (10701-22379) | | 10.35 (11.36-10.07) | 4.02 (2.48-5.78) | 22.61 (15.18-31.74) | 5.41 (5-5.83) |
| High-income North America | 2863 (1960-3961) | 17541 (12797-23902) | | 5.13 (5.53-5.03) | 4.94 (3.38-6.84) | 15.59 (11.37-21.24) | 3.93 (3.49-4.37) |
| North Africa and Middle East | 126 (98-161) | 703 (544-900) | | 4.58 (4.55-4.59) | 0.44 (0.35-0.57) | 0.92 (0.71-1.18) | 3.02 (2.82-3.22) |
| Oceania | 6 (4-7) | 17 (13-23) | | 1.83 (2.25-2.29) | 1.14 (0.86-1.5) | 1.41 (1.06-1.84) | 0.58 (0.5-0.65) |
| South Asia | 2868 (2152-3753) | 11274 (8141-15623) | | 2.93 (2.78-3.16) | 3.02 (2.27-3.95) | 4.54 (3.28-6.29) | 1.47 (1.38-1.56) |
| Southeast Asia | 113 (88-147) | 541 (416-702) | | 3.79 (3.73-3.78) | 0.27 (0.21-0.35) | 0.47 (0.36-0.61) | 2.01 (1.94-2.09) |
| Southern Latin America | 213 (151-284) | 1599 (1125-2190) | | 6.51 (6.45-6.71) | 2.69 (1.9-3.59) | 10.87 (7.65-14.88) | 4.56 (4.3-4.82) |
| Southern Sub-Saharan Africa | 338 (228-470) | 637 (417-883) | | 0.88 (0.83-0.88) | 7.63 (5.16-10.62) | 6.54 (4.28-9.07) | -0.55 (-0.81--0.28) |
| Tropical Latin America | 154 (111-211) | 1887 (1311-2662) | | 11.25 (10.81-11.62) | 1.01 (0.73-1.4) | 4.26 (2.96-6.01) | 5.29 (4.93-5.66) |
| Western Europe | 2309 (1664-3098) | 20127 (15705-25803) | | 7.72 (8.44-7.33) | 2.38 (1.71-3.19) | 13.5 (10.53-17.3) | 6.72 (6.13-7.32) |
| Western Sub-Saharan Africa | 487 (304-713) | 899 (564-1365) | | 0.85 (0.86-0.91) | 3.37 (2.1-4.94) | 2.8 (1.75-4.25) | -0.67 (-0.77--0.57) |

**Table S5. YLDs, YLD rates, and EAPC trends of HF impairment under the ILD&PS cause category from 1990 to 2021 among adults aged ≥55 years.**

| **Location** | **YLDs** | |  | **YLD rates** | |  |
| --- | --- | --- | --- | --- | --- | --- |
|  | **1990 (95% UI)** | **2021 (95% UI)** | **Relative change, 1990–2021(95% UI)** | **1990, per 100,000 (95% UI)** | **2021, per 100,000 (95% UI)** | **EAPC (95% CI)** |
| Global | 1248 (749-1899) | 7972 (4905-12348) | 5.39 (5.55-5.5) | 0.19 (0.11-0.28) | 0.54 (0.33-0.83) | 3.87 (3.54-4.2) |
| Low SDI | 88 (53-135) | 234 (139-364) | 1.66 (1.62-1.7) | 0.23 (0.14-0.36) | 0.29 (0.17-0.44) | 0.7 (0.65-0.75) |
| Low-middle SDI | 223 (138-339) | 781 (474-1208) | 2.5 (2.43-2.56) | 0.22 (0.14-0.34) | 0.32 (0.2-0.5) | 1.43 (1.33-1.53) |
| Middle SDI | 193 (122-289) | 1231 (759-1873) | 5.38 (5.22-5.48) | 0.11 (0.07-0.17) | 0.26 (0.16-0.4) | 3.28 (3.08-3.49) |
| High-middle SDI | 139 (83-210) | 1063 (652-1612) | 6.65 (6.86-6.68) | 0.08 (0.05-0.12) | 0.31 (0.19-0.46) | 5 (4.57-5.43) |
| High SDI | 604 (345-941) | 4659 (2793-7307) | 6.71 (7.1-6.77) | 0.32 (0.19-0.5) | 1.35 (0.81-2.12) | 4.99 (4.57-5.41) |
| Andean Latin America | 49 (30-73) | 378 (232-563) | 6.71 (6.73-6.71) | 1.46 (0.89-2.17) | 3.81 (2.34-5.68) | 4.08 (3.77-4.4) |
| Australasia | 10 (6-16) | 182 (106-280) | 17.2 (16.67-16.5) | 0.27 (0.16-0.4) | 2.06 (1.2-3.17) | 7.03 (6.08-8) |
| Caribbean | 4 (2-6) | 22 (14-33) | 4.5 (6-4.5) | 0.09 (0.05-0.14) | 0.24 (0.15-0.36) | 4.04 (3.65-4.43) |
| Central Asia | 8 (4-12) | 8 (4-12) | 0 (0-0) | 0.1 (0.06-0.15) | 0.05 (0.03-0.08) | -1.41 (-1.66--1.17) |
| Central Europe | 19 (11-29) | 73 (43-112) | 2.84 (2.91-2.86) | 0.07 (0.04-0.11) | 0.2 (0.12-0.3) | 3.86 (3.61-4.1) |
| Central Latin America | 29 (18-44) | 258 (157-396) | 7.9 (7.72-8) | 0.22 (0.13-0.33) | 0.6 (0.37-0.93) | 3.52 (3.27-3.77) |
| Central Sub-Saharan Africa | 6 (3-10) | 15 (8-25) | 1.5 (1.67-1.5) | 0.16 (0.08-0.27) | 0.16 (0.09-0.28) | 0.11 (0-0.23) |
| East Asia | 58 (34-92) | 469 (270-758) | 7.09 (6.94-7.24) | 0.04 (0.02-0.06) | 0.12 (0.07-0.19) | 3.96 (3.76-4.16) |
| Eastern Europe | 34 (20-53) | 26 (14-43) | -0.24 (-0.3--0.19) | 0.07 (0.04-0.11) | 0.04 (0.02-0.07) | -3.09 (-4.28--1.88) |
| Eastern Sub-Saharan Africa | 22 (12-37) | 51 (28-83) | 1.32 (1.33-1.24) | 0.18 (0.1-0.3) | 0.19 (0.1-0.31) | 0.03 (-0.04-0.1) |
| High-income Asia Pacific | 130 (68-214) | 1442 (796-2306) | 10.09 (10.71-9.78) | 0.37 (0.19-0.61) | 2.05 (1.13-3.27) | 5.34 (4.93-5.75) |
| High-income North America | 265 (151-421) | 1594 (940-2531) | 5.02 (5.23-5.01) | 0.46 (0.26-0.73) | 1.42 (0.84-2.25) | 3.84 (3.41-4.28) |
| North Africa and Middle East | 12 (7-17) | 65 (39-95) | 4.42 (4.57-4.59) | 0.04 (0.02-0.06) | 0.09 (0.05-0.12) | 3.01 (2.81-3.22) |
| Oceania | 1 (0-1) | 2 (1-2) | 1 (0-1) | 0.11 (0.06-0.16) | 0.13 (0.08-0.19) | 0.58 (0.51-0.65) |
| South Asia | 266 (160-406) | 1040 (612-1628) | 2.91 (2.83-3.01) | 0.28 (0.17-0.43) | 0.42 (0.25-0.66) | 1.45 (1.36-1.54) |
| Southeast Asia | 11 (6-16) | 50 (30-74) | 3.55 (4-3.62) | 0.02 (0.02-0.04) | 0.04 (0.03-0.06) | 2 (1.93-2.08) |
| Southern Latin America | 20 (11-30) | 146 (85-221) | 6.3 (6.73-6.37) | 0.25 (0.14-0.38) | 0.99 (0.58-1.5) | 4.5 (4.23-4.76) |
| Southern Sub-Saharan Africa | 31 (18-48) | 59 (35-92) | 0.9 (0.94-0.92) | 0.7 (0.42-1.09) | 0.61 (0.36-0.95) | -0.53 (-0.8--0.27) |
| Tropical Latin America | 14 (8-22) | 175 (103-280) | 11.5 (11.88-11.73) | 0.09 (0.06-0.15) | 0.39 (0.23-0.63) | 5.29 (4.92-5.65) |
| Western Europe | 214 (130-319) | 1835 (1186-2728) | 7.57 (8.12-7.55) | 0.22 (0.13-0.33) | 1.23 (0.8-1.83) | 6.65 (6.06-7.25) |
| Western Sub-Saharan Africa | 45 (26-72) | 83 (45-138) | 0.84 (0.73-0.92) | 0.31 (0.18-0.5) | 0.26 (0.14-0.43) | -0.66 (-0.76--0.57) |

**Table S6. Prevalent cases, prevalence rates, and EAPC trends of HF impairment under the PC cause category from 1990 to 2021 among adults aged ≥55 years.**

| **Location** | **Prevalent cases** | |  | **Prevalence rates** | |  |
| --- | --- | --- | --- | --- | --- | --- |
|  | **1990 (95% UI)** | **2021 (95% UI)** | **Relative change, 1990–2021(95% UI)** | **1990, per 100,000 (95% UI)** | **2021, per 100,000 (95% UI)** | **EAPC (95% CI)** |
| Global | 4855 (3598-6491) | 10317 (7672-13712) | 1.13 (1.13-1.11) | 0.72 (0.54-0.97) | 0.69 (0.52-0.92) | -0.37 (-0.54--0.19) |
| Low SDI | 167 (119-222) | 303 (216-405) | 0.81 (0.82-0.82) | 0.45 (0.32-0.59) | 0.37 (0.26-0.49) | -0.76 (-0.85--0.68) |
| Low-middle SDI | 250 (196-320) | 569 (435-744) | 1.28 (1.22-1.32) | 0.25 (0.19-0.32) | 0.24 (0.18-0.31) | -0.2 (-0.28--0.11) |
| Middle SDI | 928 (686-1261) | 2688 (1940-3724) | 1.9 (1.83-1.95) | 0.53 (0.4-0.73) | 0.57 (0.41-0.79) | 0.19 (0.08-0.29) |
| High-middle SDI | 1349 (957-1848) | 2967 (2136-4209) | 1.2 (1.23-1.28) | 0.78 (0.55-1.07) | 0.86 (0.62-1.21) | -0.1 (-0.36-0.16) |
| High SDI | 2157 (1557-2902) | 3787 (2910-4842) | 0.76 (0.87-0.67) | 1.16 (0.84-1.56) | 1.1 (0.84-1.4) | -0.46 (-0.66--0.26) |
| Andean Latin America | 24 (19-31) | 94 (75-118) | 2.92 (2.95-2.81) | 0.71 (0.55-0.92) | 0.95 (0.76-1.19) | 1.52 (1.11-1.93) |
| Australasia | 22 (16-29) | 198 (154-254) | 8 (8.62-7.76) | 0.55 (0.41-0.73) | 2.24 (1.74-2.87) | 4.96 (4.21-5.71) |
| Caribbean | 4 (3-5) | 4 (3-5) | 0 (0-0) | 0.09 (0.07-0.12) | 0.05 (0.04-0.06) | -3.58 (-4.12--3.05) |
| Central Asia | 14 (10-20) | 16 (10-23) | 0.14 (0-0.15) | 0.18 (0.12-0.24) | 0.11 (0.07-0.16) | -1.17 (-1.44--0.9) |
| Central Europe | 115 (81-158) | 76 (55-102) | -0.34 (-0.32--0.35) | 0.43 (0.31-0.6) | 0.21 (0.15-0.27) | -3.67 (-4.25--3.08) |
| Central Latin America | 92 (68-122) | 124 (93-163) | 0.35 (0.37-0.34) | 0.67 (0.5-0.9) | 0.29 (0.22-0.38) | -3.38 (-3.59--3.17) |
| Central Sub-Saharan Africa | 24 (14-36) | 47 (29-70) | 0.96 (1.07-0.94) | 0.64 (0.38-0.96) | 0.52 (0.32-0.78) | -0.66 (-0.81--0.5) |
| East Asia | 1614 (1141-2230) | 5189 (3628-7473) | 2.21 (2.18-2.35) | 1.08 (0.77-1.5) | 1.32 (0.93-1.91) | 0.4 (0.21-0.6) |
| Eastern Europe | 70 (47-101) | 62 (40-92) | -0.11 (-0.15--0.09) | 0.14 (0.1-0.21) | 0.1 (0.06-0.15) | -1.89 (-2.82--0.96) |
| Eastern Sub-Saharan Africa | 99 (62-142) | 155 (101-220) | 0.57 (0.63-0.55) | 0.81 (0.51-1.16) | 0.57 (0.37-0.81) | -1.41 (-1.54--1.28) |
| High-income Asia Pacific | 340 (208-477) | 910 (675-1213) | 1.68 (2.25-1.54) | 0.97 (0.59-1.36) | 1.29 (0.96-1.72) | 0.3 (0.05-0.55) |
| High-income North America | 487 (304-711) | 575 (400-782) | 0.18 (0.32-0.1) | 0.84 (0.53-1.23) | 0.51 (0.36-0.69) | -1.83 (-2.02--1.64) |
| North Africa and Middle East | 29 (23-37) | 87 (68-110) | 2 (1.96-1.97) | 0.1 (0.08-0.13) | 0.11 (0.09-0.14) | 0.68 (0.52-0.83) |
| Oceania | 0 (0-0) | 1 (0-1) | 1(0-1) | 0.02 (0.02-0.03) | 0.05 (0.04-0.07) | 2.52 (2.29-2.75) |
| South Asia | 212 (161-276) | 531 (392-723) | 1.5 (1.43-1.62) | 0.22 (0.17-0.29) | 0.21 (0.16-0.29) | -0.16 (-0.23--0.09) |
| Southeast Asia | 9 (7-11) | 23 (18-30) | 1.56 (1.57-1.73) | 0.02 (0.02-0.03) | 0.02 (0.02-0.03) | -0.37 (-0.57--0.18) |
| Southern Latin America | 32 (23-44) | 82 (57-109) | 1.56 (1.48-1.48) | 0.41 (0.29-0.56) | 0.55 (0.39-0.74) | 0.44 (0.26-0.62) |
| Southern Sub-Saharan Africa | 58 (42-74) | 98 (70-127) | 0.69 (0.67-0.72) | 1.31 (0.94-1.68) | 1.01 (0.72-1.31) | -1.04 (-1.35--0.73) |
| Tropical Latin America | 30 (22-41) | 193 (135-272) | 5.43 (5.14-5.63) | 0.2 (0.15-0.27) | 0.43 (0.31-0.61) | 2.55 (2.1-3.01) |
| Western Europe | 1520 (1107-200 2) | 1738 (1376-2193) | 0.14 (0.24-0.1) | 1.56 (1.14-2.06) | 1.17 (0.92-1.47) | -1.08 (-1.31--0.84) |
| Western Sub-Saharan Africa | 61 (42-84) | 114 (80-155) | 0.87 (0.9-0.85) | 0.42 (0.29-0.58) | 0.35 (0.25-0.48) | -0.53 (-0.68--0.38) |

**Table S7. YLDs, YLD rates, and EAPC trends of HF impairment under the PC cause category from 1990 to 2021 among adults aged ≥55 years.**

| **Location** | **YLDs** | |  | **YLD rates** | |  |
| --- | --- | --- | --- | --- | --- | --- |
|  | **1990 (95% UI)** | **2021 (95% UI)** | **Relative change, 1990–2021(95% UI)** | **1990, per 100,000 (95% UI)** | **2021, per 100,000 (95% UI)** | **EAPC (95% CI)** |
| Global | 450 (272-688) | 957 (594-1477) | 1.13 (1.18-1.15) | 0.07 (0.04-0.1) | 0.06 (0.04-0.1) | -0.36 (-0.54--0.19) |
| Low SDI | 16 (9-24) | 28 (17-43) | 0.75 (0.89-0.79) | 0.04 (0.03-0.06) | 0.03 (0.02-0.05) | -0.76 (-0.85--0.68) |
| Low-middle SDI | 23 (15-35) | 53 (33-80) | 1.3 (1.2-1.29) | 0.02 (0.01-0.03) | 0.02 (0.01-0.03) | -0.2 (-0.28--0.11) |
| Middle SDI | 86 (52-135) | 249 (150-395) | 1.9 (1.88-1.93) | 0.05 (0.03-0.08) | 0.05 (0.03-0.08) | 0.19 (0.08-0.29) |
| High-middle SDI | 125 (72-194) | 275 (162-434) | 1.2 (1.25-1.24) | 0.07 (0.04-0.11) | 0.08 (0.05-0.13) | -0.09 (-0.35-0.17) |
| High SDI | 200 (118-302) | 351 (217-530) | 0.76 (0.84-0.75) | 0.11 (0.06-0.16) | 0.1 (0.06-0.15) | -0.46 (-0.66--0.26) |
| Andean Latin America | 2 (1-3) | 9 (6-12) | 3.5 (5-3) | 0.07 (0.04-0.1) | 0.09 (0.06-0.13) | 1.52 (1.11-1.93) |
| Australasia | 2 (1-3) | 18 (12-27) | 8 (11-8) | 0.05 (0.03-0.08) | 0.21 (0.13-0.3) | 4.95 (4.2-5.71) |
| Caribbean | 0 (0-1) | 0 (0-1) | 0 (0-1) | 0.01 (0.01-0.01) | 0 (0-0.01) | -3.59 (-4.12--3.05) |
| Central Asia | 1 (1-2) | 1 (1-2) | 0 (0-0) | 0.02 (0.01-0.03) | 0.01 (0.01-0.02) | -1.17 (-1.44--0.9) |
| Central Europe | 11 (6-16) | 7 (4-11) | -0.36 (-0.33--0.31) | 0.04 (0.02-0.06) | 0.02 (0.01-0.03) | -3.67 (-4.25--3.08) |
| Central Latin America | 8 (5-13) | 12 (7-17) | 0.5 (0.4-0.31) | 0.06 (0.04-0.1) | 0.03 (0.02-0.04) | -3.38 (-3.59--3.17) |
| Central Sub-Saharan Africa | 2 (1-4) | 4 (2-7) | 1 (1-0.75) | 0.06 (0.03-0.1) | 0.05 (0.03-0.08) | -0.66 (-0.82--0.5) |
| East Asia | 150 (88-238) | 482 (281-771) | 2.21 (2.19-2.24) | 0.1 (0.06-0.16) | 0.12 (0.07-0.2) | 0.41 (0.21-0.6) |
| Eastern Europe | 6 (4-10) | 6 (3-9) | 0 (-0.25--0.1) | 0.01 (0.01-0.02) | 0.01 (0-0.02) | -1.89 (-2.81--0.96) |
| Eastern Sub-Saharan Africa | 9 (5-15) | 14 (8-24) | 0.56 (0.6-0.6) | 0.08 (0.04-0.12) | 0.05 (0.03-0.09) | -1.41 (-1.55--1.28) |
| High-income Asia Pacific | 31 (17-49) | 84 (50-128) | 1.71 (1.94-1.61) | 0.09 (0.05-0.14) | 0.12 (0.07-0.18) | 0.3 (0.05-0.55) |
| High-income North America | 45 (23-71) | 53 (30-86) | 0.18 (0.3-0.21) | 0.08 (0.04-0.12) | 0.05 (0.03-0.08) | -1.82 (-2.01--1.63) |
| North Africa and Middle East | 3 (2-4) | 8 (5-12) | 1.67 (1.5-2) | 0.01 (0.01-0.01) | 0.01 (0.01-0.02) | 0.68 (0.53-0.83) |
| Oceania | 0 (0-0) | 0 (0-0) | 0 (0-0) | 0 (0-0) | 0 (0-0.01) | 2.52 (2.29-2.75) |
| South Asia | 20 (12-30) | 49 (29-77) | 1.45 (1.42-1.57) | 0.02 (0.01-0.03) | 0.02 (0.01-0.03) | -0.16 (-0.23--0.09) |
| Southeast Asia | 1 (1-1) | 2 (1-3) | 1 (0-2) | 0 (0-0) | 0 (0-0) | -0.37 (-0.57--0.18) |
| Southern Latin America | 3 (2-5) | 8 (4-11) | 1.67 (1-1.2) | 0.04 (0.02-0.06) | 0.05 (0.03-0.08) | 0.44 (0.26-0.62) |
| Southern Sub-Saharan Africa | 5 (3-8) | 9 (6-14) | 0.8 (1-0.75) | 0.12 (0.08-0.18) | 0.09 (0.06-0.14) | -1.04 (-1.35--0.73) |
| Tropical Latin America | 3 (2-4) | 18 (11-28) | 5 (4.5-6) | 0.02 (0.01-0.03) | 0.04 (0.02-0.06) | 2.55 (2.1-3.01) |
| Western Europe | 141 (83-210) | 161 (103-234) | 0.14 (0.24-0.11) | 0.14 (0.09-0.22) | 0.11 (0.07-0.16) | -1.08 (-1.31--0.84) |
| Western Sub-Saharan Africa | 6 (3-9) | 11 (6-16) | 0.83 (1-0.78) | 0.04 (0.02-0.06) | 0.03 (0.02-0.05) | -0.53 (-0.67--0.38) |

**Table S8. Prevalent cases, prevalence rates, and EAPC trends of HF impairment under CRD cause categories across 204 countries and territories from 1990 to 2021 among adults aged ≥55 years.**

| **Location** | **Prevalent Cases** | |  | **Prevalent Rates** | |  |
| --- | --- | --- | --- | --- | --- | --- |
|  | **1990 (95% UI)** | **2021 (95% UI)** | **Relative change, 1990–2021(95% UI)** | **1990, per 100,000 (95% UI)** | **2021, per 100,000 (95% UI)** | **EAPC (95% CI)** |
| Afghanistan | 506.88 (345.86-698.03) | 345.99 (226.29-483.34) | -0.32 (-0.35--0.31) | 63.96 (43.64-88.09) | 35.75 (23.38-49.94) | -2.02 (-2.14--1.89) |
| Albania | 9744.4 (7048.76-12775.79) | 46451.33 (34863.38-60565.93) | 3.77 (3.95-3.74) | 178.5 (129.12-234.03) | 378.54 (284.11-493.56) | 2.33 (1.95-2.71) |
| Algeria | 15156.4 (10674.98-20350.66) | 41048.72 (34287.16-49336.43) | 1.71 (2.21-1.42) | 158.53 (111.66-212.86) | 265.26 (221.56-318.81) | 1.62 (1.46-1.78) |
| American Samoa | 4053.49 (2819.36-5568.73) | 10301.02 (7099.39-15010.15) | 1.54 (1.52-1.7) | 84.91 (59.06-116.65) | 73.87 (50.91-107.64) | -0.68 (-0.78--0.59) |
| Andorra | 1244.05 (857.36-1642.43) | 5390.83 (3900.4-7296.34) | 3.33 (3.55-3.44) | 73.97 (50.98-97.66) | 120.39 (87.11-162.95) | 1.76 (1.63-1.88) |
| Angola | 2.38 (1.78-3.05) | 4.86 (3.48-6.52) | 1.04 (0.96-1.14) | 27.58 (20.58-35.26) | 25.77 (18.45-34.57) | -0.45 (-0.65--0.25) |
| Antigua and Barbuda | 658.55 (493.82-879.75) | 1966.09 (1477.83-2634.21) | 1.99 (1.99-1.99) | 88.88 (66.65-118.74) | 90.44 (67.98-121.18) | 0.11 (-0.06-0.27) |
| Argentina | 2735.58 (2024.97-3604.37) | 9004.41 (6676.72-11824.19) | 2.29 (2.3-2.28) | 153.7 (113.78-202.52) | 186.96 (138.63-245.5) | 0.53 (0.44-0.63) |
| Armenia | 6.06 (4.53-8.12) | 18.42 (13.5-24.42) | 2.04 (1.98-2.01) | 24.68 (18.44-33.09) | 25.6 (18.76-33.94) | 0.42 (0.34-0.49) |
| Australia | 17519.55 (12468.43-23883.41) | 40070.59 (27118.51-60189.06) | 1.29 (1.17-1.52) | 108.44 (77.18-147.83) | 95.68 (64.75-143.72) | -0.49 (-0.63--0.34) |
| Austria | 1292.85 (971.15-1732.17) | 4852.37 (3673.06-6288.35) | 2.75 (2.78-2.63) | 76.29 (57.3-102.21) | 142 (107.49-184.03) | 2.22 (1.95-2.49) |
| Azerbaijan | 21.81 (15.98-29.2) | 51.02 (38.25-67.42) | 1.34 (1.39-1.31) | 46.53 (34.08-62.28) | 55.99 (41.98-73.99) | 0.36 (0.1-0.62) |
| Bahrain | 8.41 (3.68-13.2) | 20.25 (8.78-32.52) | 1.41 (1.39-1.46) | 53.73 (23.49-84.33) | 33.83 (14.66-54.31) | -1.81 (-1.99--1.64) |
| Bangladesh | 15965.1 (10456.88-22991.09) | 38619.57 (26394.14-54763.3) | 1.42 (1.52-1.38) | 107.49 (70.4-154.79) | 183.64 (125.51-260.4) | 1.84 (1.71-1.97) |
| Barbados | 8204.61 (6037.11-10978.54) | 18393.24 (13761.91-24662.83) | 1.24 (1.28-1.25) | 308.28 (226.84-412.51) | 326.37 (244.19-437.61) | 0.61 (0.42-0.81) |
| Belarus | 4.68 (3.48-6.25) | 6.73 (4.86-9.07) | 0.44 (0.4-0.45) | 47.7 (35.44-63.62) | 44.64 (32.22-60.12) | -0.11 (-0.27-0.05) |
| Belgium | 10960.84 (7366.16-15920.25) | 6071.6 (3622.49-9614.56) | -0.45 (-0.51--0.4) | 87.92 (59.09-127.7) | 44.72 (26.68-70.81) | -3.83 (-4.25--3.4) |
| Belize | 340.28 (254.39-453.95) | 743.54 (548.12-1007.27) | 1.19 (1.15-1.22) | 99.23 (74.18-132.37) | 96.17 (70.89-130.28) | -0.18 (-0.35-0) |
| Benin | 12.69 (9.62-16.81) | 46.3 (34.32-61.74) | 2.65 (2.57-2.67) | 82.42 (62.52-109.21) | 92.43 (68.51-123.26) | 0.63 (0.37-0.89) |
| Bermuda | 1925.89 (1414.76-2531.16) | 5553.05 (4154.71-7281.24) | 1.88 (1.94-1.88) | 130.49 (95.86-171.5) | 113.26 (84.74-148.51) | -0.93 (-1.14--0.72) |
| Bhutan | 12661.97 (7699.06-18836.96) | 54425.32 (35496.39-79703.29) | 3.3 (3.61-3.23) | 42.76 (26-63.61) | 104.26 (68-152.69) | 2.76 (2.62-2.89) |
| Bolivia | 1033.95 (623.88-1492.58) | 3349.63 (2370.86-4569.1) | 2.24 (2.8-2.06) | 61.21 (36.93-88.36) | 114.29 (80.89-155.9) | 3.16 (2.59-3.75) |
| Bosnia and Herzegovina | 23.93 (17.84-31.63) | 93.22 (69.95-120.96) | 2.9 (2.92-2.82) | 156.11 (116.38-206.35) | 171.38 (128.59-222.37) | 0.71 (0.38-1.05) |
| Botswana | 333.81 (250.74-437.86) | 996.06 (747.62-1328.69) | 1.98 (1.98-2.03) | 54.6 (41.01-71.62) | 59.61 (44.74-79.51) | 0.17 (0.01-0.33) |
| Brazil | 203.82 (132.23-306.12) | 254.7 (104.01-417.3) | 0.25 (-0.21-0.36) | 18.35 (11.9-27.56) | 24.26 (9.91-39.75) | 1 (0.79-1.22) |
| Brunei | 7183.44 (5425.8-9546.01) | 17879.08 (13384.48-23402.69) | 1.49 (1.47-1.45) | 183.51 (138.61-243.86) | 211.78 (158.54-277.21) | 0.47 (0.27-0.66) |
| Bulgaria | 6.06 (4.5-8.11) | 8.04 (5.86-10.6) | 0.33 (0.3-0.31) | 50.23 (37.28-67.22) | 40.04 (29.15-52.76) | -1.02 (-1.27--0.76) |
| Burkina Faso | 17560.23 (12942.86-23323.66) | 42660.64 (31854.06-55327.6) | 1.43 (1.46-1.37) | 295.91 (218.1-393.03) | 219.47 (163.88-284.64) | -1.1 (-1.23--0.97) |
| Burundi | 321.2 (232.8-428.51) | 765.49 (566.12-1000.59) | 1.38 (1.43-1.34) | 59.55 (43.16-79.45) | 64.59 (47.77-84.43) | 0.3 (0.08-0.52) |
| Cabo Verde | 12.5 (9.01-17.37) | 27.27 (19.71-37.06) | 1.18 (1.19-1.13) | 20.39 (14.7-28.33) | 24.21 (17.5-32.91) | 0.58 (0.34-0.83) |
| Cambodia | 2761.92 (1673.9-3885.11) | 18312.57 (13752.94-23910.8) | 5.63 (7.22-5.15) | 55.5 (33.63-78.07) | 109.5 (82.24-142.98) | 2.69 (2.3-3.09) |
| Cameroon | 11.59 (8.3-15.59) | 40.83 (30.01-54.05) | 2.52 (2.62-2.47) | 79.25 (56.75-106.54) | 97.02 (71.32-128.43) | 0.75 (0.37-1.13) |
| Canada | 3.54 (2.54-4.88) | 4.62 (3.36-6.22) | 0.31 (0.32-0.27) | 138.97 (99.47-191.52) | 78.91 (57.38-106.21) | -2.27 (-2.55--2) |
| Central African Republic | 44.24 (34.15-58.04) | 166.35 (123.79-222.97) | 2.76 (2.62-2.84) | 101.8 (78.57-133.55) | 117.12 (87.16-156.99) | 0.23 (0.08-0.38) |
| Chad | 195.13 (145.21-263.23) | 433.96 (322.83-576.36) | 1.22 (1.22-1.19) | 66.25 (49.3-89.37) | 82.05 (61.04-108.97) | 0.37 (-0.33-1.07) |
| Chile | 3.1 (2.15-4.47) | 8.53 (6.17-11.6) | 1.75 (1.87-1.6) | 25.77 (17.89-37.22) | 33.54 (24.26-45.63) | 0.95 (0.57-1.33) |
| China | 3257.95 (2263.61-4579.51) | 10013.7 (6688.97-15194.26) | 2.07 (1.96-2.32) | 137.23 (95.35-192.9) | 297.45 (198.69-451.34) | 2.73 (2.56-2.91) |
| Colombia | 26.93 (20.54-34.6) | 76.61 (57.9-100.15) | 1.84 (1.82-1.89) | 61.88 (47.21-79.51) | 68.59 (51.84-89.66) | 0.47 (0.23-0.71) |
| Comoros | 4.68 (3.41-6.33) | 12.97 (9.4-17.51) | 1.77 (1.76-1.77) | 79.64 (58.09-107.85) | 109.64 (79.48-148.04) | 1.34 (1.12-1.56) |
| Congo (Brazzaville) | 825.79 (474.72-1322.63) | 1557.05 (918.85-2477.79) | 0.89 (0.94-0.87) | 179.39 (103.13-287.32) | 144.15 (85.07-229.39) | -0.66 (-0.78--0.54) |
| Cook Islands | 919.79 (684.17-1202.2) | 2375.61 (1780.49-3112.76) | 1.58 (1.6-1.59) | 310.87 (231.24-406.32) | 289.25 (216.79-379) | -0.46 (-0.61--0.32) |
| Costa Rica | 52.99 (38.06-71.63) | 193.23 (139.21-258.62) | 2.65 (2.66-2.61) | 38.6 (27.72-52.17) | 55.47 (39.97-74.24) | 1.26 (1.22-1.31) |
| Côte d'Ivoire | 3455.39 (2593.82-4560.13) | 20900.47 (15594.57-27035.66) | 5.05 (5.01-4.93) | 120.02 (90.09-158.39) | 218.54 (163.06-282.7) | 2.08 (1.86-2.3) |
| Croatia | 513.42 (382.38-684.37) | 1673.42 (1263.38-2204.93) | 2.26 (2.3-2.22) | 97.92 (72.93-130.52) | 108.28 (81.75-142.67) | 0.64 (0.5-0.78) |
| Cuba | 1999.97 (1552.64-2537.18) | 9367.64 (7299.15-11950.53) | 3.68 (3.7-3.71) | 101.43 (78.75-128.68) | 167.48 (130.49-213.65) | 2.35 (2.05-2.66) |
| Cyprus | 791.74 (595.87-1029.4) | 2771.16 (2072.99-3710.9) | 2.5 (2.48-2.6) | 92.08 (69.3-119.72) | 100.13 (74.9-134.09) | 0.36 (0.3-0.42) |
| Czechia | 23.06 (16.95-31.22) | 52 (38.4-70.15) | 1.25 (1.27-1.25) | 101.97 (74.96-138.1) | 95.85 (70.8-129.32) | 0.02 (-0.1-0.15) |
| Denmark | 186.38 (104.48-270.66) | 647.88 (459.52-865.59) | 2.48 (3.4-2.2) | 51.4 (28.82-74.65) | 42.66 (30.25-56.99) | -0.86 (-0.98--0.75) |
| Djibouti | 341.6 (259.18-436.12) | 1628.86 (1217.83-2145.3) | 3.77 (3.7-3.92) | 120.11 (91.13-153.35) | 170.12 (127.19-224.06) | 1.01 (0.84-1.18) |
| Dominica | 12.12 (9.03-16.48) | 10.21 (7.59-13.54) | -0.16 (-0.16--0.18) | 152.27 (113.44-207.06) | 77.57 (57.64-102.89) | -2.7 (-2.86--2.54) |
| Dominican Republic | 108.2 (65.84-156.02) | 151.19 (86.45-224.64) | 0.4 (0.31-0.44) | 62.44 (38-90.04) | 38.29 (21.9-56.9) | -1.73 (-1.82--1.63) |
| DR Congo | 45.73 (33.65-61.48) | 77.08 (56.51-104.42) | 0.69 (0.68-0.7) | 81.13 (59.7-109.08) | 56.37 (41.33-76.36) | -1.39 (-1.55--1.23) |
| Ecuador | 685.21 (523.47-882.21) | 1431.58 (1085.07-1855.64) | 1.09 (1.07-1.1) | 140.14 (107.06-180.43) | 140 (106.11-181.47) | 0.3 (0.18-0.41) |
| Egypt | 659267.32 (487061.02-862270.01) | 1463648.37 (1047688.94-2001167.71) | 1.22 (1.15-1.32) | 459.36 (339.37-600.81) | 386.23 (276.47-528.08) | -1.29 (-1.55--1.04) |
| El Salvador | 510.09 (380.93-679.62) | 2025.73 (1475.62-2694.77) | 2.97 (2.87-2.97) | 88.7 (66.24-118.18) | 110.65 (80.6-147.19) | 0.88 (0.72-1.04) |
| Equatorial Guinea | 11659.42 (8573.78-15135.91) | 35356.24 (24383.82-49651.17) | 2.03 (1.84-2.28) | 167.7 (123.32-217.7) | 164.06 (113.15-230.4) | -0.24 (-0.36--0.13) |
| Eritrea | 13837.77 (8684.81-21238.38) | 19037.43 (11823.89-28842.98) | 0.38 (0.36-0.36) | 189.95 (119.21-291.53) | 126.9 (78.82-192.27) | -1.44 (-1.51--1.37) |
| Estonia | 218.94 (167.73-286.32) | 875.67 (661.09-1142.01) | 3 (2.94-2.99) | 88.11 (67.5-115.23) | 107.57 (81.21-140.29) | 0.88 (0.66-1.1) |
| Eswatini | 1151.5 (756.12-1655.49) | 2880.45 (1944.34-4060.18) | 1.5 (1.57-1.45) | 175.01 (114.92-251.6) | 201.94 (136.31-284.64) | 0.58 (0.48-0.67) |
| Ethiopia | 393 (270.64-530.07) | 412.8 (301.71-564.02) | 0.05 (0.11-0.06) | 80.74 (55.6-108.9) | 52.48 (38.36-71.7) | -0.41 (-0.88-0.06) |
| Federated States of Micronesia | 838.66 (560.86-1166.87) | 461.23 (306.37-649.76) | -0.45 (-0.45--0.44) | 37.05 (24.78-51.56) | 19.4 (12.89-27.34) | -2.15 (-2.44--1.85) |
| Fiji | 433.33 (330.15-568.57) | 1234.15 (888.97-1650.34) | 1.85 (1.69-1.9) | 130.01 (99.05-170.58) | 117.35 (84.53-156.93) | -0.29 (-0.36--0.21) |
| Finland | 281.61 (210.11-377.02) | 1163.94 (888.52-1528.86) | 3.13 (3.23-3.06) | 116.23 (86.72-155.61) | 156.54 (119.49-205.61) | 0.68 (0.4-0.95) |
| France | 337.19 (220.24-476.3) | 858.74 (518.71-1210.55) | 1.55 (1.36-1.54) | 30.29 (19.78-42.79) | 57.58 (34.78-81.17) | 1.45 (1.08-1.82) |
| Gabon | 1034.92 (718.31-1402.79) | 448.91 (310.89-611.58) | -0.57 (-0.57--0.56) | 200.99 (139.5-272.43) | 53.08 (36.76-72.31) | -4.65 (-5.2--4.1) |
| Georgia | 312.9 (214.69-422.58) | 443.91 (308.73-611.66) | 0.42 (0.44-0.45) | 90.17 (61.87-121.77) | 56.46 (39.26-77.79) | -1.68 (-1.8--1.56) |
| Germany | 347.14 (241.34-469.29) | 423.31 (271.34-591.76) | 0.22 (0.12-0.26) | 39.8 (27.67-53.8) | 22.1 (14.16-30.89) | -2.09 (-2.33--1.85) |
| Ghana | 778.73 (592.32-1030.43) | 4060.08 (3056.72-5300.3) | 4.21 (4.16-4.14) | 49.73 (37.83-65.81) | 77.6 (58.42-101.3) | 1.46 (1.12-1.79) |
| Greece | 299.12 (219.21-396.16) | 651.86 (462.92-887.07) | 1.18 (1.11-1.24) | 28.83 (21.13-38.19) | 39.74 (28.22-54.08) | 1.53 (1.31-1.74) |
| Greenland | 3425.54 (2028.76-5276.1) | 2450.56 (1481.33-3464.29) | -0.28 (-0.27--0.34) | 68.46 (40.54-105.44) | 40.81 (24.67-57.69) | -1.34 (-1.56--1.11) |
| Grenada | 462.67 (311.91-634.47) | 593.05 (381.58-830.25) | 0.28 (0.22-0.31) | 63.07 (42.52-86.49) | 54.15 (34.84-75.8) | -0.31 (-0.51--0.11) |
| Guam | 707.94 (426.29-1018.26) | 1205.8 (701.88-1710.81) | 0.7 (0.65-0.68) | 34.04 (20.5-48.96) | 42.98 (25.02-60.99) | -0.3 (-0.76-0.17) |
| Guatemala | 18521.07 (13500.98-24892.09) | 71251.91 (50516.45-100107.66) | 2.85 (2.74-3.02) | 125.32 (91.36-168.43) | 164.52 (116.65-231.15) | 0.79 (0.69-0.89) |
| Guinea | 704.2 (519.2-916.66) | 2658.26 (1997.82-3492.43) | 2.77 (2.85-2.81) | 33.61 (24.78-43.75) | 43.77 (32.89-57.5) | 1.07 (0.94-1.2) |
| Guinea-Bissau | 268.37 (203.38-346.96) | 836.7 (626.29-1088.78) | 2.12 (2.08-2.14) | 73.98 (56.06-95.64) | 84.51 (63.25-109.97) | 0.51 (0.34-0.67) |
| Guyana | 19.23 (14.57-25.09) | 110.78 (83.84-143.88) | 4.76 (4.75-4.73) | 68.52 (51.94-89.43) | 68.22 (51.64-88.61) | -0.38 (-0.63--0.13) |
| Haiti | 1992.85 (1507.83-2571.67) | 2825.5 (2111.19-3752.04) | 0.42 (0.4-0.46) | 44.57 (33.72-57.51) | 25.53 (19.08-33.91) | -2.1 (-2.25--1.95) |
| Honduras | 99.62 (75.87-131.31) | 467.09 (349.05-614.94) | 3.69 (3.6-3.68) | 46.33 (35.29-61.07) | 37.15 (27.76-48.91) | -0.45 (-0.63--0.27) |
| Hungary | 244.05 (136.23-352.06) | 633.4 (393.48-887.03) | 1.6 (1.89-1.52) | 56.66 (31.63-81.74) | 86.51 (53.74-121.15) | 1.29 (1.12-1.46) |
| Iceland | 1879.79 (1363.9-2620.04) | 8198.66 (5726.72-11709.97) | 3.36 (3.2-3.47) | 40.78 (29.59-56.83) | 63.11 (44.08-90.14) | 1.71 (1.52-1.9) |
| India | 15.44 (11.65-20.48) | 79.34 (60.83-104.5) | 4.14 (4.22-4.1) | 16.88 (12.74-22.4) | 17.02 (13.05-22.41) | 0.56 (0.1-1.02) |
| Indonesia | 279.35 (210.74-364.85) | 1480.49 (1116.69-1912.22) | 4.3 (4.3-4.24) | 73.68 (55.58-96.23) | 151.07 (113.94-195.12) | 3.15 (2.82-3.48) |
| Iran | 6771.69 (4881.8-9444.89) | 12683.97 (8665.51-18436.43) | 0.87 (0.78-0.95) | 87.88 (63.35-122.57) | 104.69 (71.53-152.17) | 0.63 (0.36-0.89) |
| Iraq | 364.97 (272.84-471.23) | 866.67 (659.31-1144.97) | 1.37 (1.42-1.43) | 28.91 (21.62-37.33) | 22.31 (16.97-29.47) | -0.76 (-0.86--0.66) |
| Ireland | 18.47 (13.24-25.01) | 40.72 (23.81-64.8) | 1.2 (0.8-1.59) | 16.95 (12.15-22.95) | 23.35 (13.65-37.16) | 0 (-0.26-0.27) |
| Israel | 705.22 (418.53-1136.75) | 935.48 (533.87-1498.54) | 0.33 (0.28-0.32) | 212.76 (126.27-342.95) | 154.86 (88.38-248.07) | -1.05 (-1.13--0.97) |
| Italy | 194.72 (152.91-251.61) | 399.02 (300.49-512.61) | 1.05 (0.97-1.04) | 62.6 (49.16-80.9) | 47.71 (35.93-61.29) | -0.51 (-0.68--0.33) |
| Jamaica | 74.92 (51.15-103.56) | 97.48 (57.56-152.1) | 0.3 (0.13-0.47) | 20.9 (14.27-28.89) | 22.28 (13.16-34.77) | -0.58 (-0.82--0.34) |
| Japan | 18580.98 (12374.09-26708.23) | 18311.86 (11920.52-26984.53) | -0.01 (-0.04-0.01) | 58.87 (39.2-84.62) | 43.08 (28.04-63.49) | -1.44 (-1.75--1.12) |
| Jordan | 1432.8 (890.05-2016.63) | 2790.48 (1766.15-3951.09) | 0.95 (0.98-0.96) | 55.63 (34.56-78.29) | 87.75 (55.54-124.24) | 1.63 (1.43-1.83) |
| Kazakhstan | 871.55 (645.51-1139.45) | 2336.5 (1753.43-3108.84) | 1.68 (1.72-1.73) | 37.17 (27.53-48.59) | 38.89 (29.18-51.75) | 0.34 (0.24-0.44) |
| Kenya | 34.36 (25.41-45.58) | 75.67 (55.93-99.44) | 1.2 (1.2-1.18) | 34.19 (25.28-45.35) | 24.22 (17.91-31.83) | -0.7 (-0.85--0.56) |
| Kiribati | 519.39 (393.9-676.89) | 1068.07 (797.19-1387.99) | 1.06 (1.02-1.05) | 58.3 (44.21-75.98) | 35.48 (26.48-46.1) | -1.4 (-1.51--1.28) |
| Kuwait | 346.14 (198.01-544.98) | 799.41 (467.71-1305.5) | 1.31 (1.36-1.4) | 177.44 (101.5-279.37) | 127.97 (74.87-208.98) | -0.93 (-1.01--0.86) |
| Kyrgyzstan | 488.24 (290.95-711.26) | 2074.73 (1373.41-2899.34) | 3.25 (3.72-3.08) | 41.12 (24.5-59.9) | 103.08 (68.24-144.05) | 2.98 (2.51-3.45) |
| Laos | 20.7 (12.22-29.61) | 89.24 (61.22-123.23) | 3.31 (4.01-3.16) | 43.38 (25.6-62.06) | 91.63 (62.86-126.53) | 1.93 (1.61-2.25) |
| Latvia | 352.12 (264.12-456.07) | 963.74 (712.67-1296.98) | 1.74 (1.7-1.84) | 39.94 (29.96-51.73) | 40.79 (30.16-54.89) | 0.27 (0.17-0.37) |
| Lebanon | 908.9 (613.03-1230.31) | 3133.72 (2251.72-4174.11) | 2.45 (2.67-2.39) | 133.52 (90.06-180.74) | 238.78 (171.58-318.06) | 2.04 (1.95-2.14) |
| Lesotho | 16.03 (11.31-21.78) | 50.65 (37.02-66.28) | 2.16 (2.27-2.04) | 162.43 (114.58-220.76) | 191.41 (139.89-250.47) | 0.44 (0.24-0.65) |
| Liberia | 340.91 (253.95-452.09) | 1123.51 (851.62-1450.65) | 2.3 (2.35-2.21) | 39.15 (29.17-51.92) | 48.07 (36.44-62.07) | 0.66 (0.45-0.86) |
| Libya | 5.64 (4.12-7.5) | 10.26 (7.3-14.33) | 0.82 (0.77-0.91) | 154.24 (112.56-205.05) | 121.5 (86.47-169.71) | -0.95 (-1.06--0.84) |
| Lithuania | 5.1 (3.84-6.64) | 53.12 (40.68-68.79) | 9.42 (9.59-9.36) | 31.25 (23.48-40.67) | 34.68 (26.56-44.91) | 0.57 (0.4-0.74) |
| Luxembourg | 1118.11 (750.5-1595.52) | 4697.7 (3190.29-6692.24) | 3.2 (3.25-3.19) | 47.22 (31.7-67.38) | 134.47 (91.32-191.56) | 4.53 (4.2-4.86) |
| Madagascar | 7870.54 (6075.03-10118.33) | 27906.39 (21119.67-36246.27) | 2.55 (2.48-2.58) | 131.73 (101.68-169.36) | 168.91 (127.83-219.39) | 1.4 (1.19-1.62) |
| Malawi | 344.86 (259.73-449.04) | 969.1 (723.51-1268.34) | 1.81 (1.79-1.82) | 43 (32.38-55.99) | 43.09 (32.17-56.4) | 0.28 (0.19-0.37) |
| Malaysia | 443.99 (297.73-610.86) | 2853.01 (2027.68-3886.08) | 5.43 (5.81-5.36) | 54.78 (36.73-75.36) | 142.75 (101.46-194.45) | 3.06 (2.73-3.4) |
| Maldives | 35.3 (26.89-45.56) | 318.11 (228.77-422.4) | 8.01 (7.51-8.27) | 62.89 (47.91-81.18) | 44.3 (31.86-58.82) | -1.53 (-1.69--1.38) |
| Mali | 614.95 (450.57-801.47) | 703.93 (512.13-938.47) | 0.14 (0.14-0.17) | 50.61 (37.08-65.95) | 57.29 (41.68-76.38) | 0.82 (0.68-0.96) |
| Malta | 19895.79 (15069.43-26370.01) | 47445.91 (35772.94-62699.47) | 1.38 (1.37-1.38) | 262.66 (198.95-348.13) | 203.05 (153.1-268.33) | -0.79 (-0.86--0.72) |
| Marshall Islands | 270.13 (155.7-437.38) | 479.71 (277.42-766.2) | 0.78 (0.78-0.75) | 248.94 (143.49-403.08) | 212.31 (122.78-339.11) | -0.48 (-0.51--0.45) |
| Mauritania | 557.28 (377.97-759.76) | 274.9 (192.79-380.06) | -0.51 (-0.49--0.5) | 72.19 (48.96-98.41) | 26.04 (18.26-36) | -3.39 (-3.79--2.99) |
| Mauritius | 88.36 (68.19-114.46) | 281.56 (215.28-361.56) | 2.19 (2.16-2.16) | 222.66 (171.82-288.41) | 282.72 (216.17-363.05) | 0.81 (0.76-0.86) |
| Mexico | 1.28 (0.93-1.7) | 1.66 (1.21-2.21) | 0.3 (0.3-0.3) | 110.43 (80.4-146.42) | 90.94 (66.26-121.13) | -1.07 (-1.25--0.88) |
| Moldova | 193678.21 (143558.57-255779.12) | 602593.75 (428286.31-840185.47) | 2.11 (1.98-2.28) | 252.62 (187.24-333.61) | 299.73 (213.03-417.91) | 0.61 (0.49-0.72) |
| Monaco | 586.02 (394.2-814.73) | 1409.64 (894.44-1978.95) | 1.41 (1.27-1.43) | 86.85 (58.42-120.74) | 155.88 (98.91-218.84) | 2.14 (1.92-2.37) |
| Mongolia | 43.77 (30.6-60.74) | 149.04 (107.08-202.8) | 2.41 (2.5-2.34) | 60.42 (42.24-83.84) | 94.45 (67.86-128.52) | 2.13 (1.85-2.41) |
| Montenegro | 11828.8 (7021.74-17470.01) | 34787.76 (23336.42-50097.43) | 1.94 (2.32-1.87) | 77.72 (46.14-114.79) | 153.14 (102.73-220.53) | 2.64 (2.05-3.22) |
| Morocco | 6143.64 (4529.19-8222.6) | 26892.79 (21477.79-33196.91) | 3.38 (3.74-3.04) | 187.22 (138.02-250.57) | 363.03 (289.93-448.13) | 1.85 (1.64-2.06) |
| Mozambique | 5012.49 (3766.22-6601.66) | 13381.4 (9917.25-17600.54) | 1.67 (1.63-1.67) | 325.63 (244.67-428.87) | 338.1 (250.57-444.7) | 0.19 (0.14-0.24) |
| Myanmar | 102.12 (60.32-165.25) | 148.1 (84.55-234.06) | 0.45 (0.4-0.42) | 226.16 (133.59-365.97) | 163.57 (93.38-258.52) | -1.06 (-1.26--0.87) |
| Namibia | 18858.53 (13577.46-25929.92) | 31577.38 (22082.25-45145.1) | 0.67 (0.63-0.74) | 206.79 (148.88-284.34) | 159.27 (111.38-227.71) | -1.03 (-1.1--0.96) |
| Nauru | 1050.26 (606.09-1719.87) | 2023.87 (1140.73-3356.56) | 0.93 (0.88-0.95) | 168.6 (97.3-276.1) | 104.67 (58.99-173.59) | -1.57 (-1.67--1.46) |
| Nepal | 150.94 (101.51-214.23) | 107.25 (71.89-150.11) | -0.29 (-0.29--0.3) | 46.48 (31.26-65.96) | 15.15 (10.16-21.21) | -3.88 (-4.19--3.58) |
| Netherlands | 15448.07 (10701.21-21116.53) | 53444.14 (38368.4-71762.5) | 2.46 (2.59-2.4) | 73.62 (51-100.64) | 169.59 (121.75-227.72) | 4.33 (3.81-4.85) |
| New Zealand | 4567.75 (3404.49-5949) | 18730.14 (13376.93-25142.49) | 3.1 (2.93-3.23) | 137.96 (102.82-179.68) | 320.13 (228.63-429.72) | 3.07 (2.83-3.3) |
| Nicaragua | 323.13 (188.23-530.95) | 497.15 (290.99-804.14) | 0.54 (0.55-0.51) | 171.14 (99.69-281.2) | 138.99 (81.35-224.81) | -0.61 (-0.66--0.56) |
| Niger | 12045.64 (7724.37-17402.21) | 22449.39 (14302.95-33375.4) | 0.86 (0.85-0.92) | 356.86 (228.84-515.55) | 284.58 (181.31-423.09) | -0.74 (-1.04--0.44) |
| Nigeria | 922.73 (647.95-1303.85) | 552.74 (341.92-796.8) | -0.4 (-0.47--0.39) | 47.18 (33.13-66.67) | 11.89 (7.36-17.14) | -5.42 (-5.8--5.03) |
| Niue | 228.84 (125.34-390.45) | 420.67 (237.91-693.25) | 0.84 (0.9-0.78) | 128.55 (70.41-219.34) | 96.81 (54.75-159.54) | -0.84 (-0.88--0.8) |
| North Korea | 23.96 (18.06-31.67) | 34.01 (25.36-45.22) | 0.42 (0.4-0.43) | 166.22 (125.29-219.76) | 138.13 (102.98-183.67) | -0.67 (-0.79--0.54) |
| North Macedonia | 894.07 (420.89-1487.36) | 4085.05 (2535.9-6044.03) | 3.57 (5.03-3.06) | 82.56 (38.87-137.35) | 251.89 (156.37-372.69) | 4.32 (3.97-4.67) |
| Northern Mariana Islands | 79.92 (46.6-117.17) | 321.66 (227.02-448.94) | 3.02 (3.87-2.83) | 85.58 (49.9-125.47) | 181.01 (127.75-252.64) | 2.43 (1.85-3.01) |
| Norway | 1586.61 (1201.75-1941.01) | 4118.04 (3049.58-5180.14) | 1.6 (1.54-1.67) | 81.54 (61.76-99.75) | 139.35 (103.19-175.29) | 1.28 (1.06-1.49) |
| Oman | 1.34 (0.98-1.79) | 3.02 (2.23-4.09) | 1.25 (1.28-1.28) | 62.98 (45.99-83.94) | 63.97 (47.3-86.71) | 0.25 (0.15-0.34) |
| Pakistan | 2150.33 (1519.02-2947.59) | 707.76 (467.12-1057.64) | -0.67 (-0.69--0.64) | 92.89 (65.62-127.33) | 24.6 (16.24-36.77) | -5.69 (-6.09--5.3) |
| Palau | 131.97 (74.85-216.51) | 167.49 (96.43-268.79) | 0.27 (0.29-0.24) | 134.79 (76.46-221.15) | 93.7 (53.94-150.37) | -0.97 (-1.04--0.9) |
| Palestine | 2666.58 (1989.3-3512.21) | 7651.46 (5558.3-10114.93) | 1.87 (1.79-1.88) | 109.38 (81.6-144.06) | 196.66 (142.86-259.98) | 2.19 (1.9-2.49) |
| Panama | 4313.1 (2499.39-6958.38) | 8542.79 (5066.54-13944.11) | 0.98 (1.03-1) | 163.33 (94.65-263.5) | 141.44 (83.89-230.87) | -0.42 (-0.73--0.12) |
| Papua New Guinea | 140.55 (96.13-189.68) | 226.43 (153.17-314.57) | 0.61 (0.59-0.66) | 42.72 (29.22-57.66) | 38.19 (25.83-53.06) | -0.25 (-0.3--0.19) |
| Paraguay | 64.62 (36.79-103.88) | 83.25 (49.31-135.39) | 0.29 (0.34-0.3) | 202.45 (115.24-325.44) | 105.25 (62.34-171.17) | -1.96 (-2.22--1.71) |
| Peru | 997.64 (588.7-1600.39) | 1560.8 (929-2476.79) | 0.56 (0.58-0.55) | 265.09 (156.42-425.24) | 194.97 (116.05-309.39) | -1.31 (-1.45--1.18) |
| Philippines | 513.2 (317.35-797.78) | 465.55 (271.83-757.47) | -0.09 (-0.14--0.05) | 366.99 (226.94-570.5) | 257.84 (150.55-419.53) | -1.32 (-1.47--1.18) |
| Poland | 65.82 (38.47-105.66) | 129.87 (76.17-206.77) | 0.97 (0.98-0.96) | 202.8 (118.55-325.58) | 160.67 (94.24-255.8) | -0.65 (-0.69--0.62) |
| Portugal | 3423.31 (2390.08-4627.78) | 9286.78 (6061.82-12832.36) | 1.71 (1.54-1.77) | 61.52 (42.95-83.16) | 99.5 (64.95-137.49) | 1.69 (1.42-1.95) |
| Puerto Rico | 83896.45 (55995.53-118180.85) | 256006.57 (175931.47-364710.77) | 2.05 (2.14-2.09) | 159.92 (106.73-225.27) | 255.37 (175.5-363.81) | 1.48 (1.26-1.71) |
| Qatar | 5.02 (3.68-6.8) | 23.48 (16.43-32.19) | 3.68 (3.46-3.73) | 47.42 (34.83-64.29) | 100.8 (70.56-138.22) | 2.44 (2.22-2.67) |
| Romania | 105.61 (60.66-169.07) | 132.01 (74.63-209.63) | 0.25 (0.23-0.24) | 165.44 (95.04-264.86) | 114.92 (64.97-182.49) | -1.07 (-1.15--0.99) |
| Russia | 1205.55 (703.42-1885.07) | 1566.92 (939.75-2487.29) | 0.3 (0.34-0.32) | 217.85 (127.11-340.64) | 171.52 (102.87-272.26) | -0.66 (-0.75--0.57) |
| Rwanda | 34.09 (19.98-55.18) | 120.73 (68.53-198.59) | 2.54 (2.43-2.6) | 158.89 (93.15-257.2) | 116.42 (66.08-191.5) | -1.05 (-1.25--0.85) |
| Saint Kitts and Nevis | 1409.99 (816.74-2275.74) | 2591.04 (1532.47-4190.68) | 0.84 (0.88-0.84) | 210.87 (122.15-340.35) | 179.02 (105.88-289.54) | -0.36 (-0.4--0.31) |
| Saint Lucia | 266.17 (149.87-422.89) | 433.6 (244.31-692.3) | 0.63 (0.63-0.64) | 160.33 (90.28-254.73) | 120.86 (68.1-192.97) | -0.93 (-0.96--0.9) |
| Saint Vincent and the Grenadines | 310.6 (177.77-503.54) | 415.99 (238.54-660.6) | 0.34 (0.34-0.31) | 164.83 (94.34-267.22) | 126.01 (72.26-200.11) | -1.14 (-1.4--0.89) |
| Samoa | 185.38 (125.67-264.37) | 192.57 (119.36-286.56) | 0.04 (-0.05-0.08) | 29.65 (20.1-42.28) | 29.26 (18.14-43.55) | -0.59 (-1.08--0.1) |
| San Marino | 3468.65 (2242.78-5102.1) | 6714.17 (4190.42-10167.91) | 0.94 (0.87-0.99) | 260.26 (168.28-382.82) | 179.49 (112.02-271.81) | -1.73 (-1.94--1.51) |
| São Tomé and Príncipe | 10.66 (7.8-14.2) | 15.67 (11.24-20.9) | 0.47 (0.44-0.47) | 178.99 (131-238.38) | 124.6 (89.4-166.16) | -1.31 (-1.45--1.16) |
| Saudi Arabia | 3537.12 (2393.74-4853.17) | 7050.61 (5573.1-9087.96) | 0.99 (1.33-0.87) | 134.91 (91.3-185.1) | 186.49 (147.41-240.37) | 1.84 (1.64-2.05) |
| Senegal | 293.45 (168.25-472.56) | 606.47 (340.91-960.72) | 1.07 (1.03-1.03) | 166.05 (95.21-267.41) | 137.64 (77.37-218.04) | -0.51 (-0.6--0.43) |
| Serbia | 7885.09 (4762.11-11930.71) | 14054.21 (8820.08-21323.27) | 0.78 (0.85-0.79) | 245.41 (148.21-371.33) | 205.3 (128.84-311.49) | -0.68 (-0.76--0.6) |
| Seychelles | 1717.48 (1006.58-2736.77) | 2517.51 (1464.51-4142.46) | 0.47 (0.45-0.51) | 203.27 (119.13-323.91) | 136.33 (79.31-224.32) | -1.59 (-1.75--1.42) |
| Sierra Leone | 1359.57 (805.79-2166.3) | 2046.52 (1179.42-3296.84) | 0.51 (0.46-0.52) | 216.16 (128.11-344.42) | 175.05 (100.88-281.99) | -0.92 (-1.04--0.8) |
| Singapore | 66.91 (48.82-93.46) | 226.62 (167.11-298.13) | 2.39 (2.42-2.19) | 55 (40.14-76.83) | 66.98 (49.39-88.12) | 0.4 (0.28-0.51) |
| Slovakia | 880.6 (514.01-1415.5) | 2617.66 (1560.74-4231.02) | 1.97 (2.04-1.99) | 200.36 (116.95-322.07) | 188.02 (112.1-303.91) | 0.03 (-0.08-0.14) |
| Slovenia | 2181.47 (1292.85-3452.29) | 2910.17 (1684.63-4722.59) | 0.33 (0.3-0.37) | 223.24 (132.3-353.29) | 162.66 (94.16-263.96) | -1.2 (-1.35--1.05) |
| Solomon Islands | 784.29 (460.3-1253.13) | 1442.37 (828.76-2233.85) | 0.84 (0.8-0.78) | 148.42 (87.11-237.14) | 111.71 (64.19-173.01) | -0.62 (-0.81--0.44) |
| Somalia | 1861.33 (1280.74-2607.79) | 2675.74 (1699.2-3912.54) | 0.44 (0.33-0.5) | 89 (61.24-124.69) | 84.32 (53.54-123.29) | 0.24 (-0.34-0.82) |
| South Africa | 869.99 (518.94-1371.35) | 2397.77 (1396.72-3887.18) | 1.76 (1.69-1.83) | 241.62 (144.12-380.86) | 254.25 (148.1-412.19) | 0.04 (-0.1-0.18) |
| South Korea | 67.13 (50.56-90.3) | 161.36 (119.47-210.03) | 1.4 (1.36-1.33) | 46.25 (34.84-62.22) | 37.5 (27.77-48.82) | -0.5 (-0.57--0.43) |
| South Sudan | 1084.31 (635.24-1718.92) | 2240.19 (1322.29-3571.85) | 1.07 (1.08-1.08) | 231.72 (135.75-367.33) | 211.85 (125.04-337.77) | -0.58 (-0.72--0.43) |
| Spain | 89.19 (54.12-126.35) | 352.54 (249.33-488.11) | 2.95 (3.61-2.86) | 63.85 (38.75-90.45) | 100.87 (71.34-139.66) | 1.01 (0.75-1.27) |
| Sri Lanka | 13.99 (10.38-18.64) | 30.8 (22.94-41.44) | 1.2 (1.21-1.22) | 140.39 (104.12-186.98) | 107.41 (80.01-144.51) | -1.01 (-1.08--0.95) |
| Sudan | 4286.38 (2528.56-6815.93) | 7192.51 (4230.61-11322.05) | 0.68 (0.67-0.66) | 237.58 (140.15-377.79) | 174.48 (102.63-274.66) | -0.81 (-0.98--0.64) |
| Suriname | 5.52 (4.1-7.18) | 10.08 (7.43-13.64) | 0.83 (0.81-0.9) | 57.56 (42.73-74.86) | 48.61 (35.84-65.78) | -0.5 (-0.74--0.26) |
| Sweden | 1425.27 (954.57-2000.21) | 6544.49 (4608.81-8967.53) | 3.59 (3.83-3.48) | 109.25 (73.17-153.32) | 339.93 (239.39-465.79) | 4.22 (3.89-4.56) |
| Switzerland | 8.89 (4.78-13.03) | 23.45 (13.86-34.48) | 1.64 (1.9-1.65) | 161.41 (86.7-236.45) | 172.72 (102.07-254.01) | 0.63 (0.48-0.77) |
| Syria | 8.67 (6.38-11.61) | 34.47 (25.26-46.03) | 2.98 (2.96-2.96) | 67.23 (49.46-90.04) | 91.46 (67.03-122.14) | 0.93 (0.69-1.17) |
| Taiwan (province of China) | 8.87 (6.52-11.63) | 19.61 (14.23-26.04) | 1.21 (1.18-1.24) | 80.08 (58.84-104.94) | 125.36 (90.92-166.46) | 1.47 (1.36-1.58) |
| Tajikistan | 2599.11 (1540.48-4091.56) | 4040.4 (2290.3-6318.92) | 0.55 (0.49-0.54) | 248.5 (147.29-391.19) | 170.37 (96.58-266.45) | -1.25 (-1.29--1.21) |
| Tanzania | 4.58 (3.43-6.14) | 15.68 (11.37-20.85) | 2.42 (2.31-2.4) | 32.82 (24.55-43.94) | 48.71 (35.33-64.77) | 1.19 (1.02-1.36) |
| Thailand | 30016.07 (22982.38-39438.88) | 69694.15 (55440.63-88551.42) | 1.32 (1.41-1.25) | 215.69 (165.14-283.4) | 315.21 (250.75-400.5) | 1 (0.88-1.12) |
| The Bahamas | 11307.7 (8570.54-15010.92) | 24167.96 (18230.88-32306.54) | 1.14 (1.13-1.15) | 162.19 (122.93-215.3) | 138.35 (104.36-184.94) | -0.66 (-1.01--0.3) |
| The Gambia | 219.19 (127.92-358.91) | 468.34 (281.96-735.44) | 1.14 (1.2-1.05) | 235.77 (137.59-386.05) | 193.19 (116.31-303.37) | -0.68 (-0.75--0.62) |
| Timor-Leste | 19.95 (14.88-26.05) | 27.64 (20.61-36.96) | 0.39 (0.39-0.42) | 210.28 (156.78-274.56) | 205.83 (153.43-275.19) | 0.03 (-0.05-0.11) |
| Togo | 0.66 (0.47-0.9) | 0.66 (0.48-0.89) | 0 (0.02--0.01) | 91.4 (65.37-124.69) | 71.72 (51.61-96.46) | -1.02 (-1.12--0.93) |
| Tokelau | 0.67 (0.5-0.89) | 0.41 (0.3-0.54) | -0.39 (-0.4--0.39) | 183.43 (137.11-243.16) | 104.16 (77.52-138.96) | -1.58 (-1.69--1.47) |
| Tonga | 1139.11 (814.22-1529.39) | 2337.02 (1575.16-3132.41) | 1.05 (0.93-1.05) | 42.6 (30.45-57.2) | 63.58 (42.86-85.23) | 1.05 (0.8-1.29) |
| Trinidad and Tobago | 1485.38 (895.32-2335.27) | 1973.7 (1134.97-3080.18) | 0.33 (0.27-0.32) | 224.1 (135.08-352.32) | 178.25 (102.5-278.18) | -0.71 (-0.76--0.65) |
| Tunisia | 713.43 (443.76-1111.84) | 1205.35 (688.09-1929.99) | 0.69 (0.55-0.74) | 223 (138.71-347.53) | 145.74 (83.2-233.35) | -1.22 (-1.27--1.16) |
| Türkiye | 1451.85 (843.69-2318.26) | 2534.44 (1493.02-4081.89) | 0.75 (0.77-0.76) | 200.74 (116.65-320.54) | 168.58 (99.31-271.51) | -0.62 (-0.66--0.58) |
| Turkmenistan | 4.18 (3.06-5.46) | 11.79 (8.7-15.77) | 1.82 (1.84-1.89) | 171.25 (125.37-223.64) | 121.25 (89.46-162.27) | -2.03 (-2.36--1.71) |
| Tuvalu | 2.75 (2.01-3.65) | 5.26 (3.91-7.07) | 0.91 (0.95-0.94) | 171.8 (125.9-228.22) | 125.53 (93.31-168.74) | -1.39 (-1.54--1.25) |
| Uganda | 1304.76 (745.41-2068.57) | 2569.91 (1481.71-4138.05) | 0.97 (0.99-1) | 176.21 (100.67-279.37) | 126.26 (72.79-203.3) | -1.11 (-1.17--1.05) |
| UK | 0.32 (0.24-0.43) | 0.34 (0.25-0.45) | 0.06 (0.04-0.05) | 136.54 (102.14-183.66) | 135.21 (100.33-179.96) | -0.1 (-0.26-0.07) |
| Ukraine | 2.77 (1.99-3.81) | 5.58 (4.11-7.42) | 1.01 (1.07-0.95) | 43.36 (31.05-59.64) | 43.31 (31.9-57.58) | -0.52 (-1.01--0.03) |
| United Arab Emirates | 747.61 (543.64-1003.43) | 2372.85 (1699.51-3243.99) | 2.17 (2.13-2.23) | 123.21 (89.6-165.37) | 203.27 (145.59-277.89) | 1.74 (1.53-1.96) |
| Uruguay | 339.26 (228.98-465.19) | 426.06 (291.89-599.61) | 0.26 (0.27-0.29) | 72.44 (48.89-99.33) | 41.48 (28.42-58.38) | -1.62 (-1.98--1.26) |
| United States | 925.56 (532.76-1499.3) | 986.99 (589.89-1539.83) | 0.07 (0.11-0.03) | 223.19 (128.47-361.54) | 158.5 (94.73-247.27) | -1.24 (-1.38--1.09) |
| Uzbekistan | 80.04 (46.66-124.82) | 89.58 (52.28-139.76) | 0.12 (0.12-0.12) | 202.8 (118.22-316.24) | 116.87 (68.21-182.34) | -1.68 (-1.81--1.56) |
| Vanuatu | 1169.36 (700.79-1808.2) | 1586.84 (942.12-2538.35) | 0.36 (0.34-0.4) | 252.34 (151.23-390.2) | 170.9 (101.46-273.38) | -1.24 (-1.28--1.2) |
| Venezuela | 3398.75 (2399.37-4820.05) | 15116.87 (10946.23-20303.15) | 3.45 (3.56-3.21) | 122.54 (86.51-173.78) | 200.76 (145.37-269.63) | 0.9 (0.44-1.37) |
| Viet Nam | 17.2 (9.92-27.25) | 17.83 (10.25-27.98) | 0.04 (0.03-0.03) | 153.64 (88.62-243.34) | 97.5 (56.06-153.02) | -1.48 (-1.63--1.34) |
| Virgin Islands | 745.08 (549.4-972.79) | 1293.62 (983.68-1682.28) | 0.74 (0.79-0.73) | 50.1 (36.95-65.42) | 41.54 (31.59-54.03) | -0.41 (-0.62--0.2) |
| Yemen | 895.31 (512.32-1450.45) | 2126.86 (1232.01-3475.93) | 1.38 (1.4-1.4) | 139.69 (79.93-226.3) | 118.61 (68.71-193.84) | -0.34 (-0.41--0.28) |
| Zambia | 112.79 (65.95-183.17) | 225.97 (132.34-364.99) | 1 (1.01-0.99) | 203.68 (119.09-330.76) | 146.12 (85.58-236.03) | -0.83 (-0.9--0.75) |
| Zimbabwe | 1376.29 (820.77-2212.1) | 2719.17 (1574.21-4293.8) | 0.98 (0.92-0.94) | 134.77 (80.37-216.62) | 99.71 (57.72-157.45) | -0.96 (-1.03--0.9) |

**Table S9. YLDs, YLD rates, and EAPC trends of HF impairment under CRD cause categories across 204 countries and territories from 1990 to 2021 among adults aged ≥55 years.**

| **Location** | **YLDs** | |  | **YLD rates** | |  |
| --- | --- | --- | --- | --- | --- | --- |
|  | **1990 (95% UI)** | **2021 (95% UI)** | **Relative change, 1990–2021(95% UI)** | **1990, per 100,000 (95% UI)** | **2021, per 100,000 (95% UI)** | **EAPC (95% CI)** |
| Afghanistan | 45.38 (24.66-69.32) | 31.12 (16.29-49.64) | -0.31 (-0.34--0.28) | 5.73 (3.11-8.75) | 3.22 (1.68-5.13) | -2.01 (-2.13--1.89) |
| Albania | 882.54 (509.96-1323.32) | 4179.59 (2541.6-6022.18) | 3.74 (3.98-3.55) | 16.17 (9.34-24.24) | 34.06 (20.71-49.08) | 2.31 (1.94-2.69) |
| Algeria | 1362.6 (792.52-2039.74) | 3671.87 (2380.74-5227.84) | 1.69 (2-1.56) | 14.25 (8.29-21.33) | 23.73 (15.38-33.78) | 1.61 (1.45-1.77) |
| American Samoa | 359.24 (212.3-578.32) | 914.24 (531.08-1486.14) | 1.54 (1.5-1.57) | 7.52 (4.45-12.11) | 6.56 (3.81-10.66) | -0.68 (-0.77--0.58) |
| Andorra | 112.12 (62.74-171.44) | 484.16 (277.31-740.76) | 3.32 (3.42-3.32) | 6.67 (3.73-10.19) | 10.81 (6.19-16.54) | 1.75 (1.62-1.87) |
| Angola | 0.21 (0.13-0.33) | 0.44 (0.24-0.69) | 1.1 (0.85-1.09) | 2.49 (1.5-3.79) | 2.31 (1.3-3.68) | -0.43 (-0.63--0.23) |
| Antigua and Barbuda | 57.84 (34.5-87.91) | 174.49 (104.48-269.84) | 2.02 (2.03-2.07) | 7.81 (4.66-11.87) | 8.03 (4.81-12.41) | 0.14 (-0.03-0.3) |
| Argentina | 241.88 (146.26-358.06) | 797.61 (476.88-1195.83) | 2.3 (2.26-2.34) | 13.59 (8.22-20.12) | 16.56 (9.9-24.83) | 0.54 (0.45-0.63) |
| Armenia | 0.55 (0.34-0.84) | 1.67 (0.95-2.56) | 2.04 (1.79-2.05) | 2.23 (1.37-3.42) | 2.32 (1.33-3.55) | 0.43 (0.35-0.5) |
| Australia | 1553.31 (939.23-2441.76) | 3563.86 (2034.98-5798.64) | 1.29 (1.17-1.37) | 9.61 (5.81-15.11) | 8.51 (4.86-13.85) | -0.47 (-0.62--0.33) |
| Austria | 116.37 (65.78-179.99) | 434.38 (257.84-653.77) | 2.73 (2.92-2.63) | 6.87 (3.88-10.62) | 12.71 (7.55-19.13) | 2.2 (1.94-2.47) |
| Azerbaijan | 1.97 (1.13-3.03) | 4.61 (2.72-7.03) | 1.34 (1.41-1.32) | 4.21 (2.41-6.46) | 5.06 (2.98-7.72) | 0.38 (0.13-0.64) |
| Bahrain | 0.76 (0.3-1.27) | 1.84 (0.71-3.14) | 1.42 (1.37-1.47) | 4.84 (1.89-8.14) | 3.07 (1.18-5.24) | -1.79 (-1.96--1.62) |
| Bangladesh | 1442.83 (771.38-2315.83) | 3482.25 (2015.15-5533.23) | 1.41 (1.61-1.39) | 9.71 (5.19-15.59) | 16.56 (9.58-26.31) | 1.84 (1.7-1.97) |
| Barbados | 732.81 (433.76-1091.03) | 1645.36 (955.58-2424.52) | 1.25 (1.2-1.22) | 27.53 (16.3-41) | 29.19 (16.96-43.02) | 0.61 (0.42-0.8) |
| Belarus | 0.42 (0.23-0.66) | 0.61 (0.36-0.94) | 0.45 (0.57-0.42) | 4.26 (2.36-6.68) | 4.02 (2.37-6.21) | -0.09 (-0.24-0.07) |
| Belgium | 980.27 (532.93-1594.01) | 548.36 (271.3-936.75) | -0.44 (-0.49--0.41) | 7.86 (4.27-12.79) | 4.04 (2-6.9) | -3.81 (-4.24--3.39) |
| Belize | 30.22 (17.61-45.81) | 66.78 (38.53-103.11) | 1.21 (1.19-1.25) | 8.81 (5.14-13.36) | 8.64 (4.98-13.34) | -0.17 (-0.33-0) |
| Benin | 1.13 (0.7-1.65) | 4.14 (2.4-6.14) | 2.66 (2.43-2.72) | 7.34 (4.53-10.73) | 8.27 (4.8-12.26) | 0.65 (0.38-0.91) |
| Bermuda | 169.56 (102.15-250.1) | 494.93 (291.46-738.32) | 1.92 (1.85-1.95) | 11.49 (6.92-16.95) | 10.09 (5.94-15.06) | -0.9 (-1.11--0.69) |
| Bhutan | 1147.3 (601.84-1940.29) | 4875.87 (2603.52-8142.45) | 3.25 (3.33-3.2) | 3.87 (2.03-6.55) | 9.34 (4.99-15.6) | 2.72 (2.59-2.86) |
| Bolivia | 93.46 (47.87-150.94) | 304.75 (171.9-474.56) | 2.26 (2.59-2.14) | 5.53 (2.83-8.94) | 10.4 (5.87-16.19) | 3.19 (2.61-3.77) |
| Bosnia and Herzegovina | 2.13 (1.28-3.26) | 8.28 (5.03-12.02) | 2.89 (2.93-2.69) | 13.88 (8.35-21.27) | 15.22 (9.25-22.1) | 0.7 (0.36-1.04) |
| Botswana | 29.84 (18.23-44.76) | 88.83 (51.76-135.87) | 1.98 (1.84-2.04) | 4.88 (2.98-7.32) | 5.32 (3.1-8.13) | 0.16 (0-0.32) |
| Brazil | 18.4 (9.17-29.98) | 22.98 (8.8-41.01) | 0.25 (-0.04-0.37) | 1.66 (0.83-2.7) | 2.19 (0.84-3.91) | 1 (0.79-1.21) |
| Brunei | 632.95 (392.67-960.39) | 1577.14 (920.48-2338.47) | 1.49 (1.34-1.43) | 16.17 (10.03-24.53) | 18.68 (10.9-27.7) | 0.48 (0.28-0.67) |
| Bulgaria | 0.54 (0.31-0.82) | 0.72 (0.41-1.15) | 0.33 (0.32-0.4) | 4.49 (2.6-6.8) | 3.6 (2.06-5.7) | -0.98 (-1.23--0.74) |
| Burkina Faso | 1560.79 (936.3-2303.89) | 3774.39 (2237.29-5561.73) | 1.42 (1.39-1.41) | 26.3 (15.78-38.82) | 19.42 (11.51-28.61) | -1.11 (-1.24--0.98) |
| Burundi | 28.72 (16.12-43.5) | 68.18 (38.91-99.25) | 1.37 (1.41-1.28) | 5.33 (2.99-8.07) | 5.75 (3.28-8.37) | 0.29 (0.07-0.51) |
| Cabo Verde | 1.13 (0.66-1.72) | 2.45 (1.4-3.81) | 1.17 (1.12-1.22) | 1.84 (1.07-2.81) | 2.17 (1.25-3.38) | 0.58 (0.35-0.82) |
| Cambodia | 250.55 (126.78-389.18) | 1655.11 (1015.27-2441.64) | 5.61 (7.01-5.27) | 5.03 (2.55-7.82) | 9.9 (6.07-14.6) | 2.69 (2.29-3.09) |
| Cameroon | 1.04 (0.58-1.62) | 3.65 (2.16-5.49) | 2.51 (2.72-2.39) | 7.1 (3.97-11.06) | 8.67 (5.13-13.04) | 0.74 (0.36-1.13) |
| Canada | 0.31 (0.18-0.48) | 0.41 (0.25-0.61) | 0.32 (0.39-0.27) | 12.27 (7.17-18.96) | 7.02 (4.2-10.4) | -2.24 (-2.51--1.96) |
| Central African Republic | 3.9 (2.38-5.77) | 14.77 (8.84-21.97) | 2.79 (2.71-2.81) | 8.96 (5.48-13.28) | 10.4 (6.23-15.47) | 0.26 (0.1-0.41) |
| Chad | 17.45 (9.9-26.58) | 38.62 (22.67-59.31) | 1.21 (1.29-1.23) | 5.93 (3.36-9.03) | 7.3 (4.29-11.21) | 0.36 (-0.34-1.06) |
| Chile | 0.28 (0.15-0.45) | 0.77 (0.43-1.19) | 1.75 (1.87-1.64) | 2.32 (1.27-3.73) | 3.01 (1.68-4.66) | 0.95 (0.58-1.33) |
| China | 295.47 (171.23-478.68) | 910.03 (497.56-1519.65) | 2.08 (1.91-2.17) | 12.45 (7.21-20.16) | 27.03 (14.78-45.14) | 2.72 (2.55-2.89) |
| Colombia | 2.4 (1.42-3.58) | 6.88 (3.95-10.4) | 1.87 (1.78-1.91) | 5.52 (3.26-8.23) | 6.16 (3.54-9.31) | 0.49 (0.25-0.73) |
| Comoros | 0.42 (0.24-0.64) | 1.17 (0.68-1.75) | 1.79 (1.83-1.73) | 7.21 (4.11-10.96) | 9.88 (5.77-14.82) | 1.33 (1.12-1.55) |
| Congo (Brazzaville) | 73.07 (35.72-124.74) | 138.99 (68.83-238.98) | 0.9 (0.93-0.92) | 15.87 (7.76-27.1) | 12.87 (6.37-22.12) | -0.64 (-0.75--0.53) |
| Cook Islands | 81.63 (48.63-119.99) | 210.57 (127.75-306.07) | 1.58 (1.63-1.55) | 27.59 (16.44-40.56) | 25.64 (15.55-37.27) | -0.46 (-0.6--0.32) |
| Costa Rica | 4.77 (2.72-7.48) | 17.37 (10.03-26.49) | 2.64 (2.69-2.54) | 3.47 (1.98-5.45) | 4.99 (2.88-7.6) | 1.25 (1.21-1.29) |
| Côte d'Ivoire | 306.74 (184.62-469.69) | 1857.31 (1133.73-2788) | 5.05 (5.14-4.94) | 10.65 (6.41-16.31) | 19.42 (11.85-29.15) | 2.08 (1.85-2.3) |
| Croatia | 45.54 (27.71-66.19) | 149.44 (89.02-221.94) | 2.28 (2.21-2.35) | 8.68 (5.28-12.62) | 9.67 (5.76-14.36) | 0.65 (0.52-0.79) |
| Cuba | 176.8 (108.99-255.12) | 829.57 (514.84-1224.6) | 3.69 (3.72-3.8) | 8.97 (5.53-12.94) | 14.83 (9.2-21.89) | 2.35 (2.05-2.66) |
| Cyprus | 70.41 (42.28-102.58) | 247.45 (148.12-373.4) | 2.51 (2.5-2.64) | 8.19 (4.92-11.93) | 8.94 (5.35-13.49) | 0.36 (0.3-0.42) |
| Czechia | 2.06 (1.23-3.14) | 4.62 (2.76-6.82) | 1.24 (1.24-1.17) | 9.12 (5.44-13.91) | 8.52 (5.08-12.57) | 0 (-0.12-0.12) |
| Denmark | 16.74 (8.02-27.53) | 58.29 (34.29-87.47) | 2.48 (3.28-2.18) | 4.62 (2.21-7.59) | 3.84 (2.26-5.76) | -0.86 (-0.97--0.74) |
| Djibouti | 30.34 (18.72-46.17) | 144.98 (89.25-214.73) | 3.78 (3.77-3.65) | 10.67 (6.58-16.24) | 15.14 (9.32-22.43) | 1.01 (0.83-1.18) |
| Dominica | 1.08 (0.63-1.66) | 0.91 (0.53-1.36) | -0.16 (-0.16--0.18) | 13.51 (7.97-20.91) | 6.94 (4.02-10.33) | -2.68 (-2.84--2.52) |
| Dominican Republic | 9.63 (4.66-15.9) | 13.54 (6.87-22.51) | 0.41 (0.47-0.42) | 5.56 (2.69-9.18) | 3.43 (1.74-5.7) | -1.71 (-1.8--1.62) |
| DR Congo | 4.04 (2.34-6.21) | 6.85 (3.89-10.67) | 0.7 (0.66-0.72) | 7.16 (4.15-11.01) | 5.01 (2.84-7.8) | -1.38 (-1.54--1.22) |
| Ecuador | 60.4 (38.29-86.44) | 126.65 (76.97-188.97) | 1.1 (1.01-1.19) | 12.35 (7.83-17.68) | 12.39 (7.53-18.48) | 0.31 (0.19-0.42) |
| Egypt | 58576.99 (36641.23-84137.3) | 130200.99 (77010.12-197259.59) | 1.22 (1.1-1.34) | 40.82 (25.53-58.62) | 34.36 (20.32-52.05) | -1.29 (-1.54--1.03) |
| El Salvador | 45.43 (27.34-68.51) | 180.62 (106.01-275.45) | 2.98 (2.88-3.02) | 7.9 (4.75-11.91) | 9.87 (5.79-15.05) | 0.87 (0.71-1.03) |
| Equatorial Guinea | 1034.35 (631.41-1494.7) | 3152.06 (1859.66-4925.21) | 2.05 (1.95-2.3) | 14.88 (9.08-21.5) | 14.63 (8.63-22.85) | -0.23 (-0.35--0.12) |
| Eritrea | 1223.39 (679.15-2041.58) | 1692.15 (915.05-2819.18) | 0.38 (0.35-0.38) | 16.79 (9.32-28.02) | 11.28 (6.1-18.79) | -1.42 (-1.5--1.35) |
| Estonia | 19.38 (11.72-28.49) | 77.96 (45.22-119.81) | 3.02 (2.86-3.21) | 7.8 (4.72-11.47) | 9.58 (5.56-14.72) | 0.89 (0.68-1.11) |
| Eswatini | 103.68 (55.29-168.1) | 259.96 (142.96-415.04) | 1.51 (1.59-1.47) | 15.76 (8.4-25.55) | 18.22 (10.02-29.1) | 0.58 (0.48-0.67) |
| Ethiopia | 35.17 (19.46-54.73) | 36.99 (21.23-56.38) | 0.05 (0.09-0.03) | 7.23 (4-11.24) | 4.7 (2.7-7.17) | -0.42 (-0.88-0.05) |
| Federated States of Micronesia | 75.84 (40.92-122.47) | 42.13 (22.84-65.75) | -0.44 (-0.44--0.46) | 3.35 (1.81-5.41) | 1.77 (0.96-2.77) | -2.11 (-2.41--1.82) |
| Fiji | 38.49 (22.44-58.62) | 110.31 (66.26-164.37) | 1.87 (1.95-1.8) | 11.55 (6.73-17.59) | 10.49 (6.3-15.63) | -0.27 (-0.35--0.19) |
| Finland | 24.96 (15.15-37.34) | 103.63 (63.29-149.16) | 3.15 (3.18-2.99) | 10.3 (6.25-15.41) | 13.94 (8.51-20.06) | 0.69 (0.41-0.97) |
| France | 30.56 (16.19-46.88) | 77.54 (38.2-125.28) | 1.54 (1.36-1.67) | 2.75 (1.45-4.21) | 5.2 (2.56-8.4) | 1.42 (1.05-1.79) |
| Gabon | 92.3 (51.83-138.18) | 40.15 (22.14-61.37) | -0.57 (-0.57--0.56) | 17.92 (10.06-26.84) | 4.75 (2.62-7.26) | -4.65 (-5.2--4.1) |
| Georgia | 28.16 (15.4-42.8) | 39.99 (21.45-63.04) | 0.42 (0.39-0.47) | 8.11 (4.44-12.33) | 5.09 (2.73-8.02) | -1.68 (-1.81--1.56) |
| Germany | 31.02 (16.73-48.89) | 37.95 (20.89-60.15) | 0.22 (0.25-0.23) | 3.56 (1.92-5.61) | 1.98 (1.09-3.14) | -2.06 (-2.3--1.82) |
| Ghana | 69.3 (41.49-104.81) | 361.54 (213.8-538.2) | 4.22 (4.15-4.14) | 4.43 (2.65-6.69) | 6.91 (4.09-10.29) | 1.47 (1.13-1.81) |
| Greece | 26.87 (15.63-41.21) | 59 (33.5-96.43) | 1.2 (1.14-1.34) | 2.59 (1.51-3.97) | 3.6 (2.04-5.88) | 1.51 (1.3-1.72) |
| Greenland | 310.34 (148.11-502.39) | 221.81 (110.56-364.82) | -0.29 (-0.25--0.27) | 6.2 (2.96-10.04) | 3.69 (1.84-6.07) | -1.34 (-1.56--1.12) |
| Grenada | 41.68 (23.32-64.4) | 53.47 (28.8-84.25) | 0.28 (0.23-0.31) | 5.68 (3.18-8.78) | 4.88 (2.63-7.69) | -0.3 (-0.5--0.1) |
| Guam | 63.93 (31.66-103.22) | 109.04 (52.58-171.48) | 0.71 (0.66-0.66) | 3.07 (1.52-4.96) | 3.89 (1.87-6.11) | -0.28 (-0.74-0.18) |
| Guatemala | 1639.92 (987.01-2490.87) | 6323.13 (3740.5-9922.02) | 2.86 (2.79-2.98) | 11.1 (6.68-16.85) | 14.6 (8.64-22.91) | 0.79 (0.69-0.89) |
| Guinea | 63.17 (36.59-96.57) | 238.75 (147.3-360.08) | 2.78 (3.03-2.73) | 3.02 (1.75-4.61) | 3.93 (2.43-5.93) | 1.06 (0.93-1.19) |
| Guinea-Bissau | 23.71 (14.18-35.68) | 74.94 (42.73-114.16) | 2.16 (2.01-2.2) | 6.53 (3.91-9.84) | 7.57 (4.32-11.53) | 0.54 (0.38-0.7) |
| Guyana | 1.72 (1.03-2.58) | 9.93 (5.74-14.41) | 4.77 (4.57-4.59) | 6.14 (3.65-9.2) | 6.12 (3.54-8.88) | -0.37 (-0.61--0.12) |
| Haiti | 178.24 (103.14-267.58) | 255.45 (146.97-393.14) | 0.43 (0.42-0.47) | 3.99 (2.31-5.98) | 2.31 (1.33-3.55) | -2.06 (-2.22--1.91) |
| Honduras | 8.9 (5.3-13.16) | 42.19 (24.2-63.99) | 3.74 (3.57-3.86) | 4.14 (2.47-6.12) | 3.36 (1.93-5.09) | -0.43 (-0.61--0.25) |
| Hungary | 21.93 (10.41-36.06) | 57.25 (30.68-89.82) | 1.61 (1.95-1.49) | 5.09 (2.42-8.37) | 7.82 (4.19-12.27) | 1.29 (1.12-1.46) |
| Iceland | 167.99 (99.92-263.03) | 728.15 (435.14-1168.06) | 3.33 (3.35-3.44) | 3.64 (2.17-5.71) | 5.61 (3.35-8.99) | 1.68 (1.49-1.88) |
| India | 1.4 (0.82-2.16) | 7.2 (4.44-10.58) | 4.14 (4.41-3.9) | 1.53 (0.9-2.37) | 1.54 (0.95-2.27) | 0.55 (0.11-1) |
| Indonesia | 24.95 (14.72-37.69) | 131.47 (77.82-192.26) | 4.27 (4.29-4.1) | 6.58 (3.88-9.94) | 13.41 (7.94-19.62) | 3.11 (2.79-3.44) |
| Iran | 608.42 (351.62-951.3) | 1141.64 (653.98-1889.06) | 0.88 (0.86-0.99) | 7.9 (4.56-12.34) | 9.42 (5.4-15.59) | 0.63 (0.36-0.9) |
| Iraq | 32.36 (18.9-48.4) | 78.11 (46.3-117.27) | 1.41 (1.45-1.42) | 2.56 (1.5-3.83) | 2.01 (1.19-3.02) | -0.73 (-0.82--0.63) |
| Ireland | 1.68 (0.94-2.57) | 3.73 (1.84-6.43) | 1.22 (0.96-1.5) | 1.54 (0.87-2.36) | 2.14 (1.05-3.68) | 0.02 (-0.25-0.29) |
| Israel | 62.9 (31.88-106.89) | 83.81 (41.31-144.46) | 0.33 (0.3-0.35) | 18.98 (9.62-32.25) | 13.87 (6.84-23.91) | -1.04 (-1.11--0.97) |
| Italy | 17.29 (10.77-25.28) | 35.55 (20.95-53.64) | 1.06 (0.95-1.12) | 5.56 (3.46-8.13) | 4.25 (2.5-6.41) | -0.47 (-0.64--0.29) |
| Jamaica | 6.85 (3.8-10.91) | 8.91 (4.26-16.06) | 0.3 (0.12-0.47) | 1.91 (1.06-3.04) | 2.04 (0.97-3.67) | -0.61 (-0.84--0.37) |
| Japan | 1666.42 (924.79-2672.87) | 1644.85 (883.98-2740.92) | -0.01 (-0.04-0.03) | 5.28 (2.93-8.47) | 3.87 (2.08-6.45) | -1.44 (-1.75--1.12) |
| Jordan | 129 (67.06-208.2) | 251.76 (129.14-403.36) | 0.95 (0.93-0.94) | 5.01 (2.6-8.08) | 7.92 (4.06-12.68) | 1.62 (1.42-1.82) |
| Kazakhstan | 77.46 (46.12-120.72) | 208.77 (120.78-314.1) | 1.7 (1.62-1.6) | 3.3 (1.97-5.15) | 3.47 (2.01-5.23) | 0.35 (0.25-0.46) |
| Kenya | 3.06 (1.83-4.73) | 6.79 (4.07-10.33) | 1.22 (1.22-1.18) | 3.04 (1.82-4.7) | 2.18 (1.3-3.31) | -0.69 (-0.83--0.54) |
| Kiribati | 45.5 (27.49-68.77) | 96.18 (57.01-142.19) | 1.11 (1.07-1.07) | 5.11 (3.09-7.72) | 3.19 (1.89-4.72) | -1.32 (-1.44--1.21) |
| Kuwait | 30.75 (15.95-50.78) | 71.63 (35.9-120.83) | 1.33 (1.25-1.38) | 15.76 (8.17-26.03) | 11.47 (5.75-19.34) | -0.92 (-1--0.85) |
| Kyrgyzstan | 44.21 (22.17-73.8) | 187.95 (102.99-291.87) | 3.25 (3.65-2.95) | 3.72 (1.87-6.22) | 9.34 (5.12-14.5) | 2.99 (2.52-3.45) |
| Laos | 1.87 (0.94-2.94) | 8.04 (4.55-12.59) | 3.3 (3.84-3.28) | 3.93 (1.96-6.15) | 8.26 (4.67-12.92) | 1.93 (1.61-2.25) |
| Latvia | 31.25 (19.42-47.13) | 86.81 (49.51-133.25) | 1.78 (1.55-1.83) | 3.54 (2.2-5.35) | 3.67 (2.1-5.64) | 0.29 (0.18-0.39) |
| Lebanon | 82.25 (46.04-127.53) | 284.05 (164.82-430.5) | 2.45 (2.58-2.38) | 12.08 (6.76-18.73) | 21.64 (12.56-32.8) | 2.05 (1.95-2.14) |
| Lesotho | 1.46 (0.84-2.26) | 4.59 (2.84-6.76) | 2.14 (2.38-1.99) | 14.78 (8.51-22.88) | 17.34 (10.72-25.53) | 0.43 (0.23-0.64) |
| Liberia | 30.61 (17.53-46.95) | 100.45 (58.53-149.18) | 2.28 (2.34-2.18) | 3.52 (2.01-5.39) | 4.3 (2.5-6.38) | 0.66 (0.46-0.86) |
| Libya | 0.5 (0.29-0.74) | 0.91 (0.55-1.42) | 0.82 (0.9-0.92) | 13.74 (7.96-20.26) | 10.83 (6.47-16.78) | -0.96 (-1.07--0.85) |
| Lithuania | 0.46 (0.28-0.68) | 4.82 (2.9-7.28) | 9.48 (9.36-9.71) | 2.81 (1.74-4.14) | 3.15 (1.89-4.75) | 0.61 (0.44-0.78) |
| Luxembourg | 101.43 (54.42-159.03) | 423.09 (226.79-659.93) | 3.17 (3.17-3.15) | 4.28 (2.3-6.72) | 12.11 (6.49-18.89) | 4.5 (4.18-4.83) |
| Madagascar | 701.55 (421.96-1033.6) | 2493.41 (1455.28-3603.21) | 2.55 (2.45-2.49) | 11.74 (7.06-17.3) | 15.09 (8.81-21.81) | 1.41 (1.2-1.62) |
| Malawi | 30.8 (18.89-46.37) | 86.6 (50.74-131.14) | 1.81 (1.69-1.83) | 3.84 (2.36-5.78) | 3.85 (2.26-5.83) | 0.29 (0.2-0.37) |
| Malaysia | 40.27 (22.26-61.95) | 258.89 (151.27-399.03) | 5.43 (5.8-5.44) | 4.97 (2.75-7.64) | 12.95 (7.57-19.97) | 3.07 (2.74-3.4) |
| Maldives | 3.17 (1.91-4.69) | 28.72 (16.15-46.72) | 8.06 (7.46-8.96) | 5.65 (3.4-8.36) | 4 (2.25-6.51) | -1.51 (-1.66--1.36) |
| Mali | 54.62 (32.56-84.34) | 62.43 (36.14-94.39) | 0.14 (0.11-0.12) | 4.5 (2.68-6.94) | 5.08 (2.94-7.68) | 0.81 (0.68-0.95) |
| Malta | 1751.19 (1059.89-2619.41) | 4213.25 (2502.04-6182.09) | 1.41 (1.36-1.36) | 23.12 (13.99-34.58) | 18.03 (10.71-26.46) | -0.76 (-0.83--0.7) |
| Marshall Islands | 24.25 (12.74-42.16) | 43.07 (22.45-71.08) | 0.78 (0.76-0.69) | 22.35 (11.74-38.86) | 19.06 (9.94-31.46) | -0.48 (-0.51--0.44) |
| Mauritania | 49.9 (27.06-78.76) | 24.8 (13.83-38.61) | -0.5 (-0.49--0.51) | 6.46 (3.51-10.2) | 2.35 (1.31-3.66) | -3.4 (-3.8--2.99) |
| Mauritius | 7.82 (4.84-11.67) | 25.04 (15.58-37.01) | 2.2 (2.22-2.17) | 19.71 (12.2-29.4) | 25.14 (15.64-37.16) | 0.82 (0.77-0.87) |
| Mexico | 0.11 (0.07-0.17) | 0.15 (0.08-0.22) | 0.36 (0.14-0.29) | 9.86 (5.83-14.52) | 8.1 (4.64-12.06) | -1.08 (-1.25--0.9) |
| Moldova | 16793.8 (10081.23-25720.96) | 52537.9 (31012.47-82115.17) | 2.13 (2.08-2.19) | 21.9 (13.15-33.55) | 26.13 (15.43-40.84) | 0.62 (0.51-0.73) |
| Monaco | 52.82 (29.69-81.82) | 126.68 (68.36-191.44) | 1.4 (1.3-1.34) | 7.83 (4.4-12.13) | 14.01 (7.56-21.17) | 2.14 (1.91-2.37) |
| Mongolia | 3.98 (2.22-6.1) | 13.49 (8-20.82) | 2.39 (2.6-2.41) | 5.49 (3.07-8.42) | 8.55 (5.07-13.19) | 2.12 (1.84-2.4) |
| Montenegro | 1062.49 (525.75-1795.03) | 3124.88 (1767.48-5129.32) | 1.94 (2.36-1.86) | 6.98 (3.45-11.79) | 13.76 (7.78-22.58) | 2.64 (2.05-3.22) |
| Morocco | 554.67 (318.84-834.67) | 2415.99 (1572.72-3336.35) | 3.36 (3.93-3) | 16.9 (9.72-25.44) | 32.61 (21.23-45.04) | 1.82 (1.61-2.04) |
| Mozambique | 439.47 (265.56-656.31) | 1183.04 (724.4-1779.77) | 1.69 (1.73-1.71) | 28.55 (17.25-42.64) | 29.89 (18.3-44.97) | 0.21 (0.16-0.25) |
| Myanmar | 9.17 (4.7-15.63) | 13.31 (6.82-23.09) | 0.45 (0.45-0.48) | 20.31 (10.41-34.62) | 14.7 (7.53-25.5) | -1.06 (-1.26--0.86) |
| Namibia | 1651.19 (991.47-2568.64) | 2772.2 (1617.33-4551.26) | 0.68 (0.63-0.77) | 18.11 (10.87-28.17) | 13.98 (8.16-22.96) | -1.01 (-1.08--0.95) |
| Nauru | 93.55 (46.83-167.78) | 180.33 (84.76-317.79) | 0.93 (0.81-0.89) | 15.02 (7.52-26.93) | 9.33 (4.38-16.43) | -1.56 (-1.66--1.46) |
| Nepal | 13.42 (7.25-20.36) | 9.73 (5.38-15.01) | -0.27 (-0.26--0.26) | 4.13 (2.23-6.27) | 1.37 (0.76-2.12) | -3.83 (-4.13--3.53) |
| Netherlands | 1400.16 (792.24-2180.15) | 4829.54 (2751.68-7127.78) | 2.45 (2.47-2.27) | 6.67 (3.78-10.39) | 15.33 (8.73-22.62) | 4.31 (3.79-4.84) |
| New Zealand | 415.45 (247.97-637.51) | 1694.75 (1004.71-2577.11) | 3.08 (3.05-3.04) | 12.55 (7.49-19.25) | 28.97 (17.17-44.05) | 3.06 (2.83-3.29) |
| Nicaragua | 29.02 (14.73-49.97) | 44.67 (22.2-78.01) | 0.54 (0.51-0.56) | 15.37 (7.8-26.47) | 12.49 (6.2-21.81) | -0.59 (-0.63--0.55) |
| Niger | 1074.38 (618.4-1732.81) | 2011.7 (1124.55-3238.13) | 0.87 (0.82-0.87) | 31.83 (18.32-51.34) | 25.5 (14.26-41.05) | -0.73 (-1.03--0.42) |
| Nigeria | 82.19 (46.81-125.29) | 50.35 (26.08-79.51) | -0.39 (-0.44--0.37) | 4.2 (2.39-6.41) | 1.08 (0.56-1.71) | -5.35 (-5.73--4.97) |
| Niue | 20.38 (9.91-36.05) | 37.73 (18.71-66.2) | 0.85 (0.89-0.84) | 11.45 (5.57-20.25) | 8.68 (4.31-15.23) | -0.81 (-0.85--0.77) |
| North Korea | 2.14 (1.26-3.22) | 3.02 (1.87-4.51) | 0.41 (0.48-0.4) | 14.82 (8.77-22.31) | 12.26 (7.58-18.33) | -0.68 (-0.81--0.55) |
| North Macedonia | 81.05 (34.31-140.33) | 369.41 (194.57-612.91) | 3.56 (4.67-3.37) | 7.48 (3.17-12.96) | 22.78 (12-37.79) | 4.33 (3.97-4.68) |
| Northern Mariana Islands | 7.22 (3.51-11.4) | 29.2 (16.63-45.12) | 3.04 (3.74-2.96) | 7.73 (3.76-12.21) | 16.43 (9.36-25.39) | 2.43 (1.84-3.01) |
| Norway | 144.01 (91.1-216) | 373.49 (221.12-571.29) | 1.59 (1.43-1.64) | 7.4 (4.68-11.1) | 12.64 (7.48-19.33) | 1.28 (1.07-1.5) |
| Oman | 0.12 (0.07-0.18) | 0.27 (0.16-0.42) | 1.25 (1.29-1.33) | 5.61 (3.2-8.66) | 5.66 (3.32-8.97) | 0.24 (0.15-0.34) |
| Pakistan | 193.15 (112.08-296.33) | 64 (34.77-102.79) | -0.67 (-0.69--0.65) | 8.34 (4.84-12.8) | 2.22 (1.21-3.57) | -5.64 (-6.03--5.25) |
| Palau | 11.76 (5.92-20.48) | 14.94 (7.2-25.62) | 0.27 (0.22-0.25) | 12.01 (6.04-20.92) | 8.36 (4.03-14.33) | -0.96 (-1.03--0.89) |
| Palestine | 241.84 (139.26-372.83) | 689.07 (410.17-1013.74) | 1.85 (1.95-1.72) | 9.92 (5.71-15.29) | 17.71 (10.54-26.06) | 2.18 (1.88-2.47) |
| Panama | 385.02 (198.87-668.34) | 762.81 (395.72-1302.73) | 0.98 (0.99-0.95) | 14.58 (7.53-25.31) | 12.63 (6.55-21.57) | -0.43 (-0.73--0.12) |
| Papua New Guinea | 12.72 (6.82-19.62) | 20.55 (10.31-33.4) | 0.62 (0.51-0.7) | 3.87 (2.07-5.97) | 3.47 (1.74-5.63) | -0.2 (-0.27--0.14) |
| Paraguay | 5.71 (2.87-9.64) | 7.42 (3.87-12.54) | 0.3 (0.35-0.3) | 17.87 (8.98-30.19) | 9.38 (4.89-15.85) | -1.94 (-2.19--1.68) |
| Peru | 89.1 (45.19-151.42) | 140.15 (72.88-239.52) | 0.57 (0.61-0.58) | 23.67 (12.01-40.23) | 17.51 (9.1-29.92) | -1.29 (-1.43--1.16) |
| Philippines | 45.9 (24.67-77.49) | 41.65 (21.4-74.72) | -0.09 (-0.13--0.04) | 32.82 (17.64-55.41) | 23.07 (11.85-41.39) | -1.32 (-1.46--1.17) |
| Poland | 5.85 (2.91-10.27) | 11.57 (6.19-19.39) | 0.98 (1.13-0.89) | 18.01 (8.96-31.66) | 14.31 (7.66-23.99) | -0.65 (-0.69--0.62) |
| Portugal | 307.55 (173.18-470.44) | 833.93 (479.54-1285.16) | 1.71 (1.77-1.73) | 5.53 (3.11-8.45) | 8.93 (5.14-13.77) | 1.68 (1.41-1.95) |
| Puerto Rico | 7581.34 (4211.15-12140.99) | 23093.18 (13310.74-39371.55) | 2.05 (2.16-2.24) | 14.45 (8.03-23.14) | 23.04 (13.28-39.27) | 1.47 (1.25-1.69) |
| Qatar | 0.45 (0.26-0.7) | 2.1 (1.18-3.26) | 3.67 (3.54-3.66) | 4.27 (2.47-6.65) | 9.04 (5.08-14) | 2.42 (2.19-2.65) |
| Romania | 9.38 (4.77-15.7) | 11.79 (5.98-19.97) | 0.26 (0.25-0.27) | 14.69 (7.47-24.6) | 10.26 (5.2-17.38) | -1.06 (-1.14--0.99) |
| Russia | 107.13 (54.31-180.64) | 139.88 (72.41-235.41) | 0.31 (0.33-0.3) | 19.36 (9.81-32.64) | 15.31 (7.93-25.77) | -0.67 (-0.75--0.58) |
| Rwanda | 3.04 (1.54-5.33) | 10.79 (5.38-18.55) | 2.55 (2.49-2.48) | 14.15 (7.16-24.83) | 10.41 (5.19-17.89) | -1.04 (-1.24--0.84) |
| Saint Kitts and Nevis | 124.9 (65.55-213.39) | 231.59 (118.15-405.72) | 0.85 (0.8-0.9) | 18.68 (9.8-31.91) | 16 (8.16-28.03) | -0.34 (-0.38--0.29) |
| Saint Lucia | 23.7 (11.89-41.29) | 38.8 (19.34-64.21) | 0.64 (0.63-0.56) | 14.28 (7.16-24.87) | 10.81 (5.39-17.9) | -0.92 (-0.95--0.89) |
| Saint Vincent and the Grenadines | 27.58 (13.76-46.63) | 37.04 (17.91-63.2) | 0.34 (0.3-0.36) | 14.63 (7.3-24.75) | 11.22 (5.42-19.14) | -1.13 (-1.38--0.87) |
| Samoa | 16.71 (8.81-26.67) | 17.29 (8.29-28.67) | 0.03 (-0.06-0.07) | 2.67 (1.41-4.27) | 2.63 (1.26-4.36) | -0.58 (-1.06--0.1) |
| San Marino | 306.23 (168.73-492.13) | 596.04 (326.49-977.63) | 0.95 (0.93-0.99) | 22.98 (12.66-36.93) | 15.93 (8.73-26.13) | -1.71 (-1.93--1.5) |
| São Tomé and Príncipe | 0.95 (0.55-1.38) | 1.39 (0.82-2.1) | 0.46 (0.49-0.52) | 15.89 (9.22-23.22) | 11.06 (6.51-16.67) | -1.31 (-1.45--1.17) |
| Saudi Arabia | 320.03 (176.71-491.13) | 639.21 (406.38-942.89) | 1 (1.3-0.92) | 12.21 (6.74-18.73) | 16.91 (10.75-24.94) | 1.85 (1.65-2.06) |
| Senegal | 26.3 (12.71-44.69) | 54.22 (26.7-95.12) | 1.06 (1.1-1.13) | 14.88 (7.19-25.29) | 12.31 (6.06-21.59) | -0.51 (-0.59--0.43) |
| Serbia | 695.95 (376.66-1142.95) | 1240.36 (671.62-2065.22) | 0.78 (0.78-0.81) | 21.66 (11.72-35.57) | 18.12 (9.81-30.17) | -0.68 (-0.76--0.6) |
| Seychelles | 153.1 (77.79-259.13) | 224.95 (112.12-399.88) | 0.47 (0.44-0.54) | 18.12 (9.21-30.67) | 12.18 (6.07-21.65) | -1.57 (-1.73--1.4) |
| Sierra Leone | 119.73 (62.78-214.28) | 182.07 (93.98-310.18) | 0.52 (0.5-0.45) | 19.04 (9.98-34.07) | 15.57 (8.04-26.53) | -0.91 (-1.03--0.79) |
| Singapore | 5.97 (3.39-9.08) | 20.1 (12.28-30.48) | 2.37 (2.62-2.36) | 4.91 (2.79-7.46) | 5.94 (3.63-9.01) | 0.4 (0.28-0.52) |
| Slovakia | 78.68 (40.81-136.57) | 233.12 (120.86-406.18) | 1.96 (1.96-1.97) | 17.9 (9.28-31.07) | 16.74 (8.68-29.18) | 0.04 (-0.07-0.15) |
| Slovenia | 193.44 (100.36-325.88) | 257.83 (130.35-445.74) | 0.33 (0.3-0.37) | 19.8 (10.27-33.35) | 14.41 (7.29-24.91) | -1.19 (-1.33--1.05) |
| Solomon Islands | 69.64 (35.67-117.27) | 128.68 (66.49-212.89) | 0.85 (0.86-0.82) | 13.18 (6.75-22.19) | 9.97 (5.15-16.49) | -0.61 (-0.79--0.42) |
| Somalia | 166.28 (91.46-258.58) | 240.08 (128.23-388.63) | 0.44 (0.4-0.5) | 7.95 (4.37-12.36) | 7.57 (4.04-12.25) | 0.26 (-0.31-0.84) |
| South Africa | 77.37 (40.23-133.39) | 214.21 (111.48-358.49) | 1.77 (1.77-1.69) | 21.49 (11.17-37.05) | 22.71 (11.82-38.01) | 0.05 (-0.09-0.19) |
| South Korea | 6.02 (3.49-8.9) | 14.62 (8.73-21.99) | 1.43 (1.5-1.47) | 4.15 (2.4-6.13) | 3.4 (2.03-5.11) | -0.47 (-0.53--0.41) |
| South Sudan | 96.5 (48.87-165.88) | 200.4 (103.43-341.83) | 1.08 (1.12-1.06) | 20.62 (10.44-35.45) | 18.95 (9.78-32.33) | -0.56 (-0.7--0.41) |
| Spain | 8.11 (4.17-13.35) | 32.03 (18.22-48.95) | 2.95 (3.37-2.67) | 5.8 (2.98-9.56) | 9.17 (5.21-14.01) | 1.01 (0.75-1.26) |
| Sri Lanka | 1.24 (0.75-1.92) | 2.74 (1.62-4.07) | 1.21 (1.16-1.12) | 12.48 (7.55-19.27) | 9.56 (5.64-14.2) | -1.01 (-1.08--0.95) |
| Sudan | 380.93 (194.42-663.22) | 638.55 (321.46-1081.59) | 0.68 (0.65-0.63) | 21.11 (10.78-36.76) | 15.49 (7.8-26.24) | -0.8 (-0.97--0.63) |
| Suriname | 0.49 (0.29-0.71) | 0.9 (0.51-1.36) | 0.84 (0.76-0.92) | 5.09 (3.01-7.44) | 4.32 (2.44-6.56) | -0.5 (-0.74--0.25) |
| Sweden | 129.71 (70.34-200.37) | 593.1 (340.21-888.57) | 3.57 (3.84-3.43) | 9.94 (5.39-15.36) | 30.81 (17.67-46.15) | 4.22 (3.89-4.56) |
| Switzerland | 0.8 (0.39-1.3) | 2.12 (1.06-3.45) | 1.65 (1.72-1.65) | 14.59 (7.02-23.59) | 15.61 (7.83-25.39) | 0.62 (0.48-0.77) |
| Syria | 0.78 (0.46-1.21) | 3.05 (1.84-4.57) | 2.91 (3-2.78) | 6.03 (3.53-9.41) | 8.1 (4.88-12.14) | 0.89 (0.65-1.13) |
| Taiwan (province of China) | 0.8 (0.46-1.25) | 1.77 (1.05-2.67) | 1.21 (1.28-1.14) | 7.23 (4.19-11.26) | 11.32 (6.7-17.05) | 1.46 (1.35-1.57) |
| Tajikistan | 231.76 (117.76-397.81) | 361.17 (185.3-607.87) | 0.56 (0.57-0.53) | 22.16 (11.26-38.03) | 15.23 (7.81-25.63) | -1.23 (-1.27--1.19) |
| Tanzania | 0.41 (0.23-0.61) | 1.4 (0.81-2.13) | 2.41 (2.52-2.49) | 2.95 (1.68-4.4) | 4.36 (2.51-6.63) | 1.17 (1-1.34) |
| Thailand | 2698.13 (1702.33-3810.33) | 6244.94 (3938.96-8472.61) | 1.31 (1.31-1.22) | 19.39 (12.23-27.38) | 28.24 (17.82-38.32) | 1 (0.89-1.12) |
| The Bahamas | 1002.04 (617.93-1499.21) | 2147.99 (1280.75-3110.51) | 1.14 (1.07-1.07) | 14.37 (8.86-21.5) | 12.3 (7.33-17.81) | -0.64 (-0.99--0.28) |
| The Gambia | 19.64 (10.07-34.51) | 41.95 (21.7-69.96) | 1.14 (1.15-1.03) | 21.13 (10.83-37.12) | 17.31 (8.95-28.86) | -0.68 (-0.74--0.61) |
| Timor-Leste | 1.78 (1.07-2.65) | 2.46 (1.49-3.64) | 0.38 (0.39-0.37) | 18.73 (11.29-27.91) | 18.32 (11.11-27.07) | 0.03 (-0.06-0.11) |
| Togo | 0.06 (0.03-0.09) | 0.06 (0.03-0.09) | 0 (0-0) | 8.17 (4.53-12.56) | 6.4 (3.75-9.82) | -1.02 (-1.12--0.93) |
| Tokelau | 0.06 (0.04-0.09) | 0.04 (0.02-0.05) | -0.33 (-0.5--0.44) | 16.16 (9.69-23.92) | 9.26 (5.52-14.01) | -1.56 (-1.66--1.45) |
| Tonga | 103.01 (57.48-156.72) | 209.92 (118.83-316.17) | 1.04 (1.07-1.02) | 3.85 (2.15-5.86) | 5.71 (3.23-8.6) | 1.04 (0.8-1.29) |
| Trinidad and Tobago | 132.87 (70.07-230.7) | 176.71 (86.22-293.36) | 0.33 (0.23-0.27) | 20.05 (10.57-34.81) | 15.96 (7.79-26.49) | -0.69 (-0.75--0.64) |
| Tunisia | 63.3 (32.91-107.76) | 106.82 (54.94-175.13) | 0.69 (0.67-0.63) | 19.79 (10.29-33.68) | 12.92 (6.64-21.17) | -1.22 (-1.27--1.16) |
| Türkiye | 129.02 (65.08-221.07) | 226.83 (116.99-388.81) | 0.76 (0.8-0.76) | 17.84 (9-30.57) | 15.09 (7.78-25.86) | -0.6 (-0.65--0.56) |
| Turkmenistan | 0.37 (0.22-0.56) | 1.05 (0.63-1.57) | 1.84 (1.86-1.8) | 15.33 (9.01-23.05) | 10.77 (6.5-16.12) | -2.03 (-2.35--1.71) |
| Tuvalu | 0.24 (0.14-0.37) | 0.47 (0.27-0.7) | 0.96 (0.93-0.89) | 15.29 (9.05-22.98) | 11.24 (6.55-16.65) | -1.38 (-1.53--1.23) |
| Uganda | 116.34 (59.94-197.29) | 230.34 (113.69-400.9) | 0.98 (0.9-1.03) | 15.71 (8.09-26.65) | 11.32 (5.59-19.7) | -1.1 (-1.16--1.03) |
| UK | 0.03 (0.02-0.04) | 0.03 (0.02-0.04) | 0 (0-0) | 12.11 (7.08-18.16) | 11.98 (7.01-17.64) | -0.09 (-0.26-0.07) |
| Ukraine | 0.25 (0.15-0.38) | 0.5 (0.29-0.75) | 1 (0.93-0.97) | 3.89 (2.28-6.01) | 3.88 (2.23-5.8) | -0.51 (-1--0.02) |
| United Arab Emirates | 66.79 (38.86-102.31) | 211.99 (133.6-321.96) | 2.17 (2.44-2.15) | 11.01 (6.4-16.86) | 18.16 (11.44-27.58) | 1.74 (1.52-1.95) |
| Uruguay | 30.55 (16.74-47.15) | 38.29 (21.38-59.35) | 0.25 (0.28-0.26) | 6.52 (3.57-10.07) | 3.73 (2.08-5.78) | -1.61 (-1.97--1.26) |
| United States | 81.64 (41.18-139.48) | 87.33 (44.17-148.56) | 0.07 (0.07-0.07) | 19.69 (9.93-33.63) | 14.02 (7.09-23.86) | -1.21 (-1.35--1.08) |
| Uzbekistan | 7.15 (3.59-11.95) | 8 (4.12-13.71) | 0.12 (0.15-0.15) | 18.11 (9.09-30.28) | 10.44 (5.37-17.89) | -1.68 (-1.8--1.57) |
| Vanuatu | 103.63 (51.47-176.35) | 141.1 (69.03-235.54) | 0.36 (0.34-0.34) | 22.36 (11.11-38.06) | 15.2 (7.43-25.37) | -1.22 (-1.26--1.17) |
| Venezuela | 307.03 (175.05-482.48) | 1357.94 (824.47-2037.77) | 3.42 (3.71-3.22) | 11.07 (6.31-17.4) | 18.03 (10.95-27.06) | 0.89 (0.43-1.36) |
| Viet Nam | 1.53 (0.76-2.61) | 1.6 (0.82-2.8) | 0.05 (0.08-0.07) | 13.64 (6.75-23.3) | 8.77 (4.5-15.32) | -1.46 (-1.59--1.32) |
| Virgin Islands | 66.22 (38.51-101.35) | 115.35 (68.74-175.74) | 0.74 (0.78-0.73) | 4.45 (2.59-6.82) | 3.7 (2.21-5.64) | -0.4 (-0.61--0.2) |
| Yemen | 79.77 (40.4-138.33) | 190.82 (93.03-327.68) | 1.39 (1.3-1.37) | 12.45 (6.3-21.58) | 10.64 (5.19-18.27) | -0.33 (-0.39--0.27) |
| Zambia | 10.08 (5.09-17.63) | 20.19 (10.14-34.49) | 1 (0.99-0.96) | 18.2 (9.18-31.83) | 13.05 (6.55-22.31) | -0.83 (-0.9--0.75) |
| Zimbabwe | 123.22 (64.23-209.93) | 243.69 (128.07-418.38) | 0.98 (0.99-0.99) | 12.07 (6.29-20.56) | 8.94 (4.7-15.34) | -0.96 (-1.03--0.9) |

**Table S10. Prevalent cases, prevalence rates, and EAPC trends of HF impairment under the COPD cause category across 204 countries and territories from 1990 to 2021 among adults aged ≥55 years.**

| **Location** | **Prevalent Cases** | |  | **Prevalent Rates** | |  |
| --- | --- | --- | --- | --- | --- | --- |
|  | **1990  (95% UI)** | **2021  (95% UI)** | **Relative change, 1990–2021(95% UI)** | **1990, per 100,000 (95% UI)** | **2021, per 100,000 (95% UI)** | **EAPC (95% CI)** |
| Afghanistan | 497.13 (339.01-684.81) | 342.87 (224.22-479.08) | -0.31 (-0.34--0.3) | 62.73 (42.78-86.42) | 35.43 (23.17-49.5) | -1.98 (-2.1--1.86) |
| Albania | 9384.02 (6785.47-12305.35) | 42581.37 (31963.88-55548.59) | 3.54 (3.71-3.51) | 171.9 (124.3-225.41) | 347 (260.48-452.67) | 2.13 (1.76-2.5) |
| Algeria | 14690.95 (10340.56-19736.53) | 37253.14 (31055.46-44918.9) | 1.54 (2-1.28) | 153.66 (108.16-206.44) | 240.73 (200.68-290.27) | 1.37 (1.21-1.54) |
| American Samoa | 4050.84 (2817.47-5565.17) | 10293.8 (7094.34-14999.84) | 1.54 (1.52-1.7) | 84.85 (59.02-116.57) | 73.82 (50.87-107.56) | -0.68 (-0.78--0.59) |
| Andorra | 1141.27 (787.22-1506.32) | 4305.11 (3119.01-5830.28) | 2.77 (2.96-2.87) | 67.86 (46.81-89.57) | 96.15 (69.66-130.21) | 1.35 (1.2-1.5) |
| Angola | 2.31 (1.72-2.96) | 4.62 (3.3-6.21) | 1 (0.92-1.1) | 26.76 (19.94-34.2) | 24.51 (17.5-32.9) | -0.55 (-0.76--0.34) |
| Antigua and Barbuda | 657.74 (493.21-878.67) | 1962.59 (1475.2-2629.69) | 1.98 (1.99-1.99) | 88.77 (66.57-118.59) | 90.28 (67.86-120.97) | 0.1 (-0.06-0.27) |
| Argentina | 2716.63 (2010.87-3579.17) | 8910.88 (6607.07-11701.6) | 2.28 (2.29-2.27) | 152.64 (112.98-201.1) | 185.01 (137.18-242.96) | 0.52 (0.43-0.62) |
| Armenia | 5.75 (4.29-7.72) | 16.77 (12.27-22.29) | 1.92 (1.86-1.89) | 23.42 (17.49-31.44) | 23.3 (17.05-30.98) | 0.24 (0.16-0.31) |
| Australia | 17493.07 (12449.63-23847.86) | 39983.84 (27060.94-60057.9) | 1.29 (1.17-1.52) | 108.28 (77.06-147.61) | 95.47 (64.62-143.41) | -0.49 (-0.63--0.34) |
| Austria | 1287.25 (966.89-1724.73) | 4825.78 (3652.77-6253.97) | 2.75 (2.78-2.63) | 75.96 (57.05-101.77) | 141.23 (106.9-183.02) | 2.22 (1.95-2.49) |
| Azerbaijan | 20.74 (15.18-27.81) | 46.74 (34.9-62.15) | 1.25 (1.3-1.23) | 44.23 (32.38-59.31) | 51.29 (38.31-68.21) | 0.18 (-0.09-0.46) |
| Bahrain | 8.23 (3.6-12.92) | 19.45 (8.38-31.24) | 1.36 (1.33-1.42) | 52.56 (22.97-82.49) | 32.48 (13.99-52.17) | -1.87 (-2.05--1.7) |
| Bangladesh | 15539.21 (10179.56-22381.87) | 35321.02 (24151.49-50032.42) | 1.27 (1.37-1.24) | 104.62 (68.54-150.69) | 167.95 (114.84-237.91) | 1.6 (1.46-1.74) |
| Barbados | 8186.8 (6023.67-10954.7) | 18341.62 (13724.11-24595.79) | 1.24 (1.28-1.25) | 307.62 (226.34-411.62) | 325.45 (243.52-436.42) | 0.61 (0.41-0.81) |
| Belarus | 4.61 (3.42-6.14) | 6.57 (4.74-8.85) | 0.43 (0.39-0.44) | 46.92 (34.86-62.58) | 43.57 (31.43-58.68) | -0.13 (-0.29-0.02) |
| Belgium | 10803.81 (7261.3-15687.1) | 5992.94 (3574.34-9492.61) | -0.45 (-0.51--0.39) | 86.66 (58.24-125.83) | 44.14 (26.32-69.91) | -3.82 (-4.25--3.4) |
| Belize | 339.91 (254.11-453.46) | 742.32 (547.21-1005.63) | 1.18 (1.15-1.22) | 99.12 (74.1-132.23) | 96.01 (70.78-130.07) | -0.18 (-0.35--0.01) |
| Benin | 11.9 (9.01-15.78) | 42.8 (31.68-57.03) | 2.6 (2.52-2.61) | 77.32 (58.55-102.55) | 85.46 (63.25-113.87) | 0.55 (0.27-0.82) |
| Bermuda | 1912.83 (1405.02-2514.58) | 5483.21 (4102.27-7190.59) | 1.87 (1.92-1.86) | 129.61 (95.2-170.38) | 111.84 (83.67-146.66) | -0.96 (-1.17--0.75) |
| Bhutan | 11086.08 (6698.1-16670.1) | 39702.77 (25873.56-58706.91) | 2.58 (2.86-2.52) | 37.44 (22.62-56.29) | 76.06 (49.57-112.46) | 2.15 (2.02-2.27) |
| Bolivia | 999.6 (603.37-1443.53) | 3137.62 (2222.28-4275.7) | 2.14 (2.68-1.96) | 59.18 (35.72-85.46) | 107.06 (75.82-145.89) | 3.03 (2.46-3.61) |
| Bosnia and Herzegovina | 23.25 (17.33-30.72) | 89.29 (66.96-115.94) | 2.84 (2.86-2.77) | 151.65 (113.02-200.42) | 164.15 (123.09-213.14) | 0.66 (0.33-1) |
| Botswana | 330.57 (248.31-433.69) | 982.24 (737.28-1310.62) | 1.97 (1.97-2.02) | 54.07 (40.61-70.93) | 58.78 (44.12-78.43) | 0.16 (0-0.31) |
| Brazil | 199.04 (129.15-298.98) | 248.92 (101.61-407.92) | 0.25 (-0.21-0.36) | 17.92 (11.63-26.92) | 23.71 (9.68-38.85) | 1.02 (0.8-1.25) |
| Brunei | 7175.27 (5419.39-9535.28) | 17845.42 (13359.53-23358.52) | 1.49 (1.47-1.45) | 183.3 (138.44-243.59) | 211.39 (158.25-276.69) | 0.46 (0.27-0.66) |
| Bulgaria | 5.87 (4.36-7.87) | 7.68 (5.58-10.13) | 0.31 (0.28-0.29) | 48.7 (36.14-65.27) | 38.23 (27.8-50.45) | -1.09 (-1.35--0.83) |
| Burkina Faso | 17529.19 (12919.39-23283.1) | 42504.34 (31735.12-55126.48) | 1.42 (1.46-1.37) | 295.39 (217.71-392.35) | 218.67 (163.26-283.6) | -1.11 (-1.24--0.98) |
| Burundi | 316.45 (229.34-422.26) | 749.94 (554.37-980.54) | 1.37 (1.42-1.32) | 58.67 (42.52-78.29) | 63.28 (46.78-82.74) | 0.28 (0.06-0.5) |
| Cabo Verde | 11.93 (8.58-16.56) | 26.18 (18.93-35.59) | 1.19 (1.21-1.15) | 19.46 (14-27.01) | 23.25 (16.81-31.6) | 0.54 (0.28-0.81) |
| Cambodia | 2594.8 (1564.41-3659.73) | 16232.12 (12167.25-21255.81) | 5.26 (6.78-4.81) | 52.14 (31.43-73.54) | 97.06 (72.75-127.1) | 2.45 (2.05-2.86) |
| Cameroon | 11.35 (8.13-15.26) | 39.4 (28.96-52.14) | 2.47 (2.56-2.42) | 77.59 (55.57-104.28) | 93.62 (68.82-123.91) | 0.68 (0.3-1.07) |
| Canada | 3.53 (2.52-4.86) | 4.59 (3.34-6.18) | 0.3 (0.33-0.27) | 138.3 (98.98-190.62) | 78.37 (56.98-105.44) | -2.28 (-2.56--2.01) |
| Central African Republic | 44.19 (34.11-57.97) | 166.08 (123.59-222.62) | 2.76 (2.62-2.84) | 101.67 (78.47-133.38) | 116.94 (87.02-156.74) | 0.23 (0.08-0.38) |
| Chad | 191.34 (142.5-258.34) | 422.39 (313.82-561.59) | 1.21 (1.2-1.17) | 64.97 (48.38-87.71) | 79.86 (59.34-106.18) | 0.34 (-0.36-1.05) |
| Chile | 3.06 (2.12-4.42) | 8.19 (5.93-11.12) | 1.68 (1.8-1.52) | 25.46 (17.68-36.78) | 32.22 (23.31-43.74) | 0.85 (0.49-1.22) |
| China | 3053.36 (2116.47-4297.89) | 9248.36 (6159.37-14084.47) | 2.03 (1.91-2.28) | 128.61 (89.15-181.03) | 274.72 (182.96-418.37) | 2.65 (2.45-2.85) |
| Colombia | 26.3 (20.05-33.79) | 74.28 (56.17-97.07) | 1.82 (1.8-1.87) | 60.43 (46.08-77.64) | 66.5 (50.29-86.9) | 0.45 (0.21-0.69) |
| Comoros | 4.5 (3.28-6.1) | 12.48 (9.04-16.87) | 1.77 (1.76-1.77) | 76.68 (55.9-103.84) | 105.53 (76.45-142.63) | 1.35 (1.13-1.56) |
| Congo (Brazzaville) | 813.43 (467.3-1303.53) | 1529.63 (902.97-2438.45) | 0.88 (0.93-0.87) | 176.71 (101.52-283.18) | 141.61 (83.6-225.75) | -0.67 (-0.79--0.55) |
| Cook Islands | 915.65 (681.09-1196.8) | 2361.63 (1770.18-3094.75) | 1.58 (1.6-1.59) | 309.47 (230.2-404.5) | 287.55 (215.53-376.81) | -0.47 (-0.61--0.32) |
| Costa Rica | 50.91 (36.58-68.84) | 178.45 (128.42-239.96) | 2.51 (2.51-2.49) | 37.08 (26.64-50.14) | 51.23 (36.87-68.89) | 1.08 (1.03-1.14) |
| Côte d'Ivoire | 3422.53 (2568.93-4517.21) | 20448.16 (15254.07-26463.47) | 4.97 (4.94-4.86) | 118.88 (89.23-156.9) | 213.81 (159.5-276.71) | 2.04 (1.81-2.26) |
| Croatia | 466.3 (347.13-621.82) | 1431.14 (1080.85-1888.55) | 2.07 (2.11-2.04) | 88.93 (66.21-118.59) | 92.6 (69.94-122.2) | 0.43 (0.3-0.57) |
| Cuba | 1521.03 (1166.36-1959.83) | 5801.52 (4458.55-7490.21) | 2.81 (2.82-2.82) | 77.14 (59.15-99.4) | 103.72 (79.71-133.91) | 1.55 (1.24-1.86) |
| Cyprus | 748.68 (563.13-974.08) | 2239.74 (1677.32-2991.53) | 1.99 (1.98-2.07) | 87.07 (65.49-113.29) | 80.93 (60.61-108.09) | -0.2 (-0.27--0.13) |
| Czechia | 22.97 (16.88-31.11) | 51.72 (38.2-69.78) | 1.25 (1.26-1.24) | 101.59 (74.68-137.58) | 95.34 (70.42-128.64) | 0.02 (-0.11-0.14) |
| Denmark | 184.52 (103.42-268.03) | 600.78 (425.86-800.9) | 2.26 (3.12-1.99) | 50.89 (28.52-73.92) | 39.56 (28.04-52.73) | -1.09 (-1.21--0.96) |
| Djibouti | 331.39 (251.04-423.28) | 1523.91 (1137.88-2016.29) | 3.6 (3.53-3.76) | 116.53 (88.27-148.83) | 159.16 (118.84-210.59) | 0.86 (0.69-1.03) |
| Dominica | 12.07 (8.99-16.41) | 10.13 (7.53-13.44) | -0.16 (-0.16--0.18) | 151.57 (112.92-206.1) | 76.99 (57.21-102.14) | -2.71 (-2.87--2.55) |
| Dominican Republic | 105.69 (64.32-152.4) | 146.51 (83.83-217.75) | 0.39 (0.3-0.43) | 60.99 (37.12-87.95) | 37.11 (21.23-55.15) | -1.76 (-1.85--1.67) |
| DR Congo | 45.57 (33.54-61.28) | 76.61 (56.16-103.77) | 0.68 (0.67-0.69) | 80.86 (59.5-108.71) | 56.03 (41.07-75.89) | -1.4 (-1.56--1.24) |
| Ecuador | 669.76 (511.36-862.1) | 1377.81 (1044.26-1786.68) | 1.06 (1.04-1.07) | 136.98 (104.58-176.31) | 134.74 (102.12-174.72) | 0.24 (0.13-0.35) |
| Egypt | 657107.39 (485538.74-859332.96) | 1454040.5 (1040909.52-1987551.4) | 1.21 (1.14-1.31) | 457.86 (338.31-598.76) | 383.7 (274.68-524.48) | -1.31 (-1.56--1.05) |
| El Salvador | 489.8 (365.39-653.08) | 1868.53 (1362.48-2484.3) | 2.81 (2.73-2.8) | 85.17 (63.54-113.56) | 102.06 (74.42-135.7) | 0.75 (0.58-0.91) |
| Equatorial Guinea | 11364.61 (8358.32-14738.56) | 33444.18 (23035.58-47051.71) | 1.94 (1.76-2.19) | 163.46 (120.22-211.98) | 155.19 (106.89-218.33) | -0.35 (-0.47--0.23) |
| Eritrea | 13555.91 (8462.93-20865.65) | 18557.54 (11503.47-28203.38) | 0.37 (0.36-0.35) | 186.08 (116.17-286.42) | 123.7 (76.68-188) | -1.46 (-1.53--1.38) |
| Estonia | 214.86 (164.59-281.04) | 855.59 (645.58-1116.33) | 2.98 (2.92-2.97) | 86.47 (66.24-113.1) | 105.11 (79.31-137.14) | 0.87 (0.65-1.09) |
| Eswatini | 1131.55 (742.89-1627.28) | 2706.65 (1827.02-3819.35) | 1.39 (1.46-1.35) | 171.98 (112.91-247.32) | 189.75 (128.08-267.76) | 0.42 (0.32-0.52) |
| Ethiopia | 385.53 (265.3-520.08) | 397.56 (290.62-542.66) | 0.03 (0.1-0.04) | 79.2 (54.5-106.84) | 50.54 (36.95-68.99) | -0.51 (-0.98--0.04) |
| Federated States of Micronesia | 823.45 (550.82-1145.34) | 453.06 (300.96-638.17) | -0.45 (-0.45--0.44) | 36.38 (24.34-50.61) | 19.06 (12.66-26.85) | -2.14 (-2.43--1.84) |
| Fiji | 424.67 (323.52-557.55) | 1203.8 (866.95-1608.88) | 1.83 (1.68-1.89) | 127.41 (97.06-167.27) | 114.47 (82.44-152.98) | -0.3 (-0.38--0.22) |
| Finland | 274.96 (205.02-368.23) | 1082.55 (823.69-1423.26) | 2.94 (3.02-2.87) | 113.48 (84.62-151.98) | 145.59 (110.78-191.41) | 0.48 (0.18-0.77) |
| France | 333.48 (217.83-471.04) | 850.46 (513.61-1199.13) | 1.55 (1.36-1.55) | 29.96 (19.57-42.31) | 57.03 (34.44-80.41) | 1.45 (1.08-1.83) |
| Gabon | 1030.4 (715.15-1396.64) | 447.17 (309.68-609.19) | -0.57 (-0.57--0.56) | 200.11 (138.89-271.24) | 52.87 (36.62-72.03) | -4.65 (-5.2--4.1) |
| Georgia | 306.47 (210.3-413.87) | 428.93 (298.23-591.28) | 0.4 (0.42-0.43) | 88.31 (60.6-119.26) | 54.55 (37.93-75.2) | -1.72 (-1.85--1.6) |
| Germany | 341.23 (237.22-461.31) | 414.65 (265.66-579.6) | 0.22 (0.12-0.26) | 39.12 (27.2-52.89) | 21.64 (13.87-30.25) | -2.11 (-2.35--1.86) |
| Ghana | 763.52 (580.85-1010.29) | 3948.74 (2973.65-5157.27) | 4.17 (4.12-4.1) | 48.76 (37.09-64.52) | 75.47 (56.83-98.57) | 1.42 (1.08-1.76) |
| Greece | 291.48 (213.65-386.12) | 633.32 (449.74-861.67) | 1.17 (1.11-1.23) | 28.1 (20.59-37.22) | 38.61 (27.42-52.53) | 1.52 (1.31-1.74) |
| Greenland | 3348.89 (1982.92-5157.37) | 2386.38 (1442.34-3373.43) | -0.29 (-0.27--0.35) | 66.93 (39.63-103.07) | 39.74 (24.02-56.17) | -1.35 (-1.57--1.13) |
| Grenada | 456.91 (307.98-626.66) | 583.82 (375.51-817.54) | 0.28 (0.22-0.3) | 62.29 (41.99-85.43) | 53.3 (34.28-74.64) | -0.32 (-0.52--0.13) |
| Guam | 702.21 (422.79-1009.92) | 1194.02 (694.86-1694.35) | 0.7 (0.64-0.68) | 33.76 (20.33-48.56) | 42.56 (24.77-60.4) | -0.31 (-0.77-0.16) |
| Guatemala | 18344.45 (13373.36-24656.4) | 69200.51 (49011.62-97261.52) | 2.77 (2.66-2.94) | 124.13 (90.49-166.84) | 159.79 (113.17-224.58) | 0.72 (0.61-0.82) |
| Guinea | 703.34 (518.57-915.55) | 2643.38 (1986.57-3472.91) | 2.76 (2.83-2.79) | 33.57 (24.75-43.7) | 43.52 (32.71-57.18) | 1.05 (0.92-1.18) |
| Guinea-Bissau | 261.33 (197.95-338.15) | 808.18 (604.79-1052.08) | 2.09 (2.06-2.11) | 72.04 (54.57-93.21) | 81.63 (61.08-106.26) | 0.48 (0.31-0.64) |
| Guyana | 18.89 (14.32-24.66) | 105.58 (79.92-137.14) | 4.59 (4.58-4.56) | 67.34 (51.04-87.91) | 65.02 (49.22-84.46) | -0.51 (-0.77--0.25) |
| Haiti | 1966.28 (1487.41-2538.01) | 2779.2 (2075.32-3692.89) | 0.41 (0.4-0.46) | 43.97 (33.27-56.76) | 25.12 (18.75-33.37) | -2.11 (-2.26--1.96) |
| Honduras | 95.75 (72.95-126.24) | 430.52 (321.19-567.28) | 3.5 (3.4-3.49) | 44.53 (33.93-58.71) | 34.24 (25.55-45.12) | -0.63 (-0.82--0.44) |
| Hungary | 239.42 (133.54-345.46) | 597.67 (370.84-836.48) | 1.5 (1.78-1.42) | 55.59 (31-80.21) | 81.63 (50.65-114.24) | 1.15 (0.98-1.31) |
| Iceland | 1877.19 (1362.03-2616.56) | 8171.43 (5707.55-11671.36) | 3.35 (3.19-3.46) | 40.72 (29.55-56.76) | 62.9 (43.93-89.84) | 1.7 (1.51-1.89) |
| India | 12.95 (9.71-17.2) | 57.12 (43.66-74.93) | 3.41 (3.5-3.36) | 14.16 (10.62-18.81) | 12.25 (9.36-16.07) | -0.04 (-0.67-0.58) |
| Indonesia | 272.09 (205.21-355.45) | 1420.58 (1071.51-1834.23) | 4.22 (4.22-4.16) | 71.77 (54.13-93.75) | 144.95 (109.33-187.16) | 3.1 (2.77-3.42) |
| Iran | 6653.94 (4794.63-9278.62) | 12250.12 (8376.16-17791.45) | 0.84 (0.75-0.92) | 86.35 (62.22-120.41) | 101.11 (69.14-146.85) | 0.58 (0.3-0.85) |
| Iraq | 356.06 (266.03-459.88) | 838.02 (637.75-1107.2) | 1.35 (1.4-1.41) | 28.21 (21.08-36.43) | 21.57 (16.41-28.5) | -0.8 (-0.9--0.7) |
| Ireland | 18.08 (12.96-24.49) | 39.89 (23.32-63.47) | 1.21 (0.8-1.59) | 16.6 (11.89-22.48) | 22.88 (13.38-36.4) | 0.03 (-0.24-0.29) |
| Israel | 692.72 (409.36-1119.31) | 917.94 (522.19-1476.24) | 0.33 (0.28-0.32) | 208.99 (123.5-337.69) | 151.96 (86.44-244.38) | -1.05 (-1.13--0.97) |
| Italy | 194.47 (152.71-251.3) | 396.41 (298.49-509.27) | 1.04 (0.95-1.03) | 62.52 (49.1-80.79) | 47.4 (35.69-60.89) | -0.53 (-0.7--0.35) |
| Jamaica | 67.7 (46.09-94.19) | 94.99 (55.98-148.1) | 0.4 (0.21-0.57) | 18.89 (12.86-26.28) | 21.71 (12.8-33.85) | -0.3 (-0.55--0.04) |
| Japan | 18379.58 (12234.91-26422.37) | 18067.35 (11765.78-26630.78) | -0.02 (-0.04-0.01) | 58.23 (38.76-83.71) | 42.51 (27.68-62.65) | -1.44 (-1.75--1.13) |
| Jordan | 1410.39 (876.04-1986.24) | 2740.36 (1734.44-3878.98) | 0.94 (0.98-0.95) | 54.76 (34.01-77.11) | 86.17 (54.54-121.98) | 1.62 (1.42-1.82) |
| Kazakhstan | 870.63 (644.82-1138.25) | 2325.92 (1745.44-3094.92) | 1.67 (1.71-1.72) | 37.13 (27.5-48.54) | 38.71 (29.05-51.51) | 0.33 (0.22-0.43) |
| Kenya | 33.96 (25.11-45.06) | 73.87 (54.59-97.16) | 1.18 (1.17-1.16) | 33.79 (24.98-44.83) | 23.65 (17.48-31.11) | -0.75 (-0.89--0.61) |
| Kiribati | 494.22 (374.6-644.16) | 976.73 (729.16-1270.58) | 0.98 (0.95-0.97) | 55.47 (42.05-72.3) | 32.44 (24.22-42.2) | -1.56 (-1.67--1.44) |
| Kuwait | 339.4 (193.56-536.38) | 783 (455.31-1280.27) | 1.31 (1.35-1.39) | 173.98 (99.22-274.96) | 125.34 (72.89-204.95) | -0.94 (-1.01--0.86) |
| Kyrgyzstan | 458.78 (272.98-670.22) | 1833.94 (1213.79-2560.29) | 3 (3.45-2.82) | 38.64 (22.99-56.45) | 91.12 (60.31-127.21) | 2.73 (2.26-3.2) |
| Laos | 20.39 (12.03-29.17) | 84.87 (58.22-117.12) | 3.16 (3.84-3.02) | 42.73 (25.22-61.14) | 87.14 (59.78-120.27) | 1.8 (1.48-2.12) |
| Latvia | 347.48 (260.61-450.2) | 946.33 (699.82-1273.35) | 1.72 (1.69-1.83) | 39.41 (29.56-51.06) | 40.05 (29.62-53.89) | 0.25 (0.15-0.35) |
| Lebanon | 892.87 (602.25-1208.34) | 2868.69 (2063-3823.81) | 2.21 (2.43-2.16) | 131.17 (88.47-177.51) | 218.59 (157.2-291.37) | 1.75 (1.67-1.84) |
| Lesotho | 15.49 (10.92-21.05) | 48.08 (35.16-62.9) | 2.1 (2.22-1.99) | 156.93 (110.71-213.29) | 181.71 (132.88-237.68) | 0.38 (0.18-0.58) |
| Liberia | 340.49 (253.64-451.54) | 1117.3 (846.92-1442.6) | 2.28 (2.34-2.19) | 39.1 (29.13-51.86) | 47.81 (36.24-61.73) | 0.64 (0.44-0.85) |
| Libya | 5.61 (4.1-7.46) | 10.2 (7.26-14.26) | 0.82 (0.77-0.91) | 153.46 (111.98-204.04) | 120.81 (85.98-168.78) | -0.95 (-1.06--0.84) |
| Lithuania | 5 (3.76-6.51) | 50.8 (38.91-65.74) | 9.16 (9.35-9.1) | 30.6 (23-39.82) | 33.16 (25.4-42.92) | 0.48 (0.32-0.64) |
| Luxembourg | 1074.03 (721.46-1532.47) | 4507.15 (3063.39-6422.82) | 3.2 (3.25-3.19) | 45.36 (30.47-64.72) | 129.02 (87.69-183.85) | 4.57 (4.24-4.91) |
| Madagascar | 7807.51 (6025.59-10038.51) | 27541.51 (20842.07-35776.63) | 2.53 (2.46-2.56) | 130.68 (100.85-168.02) | 166.7 (126.15-216.55) | 1.39 (1.17-1.6) |
| Malawi | 344.6 (259.54-448.7) | 965.79 (721.02-1264.03) | 1.8 (1.78-1.82) | 42.97 (32.36-55.95) | 42.94 (32.06-56.21) | 0.27 (0.18-0.36) |
| Malaysia | 434.44 (291.41-597.57) | 2712.1 (1925-3699.5) | 5.24 (5.61-5.19) | 53.6 (35.95-73.72) | 135.7 (96.32-185.11) | 2.94 (2.6-3.28) |
| Maldives | 33.51 (25.52-43.23) | 293.97 (211.32-389.89) | 7.77 (7.28-8.02) | 59.7 (45.47-77.03) | 40.94 (29.43-54.3) | -1.64 (-1.8--1.48) |
| Mali | 614.56 (450.29-800.97) | 702.03 (510.75-935.94) | 0.14 (0.13-0.17) | 50.57 (37.05-65.91) | 57.13 (41.57-76.17) | 0.81 (0.67-0.96) |
| Malta | 19645.43 (14877.39-26039.95) | 46543.37 (35092.95-61510.3) | 1.37 (1.36-1.36) | 259.36 (196.41-343.78) | 199.19 (150.19-263.25) | -0.81 (-0.88--0.74) |
| Marshall Islands | 265.25 (152.97-429.97) | 470.26 (272.12-751.78) | 0.77 (0.78-0.75) | 244.45 (140.98-396.25) | 208.13 (120.44-332.72) | -0.48 (-0.51--0.45) |
| Mauritania | 554.51 (376.1-755.95) | 274.46 (192.48-379.46) | -0.51 (-0.49--0.5) | 71.83 (48.72-97.92) | 25.99 (18.23-35.94) | -3.38 (-3.78--2.98) |
| Mauritius | 87.38 (67.43-113.18) | 277.06 (211.86-355.87) | 2.17 (2.14-2.14) | 220.19 (169.91-285.19) | 278.2 (212.73-357.33) | 0.8 (0.75-0.85) |
| Mexico | 1.28 (0.93-1.69) | 1.64 (1.2-2.19) | 0.28 (0.29-0.3) | 109.84 (79.97-145.63) | 90.16 (65.7-120.07) | -1.08 (-1.26--0.9) |
| Moldova | 191152.78 (141693.93-252460.41) | 592395.07 (420959.74-825952.61) | 2.1 (1.97-2.27) | 249.32 (184.81-329.29) | 294.66 (209.39-410.83) | 0.59 (0.48-0.71) |
| Monaco | 573.58 (385.23-798.15) | 1354.3 (860.23-1901.03) | 1.36 (1.23-1.38) | 85 (57.09-118.29) | 149.76 (95.13-210.22) | 2.07 (1.85-2.3) |
| Mongolia | 41.62 (29.07-57.86) | 124.98 (89.77-170.25) | 2 (2.09-1.94) | 57.45 (40.12-79.87) | 79.2 (56.89-107.89) | 1.63 (1.35-1.91) |
| Montenegro | 11516.03 (6836.13-17020.03) | 32474.83 (21804.04-46822.56) | 1.82 (2.19-1.75) | 75.67 (44.92-111.83) | 142.96 (95.98-206.11) | 2.45 (1.88-3.03) |
| Morocco | 6028.72 (4442.12-8068.08) | 24861.5 (19834.22-30682.8) | 3.12 (3.47-2.8) | 183.72 (135.37-245.86) | 335.61 (267.75-414.19) | 1.61 (1.41-1.81) |
| Mozambique | 4956.21 (3723.25-6527.66) | 13172.47 (9760.01-17326.82) | 1.66 (1.62-1.65) | 321.97 (241.88-424.06) | 332.82 (246.6-437.78) | 0.18 (0.13-0.22) |
| Myanmar | 99.95 (59.02-162.1) | 144.98 (82.71-229.43) | 0.45 (0.4-0.42) | 221.35 (130.72-359) | 160.13 (91.36-253.4) | -1.06 (-1.25--0.87) |
| Namibia | 18611.86 (13396.14-25590.45) | 31087.02 (21741.67-44433) | 0.67 (0.62-0.74) | 204.09 (146.9-280.61) | 156.8 (109.66-224.11) | -1.04 (-1.11--0.97) |
| Nauru | 1036.68 (598.33-1698.72) | 1985.53 (1118.16-3292.28) | 0.92 (0.87-0.94) | 166.42 (96.05-272.71) | 102.68 (57.83-170.26) | -1.59 (-1.69--1.48) |
| Nepal | 149.1 (100.26-211.65) | 105.68 (70.82-147.91) | -0.29 (-0.29--0.3) | 45.91 (30.87-65.17) | 14.93 (10.01-20.9) | -3.9 (-4.2--3.59) |
| Netherlands | 14934.4 (10342.22-20437.73) | 50918.25 (36560.1-68398.03) | 2.41 (2.54-2.35) | 71.17 (49.29-97.4) | 161.57 (116.01-217.04) | 4.27 (3.75-4.79) |
| New Zealand | 4510.26 (3362.44-5874.2) | 17963.69 (12829.05-24079.33) | 2.98 (2.82-3.1) | 136.22 (101.55-177.42) | 307.03 (219.27-411.55) | 2.95 (2.72-3.18) |
| Nicaragua | 319.12 (185.79-524.85) | 490.5 (286.97-793.7) | 0.54 (0.54-0.51) | 169.01 (98.4-277.98) | 137.13 (80.23-221.89) | -0.61 (-0.66--0.57) |
| Niger | 11686.2 (7467.58-16908.43) | 21764.94 (13828.34-32481.28) | 0.86 (0.85-0.92) | 346.21 (221.23-500.92) | 275.91 (175.3-411.75) | -0.74 (-1.04--0.44) |
| Nigeria | 886.88 (622.3-1252.45) | 537.78 (332.57-774.97) | -0.39 (-0.47--0.38) | 45.35 (31.82-64.04) | 11.57 (7.15-16.67) | -5.4 (-5.8--5) |
| Niue | 225.5 (123.45-385) | 412.62 (233.18-680.21) | 0.83 (0.89-0.77) | 126.68 (69.35-216.28) | 94.96 (53.66-156.54) | -0.85 (-0.9--0.81) |
| North Korea | 23.83 (17.96-31.5) | 33.77 (25.17-44.89) | 0.42 (0.4-0.43) | 165.33 (124.62-218.55) | 137.14 (102.23-182.34) | -0.67 (-0.8--0.54) |
| North Macedonia | 853.65 (400.54-1420.35) | 3936.64 (2449.75-5826.16) | 3.61 (5.12-3.1) | 78.83 (36.99-131.16) | 242.74 (151.06-359.25) | 4.33 (3.97-4.69) |
| Northern Mariana Islands | 78.54 (45.8-115.14) | 308.15 (217.38-430.06) | 2.92 (3.75-2.74) | 84.1 (49.04-123.3) | 173.41 (122.33-242.01) | 2.32 (1.74-2.9) |
| Norway | 1554.99 (1177.55-1902.51) | 3986.09 (2951.17-5010.39) | 1.56 (1.51-1.63) | 79.91 (60.51-97.77) | 134.89 (99.86-169.55) | 1.22 (1-1.43) |
| Oman | 1.33 (0.97-1.78) | 2.98 (2.2-4.03) | 1.24 (1.27-1.26) | 62.52 (45.66-83.31) | 63.17 (46.71-85.6) | 0.23 (0.13-0.32) |
| Pakistan | 2113.11 (1491.54-2897.79) | 696.1 (459.3-1039.91) | -0.67 (-0.69--0.64) | 91.28 (64.43-125.18) | 24.2 (15.97-36.15) | -5.69 (-6.09--5.29) |
| Palau | 129.7 (73.57-212.96) | 163.59 (94.09-262.63) | 0.26 (0.28-0.23) | 132.48 (75.15-217.52) | 91.52 (52.64-146.93) | -0.99 (-1.06--0.93) |
| Palestine | 2579.93 (1925.05-3396.41) | 7121.85 (5184.53-9407.37) | 1.76 (1.69-1.77) | 105.82 (78.96-139.31) | 183.05 (133.26-241.79) | 2.03 (1.73-2.33) |
| Panama | 4248.5 (2462.22-6860.8) | 8397.19 (4973.84-13720.76) | 0.98 (1.02-1) | 160.88 (93.24-259.81) | 139.03 (82.35-227.17) | -0.43 (-0.74--0.12) |
| Papua New Guinea | 139.54 (95.45-188.33) | 224.65 (151.95-312.08) | 0.61 (0.59-0.66) | 42.42 (29.01-57.25) | 37.89 (25.63-52.64) | -0.24 (-0.3--0.19) |
| Paraguay | 63.79 (36.3-102.53) | 81.32 (48.19-132.27) | 0.27 (0.33-0.29) | 199.85 (113.72-321.21) | 102.81 (60.93-167.23) | -2 (-2.26--1.75) |
| Peru | 987.17 (582.19-1585.21) | 1540.34 (917.52-2447.55) | 0.56 (0.58-0.54) | 262.3 (154.69-421.21) | 192.42 (114.61-305.74) | -1.32 (-1.46--1.19) |
| Philippines | 505.71 (312.4-787.14) | 459.35 (267.95-747.85) | -0.09 (-0.14--0.05) | 361.63 (223.4-562.89) | 254.41 (148.4-414.2) | -1.31 (-1.46--1.17) |
| Poland | 64.89 (37.93-104.26) | 127.69 (74.76-203.2) | 0.97 (0.97-0.95) | 199.94 (116.86-321.24) | 157.97 (92.49-251.39) | -0.66 (-0.7--0.62) |
| Portugal | 3292.84 (2301.19-4451.84) | 8747.34 (5713.62-12088.67) | 1.66 (1.48-1.72) | 59.17 (41.35-80) | 93.72 (61.22-129.52) | 1.61 (1.35-1.87) |
| Puerto Rico | 80907.41 (54028.17-114020.22) | 241761.21 (166401.67-343627.13) | 1.99 (2.08-2.01) | 154.22 (102.98-217.34) | 241.16 (165.99-342.78) | 1.41 (1.19-1.63) |
| Qatar | 4.55 (3.34-6.18) | 19.97 (13.93-27.53) | 3.39 (3.17-3.45) | 42.98 (31.58-58.43) | 85.73 (59.81-118.21) | 2.15 (1.91-2.38) |
| Romania | 103.78 (59.59-166.56) | 129.56 (73.19-206.59) | 0.25 (0.23-0.24) | 162.59 (93.35-260.92) | 112.79 (63.72-179.85) | -1.07 (-1.15--0.99) |
| Russia | 1183.6 (688.15-1855.39) | 1538.52 (922.68-2447.23) | 0.3 (0.34-0.32) | 213.88 (124.35-335.27) | 168.41 (101-267.88) | -0.65 (-0.75--0.56) |
| Rwanda | 33.53 (19.64-54.35) | 118.24 (67-194.62) | 2.53 (2.41-2.58) | 156.28 (91.54-253.33) | 114.02 (64.61-187.67) | -1.06 (-1.26--0.86) |
| Saint Kitts and Nevis | 1384.66 (798.87-2237.68) | 2541.78 (1502-4114.79) | 0.84 (0.88-0.84) | 207.08 (119.47-334.65) | 175.61 (103.77-284.29) | -0.36 (-0.41--0.31) |
| Saint Lucia | 260.35 (146.28-415.54) | 421.98 (237.45-674.47) | 0.62 (0.62-0.62) | 156.82 (88.11-250.31) | 117.62 (66.19-188) | -0.95 (-0.97--0.92) |
| Saint Vincent and the Grenadines | 303.72 (173.52-494.67) | 406.26 (232.37-647.09) | 0.34 (0.34-0.31) | 161.17 (92.08-262.51) | 123.07 (70.39-196.01) | -1.15 (-1.41--0.89) |
| Samoa | 166.51 (112.13-238) | 188.02 (116.15-279.34) | 0.13 (0.04-0.17) | 26.63 (17.93-38.07) | 28.57 (17.65-42.45) | -0.28 (-0.71-0.16) |
| San Marino | 3420.66 (2210.34-5035.51) | 6608.2 (4120.88-10018.51) | 0.93 (0.86-0.99) | 256.66 (165.85-377.82) | 176.65 (110.16-267.82) | -1.73 (-1.95--1.51) |
| São Tomé and Príncipe | 10.61 (7.77-14.13) | 15.57 (11.17-20.75) | 0.47 (0.44-0.47) | 178.15 (130.38-237.25) | 123.76 (88.82-165) | -1.31 (-1.45--1.17) |
| Saudi Arabia | 3360.11 (2276.79-4604.25) | 6721.97 (5313.71-8658.89) | 1 (1.33-0.88) | 128.15 (86.84-175.61) | 177.79 (140.55-229.02) | 1.84 (1.64-2.04) |
| Senegal | 290.27 (166.31-467.7) | 598.44 (336.32-949.2) | 1.06 (1.02-1.03) | 164.25 (94.11-264.66) | 135.82 (76.33-215.43) | -0.52 (-0.6--0.44) |
| Serbia | 7812.76 (4719.27-11826.85) | 13883.03 (8695.56-21079.99) | 0.78 (0.84-0.78) | 243.16 (146.88-368.1) | 202.8 (127.02-307.94) | -0.69 (-0.77--0.61) |
| Seychelles | 1693.6 (991.79-2700.15) | 2476.63 (1439.93-4073.14) | 0.46 (0.45-0.51) | 200.45 (117.38-319.58) | 134.11 (77.97-220.57) | -1.6 (-1.76--1.43) |
| Sierra Leone | 1341.85 (794.79-2139.67) | 2014.23 (1159.6-3245.85) | 0.5 (0.46-0.52) | 213.34 (126.36-340.18) | 172.28 (99.19-277.63) | -0.93 (-1.05--0.81) |
| Singapore | 64.31 (46.79-90.01) | 181.26 (133.65-239.6) | 1.82 (1.86-1.66) | 52.87 (38.47-74) | 53.57 (39.5-70.82) | -0.25 (-0.4--0.1) |
| Slovakia | 864.06 (501.84-1394.93) | 2570.37 (1530.05-4169.62) | 1.97 (2.05-1.99) | 196.6 (114.18-317.39) | 184.62 (109.9-299.5) | 0.04 (-0.07-0.15) |
| Slovenia | 2154.79 (1275.45-3411.98) | 2869.58 (1660.78-4661.66) | 0.33 (0.3-0.37) | 220.51 (130.52-349.16) | 160.39 (92.83-260.55) | -1.2 (-1.35--1.06) |
| Solomon Islands | 767.14 (447.81-1228.67) | 1408.56 (806.65-2188.68) | 0.84 (0.8-0.78) | 145.17 (84.74-232.51) | 109.09 (62.47-169.51) | -0.62 (-0.81--0.43) |
| Somalia | 1837.61 (1264.1-2574.91) | 2646.09 (1680.8-3868.85) | 0.44 (0.33-0.5) | 87.87 (60.44-123.12) | 83.38 (52.96-121.91) | 0.24 (-0.34-0.82) |
| South Africa | 859.96 (512.9-1356.51) | 2369.84 (1379.4-3849.9) | 1.76 (1.69-1.84) | 238.83 (142.44-376.73) | 251.29 (146.27-408.23) | 0.04 (-0.1-0.18) |
| South Korea | 63.08 (47.45-84.89) | 145.43 (107.46-189.35) | 1.31 (1.26-1.23) | 43.46 (32.69-58.49) | 33.8 (24.98-44.01) | -0.65 (-0.74--0.57) |
| South Sudan | 1073.31 (628.87-1702.41) | 2210 (1303.5-3528.36) | 1.06 (1.07-1.07) | 229.37 (134.39-363.8) | 208.99 (123.27-333.66) | -0.59 (-0.74--0.45) |
| Spain | 82.82 (50.28-117.33) | 321.61 (227.13-444.48) | 2.88 (3.52-2.79) | 59.29 (35.99-84) | 92.02 (64.99-127.17) | 0.93 (0.66-1.19) |
| Sri Lanka | 13.93 (10.33-18.55) | 30.61 (22.8-41.19) | 1.2 (1.21-1.22) | 139.78 (103.66-186.16) | 106.75 (79.52-143.64) | -1.02 (-1.09--0.96) |
| Sudan | 4229.87 (2493.44-6737.8) | 7077.1 (4157.09-11151.77) | 0.67 (0.67-0.66) | 234.45 (138.2-373.46) | 171.68 (100.85-270.53) | -0.82 (-0.99--0.65) |
| Suriname | 5.51 (4.09-7.16) | 10.05 (7.41-13.61) | 0.82 (0.81-0.9) | 57.42 (42.63-74.68) | 48.47 (35.73-65.59) | -0.5 (-0.74--0.26) |
| Sweden | 1403.71 (940.62-1969.72) | 6350.88 (4474.5-8697.31) | 3.52 (3.76-3.42) | 107.59 (72.1-150.98) | 329.88 (232.41-451.76) | 4.17 (3.84-4.5) |
| Switzerland | 8.71 (4.68-12.75) | 22.74 (13.45-33.43) | 1.61 (1.87-1.62) | 158.05 (85-231.46) | 167.52 (99.05-246.25) | 0.59 (0.44-0.73) |
| Syria | 8.35 (6.14-11.21) | 33.25 (24.36-44.54) | 2.98 (2.97-2.97) | 64.79 (47.64-86.91) | 88.23 (64.64-118.18) | 0.92 (0.68-1.16) |
| Taiwan (province of China) | 8.21 (6.03-10.76) | 17.88 (12.98-23.72) | 1.18 (1.15-1.2) | 74.1 (54.39-97.09) | 114.26 (82.99-151.62) | 1.42 (1.31-1.53) |
| Tajikistan | 2564.46 (1519.66-4040.48) | 3972.87 (2247.89-6227.24) | 0.55 (0.48-0.54) | 245.19 (145.29-386.31) | 167.53 (94.79-262.59) | -1.26 (-1.31--1.22) |
| Tanzania | 4.35 (3.25-5.82) | 14.65 (10.62-19.47) | 2.37 (2.27-2.35) | 31.13 (23.28-41.67) | 45.5 (33-60.48) | 1.14 (0.96-1.32) |
| Thailand | 28643.12 (21868.3-37686.37) | 63705.23 (50164.39-81706.98) | 1.22 (1.29-1.17) | 205.82 (157.14-270.8) | 288.13 (226.88-369.55) | 0.81 (0.67-0.96) |
| The Bahamas | 11290.5 (8556.95-14988.33) | 24106.2 (18183.18-32226.12) | 1.14 (1.12-1.15) | 161.94 (122.73-214.98) | 138 (104.09-184.48) | -0.66 (-1.01--0.31) |
| The Gambia | 214.83 (125.33-352.19) | 457.36 (274.99-719.19) | 1.13 (1.19-1.04) | 231.08 (134.81-378.83) | 188.66 (113.43-296.67) | -0.69 (-0.76--0.63) |
| Timor-Leste | 19.84 (14.79-25.9) | 27.42 (20.44-36.65) | 0.38 (0.38-0.42) | 209.04 (155.84-272.95) | 204.2 (152.19-272.87) | 0.02 (-0.06-0.11) |
| Togo | 0.66 (0.47-0.9) | 0.66 (0.47-0.88) | 0 (0--0.02) | 90.92 (65.01-124.05) | 71.14 (51.21-95.66) | -1.03 (-1.13--0.94) |
| Tokelau | 0.66 (0.5-0.88) | 0.4 (0.3-0.54) | -0.39 (-0.4--0.39) | 182.22 (136.21-241.53) | 103.13 (76.77-137.56) | -1.59 (-1.7--1.48) |
| Tonga | 1124.41 (803.6-1509.54) | 2214.45 (1493.46-2971.49) | 0.97 (0.86-0.97) | 42.05 (30.06-56.46) | 60.25 (40.63-80.85) | 0.9 (0.66-1.15) |
| Trinidad and Tobago | 1468.26 (885.28-2310.25) | 1953.21 (1123.05-3050.15) | 0.33 (0.27-0.32) | 221.51 (133.56-348.54) | 176.4 (101.43-275.47) | -0.7 (-0.76--0.65) |
| Tunisia | 701.14 (435.56-1093.39) | 1181.14 (670.17-1894.89) | 0.68 (0.54-0.73) | 219.16 (136.15-341.77) | 142.81 (81.03-229.11) | -1.22 (-1.28--1.17) |
| Türkiye | 1427 (825.79-2282.79) | 2491.75 (1464.57-4027.54) | 0.75 (0.77-0.76) | 197.31 (114.18-315.63) | 165.74 (97.42-267.89) | -0.62 (-0.66--0.58) |
| Turkmenistan | 4.12 (3.02-5.38) | 11.6 (8.56-15.53) | 1.82 (1.83-1.89) | 168.78 (123.57-220.54) | 119.35 (88.11-159.8) | -2.04 (-2.36--1.71) |
| Tuvalu | 2.73 (2-3.63) | 5.22 (3.88-7.02) | 0.91 (0.94-0.93) | 170.78 (125.13-226.85) | 124.68 (92.68-167.61) | -1.4 (-1.54--1.25) |
| Uganda | 1277.39 (728.78-2028.83) | 2510.89 (1444.77-4053.49) | 0.97 (0.98-1) | 172.52 (98.42-274) | 123.36 (70.98-199.14) | -1.11 (-1.17--1.05) |
| UK | 0.32 (0.24-0.43) | 0.34 (0.25-0.45) | 0.06 (0.04-0.05) | 135.67 (101.47-182.48) | 133.93 (99.34-178.29) | -0.11 (-0.27-0.06) |
| Ukraine | 2.7 (1.93-3.72) | 5.36 (3.95-7.13) | 0.99 (1.05-0.92) | 42.25 (30.24-58.11) | 41.63 (30.64-55.37) | -0.59 (-1.08--0.09) |
| United Arab Emirates | 727.1 (528.92-976.07) | 2237.4 (1595.79-3065.62) | 2.08 (2.02-2.14) | 119.83 (87.17-160.86) | 191.66 (136.7-262.61) | 1.6 (1.38-1.83) |
| Uruguay | 327.57 (221.28-449.21) | 410.36 (281.17-577.62) | 0.25 (0.27-0.29) | 69.95 (47.25-95.92) | 39.95 (27.37-56.24) | -1.64 (-2--1.27) |
| United States | 913 (525.6-1480.8) | 972.69 (581.29-1520.14) | 0.07 (0.11-0.03) | 220.16 (126.74-357.08) | 156.2 (93.35-244.11) | -1.24 (-1.38--1.1) |
| Uzbekistan | 78.67 (45.55-123.3) | 87.32 (50.78-137.08) | 0.11 (0.11-0.11) | 199.31 (115.41-312.39) | 113.93 (66.25-178.84) | -1.71 (-1.84--1.59) |
| Vanuatu | 1150.22 (687.7-1782.42) | 1558.3 (923.5-2498.65) | 0.35 (0.34-0.4) | 248.21 (148.4-384.64) | 167.83 (99.46-269.1) | -1.24 (-1.29--1.2) |
| Venezuela | 3337.73 (2354.38-4739.6) | 14537.4 (10525.06-19538.66) | 3.36 (3.47-3.12) | 120.34 (84.88-170.88) | 193.06 (139.78-259.48) | 0.84 (0.38-1.3) |
| Viet Nam | 16.77 (9.63-26.61) | 17.28 (9.89-27.15) | 0.03 (0.03-0.02) | 149.73 (86.04-237.62) | 94.51 (54.1-148.49) | -1.5 (-1.65--1.35) |
| Virgin Islands | 744.41 (548.9-971.92) | 1288.11 (979.46-1675.12) | 0.73 (0.78-0.72) | 50.06 (36.91-65.36) | 41.37 (31.45-53.8) | -0.42 (-0.63--0.21) |
| Yemen | 875.3 (499-1420.71) | 2078.83 (1199.63-3400.83) | 1.37 (1.4-1.39) | 136.57 (77.86-221.66) | 115.93 (66.9-189.65) | -0.34 (-0.41--0.28) |
| Zambia | 110.63 (64.39-180.25) | 221.52 (129.09-359.72) | 1 (1-1) | 199.77 (116.27-325.5) | 143.25 (83.48-232.62) | -0.83 (-0.9--0.75) |
| Zimbabwe | 1333.37 (789.12-2155.53) | 2612.46 (1497.14-4143.03) | 0.96 (0.9-0.92) | 130.57 (77.27-211.08) | 95.79 (54.9-151.92) | -0.98 (-1.04--0.93) |

**Table S11. YLDs, YLD rates, and EAPC trends of HF impairment under the COPD cause category across 204 countries and territories from 1990 to 2021 among adults aged ≥55 years.**

| **Location** | **YLDs** | |  | **YLD rates** | |  |
| --- | --- | --- | --- | --- | --- | --- |
|  | **1990 (95% UI)** | **2021 (95% UI)** | **Relative change, 1990–2021(95% UI)** | **1990, per 100,000 (95% UI)** | **2021, per 100,000 (95% UI)** | **EAPC (95% CI)** |
| Afghanistan | 44.47 (24.12-68.06) | 30.84 (16.1-49.23) | -0.31 (-0.33--0.28) | 5.61 (3.04-8.59) | 3.19 (1.66-5.09) | -1.97 (-2.09--1.86) |
| Albania | 849.15 (491.23-1275.25) | 3830.61 (2351.54-5522.03) | 3.51 (3.79-3.33) | 15.55 (9-23.36) | 31.22 (19.16-45) | 2.12 (1.75-2.49) |
| Algeria | 1319.45 (767.71-1973.92) | 3332.03 (2155.66-4701.65) | 1.53 (1.81-1.38) | 13.8 (8.03-20.65) | 21.53 (13.93-30.38) | 1.37 (1.21-1.53) |
| American Samoa | 358.99 (212.15-577.92) | 913.57 (530.69-1485.05) | 1.54 (1.5-1.57) | 7.52 (4.44-12.11) | 6.55 (3.81-10.65) | -0.68 (-0.77--0.58) |
| Andorra | 102.59 (57.4-157.11) | 385.81 (223.77-590.81) | 2.76 (2.9-2.76) | 6.1 (3.41-9.34) | 8.62 (5-13.19) | 1.35 (1.2-1.5) |
| Angola | 0.21 (0.12-0.32) | 0.41 (0.23-0.66) | 0.95 (0.92-1.06) | 2.41 (1.44-3.67) | 2.2 (1.23-3.49) | -0.53 (-0.74--0.32) |
| Antigua and Barbuda | 57.77 (34.46-87.8) | 174.16 (104.29-269.36) | 2.01 (2.03-2.07) | 7.8 (4.65-11.85) | 8.01 (4.8-12.39) | 0.14 (-0.03-0.3) |
| Argentina | 240.12 (145.19-355.46) | 788.94 (471.68-1182.88) | 2.29 (2.25-2.33) | 13.49 (8.16-19.97) | 16.38 (9.79-24.56) | 0.53 (0.43-0.62) |
| Armenia | 0.52 (0.32-0.8) | 1.52 (0.86-2.34) | 1.92 (1.69-1.92) | 2.11 (1.29-3.25) | 2.11 (1.19-3.25) | 0.25 (0.17-0.32) |
| Australia | 1550.85 (937.69-2437.9) | 3555.81 (2030.46-5785.53) | 1.29 (1.17-1.37) | 9.6 (5.8-15.09) | 8.49 (4.85-13.81) | -0.47 (-0.62--0.33) |
| Austria | 115.85 (65.5-179.26) | 431.92 (256.34-650.14) | 2.73 (2.91-2.63) | 6.84 (3.86-10.58) | 12.64 (7.5-19.03) | 2.2 (1.93-2.46) |
| Azerbaijan | 1.87 (1.06-2.88) | 4.21 (2.46-6.48) | 1.25 (1.32-1.25) | 3.99 (2.26-6.15) | 4.62 (2.7-7.11) | 0.2 (-0.06-0.47) |
| Bahrain | 0.74 (0.29-1.25) | 1.76 (0.68-3.01) | 1.38 (1.34-1.41) | 4.73 (1.84-7.98) | 2.95 (1.13-5.03) | -1.85 (-2.03--1.68) |
| Bangladesh | 1403.34 (750.48-2251.64) | 3181.93 (1839.42-5065.31) | 1.27 (1.45-1.25) | 9.45 (5.05-15.16) | 15.13 (8.75-24.09) | 1.59 (1.46-1.73) |
| Barbados | 731.16 (432.82-1088.45) | 1640.57 (952.87-2417.68) | 1.24 (1.2-1.22) | 27.47 (16.26-40.9) | 29.11 (16.91-42.9) | 0.61 (0.41-0.8) |
| Belarus | 0.41 (0.23-0.64) | 0.59 (0.35-0.91) | 0.44 (0.52-0.42) | 4.19 (2.31-6.57) | 3.92 (2.3-6.06) | -0.12 (-0.27-0.04) |
| Belgium | 965.7 (524.55-1572.16) | 541.05 (267.2-924.32) | -0.44 (-0.49--0.41) | 7.75 (4.21-12.61) | 3.98 (1.97-6.81) | -3.81 (-4.23--3.38) |
| Belize | 30.19 (17.59-45.76) | 66.66 (38.46-102.94) | 1.21 (1.19-1.25) | 8.8 (5.13-13.34) | 8.62 (4.97-13.31) | -0.17 (-0.34-0) |
| Benin | 1.06 (0.65-1.55) | 3.82 (2.22-5.69) | 2.6 (2.42-2.67) | 6.87 (4.23-10.08) | 7.63 (4.42-11.37) | 0.56 (0.29-0.84) |
| Bermuda | 168.35 (101.39-248.35) | 488.45 (287.5-728.56) | 1.9 (1.84-1.93) | 11.41 (6.87-16.83) | 9.96 (5.86-14.86) | -0.93 (-1.14--0.71) |
| Bhutan | 1001.33 (523.24-1696.88) | 3546.69 (1907.34-5932.9) | 2.54 (2.65-2.5) | 3.38 (1.77-5.73) | 6.79 (3.65-11.37) | 2.12 (2-2.24) |
| Bolivia | 90.28 (46.18-146.31) | 285.08 (160.1-442.54) | 2.16 (2.47-2.02) | 5.34 (2.73-8.66) | 9.73 (5.46-15.1) | 3.06 (2.48-3.64) |
| Bosnia and Herzegovina | 2.06 (1.24-3.16) | 7.92 (4.82-11.45) | 2.84 (2.89-2.62) | 13.46 (8.11-20.64) | 14.55 (8.86-21.05) | 0.64 (0.31-0.98) |
| Botswana | 29.54 (18.05-44.31) | 87.55 (51.07-134) | 1.96 (1.83-2.02) | 4.83 (2.95-7.25) | 5.24 (3.06-8.02) | 0.15 (-0.01-0.31) |
| Brazil | 17.95 (8.92-29.3) | 22.44 (8.57-40.13) | 0.25 (-0.04-0.37) | 1.62 (0.8-2.64) | 2.14 (0.82-3.82) | 1.02 (0.8-1.25) |
| Brunei | 632.2 (392.18-959.18) | 1574.02 (918.7-2333.95) | 1.49 (1.34-1.43) | 16.15 (10.02-24.5) | 18.64 (10.88-27.65) | 0.47 (0.28-0.67) |
| Bulgaria | 0.52 (0.3-0.79) | 0.69 (0.4-1.09) | 0.33 (0.33-0.38) | 4.35 (2.51-6.59) | 3.43 (1.97-5.44) | -1.06 (-1.31--0.8) |
| Burkina Faso | 1557.92 (934.56-2299.81) | 3759.91 (2228.02-5540.78) | 1.41 (1.38-1.41) | 26.25 (15.75-38.75) | 19.34 (11.46-28.5) | -1.12 (-1.25--0.99) |
| Burundi | 28.28 (15.87-42.86) | 66.74 (38.04-97.02) | 1.36 (1.4-1.26) | 5.24 (2.94-7.95) | 5.63 (3.21-8.19) | 0.27 (0.05-0.48) |
| Cabo Verde | 1.08 (0.62-1.65) | 2.34 (1.34-3.65) | 1.17 (1.16-1.21) | 1.75 (1.02-2.68) | 2.08 (1.19-3.24) | 0.54 (0.29-0.8) |
| Cambodia | 235.08 (117.59-366.27) | 1462.47 (892.32-2167.9) | 5.22 (6.59-4.92) | 4.72 (2.36-7.36) | 8.74 (5.34-12.96) | 2.44 (2.03-2.85) |
| Cameroon | 1.02 (0.57-1.58) | 3.52 (2.07-5.29) | 2.45 (2.63-2.35) | 6.95 (3.87-10.83) | 8.35 (4.92-12.58) | 0.67 (0.29-1.06) |
| Canada | 0.31 (0.18-0.48) | 0.41 (0.24-0.6) | 0.32 (0.33-0.25) | 12.21 (7.14-18.87) | 6.97 (4.16-10.32) | -2.24 (-2.52--1.96) |
| Central African Republic | 3.89 (2.38-5.76) | 14.74 (8.83-21.94) | 2.79 (2.71-2.81) | 8.95 (5.47-13.26) | 10.38 (6.22-15.45) | 0.25 (0.1-0.41) |
| Chad | 17.1 (9.65-26.09) | 37.54 (21.97-57.77) | 1.2 (1.28-1.21) | 5.81 (3.28-8.86) | 7.1 (4.15-10.92) | 0.32 (-0.38-1.03) |
| Chile | 0.27 (0.15-0.44) | 0.74 (0.41-1.14) | 1.74 (1.73-1.59) | 2.29 (1.26-3.69) | 2.89 (1.61-4.48) | 0.85 (0.49-1.22) |
| China | 276.53 (160.95-449.77) | 840.05 (459.6-1403.32) | 2.04 (1.86-2.12) | 11.65 (6.78-18.94) | 24.95 (13.65-41.69) | 2.64 (2.45-2.84) |
| Colombia | 2.34 (1.39-3.5) | 6.66 (3.82-10.09) | 1.85 (1.75-1.88) | 5.39 (3.19-8.04) | 5.96 (3.42-9.03) | 0.46 (0.22-0.71) |
| Comoros | 0.41 (0.23-0.62) | 1.12 (0.66-1.69) | 1.73 (1.87-1.73) | 6.93 (3.95-10.52) | 9.5 (5.55-14.25) | 1.34 (1.12-1.56) |
| Congo (Brazzaville) | 71.92 (35.1-122.75) | 136.45 (67.46-234.58) | 0.9 (0.92-0.91) | 15.62 (7.62-26.67) | 12.63 (6.25-21.72) | -0.65 (-0.77--0.54) |
| Cook Islands | 81.24 (48.39-119.44) | 209.27 (126.98-304.14) | 1.58 (1.62-1.55) | 27.46 (16.36-40.37) | 25.48 (15.46-37.03) | -0.46 (-0.6--0.32) |
| Costa Rica | 4.57 (2.61-7.19) | 16 (9.15-24.59) | 2.5 (2.51-2.42) | 3.33 (1.9-5.24) | 4.59 (2.63-7.06) | 1.06 (1.01-1.12) |
| Côte d'Ivoire | 303.69 (182.72-464.77) | 1815.37 (1107.44-2726.97) | 4.98 (5.06-4.87) | 10.55 (6.35-16.14) | 18.98 (11.58-28.51) | 2.03 (1.8-2.26) |
| Croatia | 41.24 (25.06-59.91) | 127.52 (75.96-188.64) | 2.09 (2.03-2.15) | 7.87 (4.78-11.43) | 8.25 (4.92-12.21) | 0.45 (0.32-0.58) |
| Cuba | 133.9 (82.28-195.88) | 513.25 (318.1-766.04) | 2.83 (2.87-2.91) | 6.79 (4.17-9.93) | 9.18 (5.69-13.7) | 1.55 (1.24-1.86) |
| Cyprus | 66.43 (39.87-96.93) | 199.39 (119.65-302.4) | 2 (2-2.12) | 7.73 (4.64-11.27) | 7.2 (4.32-10.93) | -0.19 (-0.26--0.13) |
| Czechia | 2.05 (1.22-3.13) | 4.6 (2.74-6.78) | 1.24 (1.25-1.17) | 9.08 (5.41-13.85) | 8.47 (5.05-12.5) | -0.01 (-0.13-0.12) |
| Denmark | 16.57 (7.93-27.26) | 53.92 (31.69-81.17) | 2.25 (3-1.98) | 4.57 (2.19-7.52) | 3.55 (2.09-5.34) | -1.09 (-1.21--0.96) |
| Djibouti | 29.39 (18.11-44.76) | 135.36 (82.95-200.31) | 3.61 (3.58-3.48) | 10.34 (6.37-15.74) | 14.14 (8.66-20.92) | 0.86 (0.68-1.03) |
| Dominica | 1.07 (0.63-1.66) | 0.91 (0.52-1.35) | -0.15 (-0.17--0.19) | 13.45 (7.94-20.81) | 6.88 (3.98-10.25) | -2.69 (-2.85--2.53) |
| Dominican Republic | 9.4 (4.55-15.52) | 13.1 (6.63-21.79) | 0.39 (0.46-0.4) | 5.42 (2.63-8.95) | 3.32 (1.68-5.52) | -1.74 (-1.83--1.65) |
| DR Congo | 4.02 (2.33-6.19) | 6.81 (3.86-10.6) | 0.69 (0.66-0.71) | 7.13 (4.13-10.97) | 4.98 (2.83-7.75) | -1.39 (-1.55--1.24) |
| Ecuador | 58.97 (37.31-84.4) | 121.66 (73.86-181.55) | 1.06 (0.98-1.15) | 12.06 (7.63-17.26) | 11.9 (7.22-17.75) | 0.25 (0.13-0.36) |
| Egypt | 58376.5 (36520.92-83834.76) | 129309.01 (76541.57-195850.58) | 1.22 (1.1-1.34) | 40.68 (25.45-58.41) | 34.12 (20.2-51.68) | -1.3 (-1.56--1.04) |
| El Salvador | 43.55 (26.17-65.84) | 166.1 (97.55-253.99) | 2.81 (2.73-2.86) | 7.57 (4.55-11.45) | 9.07 (5.33-13.87) | 0.73 (0.56-0.9) |
| Equatorial Guinea | 1007.01 (615.27-1450.07) | 2976.32 (1758.47-4642.35) | 1.96 (1.86-2.2) | 14.48 (8.85-20.86) | 13.81 (8.16-21.54) | -0.34 (-0.46--0.22) |
| Eritrea | 1197.23 (664.51-2004.04) | 1647.59 (888.51-2755.51) | 0.38 (0.34-0.37) | 16.43 (9.12-27.51) | 10.98 (5.92-18.37) | -1.44 (-1.51--1.37) |
| Estonia | 19 (11.51-27.95) | 76.1 (44.11-116.98) | 3.01 (2.83-3.19) | 7.65 (4.63-11.25) | 9.35 (5.42-14.37) | 0.88 (0.66-1.1) |
| Eswatini | 101.83 (54.24-165.22) | 244.05 (133.88-388.72) | 1.4 (1.47-1.35) | 15.48 (8.24-25.11) | 17.11 (9.39-27.25) | 0.42 (0.32-0.52) |
| Ethiopia | 34.48 (19.02-53.69) | 35.58 (20.42-54.29) | 0.03 (0.07-0.01) | 7.08 (3.91-11.03) | 4.52 (2.6-6.9) | -0.52 (-0.99--0.05) |
| Federated States of Micronesia | 74.43 (40.12-120.47) | 41.37 (22.42-64.69) | -0.44 (-0.44--0.46) | 3.29 (1.77-5.32) | 1.74 (0.94-2.72) | -2.1 (-2.39--1.81) |
| Fiji | 37.69 (21.96-57.43) | 107.5 (64.32-160.31) | 1.85 (1.93-1.79) | 11.31 (6.59-17.23) | 10.22 (6.12-15.24) | -0.28 (-0.36--0.21) |
| Finland | 24.34 (14.74-36.39) | 96.24 (58.68-139.64) | 2.95 (2.98-2.84) | 10.05 (6.08-15.02) | 12.94 (7.89-18.78) | 0.48 (0.19-0.78) |
| France | 30.22 (15.99-46.38) | 76.77 (37.76-123.95) | 1.54 (1.36-1.67) | 2.71 (1.44-4.17) | 5.15 (2.53-8.31) | 1.43 (1.06-1.8) |
| Gabon | 91.88 (51.59-137.56) | 39.99 (22.04-61.14) | -0.56 (-0.57--0.56) | 17.84 (10.02-26.72) | 4.73 (2.61-7.23) | -4.65 (-5.2--4.09) |
| Georgia | 27.56 (15.05-41.94) | 38.6 (20.71-60.92) | 0.4 (0.38-0.45) | 7.94 (4.34-12.09) | 4.91 (2.63-7.75) | -1.73 (-1.86--1.6) |
| Germany | 30.47 (16.38-48.09) | 37.14 (20.44-58.97) | 0.22 (0.25-0.23) | 3.49 (1.88-5.51) | 1.94 (1.07-3.08) | -2.08 (-2.32--1.84) |
| Ghana | 67.89 (40.56-102.86) | 351.22 (207.9-523.06) | 4.17 (4.13-4.09) | 4.34 (2.59-6.57) | 6.71 (3.97-10) | 1.43 (1.09-1.77) |
| Greece | 26.16 (15.21-40.19) | 57.28 (32.5-93.89) | 1.19 (1.14-1.34) | 2.52 (1.47-3.87) | 3.49 (1.98-5.72) | 1.51 (1.3-1.72) |
| Greenland | 303.23 (144.57-491.49) | 215.85 (107.43-355.35) | -0.29 (-0.26--0.28) | 6.06 (2.89-9.82) | 3.59 (1.79-5.92) | -1.35 (-1.57--1.13) |
| Grenada | 41.15 (23.03-63.59) | 52.62 (28.32-82.86) | 0.28 (0.23-0.3) | 5.61 (3.14-8.67) | 4.8 (2.59-7.57) | -0.31 (-0.51--0.11) |
| Guam | 63.39 (31.35-102.41) | 107.95 (52.05-169.89) | 0.7 (0.66-0.66) | 3.05 (1.51-4.92) | 3.85 (1.86-6.06) | -0.28 (-0.74-0.18) |
| Guatemala | 1623.54 (977.42-2464.96) | 6133.26 (3627.78-9627.4) | 2.78 (2.71-2.91) | 10.99 (6.61-16.68) | 14.16 (8.38-22.23) | 0.71 (0.61-0.81) |
| Guinea | 63.09 (36.54-96.46) | 237.37 (146.4-358.04) | 2.76 (3.01-2.71) | 3.01 (1.74-4.6) | 3.91 (2.41-5.9) | 1.04 (0.91-1.17) |
| Guinea-Bissau | 23.05 (13.76-34.69) | 72.29 (41.18-110.27) | 2.14 (1.99-2.18) | 6.35 (3.79-9.56) | 7.3 (4.16-11.14) | 0.51 (0.35-0.67) |
| Guyana | 1.69 (1-2.54) | 9.45 (5.43-13.71) | 4.59 (4.43-4.4) | 6.03 (3.58-9.05) | 5.82 (3.34-8.44) | -0.5 (-0.76--0.24) |
| Haiti | 175.78 (101.55-264.07) | 251.16 (144.1-387.35) | 0.43 (0.42-0.47) | 3.93 (2.27-5.91) | 2.27 (1.3-3.5) | -2.07 (-2.23--1.92) |
| Honduras | 8.54 (5.07-12.63) | 38.8 (22.19-59.24) | 3.54 (3.38-3.69) | 3.97 (2.36-5.88) | 3.09 (1.77-4.71) | -0.61 (-0.81--0.42) |
| Hungary | 21.5 (10.17-35.39) | 53.93 (28.69-84.87) | 1.51 (1.82-1.4) | 4.99 (2.36-8.22) | 7.37 (3.92-11.59) | 1.14 (0.98-1.3) |
| Iceland | 167.75 (99.78-262.64) | 725.62 (433.65-1163.96) | 3.33 (3.35-3.43) | 3.64 (2.16-5.7) | 5.59 (3.34-8.96) | 1.68 (1.48-1.87) |
| India | 1.17 (0.68-1.83) | 5.15 (3.15-7.65) | 3.4 (3.63-3.18) | 1.28 (0.75-2) | 1.11 (0.68-1.64) | -0.06 (-0.67-0.56) |
| Indonesia | 24.28 (14.37-36.72) | 125.91 (74.42-183.9) | 4.19 (4.18-4.01) | 6.4 (3.79-9.69) | 12.85 (7.59-18.76) | 3.06 (2.73-3.38) |
| Iran | 597.5 (345.34-935.11) | 1101.38 (630.74-1823.79) | 0.84 (0.83-0.95) | 7.75 (4.48-12.13) | 9.09 (5.21-15.05) | 0.58 (0.3-0.85) |
| Iraq | 31.53 (18.39-47.3) | 75.45 (44.46-113.61) | 1.39 (1.42-1.4) | 2.5 (1.46-3.75) | 1.94 (1.14-2.92) | -0.76 (-0.86--0.67) |
| Ireland | 1.65 (0.92-2.52) | 3.66 (1.79-6.3) | 1.22 (0.95-1.5) | 1.51 (0.85-2.32) | 2.1 (1.03-3.61) | 0.04 (-0.22-0.31) |
| Israel | 61.73 (31.17-105.18) | 82.18 (40.23-141.99) | 0.33 (0.29-0.35) | 18.63 (9.4-31.73) | 13.6 (6.66-23.5) | -1.04 (-1.12--0.97) |
| Italy | 17.27 (10.75-25.25) | 35.3 (20.79-53.28) | 1.04 (0.93-1.11) | 5.55 (3.46-8.12) | 4.22 (2.49-6.37) | -0.49 (-0.66--0.31) |
| Jamaica | 6.18 (3.38-9.93) | 8.68 (4.14-15.67) | 0.4 (0.22-0.58) | 1.72 (0.94-2.77) | 1.98 (0.95-3.58) | -0.32 (-0.58--0.07) |
| Japan | 1647.74 (914.18-2643.22) | 1622.17 (872.18-2703.06) | -0.02 (-0.05-0.02) | 5.22 (2.9-8.37) | 3.82 (2.05-6.36) | -1.44 (-1.75--1.13) |
| Jordan | 126.92 (66.03-204.89) | 247.1 (126.46-396.44) | 0.95 (0.92-0.93) | 4.93 (2.56-7.95) | 7.77 (3.98-12.47) | 1.61 (1.41-1.82) |
| Kazakhstan | 77.37 (46.06-120.58) | 207.79 (120.17-312.74) | 1.69 (1.61-1.59) | 3.3 (1.96-5.14) | 3.46 (2-5.21) | 0.34 (0.24-0.44) |
| Kenya | 3.02 (1.81-4.67) | 6.63 (3.97-10.09) | 1.2 (1.19-1.16) | 3.01 (1.8-4.65) | 2.12 (1.27-3.23) | -0.74 (-0.88--0.59) |
| Kiribati | 43.17 (26.04-65.26) | 87.75 (51.51-130.22) | 1.03 (0.98-1) | 4.85 (2.92-7.33) | 2.91 (1.71-4.33) | -1.48 (-1.6--1.37) |
| Kuwait | 30.13 (15.56-49.83) | 70.1 (34.87-118.61) | 1.33 (1.24-1.38) | 15.44 (7.98-25.54) | 11.22 (5.58-18.99) | -0.92 (-1--0.85) |
| Kyrgyzstan | 41.47 (20.42-69.14) | 165.8 (90.03-258.6) | 3 (3.41-2.74) | 3.49 (1.72-5.82) | 8.24 (4.47-12.85) | 2.74 (2.28-3.2) |
| Laos | 1.84 (0.92-2.89) | 7.64 (4.31-11.95) | 3.15 (3.68-3.13) | 3.86 (1.93-6.06) | 7.84 (4.43-12.27) | 1.8 (1.48-2.12) |
| Latvia | 30.82 (19.14-46.49) | 85.2 (48.52-130.89) | 1.76 (1.54-1.82) | 3.5 (2.17-5.27) | 3.61 (2.05-5.54) | 0.26 (0.16-0.37) |
| Lebanon | 80.76 (45.13-125.36) | 259.95 (151.23-392.71) | 2.22 (2.35-2.13) | 11.86 (6.63-18.42) | 19.81 (11.52-29.92) | 1.76 (1.67-1.84) |
| Lesotho | 1.41 (0.81-2.18) | 4.35 (2.68-6.39) | 2.09 (2.31-1.93) | 14.27 (8.21-22.08) | 16.45 (10.12-24.15) | 0.37 (0.17-0.57) |
| Liberia | 30.58 (17.5-46.89) | 99.87 (58.18-148.36) | 2.27 (2.32-2.16) | 3.51 (2.01-5.39) | 4.27 (2.49-6.35) | 0.65 (0.44-0.85) |
| Libya | 0.5 (0.29-0.74) | 0.91 (0.54-1.41) | 0.82 (0.86-0.91) | 13.67 (7.91-20.15) | 10.76 (6.43-16.68) | -0.96 (-1.07--0.85) |
| Lithuania | 0.45 (0.28-0.66) | 4.61 (2.77-6.97) | 9.24 (8.89-9.56) | 2.75 (1.71-4.05) | 3.01 (1.81-4.55) | 0.51 (0.36-0.67) |
| Luxembourg | 97.34 (52.17-152.78) | 405.42 (216.73-632.72) | 3.16 (3.15-3.14) | 4.11 (2.2-6.45) | 11.61 (6.2-18.11) | 4.55 (4.21-4.89) |
| Madagascar | 695.71 (418.67-1025.4) | 2459.57 (1435.64-3557.16) | 2.54 (2.43-2.47) | 11.64 (7.01-17.16) | 14.89 (8.69-21.53) | 1.39 (1.18-1.6) |
| Malawi | 30.78 (18.88-46.33) | 86.29 (50.56-130.72) | 1.8 (1.68-1.82) | 3.84 (2.35-5.78) | 3.84 (2.25-5.81) | 0.27 (0.19-0.36) |
| Malaysia | 39.39 (21.78-60.46) | 245.83 (143.28-379.7) | 5.24 (5.58-5.28) | 4.86 (2.69-7.46) | 12.3 (7.17-19) | 2.95 (2.61-3.29) |
| Maldives | 3.01 (1.81-4.47) | 26.48 (14.73-43.56) | 7.8 (7.14-8.74) | 5.36 (3.22-7.97) | 3.69 (2.05-6.07) | -1.61 (-1.77--1.46) |
| Mali | 54.59 (32.54-84.29) | 62.25 (36.03-94.14) | 0.14 (0.11-0.12) | 4.49 (2.68-6.94) | 5.07 (2.93-7.66) | 0.81 (0.67-0.94) |
| Malta | 1727.99 (1045.8-2583.93) | 4129.59 (2453.46-6053.68) | 1.39 (1.35-1.34) | 22.81 (13.81-34.11) | 17.67 (10.5-25.91) | -0.79 (-0.85--0.72) |
| Marshall Islands | 23.8 (12.51-41.39) | 42.2 (21.92-69.65) | 0.77 (0.75-0.68) | 21.93 (11.52-38.14) | 18.67 (9.7-30.83) | -0.48 (-0.52--0.45) |
| Mauritania | 49.65 (26.92-78.35) | 24.76 (13.81-38.55) | -0.5 (-0.49--0.51) | 6.43 (3.49-10.15) | 2.34 (1.31-3.65) | -3.39 (-3.79--2.98) |
| Mauritius | 7.73 (4.79-11.53) | 24.62 (15.32-36.43) | 2.18 (2.2-2.16) | 19.48 (12.06-29.06) | 24.72 (15.39-36.58) | 0.8 (0.75-0.85) |
| Mexico | 0.11 (0.07-0.17) | 0.15 (0.08-0.22) | 0.36 (0.14-0.29) | 9.81 (5.79-14.45) | 8.03 (4.6-11.95) | -1.09 (-1.27--0.91) |
| Moldova | 16559.75 (9938.5-25366.05) | 51597.11 (30456.91-80653.26) | 2.12 (2.06-2.18) | 21.6 (12.96-33.09) | 25.66 (15.15-40.12) | 0.61 (0.5-0.72) |
| Monaco | 51.67 (29.02-80.19) | 121.56 (65.48-183.56) | 1.35 (1.26-1.29) | 7.66 (4.3-11.88) | 13.44 (7.24-20.3) | 2.07 (1.84-2.3) |
| Mongolia | 3.78 (2.1-5.79) | 11.28 (6.67-17.83) | 1.98 (2.18-2.08) | 5.22 (2.9-7.99) | 7.15 (4.23-11.3) | 1.61 (1.33-1.89) |
| Montenegro | 1033.61 (510.08-1749.58) | 2910.6 (1645.71-4762.91) | 1.82 (2.23-1.72) | 6.79 (3.35-11.5) | 12.81 (7.24-20.97) | 2.45 (1.88-3.02) |
| Morocco | 544.02 (312.93-819.09) | 2231.38 (1448.76-3077.99) | 3.1 (3.63-2.76) | 16.58 (9.54-24.96) | 30.12 (19.56-41.55) | 1.59 (1.39-1.79) |
| Mozambique | 434.25 (262.42-648.37) | 1163.68 (713.26-1750.73) | 1.68 (1.72-1.7) | 28.21 (17.05-42.12) | 29.4 (18.02-44.23) | 0.19 (0.15-0.23) |
| Myanmar | 8.97 (4.57-15.31) | 13.02 (6.66-22.63) | 0.45 (0.46-0.48) | 19.87 (10.13-33.91) | 14.38 (7.36-24.99) | -1.06 (-1.25--0.86) |
| Namibia | 1628.31 (977.91-2531.7) | 2726.67 (1590.62-4477.06) | 0.67 (0.63-0.77) | 17.86 (10.72-27.76) | 13.75 (8.02-22.58) | -1.02 (-1.09--0.96) |
| Nauru | 92.29 (46.23-165.61) | 176.77 (82.8-311.5) | 0.92 (0.79-0.88) | 14.82 (7.42-26.59) | 9.14 (4.28-16.11) | -1.58 (-1.69--1.48) |
| Nepal | 13.25 (7.15-20.11) | 9.58 (5.29-14.79) | -0.28 (-0.26--0.26) | 4.08 (2.2-6.19) | 1.35 (0.75-2.09) | -3.84 (-4.14--3.54) |
| Netherlands | 1352.53 (765.96-2112.28) | 4595.53 (2609.21-6788.33) | 2.4 (2.41-2.21) | 6.45 (3.65-10.07) | 14.58 (8.28-21.54) | 4.26 (3.74-4.78) |
| New Zealand | 410.12 (244.82-629.66) | 1624.37 (959.12-2473.26) | 2.96 (2.92-2.93) | 12.39 (7.39-19.02) | 27.76 (16.39-42.27) | 2.94 (2.72-3.17) |
| Nicaragua | 28.65 (14.55-49.37) | 44.05 (21.87-77.04) | 0.54 (0.5-0.56) | 15.17 (7.71-26.15) | 12.31 (6.11-21.54) | -0.59 (-0.64--0.55) |
| Niger | 1041.23 (597.31-1685.46) | 1948.35 (1087.98-3146.77) | 0.87 (0.82-0.87) | 30.85 (17.7-49.93) | 24.7 (13.79-39.89) | -0.73 (-1.03--0.42) |
| Nigeria | 78.86 (44.79-120.4) | 48.97 (25.29-77.35) | -0.38 (-0.44--0.36) | 4.03 (2.29-6.16) | 1.05 (0.54-1.66) | -5.33 (-5.72--4.94) |
| Niue | 20.07 (9.75-35.51) | 36.98 (18.31-64.97) | 0.84 (0.88-0.83) | 11.28 (5.48-19.95) | 8.51 (4.21-14.95) | -0.83 (-0.87--0.79) |
| North Korea | 2.12 (1.26-3.2) | 3 (1.85-4.48) | 0.42 (0.47-0.4) | 14.74 (8.72-22.19) | 12.17 (7.52-18.2) | -0.69 (-0.81--0.56) |
| North Macedonia | 77.3 (32.61-133.82) | 355.75 (187.38-590.02) | 3.6 (4.75-3.41) | 7.14 (3.01-12.36) | 21.94 (11.55-36.38) | 4.34 (3.98-4.7) |
| Northern Mariana Islands | 7.1 (3.44-11.2) | 27.95 (15.92-43.09) | 2.94 (3.63-2.85) | 7.6 (3.69-11.99) | 15.73 (8.96-24.25) | 2.32 (1.74-2.9) |
| Norway | 141.08 (89.35-211.8) | 361.25 (213.57-552.78) | 1.56 (1.39-1.61) | 7.25 (4.59-10.88) | 12.22 (7.23-18.71) | 1.22 (1-1.44) |
| Oman | 0.12 (0.07-0.18) | 0.26 (0.15-0.42) | 1.17 (1.14-1.33) | 5.56 (3.17-8.59) | 5.58 (3.27-8.86) | 0.22 (0.13-0.31) |
| Pakistan | 189.7 (110-291.52) | 62.92 (34.15-101.08) | -0.67 (-0.69--0.65) | 8.19 (4.75-12.59) | 2.19 (1.19-3.51) | -5.64 (-6.03--5.24) |
| Palau | 11.55 (5.81-20.16) | 14.58 (7.01-25.06) | 0.26 (0.21-0.24) | 11.8 (5.93-20.59) | 8.16 (3.92-14.02) | -0.98 (-1.05--0.92) |
| Palestine | 233.81 (134.32-361.64) | 640.32 (382.75-943.96) | 1.74 (1.85-1.61) | 9.59 (5.51-14.83) | 16.46 (9.84-24.26) | 2.01 (1.71-2.31) |
| Panama | 379.02 (195.57-658.98) | 749.28 (387.9-1281.09) | 0.98 (0.98-0.94) | 14.35 (7.41-24.95) | 12.41 (6.42-21.21) | -0.43 (-0.74--0.13) |
| Papua New Guinea | 12.63 (6.77-19.5) | 20.39 (10.21-33.13) | 0.61 (0.51-0.7) | 3.84 (2.06-5.93) | 3.44 (1.72-5.59) | -0.2 (-0.27--0.14) |
| Paraguay | 5.63 (2.83-9.51) | 7.24 (3.77-12.24) | 0.29 (0.33-0.29) | 17.63 (8.85-29.8) | 9.16 (4.77-15.48) | -1.98 (-2.23--1.72) |
| Peru | 88.13 (44.67-149.83) | 138.25 (71.84-236.54) | 0.57 (0.61-0.58) | 23.42 (11.87-39.81) | 17.27 (8.97-29.55) | -1.31 (-1.44--1.17) |
| Philippines | 45.2 (24.24-76.35) | 41.08 (21.06-73.76) | -0.09 (-0.13--0.03) | 32.33 (17.34-54.6) | 22.75 (11.66-40.85) | -1.31 (-1.45--1.16) |
| Poland | 5.76 (2.86-10.13) | 11.36 (6.08-19.07) | 0.97 (1.13-0.88) | 17.75 (8.81-31.22) | 14.06 (7.52-23.59) | -0.66 (-0.7--0.63) |
| Portugal | 295.45 (166.38-452.43) | 783.96 (450.53-1214.17) | 1.65 (1.71-1.68) | 5.31 (2.99-8.13) | 8.4 (4.83-13.01) | 1.6 (1.34-1.86) |
| Puerto Rico | 7304.26 (4064.57-11696.59) | 21795.14 (12547.47-37153.33) | 1.98 (2.09-2.18) | 13.92 (7.75-22.3) | 21.74 (12.52-37.06) | 1.4 (1.18-1.62) |
| Qatar | 0.41 (0.23-0.64) | 1.79 (1.01-2.79) | 3.37 (3.39-3.36) | 3.86 (2.22-6.05) | 7.67 (4.33-11.97) | 2.13 (1.89-2.37) |
| Romania | 9.21 (4.67-15.42) | 11.56 (5.83-19.59) | 0.26 (0.25-0.27) | 14.42 (7.31-24.15) | 10.07 (5.08-17.05) | -1.06 (-1.14--0.99) |
| Russia | 105.09 (53.04-177.86) | 137.24 (70.62-231.61) | 0.31 (0.33-0.3) | 18.99 (9.58-32.14) | 15.02 (7.73-25.35) | -0.66 (-0.75--0.57) |
| Rwanda | 2.98 (1.51-5.24) | 10.56 (5.26-18.16) | 2.54 (2.48-2.47) | 13.91 (7.02-24.43) | 10.18 (5.07-17.51) | -1.05 (-1.26--0.85) |
| Saint Kitts and Nevis | 122.55 (64.06-209.62) | 227.02 (115.27-398.53) | 0.85 (0.8-0.9) | 18.33 (9.58-31.35) | 15.68 (7.96-27.53) | -0.34 (-0.38--0.29) |
| Saint Lucia | 23.16 (11.62-40.33) | 37.72 (18.71-62.8) | 0.63 (0.61-0.56) | 13.95 (7-24.3) | 10.51 (5.22-17.5) | -0.93 (-0.96--0.91) |
| Saint Vincent and the Grenadines | 26.94 (13.37-45.58) | 36.14 (17.41-61.72) | 0.34 (0.3-0.35) | 14.29 (7.1-24.19) | 10.95 (5.27-18.7) | -1.14 (-1.39--0.88) |
| Samoa | 14.96 (7.84-24.14) | 16.87 (8.09-27.99) | 0.13 (0.03-0.16) | 2.39 (1.25-3.86) | 2.56 (1.23-4.25) | -0.26 (-0.68-0.17) |
| San Marino | 301.77 (166.2-485.1) | 586.2 (320.67-964.08) | 0.94 (0.93-0.99) | 22.64 (12.47-36.4) | 15.67 (8.57-25.77) | -1.71 (-1.93--1.5) |
| São Tomé and Príncipe | 0.94 (0.55-1.38) | 1.38 (0.81-2.08) | 0.47 (0.47-0.51) | 15.81 (9.18-23.11) | 10.98 (6.45-16.57) | -1.32 (-1.46--1.17) |
| Saudi Arabia | 303.63 (167.6-467.64) | 608.93 (384.81-898.07) | 1.01 (1.3-0.92) | 11.58 (6.39-17.84) | 16.11 (10.18-23.75) | 1.85 (1.65-2.06) |
| Senegal | 26.01 (12.55-44.21) | 53.48 (26.36-93.9) | 1.06 (1.1-1.12) | 14.72 (7.1-25.02) | 12.14 (5.98-21.31) | -0.52 (-0.6--0.43) |
| Serbia | 689.23 (372.82-1132.51) | 1224.47 (662.48-2038.89) | 0.78 (0.78-0.8) | 21.45 (11.6-35.25) | 17.89 (9.68-29.78) | -0.69 (-0.77--0.61) |
| Seychelles | 150.88 (76.6-255.4) | 221.16 (110.26-392.85) | 0.47 (0.44-0.54) | 17.86 (9.07-30.23) | 11.98 (5.97-21.27) | -1.58 (-1.74--1.41) |
| Sierra Leone | 118.08 (61.82-211.67) | 179.08 (92.32-305.02) | 0.52 (0.49-0.44) | 18.77 (9.83-33.65) | 15.32 (7.9-26.09) | -0.92 (-1.04--0.8) |
| Singapore | 5.73 (3.24-8.65) | 16.01 (9.53-24.17) | 1.79 (1.94-1.79) | 4.71 (2.66-7.11) | 4.73 (2.82-7.14) | -0.26 (-0.41--0.11) |
| Slovakia | 77.15 (39.92-134.15) | 228.73 (118.42-399.66) | 1.96 (1.97-1.98) | 17.55 (9.08-30.52) | 16.43 (8.51-28.71) | 0.04 (-0.07-0.16) |
| Slovenia | 190.97 (98.96-321.53) | 254.06 (128.4-439.5) | 0.33 (0.3-0.37) | 19.54 (10.13-32.9) | 14.2 (7.18-24.56) | -1.19 (-1.34--1.05) |
| Solomon Islands | 68.04 (34.81-114.76) | 125.53 (64.6-208.39) | 0.84 (0.86-0.82) | 12.88 (6.59-21.72) | 9.72 (5-16.14) | -0.6 (-0.79--0.42) |
| Somalia | 164.08 (90.18-255.14) | 237.33 (126.63-384.23) | 0.45 (0.4-0.51) | 7.85 (4.31-12.2) | 7.48 (3.99-12.11) | 0.26 (-0.31-0.84) |
| South Africa | 76.43 (39.69-131.91) | 211.62 (110.13-354.56) | 1.77 (1.77-1.69) | 21.23 (11.02-36.64) | 22.44 (11.68-37.6) | 0.05 (-0.09-0.19) |
| South Korea | 5.64 (3.27-8.4) | 13.15 (7.82-19.86) | 1.33 (1.39-1.36) | 3.89 (2.25-5.79) | 3.06 (1.82-4.62) | -0.63 (-0.7--0.56) |
| South Sudan | 95.48 (48.27-164.25) | 197.6 (101.86-337.14) | 1.07 (1.11-1.05) | 20.4 (10.32-35.1) | 18.69 (9.63-31.88) | -0.57 (-0.72--0.43) |
| Spain | 7.52 (3.87-12.48) | 29.18 (16.54-44.57) | 2.88 (3.27-2.57) | 5.38 (2.77-8.94) | 8.35 (4.73-12.75) | 0.92 (0.66-1.19) |
| Sri Lanka | 1.24 (0.75-1.91) | 2.72 (1.61-4.04) | 1.19 (1.15-1.12) | 12.42 (7.52-19.18) | 9.5 (5.6-14.1) | -1.02 (-1.09--0.95) |
| Sudan | 375.68 (191.46-654.85) | 627.83 (315.26-1065.58) | 0.67 (0.65-0.63) | 20.82 (10.61-36.3) | 15.23 (7.65-25.85) | -0.81 (-0.98--0.64) |
| Suriname | 0.49 (0.29-0.71) | 0.89 (0.5-1.36) | 0.82 (0.72-0.92) | 5.08 (3-7.43) | 4.3 (2.43-6.53) | -0.5 (-0.74--0.25) |
| Sweden | 127.71 (69.05-197.22) | 575.28 (329.92-860.89) | 3.5 (3.78-3.37) | 9.79 (5.29-15.12) | 29.88 (17.14-44.72) | 4.17 (3.83-4.5) |
| Switzerland | 0.79 (0.38-1.27) | 2.05 (1.03-3.34) | 1.59 (1.71-1.63) | 14.28 (6.87-23.08) | 15.13 (7.58-24.59) | 0.58 (0.44-0.73) |
| Syria | 0.75 (0.44-1.17) | 2.94 (1.78-4.4) | 2.92 (3.05-2.76) | 5.8 (3.39-9.07) | 7.8 (4.73-11.66) | 0.88 (0.64-1.12) |
| Taiwan (province of China) | 0.74 (0.43-1.16) | 1.61 (0.95-2.43) | 1.18 (1.21-1.09) | 6.68 (3.85-10.45) | 10.29 (6.06-15.53) | 1.41 (1.29-1.52) |
| Tajikistan | 228.54 (115.95-392.94) | 354.9 (181.68-599.37) | 0.55 (0.57-0.53) | 21.85 (11.09-37.57) | 14.97 (7.66-25.27) | -1.25 (-1.29--1.21) |
| Tanzania | 0.39 (0.22-0.58) | 1.31 (0.75-1.99) | 2.36 (2.41-2.43) | 2.79 (1.58-4.17) | 4.07 (2.33-6.19) | 1.11 (0.94-1.29) |
| Thailand | 2570.92 (1624.9-3621.37) | 5699.62 (3551.19-7697.85) | 1.22 (1.19-1.13) | 18.47 (11.68-26.02) | 25.78 (16.06-34.82) | 0.82 (0.68-0.96) |
| The Bahamas | 1000.44 (616.9-1496.93) | 2142.27 (1277.32-3102.22) | 1.14 (1.07-1.07) | 14.35 (8.85-21.47) | 12.26 (7.31-17.76) | -0.64 (-1--0.29) |
| The Gambia | 19.24 (9.83-33.88) | 40.93 (21.09-68.34) | 1.13 (1.15-1.02) | 20.69 (10.58-36.44) | 16.89 (8.7-28.19) | -0.69 (-0.75--0.63) |
| Timor-Leste | 1.77 (1.06-2.63) | 2.44 (1.48-3.61) | 0.38 (0.4-0.37) | 18.62 (11.22-27.75) | 18.17 (11.02-26.85) | 0.02 (-0.06-0.1) |
| Togo | 0.06 (0.03-0.09) | 0.06 (0.03-0.09) | 0 (0-0) | 8.12 (4.51-12.49) | 6.34 (3.72-9.73) | -1.03 (-1.13--0.94) |
| Tokelau | 0.06 (0.04-0.09) | 0.04 (0.02-0.05) | -0.33 (-0.5--0.44) | 16.04 (9.62-23.76) | 9.16 (5.46-13.86) | -1.57 (-1.68--1.46) |
| Tonga | 101.64 (56.67-154.76) | 198.56 (112.53-298.64) | 0.95 (0.99-0.93) | 3.8 (2.12-5.79) | 5.4 (3.06-8.13) | 0.9 (0.65-1.15) |
| Trinidad and Tobago | 131.28 (69.25-228.11) | 174.8 (85.26-290.42) | 0.33 (0.23-0.27) | 19.81 (10.45-34.41) | 15.79 (7.7-26.23) | -0.69 (-0.74--0.63) |
| Tunisia | 62.16 (32.16-105.71) | 104.57 (53.58-172.16) | 0.68 (0.67-0.63) | 19.43 (10.05-33.04) | 12.64 (6.48-20.82) | -1.22 (-1.28--1.17) |
| Türkiye | 126.72 (63.56-218) | 222.86 (114.19-383.95) | 0.76 (0.8-0.76) | 17.52 (8.79-30.14) | 14.82 (7.6-25.54) | -0.6 (-0.64--0.56) |
| Turkmenistan | 0.37 (0.22-0.55) | 1.03 (0.62-1.54) | 1.78 (1.82-1.8) | 15.1 (8.88-22.7) | 10.6 (6.39-15.86) | -2.04 (-2.36--1.72) |
| Tuvalu | 0.24 (0.14-0.37) | 0.47 (0.27-0.69) | 0.96 (0.93-0.86) | 15.2 (8.99-22.85) | 11.16 (6.51-16.53) | -1.38 (-1.53--1.24) |
| Uganda | 113.8 (58.41-193.74) | 224.86 (110.25-392.35) | 0.98 (0.89-1.03) | 15.37 (7.89-26.17) | 11.05 (5.42-19.28) | -1.1 (-1.17--1.04) |
| UK | 0.03 (0.02-0.04) | 0.03 (0.02-0.04) | 0 (0-0) | 12.03 (7.02-18.04) | 11.86 (6.94-17.47) | -0.11 (-0.27-0.06) |
| Ukraine | 0.24 (0.14-0.38) | 0.48 (0.28-0.72) | 1 (1-0.89) | 3.79 (2.22-5.87) | 3.72 (2.14-5.57) | -0.58 (-1.07--0.08) |
| United Arab Emirates | 64.88 (37.76-99.37) | 199.6 (125.3-302.55) | 2.08 (2.32-2.04) | 10.69 (6.22-16.38) | 17.1 (10.73-25.92) | 1.6 (1.38-1.82) |
| Uruguay | 29.46 (16.16-45.52) | 36.84 (20.48-57.32) | 0.25 (0.27-0.26) | 6.29 (3.45-9.72) | 3.59 (1.99-5.58) | -1.63 (-1.99--1.27) |
| United States | 80.47 (40.47-137.5) | 86.01 (43.4-146.4) | 0.07 (0.07-0.06) | 19.4 (9.76-33.16) | 13.81 (6.97-23.51) | -1.22 (-1.36--1.08) |
| Uzbekistan | 7.02 (3.52-11.8) | 7.79 (4-13.39) | 0.11 (0.14-0.13) | 17.78 (8.91-29.89) | 10.17 (5.22-17.46) | -1.71 (-1.83--1.59) |
| Vanuatu | 101.85 (50.36-173.48) | 138.45 (67.6-231.49) | 0.36 (0.34-0.33) | 21.98 (10.87-37.44) | 14.91 (7.28-24.93) | -1.22 (-1.27--1.18) |
| Venezuela | 301.37 (171.77-473.14) | 1304.17 (791.22-1952.74) | 3.33 (3.61-3.13) | 10.87 (6.19-17.06) | 17.32 (10.51-25.93) | 0.82 (0.36-1.28) |
| Viet Nam | 1.49 (0.73-2.55) | 1.55 (0.79-2.72) | 0.04 (0.08-0.07) | 13.28 (6.54-22.78) | 8.5 (4.34-14.89) | -1.47 (-1.61--1.33) |
| Virgin Islands | 66.16 (38.46-101.26) | 114.84 (68.41-174.99) | 0.74 (0.78-0.73) | 4.45 (2.59-6.81) | 3.69 (2.2-5.62) | -0.42 (-0.62--0.21) |
| Yemen | 77.91 (39.26-135.44) | 186.36 (90.27-319.85) | 1.39 (1.3-1.36) | 12.16 (6.13-21.13) | 10.39 (5.03-17.84) | -0.33 (-0.39--0.26) |
| Zambia | 9.88 (4.97-17.33) | 19.77 (9.88-33.85) | 1 (0.99-0.95) | 17.84 (8.98-31.3) | 12.79 (6.39-21.89) | -0.82 (-0.9--0.75) |
| Zimbabwe | 119.24 (61.82-204.46) | 233.79 (121.62-403.2) | 0.96 (0.97-0.97) | 11.68 (6.05-20.02) | 8.57 (4.46-14.78) | -0.98 (-1.04--0.93) |

**Table S12. Prevalent cases, prevalence rates, and EAPC trends of HF impairment under the ILD&PS cause category across 204 countries and territories from 1990 to 2021 among adults aged ≥55 years.**

| **Location** | **Prevalent Cases** | |  | **Prevalent Rates** | |  |
| --- | --- | --- | --- | --- | --- | --- |
|  | **1990  (95% UI)** | **2021  (95% UI)** | **Relative change, 1990–2021(95% UI)** | **1990, per 100,000 (95% UI)** | **2021, per 100,000 (95% UI)** | **EAPC (95% CI)** |
| Afghanistan | 9.655 (6.807-12.999) | 2.797 (1.912-3.84) | -0.71 (-0.72--0.7) | 1.22 (0.86-1.64) | 0.29 (0.2-0.4) | -5.68 (-8.29--3) |
| Albania | 324.697 (239.028-423.233) | 3748.191 (2802.254-4932.571) | 10.54 (10.72-10.65) | 5.95 (4.38-7.75) | 30.54 (22.84-40.2) | 5.85 (5.21-6.5) |
| Algeria | 343.235 (242.495-457.652) | 3638.018 (3027.945-4377.17) | 9.6 (11.49-8.56) | 3.59 (2.54-4.79) | 23.51 (19.57-28.29) | 6.61 (6.16-7.06) |
| American Samoa | 1.579 (1.101-2.184) | 5.662 (3.92-8.315) | 2.59 (2.56-2.81) | 0.03 (0.02-0.05) | 0.04 (0.03-0.06) | 0.25 (0.08-0.41) |
| Andorra | 75.496 (52.272-99.614) | 1014.542 (730.357-1372.213) | 12.44 (12.97-12.78) | 4.49 (3.11-5.92) | 22.66 (16.31-30.65) | 4.86 (4.57-5.16) |
| Angola | 0.071 (0.055-0.09) | 0.237 (0.171-0.33) | 2.34 (2.11-2.67) | 0.82 (0.64-1.05) | 1.26 (0.9-1.75) | 1.98 (1.62-2.34) |
| Antigua and Barbuda | 0.736 (0.556-0.981) | 3.193 (2.419-4.199) | 3.34 (3.35-3.28) | 0.1 (0.08-0.13) | 0.15 (0.11-0.19) | 1.55 (1.4-1.7) |
| Argentina | 18.261 (13.61-23.944) | 92.081 (69.124-120.96) | 4.04 (4.08-4.05) | 1.03 (0.76-1.35) | 1.91 (1.44-2.51) | 1.95 (1.87-2.03) |
| Armenia | 0.294 (0.219-0.391) | 1.628 (1.199-2.147) | 4.54 (4.47-4.49) | 1.2 (0.89-1.59) | 2.26 (1.67-2.98) | 3 (2.68-3.32) |
| Australia | 24.61 (17.607-33.257) | 81.93 (55.473-122.925) | 2.33 (2.15-2.7) | 0.15 (0.11-0.21) | 0.2 (0.13-0.29) | 0.69 (0.6-0.78) |
| Austria | 4.745 (3.586-6.281) | 26.445 (20.103-34.13) | 4.57 (4.61-4.43) | 0.28 (0.21-0.37) | 0.77 (0.59-1) | 4.03 (3.63-4.43) |
| Azerbaijan | 1.064 (0.783-1.452) | 4.266 (3.27-5.474) | 3.01 (3.18-2.77) | 2.27 (1.67-3.1) | 4.68 (3.59-6.01) | 2.96 (2.59-3.34) |
| Bahrain | 0.183 (0.083-0.287) | 0.8 (0.368-1.269) | 3.37 (3.43-3.42) | 1.17 (0.53-1.84) | 1.34 (0.61-2.12) | 0.19 (0.05-0.34) |
| Bangladesh | 358.109 (235.644-515.966) | 3123.608 (2129.327-4416.788) | 7.72 (8.04-7.56) | 2.41 (1.59-3.47) | 14.85 (10.13-21) | 7.01 (6.6-7.43) |
| Barbados | 8.678 (6.416-11.705) | 28.791 (21.129-38.761) | 2.32 (2.29-2.31) | 0.33 (0.24-0.44) | 0.51 (0.37-0.69) | 2.11 (1.85-2.38) |
| Belarus | 0.076 (0.057-0.101) | 0.159 (0.117-0.212) | 1.09 (1.05-1.1) | 0.77 (0.58-1.03) | 1.06 (0.78-1.41) | 1.25 (0.94-1.57) |
| Belgium | 100.995 (67.348-145.626) | 64.02 (38.822-102.652) | -0.37 (-0.42--0.3) | 0.81 (0.54-1.17) | 0.47 (0.29-0.76) | -3.27 (-3.82--2.71) |
| Belize | 0.338 (0.253-0.445) | 1.138 (0.849-1.534) | 2.37 (2.36-2.45) | 0.1 (0.07-0.13) | 0.15 (0.11-0.2) | 1.52 (1.38-1.67) |
| Benin | 0.693 (0.521-0.922) | 3.375 (2.56-4.492) | 3.87 (3.91-3.87) | 4.5 (3.39-5.99) | 6.74 (5.11-8.97) | 2.21 (1.8-2.62) |
| Bermuda | 13.002 (9.708-16.974) | 69.503 (52.723-89.485) | 4.35 (4.43-4.27) | 0.88 (0.66-1.15) | 1.42 (1.08-1.83) | 1.82 (1.7-1.95) |
| Bhutan | 1303.487 (801.044-1899.59) | 14160.246 (9207.93-20276.297) | 9.86 (10.49-9.67) | 4.4 (2.71-6.41) | 27.13 (17.64-38.84) | 5.6 (5.18-6.02) |
| Bolivia | 26.841 (16.607-38.133) | 201.52 (141.076-278.788) | 6.51 (7.49-6.31) | 1.59 (0.98-2.26) | 6.88 (4.81-9.51) | 6.57 (6-7.14) |
| Bosnia and Herzegovina | 0.659 (0.492-0.876) | 3.839 (2.913-5.004) | 4.83 (4.92-4.71) | 4.3 (3.21-5.71) | 7.06 (5.36-9.2) | 2.23 (1.91-2.56) |
| Botswana | 3.026 (2.27-3.959) | 12.998 (9.566-17.073) | 3.3 (3.21-3.31) | 0.49 (0.37-0.65) | 0.78 (0.57-1.02) | 1.23 (1.03-1.43) |
| Brazil | 4.693 (3.019-7.015) | 5.111 (2.117-8.338) | 0.09 (-0.3-0.19) | 0.42 (0.27-0.63) | 0.49 (0.2-0.79) | -0.04 (-0.99-0.91) |
| Brunei | 7.459 (5.759-9.785) | 31.638 (23.63-41.084) | 3.24 (3.1-3.2) | 0.19 (0.15-0.25) | 0.37 (0.28-0.49) | 2.53 (2.42-2.64) |
| Bulgaria | 0.131 (0.099-0.173) | 0.347 (0.26-0.453) | 1.65 (1.63-1.62) | 1.08 (0.82-1.43) | 1.73 (1.29-2.26) | 2 (1.78-2.23) |
| Burkina Faso | 28.343 (21.57-36.916) | 148.084 (113.417-193.101) | 4.22 (4.26-4.23) | 0.48 (0.36-0.62) | 0.76 (0.58-0.99) | 1.73 (1.6-1.86) |
| Burundi | 4.512 (3.286-5.981) | 15.066 (11.23-19.725) | 2.34 (2.42-2.3) | 0.84 (0.61-1.11) | 1.27 (0.95-1.66) | 1.49 (1.2-1.78) |
| Cabo Verde | 0.565 (0.41-0.768) | 1.068 (0.781-1.441) | 0.89 (0.9-0.88) | 0.92 (0.67-1.25) | 0.95 (0.69-1.28) | 1.94 (1.34-2.54) |
| Cambodia | 100.063 (62.202-139.374) | 1733.745 (1334.269-2240.216) | 16.33 (20.45-15.07) | 2.01 (1.25-2.8) | 10.37 (7.98-13.4) | 6.74 (6.2-7.29) |
| Cameroon | 0.221 (0.16-0.301) | 1.422 (1.047-1.888) | 5.43 (5.54-5.27) | 1.51 (1.09-2.06) | 3.38 (2.49-4.49) | 3.46 (2.99-3.93) |
| Canada | 0.017 (0.012-0.023) | 0.031 (0.023-0.041) | 0.82 (0.92-0.78) | 0.65 (0.48-0.89) | 0.53 (0.38-0.7) | -1.14 (-1.44--0.84) |
| Central African Republic | 0.051 (0.04-0.066) | 0.239 (0.181-0.321) | 3.69 (3.52-3.86) | 0.12 (0.09-0.15) | 0.17 (0.13-0.23) | 1.09 (0.97-1.21) |
| Chad | 2.122 (1.574-2.857) | 10.237 (7.885-13.183) | 3.82 (4.01-3.61) | 0.72 (0.53-0.97) | 1.94 (1.49-2.49) | 3.77 (3.16-4.39) |
| Chile | 0.036 (0.024-0.051) | 0.333 (0.239-0.451) | 8.25 (8.96-7.84) | 0.3 (0.2-0.43) | 1.31 (0.94-1.77) | 4.97 (4.09-5.85) |
| China | 188.509 (131.798-265.273) | 747.76 (502.086-1089.551) | 2.97 (2.81-3.11) | 7.94 (5.55-11.17) | 22.21 (14.91-32.36) | 4.2 (3.85-4.55) |
| Colombia | 0.517 (0.401-0.66) | 2.158 (1.638-2.84) | 3.17 (3.08-3.3) | 1.19 (0.92-1.52) | 1.93 (1.47-2.54) | 2 (1.73-2.26) |
| Comoros | 0.095 (0.069-0.128) | 0.317 (0.232-0.431) | 2.34 (2.36-2.37) | 1.61 (1.18-2.18) | 2.68 (1.96-3.64) | 2.07 (1.74-2.4) |
| Congo (Brazzaville) | 8.396 (4.953-13.177) | 21.198 (12.393-33.982) | 1.52 (1.5-1.58) | 1.82 (1.08-2.86) | 1.96 (1.15-3.15) | 0.57 (0.48-0.66) |
| Cook Islands | 4.051 (3.014-5.292) | 13.466 (10.12-17.626) | 2.32 (2.36-2.33) | 1.37 (1.02-1.79) | 1.64 (1.23-2.15) | 0.43 (0.33-0.52) |
| Costa Rica | 2.041 (1.493-2.698) | 14.692 (10.981-19.503) | 6.2 (6.35-6.23) | 1.49 (1.09-1.96) | 4.22 (3.15-5.6) | 4.36 (4.01-4.71) |
| Côte d'Ivoire | 16.773 (12.769-21.902) | 397.762 (298.902-522.465) | 22.71 (22.41-22.85) | 0.58 (0.44-0.76) | 4.16 (3.13-5.46) | 7.36 (6.77-7.95) |
| Croatia | 45.229 (33.928-60.163) | 236.209 (176.284-310.403) | 4.22 (4.2-4.16) | 8.63 (6.47-11.47) | 15.28 (11.41-20.08) | 2.26 (2.07-2.45) |
| Cuba | 458.352 (361.47-575.256) | 3482.834 (2710.681-4442.781) | 6.6 (6.5-6.72) | 23.25 (18.33-29.18) | 62.27 (48.46-79.43) | 4.27 (3.91-4.64) |
| Cyprus | 41.605 (31.881-54.027) | 526.862 (394.257-699.972) | 11.66 (11.37-11.96) | 4.84 (3.71-6.28) | 19.04 (14.25-25.29) | 5.08 (4.72-5.44) |
| Czechia | 0.086 (0.064-0.115) | 0.268 (0.199-0.365) | 2.12 (2.11-2.17) | 0.38 (0.28-0.51) | 0.49 (0.37-0.67) | 1.2 (1.08-1.33) |
| Denmark | 1.761 (1.023-2.512) | 46.543 (32.819-63.154) | 25.43 (31.08-24.14) | 0.49 (0.28-0.69) | 3.06 (2.16-4.16) | 6.18 (5.9-6.46) |
| Djibouti | 9.897 (7.628-12.639) | 103.65 (78.471-134.216) | 9.47 (9.29-9.62) | 3.48 (2.68-4.44) | 10.83 (8.2-14.02) | 4.33 (3.95-4.7) |
| Dominica | 0.055 (0.041-0.074) | 0.074 (0.055-0.1) | 0.35 (0.34-0.35) | 0.68 (0.51-0.93) | 0.56 (0.42-0.76) | -0.94 (-1.04--0.84) |
| Dominican Republic | 2.314 (1.414-3.324) | 4.15 (2.438-6.075) | 0.79 (0.72-0.83) | 1.34 (0.82-1.92) | 1.05 (0.62-1.54) | -0.69 (-0.9--0.48) |
| DR Congo | 0.152 (0.112-0.202) | 0.472 (0.344-0.652) | 2.11 (2.07-2.23) | 0.27 (0.2-0.36) | 0.34 (0.25-0.48) | 1.22 (0.88-1.57) |
| Ecuador | 15.336 (11.944-19.688) | 53.598 (40.625-69.626) | 2.49 (2.4-2.54) | 3.14 (2.44-4.03) | 5.24 (3.97-6.81) | 2.28 (2.09-2.48) |
| Egypt | 600.768 (425.384-835.549) | 4631.722 (3186.857-6948.341) | 6.71 (6.49-7.32) | 0.42 (0.3-0.58) | 1.22 (0.84-1.83) | 3.79 (3.59-4) |
| El Salvador | 18.505 (14.111-24.55) | 153.289 (112.697-205.092) | 7.28 (6.99-7.35) | 3.22 (2.45-4.27) | 8.37 (6.16-11.2) | 3.47 (3.23-3.7) |
| Equatorial Guinea | 224.349 (162.767-306.11) | 1854.221 (1296.958-2655.417) | 7.26 (6.97-7.67) | 3.23 (2.34-4.4) | 8.6 (6.02-12.32) | 3.22 (2.95-3.49) |
| Eritrea | 252.135 (156.687-366.453) | 430.222 (269.03-649.745) | 0.71 (0.72-0.77) | 3.46 (2.15-5.03) | 2.87 (1.79-4.33) | -0.67 (-0.75--0.59) |
| Estonia | 3.583 (2.753-4.658) | 18.833 (14.307-24.586) | 4.26 (4.2-4.28) | 1.44 (1.11-1.87) | 2.31 (1.76-3.02) | 1.85 (1.75-1.96) |
| Eswatini | 18.892 (12.301-26.869) | 157.05 (105.297-221.209) | 7.31 (7.56-7.23) | 2.87 (1.87-4.08) | 11.01 (7.38-15.51) | 4.94 (4.77-5.11) |
| Ethiopia | 6.984 (4.948-9.269) | 14.781 (10.849-20.28) | 1.12 (1.19-1.19) | 1.43 (1.02-1.9) | 1.88 (1.38-2.58) | 3.34 (2.43-4.25) |
| Federated States of Micronesia | 4.7 (3.123-6.552) | 5.455 (3.656-7.674) | 0.16 (0.17-0.17) | 0.21 (0.14-0.29) | 0.23 (0.15-0.32) | 0.11 (-0.3-0.52) |
| Fiji | 7.805 (5.993-10.028) | 28.656 (21.284-37.93) | 2.67 (2.55-2.78) | 2.34 (1.8-3.01) | 2.72 (2.02-3.61) | 0.63 (0.5-0.76) |
| Finland | 6.469 (4.958-8.519) | 80.833 (62.286-103.958) | 11.5 (11.56-11.2) | 2.67 (2.05-3.52) | 10.87 (8.38-13.98) | 5.38 (5.06-5.71) |
| France | 2.01 (1.311-2.817) | 6.786 (4.413-9.278) | 2.38 (2.37-2.29) | 0.18 (0.12-0.25) | 0.46 (0.3-0.62) | 2.65 (2.32-2.99) |
| Gabon | 4.319 (3.006-5.848) | 1.355 (0.943-1.845) | -0.69 (-0.69--0.68) | 0.84 (0.58-1.14) | 0.16 (0.11-0.22) | -5.94 (-6.86--5) |
| Georgia | 4.054 (2.779-5.468) | 11.168 (7.798-15.438) | 1.75 (1.81-1.82) | 1.17 (0.8-1.58) | 1.42 (0.99-1.96) | 0.49 (0.31-0.67) |
| Germany | 5.738 (4.015-7.777) | 8.216 (5.41-11.394) | 0.43 (0.35-0.47) | 0.66 (0.46-0.89) | 0.43 (0.28-0.59) | -1.23 (-1.32--1.13) |
| Ghana | 13.99 (10.583-18.372) | 108.168 (81.724-140.482) | 6.73 (6.72-6.65) | 0.89 (0.68-1.17) | 2.07 (1.56-2.68) | 3.23 (2.78-3.69) |
| Greece | 4.871 (3.522-6.444) | 15.74 (11.121-21.604) | 2.23 (2.16-2.35) | 0.47 (0.34-0.62) | 0.96 (0.68-1.32) | 2.88 (2.61-3.15) |
| Greenland | 63.465 (37.914-97.369) | 57.486 (34.916-82.19) | -0.09 (-0.08--0.16) | 1.27 (0.76-1.95) | 0.96 (0.58-1.37) | -0.53 (-0.78--0.27) |
| Grenada | 4.655 (3.148-6.309) | 7.747 (5.016-10.699) | 0.66 (0.59-0.7) | 0.63 (0.43-0.86) | 0.71 (0.46-0.98) | 0.65 (0.39-0.91) |
| Guam | 4.828 (2.976-6.802) | 10.782 (6.554-15.202) | 1.23 (1.2-1.23) | 0.23 (0.14-0.33) | 0.38 (0.23-0.54) | 1.02 (0.58-1.47) |
| Guatemala | 148.057 (106.492-204.473) | 1862.988 (1291.588-2634.973) | 11.58 (11.13-11.89) | 1 (0.72-1.38) | 4.3 (2.98-6.08) | 5.37 (5-5.74) |
| Guinea | 0.587 (0.435-0.762) | 12.226 (9.201-16.085) | 19.83 (20.15-20.11) | 0.03 (0.02-0.04) | 0.2 (0.15-0.26) | 8.35 (7.67-9.04) |
| Guinea-Bissau | 5.478 (4.261-6.956) | 24.322 (18.414-31.342) | 3.44 (3.32-3.51) | 1.51 (1.17-1.92) | 2.46 (1.86-3.17) | 1.88 (1.64-2.11) |
| Guyana | 0.318 (0.239-0.412) | 4.888 (3.743-6.388) | 14.37 (14.66-14.5) | 1.13 (0.85-1.47) | 3.01 (2.3-3.93) | 3.87 (3.61-4.13) |
| Haiti | 11.176 (8.349-14.506) | 20.86 (15.691-27.6) | 0.87 (0.88-0.9) | 0.25 (0.19-0.32) | 0.19 (0.14-0.25) | -1.33 (-1.54--1.12) |
| Honduras | 3.832 (2.961-4.994) | 35.953 (27.064-47.068) | 8.38 (8.14-8.42) | 1.78 (1.38-2.32) | 2.86 (2.15-3.74) | 2.53 (2.19-2.87) |
| Hungary | 3.276 (1.997-4.611) | 33.513 (21.245-46.635) | 9.23 (9.64-9.11) | 0.76 (0.46-1.07) | 4.58 (2.9-6.37) | 6.67 (6.45-6.89) |
| Iceland | 1.206 (0.869-1.645) | 11.802 (8.242-16.751) | 8.79 (8.48-9.18) | 0.03 (0.02-0.04) | 0.09 (0.06-0.13) | 4.93 (4.65-5.2) |
| India | 2.47 (1.893-3.228) | 22.134 (17.022-28.454) | 7.96 (7.99-7.81) | 2.7 (2.07-3.53) | 4.75 (3.65-6.1) | 3.12 (2.33-3.93) |
| Indonesia | 6.511 (4.923-8.358) | 55.321 (42.134-71.647) | 7.5 (7.56-7.57) | 1.72 (1.3-2.2) | 5.64 (4.3-7.31) | 4.78 (4.4-5.15) |
| Iran | 77 (54.155-105.681) | 407.761 (275.617-602.235) | 4.3 (4.09-4.7) | 1 (0.7-1.37) | 3.37 (2.27-4.97) | 4.33 (4.01-4.66) |
| Iraq | 7.265 (5.516-9.295) | 24.347 (18.588-31.893) | 2.35 (2.37-2.43) | 0.58 (0.44-0.74) | 0.63 (0.48-0.82) | 0.64 (0.45-0.83) |
| Ireland | 0.137 (0.099-0.183) | 0.346 (0.197-0.551) | 1.53 (0.99-2.01) | 0.13 (0.09-0.17) | 0.2 (0.11-0.32) | 0.37 (0.04-0.69) |
| Israel | 10.997 (6.927-16.272) | 15.215 (9.102-23.255) | 0.38 (0.31-0.43) | 3.32 (2.09-4.91) | 2.52 (1.51-3.85) | -1.02 (-1.17--0.87) |
| Italy | 0.18 (0.142-0.232) | 2.187 (1.65-2.814) | 11.15 (10.62-11.13) | 0.06 (0.05-0.07) | 0.26 (0.2-0.34) | 6.67 (6.09-7.25) |
| Jamaica | 7.192 (5.102-9.707) | 2.348 (1.387-3.74) | -0.67 (-0.73--0.61) | 2.01 (1.42-2.71) | 0.54 (0.32-0.85) | -6.67 (-8.76--4.53) |
| Japan | 193.073 (131.655-272.933) | 199.663 (128.411-303.229) | 0.03 (-0.02-0.11) | 0.61 (0.42-0.86) | 0.47 (0.3-0.71) | -2.44 (-4.02--0.84) |
| Jordan | 14.338 (8.879-20.113) | 45.314 (28.631-64.544) | 2.16 (2.22-2.21) | 0.56 (0.34-0.78) | 1.42 (0.9-2.03) | 3.52 (3.28-3.76) |
| Kazakhstan | 0.618 (0.462-0.802) | 8.641 (6.535-11.406) | 12.98 (13.15-13.22) | 0.03 (0.02-0.03) | 0.14 (0.11-0.19) | 7.18 (6.5-7.87) |
| Kenya | 0.396 (0.294-0.522) | 1.787 (1.333-2.338) | 3.51 (3.53-3.48) | 0.39 (0.29-0.52) | 0.57 (0.43-0.75) | 2.02 (1.69-2.36) |
| Kiribati | 25.128 (19.231-32.614) | 91.115 (68.352-116.926) | 2.63 (2.55-2.59) | 2.82 (2.16-3.66) | 3.03 (2.27-3.88) | 0.88 (0.65-1.11) |
| Kuwait | 5.905 (3.613-8.995) | 14.174 (8.489-22.027) | 1.4 (1.35-1.45) | 3.03 (1.85-4.61) | 2.27 (1.36-3.53) | -0.95 (-1.13--0.77) |
| Kyrgyzstan | 24.04 (14.181-36.069) | 225.385 (151.266-310.549) | 8.38 (9.67-7.61) | 2.02 (1.19-3.04) | 11.2 (7.52-15.43) | 6.24 (5.57-6.92) |
| Laos | 0.282 (0.165-0.402) | 4.307 (2.916-6.024) | 14.27 (16.67-13.99) | 0.59 (0.35-0.84) | 4.42 (2.99-6.19) | 6.57 (6.1-7.05) |
| Latvia | 4.515 (3.356-5.802) | 16.385 (12.094-22.174) | 2.63 (2.6-2.82) | 0.51 (0.38-0.66) | 0.69 (0.51-0.94) | 1.55 (1.34-1.75) |
| Lebanon | 15.02 (10.317-20.56) | 262.737 (188.997-353.122) | 16.49 (17.32-16.18) | 2.21 (1.52-3.02) | 20.02 (14.4-26.91) | 8.55 (8.02-9.09) |
| Lesotho | 0.442 (0.313-0.601) | 2.288 (1.671-3.017) | 4.18 (4.34-4.02) | 4.48 (3.17-6.09) | 8.65 (6.31-11.4) | 2.21 (1.8-2.62) |
| Liberia | 0.272 (0.204-0.359) | 4.957 (3.787-6.352) | 17.22 (17.56-16.69) | 0.03 (0.02-0.04) | 0.21 (0.16-0.27) | 7.91 (7.26-8.57) |
| Libya | 0.028 (0.021-0.037) | 0.056 (0.039-0.078) | 1 (0.86-1.11) | 0.77 (0.56-1.01) | 0.66 (0.47-0.92) | -0.41 (-0.72--0.11) |
| Lithuania | 0.1 (0.075-0.129) | 2.211 (1.669-2.867) | 21.11 (21.25-21.22) | 0.61 (0.46-0.79) | 1.44 (1.09-1.87) | 3.7 (3.09-4.32) |
| Luxembourg | 14.436 (9.938-20.153) | 169.636 (113.254-242.025) | 10.75 (10.4-11.01) | 0.61 (0.42-0.85) | 4.86 (3.24-6.93) | 7.36 (6.87-7.85) |
| Madagascar | 54.493 (42.284-70.146) | 339.504 (259.063-438.207) | 5.23 (5.13-5.25) | 0.91 (0.71-1.17) | 2.05 (1.57-2.65) | 3.37 (3.1-3.65) |
| Malawi | 0.162 (0.123-0.211) | 2.537 (1.905-3.294) | 14.66 (14.49-14.61) | 0.02 (0.02-0.03) | 0.11 (0.08-0.15) | 7.35 (6.72-7.98) |
| Malaysia | 8.734 (5.735-12.148) | 138.59 (99.709-186.456) | 14.87 (16.39-14.35) | 1.08 (0.71-1.5) | 6.93 (4.99-9.33) | 7 (6.55-7.46) |
| Maldives | 1.78 (1.351-2.31) | 23.794 (17.123-31.324) | 12.37 (11.67-12.56) | 3.17 (2.41-4.12) | 3.31 (2.38-4.36) | 0.07 (-0.03-0.17) |
| Mali | 0.246 (0.183-0.322) | 1.498 (1.096-1.985) | 5.09 (4.99-5.16) | 0.02 (0.02-0.03) | 0.12 (0.09-0.16) | 7.98 (7.32-8.65) |
| Malta | 230.081 (176.489-305.034) | 860.059 (649.637-1138.541) | 2.74 (2.68-2.73) | 3.04 (2.33-4.03) | 3.68 (2.78-4.87) | 0.81 (0.68-0.94) |
| Marshall Islands | 3.625 (2.1-5.739) | 7.503 (4.404-11.978) | 1.07 (1.1-1.09) | 3.34 (1.93-5.29) | 3.32 (1.95-5.3) | 0.07 (-0.08-0.23) |
| Mauritania | 2.747 (1.848-3.747) | 0.39 (0.275-0.535) | -0.86 (-0.85--0.86) | 0.36 (0.24-0.49) | 0.04 (0.03-0.05) | -8.16 (-10.25--6.03) |
| Mauritius | 0.925 (0.714-1.197) | 4.305 (3.286-5.512) | 3.65 (3.6-3.6) | 2.33 (1.8-3.02) | 4.32 (3.3-5.54) | 2.17 (2.07-2.26) |
| Mexico | 0.007 (0.005-0.009) | 0.014 (0.01-0.018) | 1 (1-1) | 0.58 (0.42-0.77) | 0.75 (0.55-1.01) | 0.65 (0.53-0.77) |
| Moldova | 2357.078 (1738.176-3108.656) | 9747.897 (6859.201-13626.099) | 3.14 (2.95-3.38) | 3.07 (2.27-4.05) | 4.85 (3.41-6.78) | 1.62 (1.52-1.73) |
| Monaco | 11.876 (8.363-16.216) | 54.625 (34.894-77.576) | 3.6 (3.17-3.78) | 1.76 (1.24-2.4) | 6.04 (3.86-8.58) | 4.67 (4.25-5.08) |
| Mongolia | 1.938 (1.28-2.831) | 23.654 (17.055-32.039) | 11.21 (12.32-10.32) | 2.67 (1.77-3.91) | 14.99 (10.81-20.3) | 6.94 (6.55-7.33) |
| Montenegro | 53.27 (33.171-77.782) | 2120.796 (1388.784-3165.517) | 38.81 (40.87-39.7) | 0.35 (0.22-0.51) | 9.34 (6.11-13.93) | 12.48 (10.35-14.65) |
| Morocco | 94.14 (69.609-125.28) | 1849.863 (1450.251-2349.791) | 18.65 (19.83-17.76) | 2.87 (2.12-3.82) | 24.97 (19.58-31.72) | 7.42 (6.36-8.49) |
| Mozambique | 52.089 (39.318-67.987) | 199.399 (149.584-261.286) | 2.83 (2.8-2.84) | 3.38 (2.55-4.42) | 5.04 (3.78-6.6) | 1.39 (1.31-1.47) |
| Myanmar | 1.393 (0.836-2.195) | 2.119 (1.214-3.364) | 0.52 (0.45-0.53) | 3.08 (1.85-4.86) | 2.34 (1.34-3.72) | -1.1 (-1.62--0.59) |
| Namibia | 227.407 (167.015-310.976) | 462.126 (315.595-668.484) | 1.03 (0.89-1.15) | 2.49 (1.83-3.41) | 2.33 (1.59-3.37) | -0.24 (-0.29--0.2) |
| Nauru | 9.672 (5.721-15.553) | 30.643 (17.068-51.04) | 2.17 (1.98-2.28) | 1.55 (0.92-2.5) | 1.58 (0.88-2.64) | 0.21 (0.16-0.26) |
| Nepal | 1.734 (1.178-2.435) | 1.488 (1.01-2.074) | -0.14 (-0.14--0.15) | 0.53 (0.36-0.75) | 0.21 (0.14-0.29) | -2.99 (-3.41--2.57) |
| Netherlands | 333.443 (228.734-455.512) | 2247.248 (1605.182-3045.215) | 5.74 (6.02-5.69) | 1.59 (1.09-2.17) | 7.13 (5.09-9.66) | 6.89 (6.18-7.61) |
| New Zealand | 33.311 (25.481-43.824) | 732.508 (514.342-1001.851) | 20.99 (19.19-21.86) | 1.01 (0.77-1.32) | 12.52 (8.79-17.12) | 9.31 (8.43-10.2) |
| Nicaragua | 2.816 (1.623-4.531) | 4.847 (2.851-7.9) | 0.72 (0.76-0.74) | 1.49 (0.86-2.4) | 1.36 (0.8-2.21) | -0.22 (-0.26--0.19) |
| Niger | 311.141 (211.23-430.82) | 598.376 (392.341-825.347) | 0.92 (0.86-0.92) | 9.22 (6.26-12.76) | 7.59 (4.97-10.46) | -0.68 (-0.97--0.39) |
| Nigeria | 33.749 (23.575-48.657) | 13.459 (8.419-19.313) | -0.6 (-0.64--0.6) | 1.73 (1.21-2.49) | 0.29 (0.18-0.42) | -6.06 (-6.28--5.84) |
| Niue | 2.51 (1.389-4.209) | 6.235 (3.552-10.231) | 1.48 (1.56-1.43) | 1.41 (0.78-2.36) | 1.43 (0.82-2.35) | 0.29 (0.23-0.35) |
| North Korea | 0.126 (0.095-0.167) | 0.239 (0.179-0.318) | 0.9 (0.88-0.9) | 0.87 (0.66-1.16) | 0.97 (0.73-1.29) | 0.37 (0.2-0.53) |
| North Macedonia | 38.093 (19.829-62.671) | 142.48 (84.863-211.669) | 2.74 (3.28-2.38) | 3.52 (1.83-5.79) | 8.79 (5.23-13.05) | 4.32 (3.95-4.69) |
| Northern Mariana Islands | 1.022 (0.61-1.49) | 12.809 (9.112-17.806) | 11.53 (13.94-10.95) | 1.09 (0.65-1.6) | 7.21 (5.13-10.02) | 7.03 (5.97-8.11) |
| Norway | 23.31 (17.702-28.501) | 122.797 (89.832-156.326) | 4.27 (4.07-4.48) | 1.2 (0.91-1.46) | 4.16 (3.04-5.29) | 4.4 (4.12-4.69) |
| Oman | 0.01 (0.007-0.013) | 0.036 (0.027-0.05) | 2.6 (2.86-2.85) | 0.45 (0.33-0.61) | 0.77 (0.58-1.05) | 2.29 (2.07-2.51) |
| Pakistan | 31.899 (23.005-43.566) | 10.359 (6.825-15.356) | -0.68 (-0.7--0.65) | 1.38 (0.99-1.88) | 0.36 (0.24-0.53) | -5.8 (-6.16--5.43) |
| Palau | 1.81 (1.044-2.9) | 3.207 (1.835-5.083) | 0.77 (0.76-0.75) | 1.85 (1.07-2.96) | 1.79 (1.03-2.84) | 0.17 (0.1-0.25) |
| Palestine | 31.378 (23.442-41.331) | 460.485 (332.6-611.242) | 13.68 (13.19-13.79) | 1.29 (0.96-1.7) | 11.84 (8.55-15.71) | 8.36 (7.62-9.11) |
| Panama | 47.283 (27.12-75.283) | 110.941 (66.652-180.368) | 1.35 (1.46-1.4) | 1.79 (1.03-2.85) | 1.84 (1.1-2.99) | 0.12 (-0.04-0.28) |
| Papua New Guinea | 0.406 (0.279-0.543) | 1.166 (0.797-1.636) | 1.87 (1.86-2.01) | 0.12 (0.08-0.17) | 0.2 (0.13-0.28) | 1.75 (1.53-1.97) |
| Paraguay | 0.593 (0.354-0.913) | 1.612 (0.93-2.583) | 1.72 (1.63-1.83) | 1.86 (1.11-2.86) | 2.04 (1.18-3.27) | 0.77 (0.65-0.89) |
| Peru | 7.271 (4.333-11.287) | 15.173 (9.187-23.886) | 1.09 (1.12-1.12) | 1.93 (1.15-3) | 1.9 (1.15-2.98) | -0.11 (-0.19--0.02) |
| Philippines | 5.455 (3.332-8.298) | 4.109 (2.445-6.624) | -0.25 (-0.27--0.2) | 3.9 (2.38-5.93) | 2.28 (1.35-3.67) | -2.33 (-2.7--1.96) |
| Poland | 0.669 (0.387-1.056) | 1.698 (1.048-2.675) | 1.54 (1.71-1.53) | 2.06 (1.19-3.25) | 2.1 (1.3-3.31) | 0.21 (0.16-0.27) |
| Portugal | 125.843 (88.287-168.114) | 529.694 (348.749-734.682) | 3.21 (2.95-3.37) | 2.26 (1.59-3.02) | 5.68 (3.74-7.87) | 3.25 (2.82-3.69) |
| Puerto Rico | 2537.619 (1694.531-3542.384) | 13791.979 (9358.922-19545.115) | 4.44 (4.52-4.52) | 4.84 (3.23-6.75) | 13.76 (9.34-19.5) | 3.51 (3.1-3.92) |
| Qatar | 0.443 (0.326-0.596) | 3.506 (2.496-4.77) | 6.91 (6.66-7) | 4.19 (3.08-5.63) | 15.05 (10.72-20.48) | 4.76 (4.37-5.16) |
| Romania | 1.551 (0.945-2.383) | 2.057 (1.189-3.256) | 0.33 (0.26-0.37) | 2.43 (1.48-3.73) | 1.79 (1.03-2.83) | -1.02 (-1.17--0.86) |
| Russia | 19.526 (11.482-29.077) | 24.778 (14.917-38.339) | 0.27 (0.3-0.32) | 3.53 (2.07-5.25) | 2.71 (1.63-4.2) | -1.04 (-1.16--0.93) |
| Rwanda | 0.418 (0.246-0.665) | 1.912 (1.095-3.131) | 3.57 (3.45-3.71) | 1.95 (1.15-3.1) | 1.84 (1.06-3.02) | -0.16 (-0.33-0.02) |
| Saint Kitts and Nevis | 22.325 (13.257-32.99) | 43.32 (25.184-68.277) | 0.94 (0.9-1.07) | 3.34 (1.98-4.93) | 2.99 (1.74-4.72) | -0.36 (-0.4--0.32) |
| Saint Lucia | 5.121 (3.102-7.71) | 10.239 (6.013-16.046) | 1 (0.94-1.08) | 3.08 (1.87-4.64) | 2.85 (1.68-4.47) | -0.36 (-0.42--0.3) |
| Saint Vincent and the Grenadines | 6.04 (3.664-9.252) | 8.458 (5.087-13.29) | 0.4 (0.39-0.44) | 3.21 (1.94-4.91) | 2.56 (1.54-4.03) | -0.83 (-0.93--0.73) |
| Samoa | 18.703 (13.385-25.775) | 4.277 (2.701-6.376) | -0.77 (-0.8--0.75) | 2.99 (2.14-4.12) | 0.65 (0.41-0.97) | -6.67 (-8.7--4.59) |
| San Marino | 37.525 (23.885-53.937) | 86.099 (53.878-127.465) | 1.29 (1.26-1.36) | 2.82 (1.79-4.05) | 2.3 (1.44-3.41) | -1.41 (-1.66--1.16) |
| São Tomé and Príncipe | 0.049 (0.036-0.066) | 0.1 (0.07-0.136) | 1.04 (0.94-1.06) | 0.82 (0.6-1.11) | 0.79 (0.55-1.08) | -0.15 (-0.22--0.08) |
| Saudi Arabia | 44.743 (30.277-61.295) | 274.501 (211.762-357.452) | 5.14 (5.99-4.83) | 1.71 (1.15-2.34) | 7.26 (5.6-9.45) | 6.3 (5.89-6.72) |
| Senegal | 2.305 (1.354-3.694) | 6.338 (3.63-10.045) | 1.75 (1.68-1.72) | 1.3 (0.77-2.09) | 1.44 (0.82-2.28) | 0.31 (0.28-0.34) |
| Serbia | 47.831 (29.256-71.813) | 132.693 (85.35-191.03) | 1.77 (1.92-1.66) | 1.49 (0.91-2.24) | 1.94 (1.25-2.79) | 0.94 (0.82-1.06) |
| Seychelles | 17.163 (10.207-27.127) | 31.106 (17.613-51.635) | 0.81 (0.73-0.9) | 2.03 (1.21-3.21) | 1.68 (0.95-2.8) | -0.75 (-0.87--0.64) |
| Sierra Leone | 12.639 (7.404-19.561) | 24.895 (14.801-38.834) | 0.97 (1-0.99) | 2.01 (1.18-3.11) | 2.13 (1.27-3.32) | 0.14 (0.06-0.21) |
| Singapore | 2.586 (1.875-3.588) | 45.31 (33.578-59.803) | 16.52 (16.91-15.67) | 2.13 (1.54-2.95) | 13.39 (9.92-17.68) | 6.62 (6.23-7.01) |
| Slovakia | 14.557 (8.568-22.416) | 41.881 (25.227-61.958) | 1.88 (1.94-1.76) | 3.31 (1.95-5.1) | 3.01 (1.81-4.45) | -0.23 (-0.33--0.12) |
| Slovenia | 18.671 (11.364-27.934) | 30.841 (18.091-47.278) | 0.65 (0.59-0.69) | 1.91 (1.16-2.86) | 1.72 (1.01-2.64) | -0.61 (-0.76--0.45) |
| Solomon Islands | 14.771 (9.071-22.404) | 29.052 (17.343-45.378) | 0.97 (0.91-1.03) | 2.8 (1.72-4.24) | 2.25 (1.34-3.51) | -0.62 (-0.74--0.5) |
| Somalia | 13.054 (9.213-18.181) | 17.879 (11.307-25.971) | 0.37 (0.23-0.43) | 0.62 (0.44-0.87) | 0.56 (0.36-0.82) | 0.73 (0.06-1.41) |
| South Africa | 6.567 (4.007-10.005) | 19.529 (11.486-30.219) | 1.97 (1.87-2.02) | 1.82 (1.11-2.78) | 2.07 (1.22-3.2) | 0.32 (0.18-0.47) |
| South Korea | 3.904 (2.987-5.178) | 15.457 (11.663-20.104) | 2.96 (2.9-2.88) | 2.69 (2.06-3.57) | 3.59 (2.71-4.67) | 1.41 (1.23-1.59) |
| South Sudan | 7.407 (4.367-11.568) | 23.567 (14.141-37.447) | 2.18 (2.24-2.24) | 1.58 (0.93-2.47) | 2.23 (1.34-3.54) | 1.34 (1.23-1.45) |
| Spain | 5.986 (3.618-8.463) | 30.009 (20.781-41.848) | 4.01 (4.74-3.94) | 4.29 (2.59-6.06) | 8.59 (5.95-11.97) | 2.15 (1.93-2.37) |
| Sri Lanka | 0.061 (0.045-0.081) | 0.186 (0.138-0.251) | 2.05 (2.07-2.1) | 0.61 (0.45-0.81) | 0.65 (0.48-0.87) | 0.16 (0.11-0.22) |
| Sudan | 40.885 (25.032-62.901) | 91.676 (55.815-139.869) | 1.24 (1.23-1.22) | 2.27 (1.39-3.49) | 2.22 (1.35-3.39) | 0.27 (0.15-0.4) |
| Suriname | 0.013 (0.009-0.016) | 0.028 (0.021-0.036) | 1.15 (1.33-1.25) | 0.13 (0.1-0.17) | 0.13 (0.1-0.18) | 0.24 (0.03-0.45) |
| Sweden | 16.347 (10.824-22.781) | 184.046 (126.589-260.418) | 10.26 (10.7-10.43) | 1.25 (0.83-1.75) | 9.56 (6.58-13.53) | 7.76 (7.38-8.14) |
| Switzerland | 0.181 (0.095-0.269) | 0.7 (0.401-1.029) | 2.87 (3.22-2.83) | 3.29 (1.72-4.88) | 5.16 (2.95-7.58) | 2.17 (1.94-2.41) |
| Syria | 0.314 (0.232-0.416) | 1.207 (0.889-1.604) | 2.84 (2.83-2.86) | 2.44 (1.8-3.22) | 3.2 (2.36-4.26) | 1.2 (0.84-1.56) |
| Taiwan (province of China) | 0.61 (0.444-0.804) | 1.649 (1.213-2.18) | 1.7 (1.73-1.71) | 5.5 (4.01-7.25) | 10.54 (7.75-13.93) | 2.15 (2.05-2.25) |
| Tajikistan | 25.469 (15.505-38.67) | 54.462 (32.91-83.218) | 1.14 (1.12-1.15) | 2.44 (1.48-3.7) | 2.3 (1.39-3.51) | 0.06 (-0.05-0.18) |
| Tanzania | 0.227 (0.17-0.303) | 1.016 (0.735-1.343) | 3.48 (3.32-3.43) | 1.62 (1.22-2.17) | 3.16 (2.28-4.17) | 2.14 (2.07-2.21) |
| Thailand | 744.558 (559.698-1000.628) | 5289.095 (3985.803-6871.493) | 6.1 (6.12-5.87) | 5.35 (4.02-7.19) | 23.92 (18.03-31.08) | 5.37 (4.72-6.03) |
| The Bahamas | 15.613 (12.011-20.601) | 57.724 (43.672-76.313) | 2.7 (2.64-2.7) | 0.22 (0.17-0.3) | 0.33 (0.25-0.44) | 1.36 (1.08-1.64) |
| The Gambia | 3.228 (1.904-5.25) | 8.472 (5.123-13.108) | 1.62 (1.69-1.5) | 3.47 (2.05-5.65) | 3.49 (2.11-5.41) | -0.13 (-0.24--0.02) |
| Timor-Leste | 0.115 (0.086-0.152) | 0.21 (0.157-0.282) | 0.83 (0.83-0.86) | 1.21 (0.9-1.6) | 1.56 (1.17-2.1) | 1 (0.91-1.09) |
| Togo | 0.003 (0.002-0.005) | 0.005 (0.004-0.007) | 0.67 (1-0.4) | 0.47 (0.34-0.64) | 0.57 (0.4-0.78) | 0.53 (0.4-0.66) |
| Tokelau | 0.004 (0.003-0.006) | 0.004 (0.003-0.005) | 0 (0--0.17) | 1.18 (0.88-1.58) | 1 (0.73-1.34) | -0.06 (-0.24-0.13) |
| Tonga | 14.177 (10.169-18.86) | 122.202 (82.47-165.576) | 7.62 (7.11-7.78) | 0.53 (0.38-0.71) | 3.32 (2.24-4.5) | 5.55 (5.08-6.02) |
| Trinidad and Tobago | 12.843 (7.643-20.028) | 16.212 (9.282-25.512) | 0.26 (0.21-0.27) | 1.94 (1.15-3.02) | 1.46 (0.84-2.3) | -1.42 (-1.8--1.03) |
| Tunisia | 10.859 (6.803-15.848) | 21.019 (12.775-31.763) | 0.94 (0.88-1) | 3.39 (2.13-4.95) | 2.54 (1.54-3.84) | -0.92 (-0.99--0.86) |
| Türkiye | 21.889 (13.236-32.165) | 37.187 (22.577-55.468) | 0.7 (0.71-0.72) | 3.03 (1.83-4.45) | 2.47 (1.5-3.69) | -0.77 (-0.84--0.7) |
| Turkmenistan | 0.059 (0.042-0.078) | 0.175 (0.126-0.239) | 1.97 (2-2.06) | 2.4 (1.74-3.2) | 1.8 (1.29-2.46) | -1.82 (-2.27--1.37) |
| Tuvalu | 0.016 (0.012-0.021) | 0.035 (0.026-0.047) | 1.19 (1.17-1.24) | 1 (0.74-1.33) | 0.84 (0.62-1.13) | -1.02 (-1.19--0.86) |
| Uganda | 24.221 (14.287-36.987) | 51.694 (30.85-78.948) | 1.13 (1.16-1.13) | 3.27 (1.93-5) | 2.54 (1.52-3.88) | -0.93 (-1.11--0.74) |
| UK | 0.002 (0.002-0.003) | 0.003 (0.002-0.004) | 0.5 (0-0.33) | 0.86 (0.64-1.17) | 1.24 (0.91-1.67) | 1.34 (1.22-1.45) |
| Ukraine | 0.066 (0.048-0.092) | 0.211 (0.158-0.28) | 2.2 (2.29-2.04) | 1.04 (0.75-1.44) | 1.64 (1.22-2.17) | 1.74 (1.26-2.23) |
| United Arab Emirates | 20.025 (14.555-27.003) | 134.68 (98.472-183.709) | 5.73 (5.77-5.8) | 3.3 (2.4-4.45) | 11.54 (8.44-15.74) | 5.16 (4.76-5.57) |
| Uruguay | 11.627 (7.745-16.135) | 15.505 (10.591-21.489) | 0.33 (0.37-0.33) | 2.48 (1.65-3.45) | 1.51 (1.03-2.09) | -1.17 (-1.44--0.91) |
| United States | 8.791 (5.234-13.626) | 10.421 (6.266-15.826) | 0.19 (0.2-0.16) | 2.12 (1.26-3.29) | 1.67 (1.01-2.54) | -0.79 (-0.95--0.63) |
| Uzbekistan | 1.206 (0.758-1.697) | 1.978 (1.18-2.987) | 0.64 (0.56-0.76) | 3.06 (1.92-4.3) | 2.58 (1.54-3.9) | -0.26 (-0.39--0.12) |
| Vanuatu | 17.1 (10.697-25.347) | 24.656 (14.472-38.555) | 0.44 (0.35-0.52) | 3.69 (2.31-5.47) | 2.66 (1.56-4.15) | -1.02 (-1.15--0.9) |
| Venezuela | 15.174 (10.861-20.896) | 389.127 (282.343-547.15) | 24.64 (25-25.18) | 0.55 (0.39-0.75) | 5.17 (3.75-7.27) | 6.93 (6.05-7.82) |
| Viet Nam | 0.384 (0.229-0.601) | 0.463 (0.268-0.732) | 0.21 (0.17-0.22) | 3.43 (2.05-5.36) | 2.53 (1.47-4.01) | -0.99 (-1.01--0.97) |
| Virgin Islands | 0.43 (0.32-0.557) | 4.387 (3.357-5.679) | 9.2 (9.49-9.2) | 0.03 (0.02-0.04) | 0.14 (0.11-0.18) | 6.89 (6.25-7.55) |
| Yemen | 17.258 (10.007-26.671) | 41.419 (24.109-63.697) | 1.4 (1.41-1.39) | 2.69 (1.56-4.16) | 2.31 (1.34-3.55) | -0.38 (-0.53--0.23) |
| Zambia | 1.92 (1.186-2.8) | 3.823 (2.306-5.731) | 0.99 (0.94-1.05) | 3.47 (2.14-5.06) | 2.47 (1.49-3.71) | -1.02 (-1.08--0.96) |
| Zimbabwe | 38.763 (24.626-56.228) | 97.113 (58.797-145.638) | 1.51 (1.39-1.59) | 3.8 (2.41-5.51) | 3.56 (2.16-5.34) | -0.41 (-0.66--0.16) |

**Table S13. YLDs, YLD rates, and EAPC trends of HF impairment under the ILD&PS cause category across 204 countries and territories from 1990 to 2021 among adults aged ≥55 years.**

| **Location** | **YLDs** | |  | **YLD rates** | |  |
| --- | --- | --- | --- | --- | --- | --- |
|  | **1990 (95% UI)** | **2021 (95% UI)** | **Relative change, 1990–2021(95% UI)** | **1990, per 100,000 (95% UI)** | **2021, per 100,000 (95% UI)** | **EAPC (95% CI)** |
| Afghanistan | 0.8949 (0.5099-1.3346) | 0.2591 (0.143-0.3938) | -0.71 (-0.72--0.7) | 0.113 (0.064-0.168) | 0.027 (0.015-0.041) | -5.68 (-8.29--3) |
| Albania | 30.0915 (17.764-44.6158) | 337.699 (195.7031-534.2105) | 10.22 (10.02-10.97) | 0.551 (0.325-0.817) | 2.752 (1.595-4.353) | 5.74 (5.11-6.37) |
| Algeria | 31.8154 (18.3352-47.8074) | 325.2458 (202.7564-483.6375) | 9.22 (10.06-9.12) | 0.333 (0.192-0.5) | 2.102 (1.31-3.125) | 6.46 (6.01-6.92) |
| American Samoa | 0.1466 (0.0854-0.242) | 0.5255 (0.3039-0.8647) | 2.58 (2.56-2.57) | 0.003 (0.002-0.005) | 0.004 (0.002-0.006) | 0.24 (0.08-0.41) |
| Andorra | 7.0015 (3.9145-10.4285) | 91.7522 (54.1149-145.5133) | 12.1 (12.82-12.95) | 0.416 (0.233-0.62) | 2.049 (1.209-3.25) | 4.77 (4.48-5.06) |
| Angola | 0.0065 (0.004-0.0099) | 0.022 (0.0129-0.0334) | 2.38 (2.22-2.37) | 0.076 (0.047-0.114) | 0.117 (0.068-0.177) | 1.98 (1.62-2.34) |
| Antigua and Barbuda | 0.0682 (0.0405-0.1013) | 0.296 (0.177-0.4397) | 3.34 (3.37-3.34) | 0.009 (0.005-0.014) | 0.014 (0.008-0.02) | 1.55 (1.4-1.7) |
| Argentina | 1.6932 (0.9876-2.5375) | 8.5318 (5.008-12.779) | 4.04 (4.07-4.04) | 0.095 (0.055-0.143) | 0.177 (0.104-0.265) | 1.95 (1.87-2.03) |
| Armenia | 0.0272 (0.0163-0.0416) | 0.151 (0.0875-0.2203) | 4.55 (4.37-4.3) | 0.111 (0.066-0.169) | 0.21 (0.122-0.306) | 3 (2.68-3.32) |
| Australia | 2.2839 (1.3515-3.6716) | 7.6045 (4.2979-12.5611) | 2.33 (2.18-2.42) | 0.014 (0.008-0.023) | 0.018 (0.01-0.03) | 0.69 (0.6-0.78) |
| Austria | 0.44 (0.2557-0.6595) | 2.4528 (1.4143-3.6174) | 4.57 (4.53-4.49) | 0.026 (0.015-0.039) | 0.072 (0.041-0.106) | 4.03 (3.63-4.43) |
| Azerbaijan | 0.0987 (0.0576-0.1494) | 0.3956 (0.2402-0.5818) | 3.01 (3.17-2.89) | 0.21 (0.123-0.319) | 0.434 (0.264-0.638) | 2.96 (2.59-3.34) |
| Bahrain | 0.017 (0.0068-0.0288) | 0.074 (0.0311-0.1278) | 3.35 (3.57-3.44) | 0.108 (0.043-0.184) | 0.124 (0.052-0.213) | 0.19 (0.05-0.34) |
| Bangladesh | 33.205 (17.6736-53.7309) | 284.133 (163.8207-448.9748) | 7.56 (8.27-7.36) | 0.224 (0.119-0.362) | 1.351 (0.779-2.135) | 6.93 (6.51-7.34) |
| Barbados | 0.8046 (0.4763-1.1902) | 2.6713 (1.5582-3.8991) | 2.32 (2.27-2.28) | 0.03 (0.018-0.045) | 0.047 (0.028-0.069) | 2.11 (1.85-2.38) |
| Belarus | 0.007 (0.004-0.0105) | 0.0148 (0.0085-0.0223) | 1.11 (1.12-1.12) | 0.072 (0.041-0.107) | 0.098 (0.056-0.148) | 1.25 (0.94-1.57) |
| Belgium | 9.3696 (5.2973-14.8226) | 5.9482 (3.1522-10.3139) | -0.37 (-0.4--0.3) | 0.075 (0.042-0.119) | 0.044 (0.023-0.076) | -3.26 (-3.82--2.7) |
| Belize | 0.0313 (0.0183-0.0466) | 0.1055 (0.0621-0.1572) | 2.37 (2.39-2.37) | 0.009 (0.005-0.014) | 0.014 (0.008-0.02) | 1.52 (1.38-1.67) |
| Benin | 0.0642 (0.039-0.0956) | 0.3078 (0.18-0.4612) | 3.79 (3.62-3.82) | 0.417 (0.253-0.621) | 0.615 (0.359-0.921) | 2.15 (1.75-2.55) |
| Bermuda | 1.205 (0.7049-1.7573) | 6.4442 (3.824-9.4373) | 4.35 (4.42-4.37) | 0.082 (0.048-0.119) | 0.131 (0.078-0.192) | 1.83 (1.7-1.95) |
| Bhutan | 120.708 (62.7648-201.4828) | 1277.0841 (690.3289-2073.3129) | 9.58 (10-9.29) | 0.408 (0.212-0.68) | 2.446 (1.322-3.972) | 5.51 (5.09-5.93) |
| Bolivia | 2.4872 (1.2771-3.8492) | 18.702 (10.8986-29.2789) | 6.52 (7.53-6.61) | 0.147 (0.076-0.228) | 0.638 (0.372-0.999) | 6.57 (6-7.14) |
| Bosnia and Herzegovina | 0.061 (0.0363-0.0911) | 0.3528 (0.2089-0.5235) | 4.78 (4.75-4.75) | 0.398 (0.237-0.594) | 0.649 (0.384-0.962) | 2.21 (1.87-2.54) |
| Botswana | 0.2806 (0.1672-0.421) | 1.2052 (0.7294-1.8321) | 3.3 (3.36-3.35) | 0.046 (0.027-0.069) | 0.072 (0.044-0.11) | 1.23 (1.03-1.43) |
| Brazil | 0.435 (0.2324-0.7296) | 0.474 (0.1781-0.8552) | 0.09 (-0.23-0.17) | 0.039 (0.021-0.066) | 0.045 (0.017-0.081) | -0.05 (-0.99-0.9) |
| Brunei | 0.6911 (0.4173-1.0401) | 2.9339 (1.7324-4.3587) | 3.25 (3.15-3.19) | 0.018 (0.011-0.027) | 0.035 (0.021-0.052) | 2.53 (2.42-2.64) |
| Bulgaria | 0.0121 (0.0072-0.0186) | 0.0322 (0.0189-0.0477) | 1.66 (1.62-1.56) | 0.1 (0.06-0.154) | 0.16 (0.094-0.237) | 2 (1.78-2.22) |
| Burkina Faso | 2.6271 (1.5846-3.8967) | 13.7187 (8.1118-20.0423) | 4.22 (4.12-4.14) | 0.044 (0.027-0.066) | 0.071 (0.042-0.103) | 1.73 (1.6-1.86) |
| Burundi | 0.4185 (0.2447-0.62) | 1.3965 (0.8081-2.0249) | 2.34 (2.3-2.27) | 0.078 (0.045-0.115) | 0.118 (0.068-0.171) | 1.49 (1.2-1.79) |
| Cabo Verde | 0.0525 (0.0312-0.0803) | 0.099 (0.0591-0.1496) | 0.89 (0.89-0.86) | 0.086 (0.051-0.131) | 0.088 (0.052-0.133) | 1.94 (1.34-2.54) |
| Cambodia | 9.2683 (4.7433-14.6288) | 160.4998 (100.4762-236.7051) | 16.32 (20.18-15.18) | 0.186 (0.095-0.294) | 0.96 (0.601-1.415) | 6.73 (6.18-7.28) |
| Cameroon | 0.0205 (0.0119-0.0314) | 0.1318 (0.0775-0.1949) | 5.43 (5.51-5.21) | 0.14 (0.081-0.215) | 0.313 (0.184-0.463) | 3.46 (2.99-3.93) |
| Canada | 0.0015 (0.0009-0.0023) | 0.0029 (0.0017-0.0043) | 0.93 (0.89-0.87) | 0.061 (0.035-0.091) | 0.049 (0.029-0.073) | -1.14 (-1.44--0.84) |
| Central African Republic | 0.0047 (0.0029-0.0069) | 0.0222 (0.0131-0.0327) | 3.72 (3.52-3.74) | 0.011 (0.007-0.016) | 0.016 (0.009-0.023) | 1.09 (0.97-1.21) |
| Chad | 0.1968 (0.1155-0.3032) | 0.9498 (0.5666-1.3867) | 3.83 (3.91-3.57) | 0.067 (0.039-0.103) | 0.18 (0.107-0.262) | 3.77 (3.16-4.38) |
| Chile | 0.0033 (0.0019-0.0052) | 0.0309 (0.0178-0.0478) | 8.36 (8.37-8.19) | 0.028 (0.016-0.043) | 0.122 (0.07-0.188) | 4.96 (4.09-5.85) |
| China | 17.4582 (9.9893-28.2735) | 68.3467 (34.5797-114.2482) | 2.91 (2.46-3.04) | 0.735 (0.421-1.191) | 2.03 (1.027-3.394) | 4.13 (3.79-4.48) |
| Colombia | 0.0479 (0.0285-0.072) | 0.2001 (0.1198-0.2973) | 3.18 (3.2-3.13) | 0.11 (0.065-0.165) | 0.179 (0.107-0.266) | 1.99 (1.73-2.26) |
| Comoros | 0.0088 (0.0051-0.0134) | 0.0294 (0.0176-0.0445) | 2.34 (2.45-2.32) | 0.149 (0.087-0.229) | 0.248 (0.149-0.376) | 2.07 (1.74-2.4) |
| Congo (Brazzaville) | 0.7799 (0.4159-1.3021) | 1.9696 (1.0054-3.2376) | 1.53 (1.42-1.49) | 0.169 (0.09-0.283) | 0.182 (0.093-0.3) | 0.57 (0.48-0.66) |
| Cook Islands | 0.3755 (0.2237-0.5597) | 1.2488 (0.7514-1.8482) | 2.33 (2.36-2.3) | 0.127 (0.076-0.189) | 0.152 (0.091-0.225) | 0.43 (0.33-0.52) |
| Costa Rica | 0.1893 (0.1094-0.2878) | 1.363 (0.7928-2.0079) | 6.2 (6.25-5.98) | 0.138 (0.08-0.21) | 0.391 (0.228-0.576) | 4.36 (4.01-4.71) |
| Côte d'Ivoire | 1.5555 (0.9287-2.3719) | 36.8828 (21.7557-54.2463) | 22.71 (22.43-21.87) | 0.054 (0.032-0.082) | 0.386 (0.227-0.567) | 7.35 (6.77-7.94) |
| Croatia | 4.1207 (2.5172-6.0522) | 21.3596 (12.6795-33.0139) | 4.18 (4.04-4.45) | 0.786 (0.48-1.154) | 1.382 (0.82-2.136) | 2.21 (2.01-2.4) |
| Cuba | 40.9849 (25.7483-61.2103) | 308.6039 (189.6496-460.3361) | 6.53 (6.37-6.52) | 2.079 (1.306-3.104) | 5.517 (3.391-8.23) | 4.25 (3.88-4.61) |
| Cyprus | 3.8491 (2.3127-5.7964) | 47.6386 (28.6641-70.8382) | 11.38 (11.39-11.22) | 0.448 (0.269-0.674) | 1.721 (1.036-2.56) | 4.98 (4.63-5.33) |
| Czechia | 0.0079 (0.0048-0.0118) | 0.0249 (0.0144-0.037) | 2.15 (2-2.14) | 0.035 (0.021-0.052) | 0.046 (0.027-0.068) | 1.21 (1.08-1.33) |
| Denmark | 0.1631 (0.0807-0.2606) | 4.3145 (2.5451-6.4849) | 25.45 (30.54-23.88) | 0.045 (0.022-0.072) | 0.284 (0.168-0.427) | 6.18 (5.9-6.46) |
| Djibouti | 0.9178 (0.5522-1.349) | 9.495 (5.6995-14.5333) | 9.35 (9.32-9.77) | 0.323 (0.194-0.474) | 0.992 (0.595-1.518) | 4.26 (3.89-4.63) |
| Dominica | 0.0051 (0.0029-0.0077) | 0.0068 (0.004-0.0102) | 0.33 (0.38-0.32) | 0.063 (0.037-0.096) | 0.052 (0.03-0.077) | -0.94 (-1.05--0.84) |
| Dominican Republic | 0.2145 (0.11-0.3531) | 0.3848 (0.1943-0.6271) | 0.79 (0.77-0.78) | 0.124 (0.063-0.204) | 0.097 (0.049-0.159) | -0.69 (-0.9--0.48) |
| DR Congo | 0.0141 (0.0083-0.0211) | 0.0437 (0.0256-0.0668) | 2.1 (2.08-2.17) | 0.025 (0.015-0.037) | 0.032 (0.019-0.049) | 1.22 (0.88-1.57) |
| Ecuador | 1.421 (0.8643-2.0954) | 4.9694 (2.9965-7.3577) | 2.5 (2.47-2.51) | 0.291 (0.177-0.429) | 0.486 (0.293-0.72) | 2.28 (2.09-2.48) |
| Egypt | 55.7646 (32.6919-88.5192) | 430.0097 (244.9208-700.7004) | 6.71 (6.49-6.92) | 0.039 (0.023-0.062) | 0.113 (0.065-0.185) | 3.79 (3.59-4) |
| El Salvador | 1.717 (1.0217-2.5907) | 14.1601 (8.1857-20.842) | 7.25 (7.01-7.04) | 0.299 (0.178-0.45) | 0.773 (0.447-1.138) | 3.45 (3.22-3.69) |
| Equatorial Guinea | 20.8022 (12.3042-32.4816) | 170.3778 (98.3463-273.8381) | 7.19 (6.99-7.43) | 0.299 (0.177-0.467) | 0.791 (0.456-1.271) | 3.18 (2.91-3.45) |
| Eritrea | 23.4067 (13.3173-36.8925) | 39.9539 (21.8589-64.687) | 0.71 (0.64-0.75) | 0.321 (0.183-0.506) | 0.266 (0.146-0.431) | -0.66 (-0.75--0.58) |
| Estonia | 0.3321 (0.1963-0.4854) | 1.7463 (1.0365-2.555) | 4.26 (4.28-4.26) | 0.134 (0.079-0.195) | 0.215 (0.127-0.314) | 1.85 (1.75-1.96) |
| Eswatini | 1.7513 (0.9594-2.7846) | 14.3498 (7.7745-23.524) | 7.19 (7.1-7.45) | 0.266 (0.146-0.423) | 1.006 (0.545-1.649) | 4.89 (4.72-5.06) |
| Ethiopia | 0.6471 (0.3838-0.9845) | 1.3699 (0.7979-2.0416) | 1.12 (1.08-1.07) | 0.133 (0.079-0.202) | 0.174 (0.101-0.26) | 3.34 (2.44-4.25) |
| Federated States of Micronesia | 0.4364 (0.2401-0.6791) | 0.5062 (0.2765-0.7807) | 0.16 (0.15-0.15) | 0.019 (0.011-0.03) | 0.021 (0.012-0.033) | 0.1 (-0.31-0.51) |
| Fiji | 0.7233 (0.4317-1.0742) | 2.6573 (1.5777-3.9841) | 2.67 (2.65-2.71) | 0.217 (0.13-0.322) | 0.253 (0.15-0.379) | 0.63 (0.5-0.76) |
| Finland | 0.5999 (0.3638-0.909) | 7.3408 (4.3319-10.8567) | 11.24 (10.91-10.94) | 0.248 (0.15-0.375) | 0.987 (0.583-1.46) | 5.31 (4.98-5.64) |
| France | 0.1863 (0.1028-0.2842) | 0.6295 (0.3199-0.9971) | 2.38 (2.11-2.51) | 0.017 (0.009-0.026) | 0.042 (0.021-0.067) | 2.65 (2.32-2.99) |
| Gabon | 0.4002 (0.2286-0.5924) | 0.1256 (0.0725-0.1858) | -0.69 (-0.68--0.69) | 0.078 (0.044-0.115) | 0.015 (0.009-0.022) | -5.94 (-6.86--5) |
| Georgia | 0.376 (0.2106-0.5635) | 1.0362 (0.5728-1.6225) | 1.76 (1.72-1.88) | 0.108 (0.061-0.162) | 0.132 (0.073-0.206) | 0.49 (0.31-0.67) |
| Germany | 0.5318 (0.3054-0.8156) | 0.7621 (0.4341-1.1718) | 0.43 (0.42-0.44) | 0.061 (0.035-0.094) | 0.04 (0.023-0.061) | -1.22 (-1.32--1.13) |
| Ghana | 1.2972 (0.7826-1.9095) | 10.0271 (5.7208-14.7728) | 6.73 (6.31-6.74) | 0.083 (0.05-0.122) | 0.192 (0.109-0.282) | 3.23 (2.78-3.68) |
| Greece | 0.4514 (0.2658-0.682) | 1.4608 (0.8176-2.3109) | 2.24 (2.08-2.39) | 0.044 (0.026-0.066) | 0.089 (0.05-0.141) | 2.88 (2.61-3.16) |
| Greenland | 5.8831 (2.8082-9.3615) | 5.3344 (2.6737-8.5488) | -0.09 (-0.05--0.09) | 0.118 (0.056-0.187) | 0.089 (0.045-0.142) | -0.53 (-0.78--0.27) |
| Grenada | 0.4315 (0.2447-0.6604) | 0.7183 (0.4075-1.0951) | 0.66 (0.67-0.66) | 0.059 (0.033-0.09) | 0.066 (0.037-0.1) | 0.65 (0.39-0.91) |
| Guam | 0.4477 (0.2323-0.7201) | 0.9996 (0.4989-1.5326) | 1.23 (1.15-1.13) | 0.022 (0.011-0.035) | 0.036 (0.018-0.055) | 1.02 (0.57-1.47) |
| Guatemala | 13.7329 (8.0803-21.4564) | 172.4064 (100.9619-277.1293) | 11.55 (11.49-11.92) | 0.093 (0.055-0.145) | 0.398 (0.233-0.64) | 5.36 (5-5.73) |
| Guinea | 0.0544 (0.0319-0.081) | 1.1335 (0.6717-1.6798) | 19.84 (20.06-19.74) | 0.003 (0.002-0.004) | 0.019 (0.011-0.028) | 8.35 (7.67-9.04) |
| Guinea-Bissau | 0.508 (0.2994-0.7546) | 2.2564 (1.3513-3.3207) | 3.44 (3.51-3.4) | 0.14 (0.083-0.208) | 0.228 (0.136-0.335) | 1.88 (1.64-2.11) |
| Guyana | 0.0294 (0.0181-0.0436) | 0.4531 (0.2759-0.6818) | 14.41 (14.24-14.64) | 0.105 (0.064-0.155) | 0.279 (0.17-0.42) | 3.87 (3.61-4.13) |
| Haiti | 1.0363 (0.6239-1.5488) | 1.9348 (1.1431-2.8566) | 0.87 (0.83-0.84) | 0.023 (0.014-0.035) | 0.017 (0.01-0.026) | -1.33 (-1.54--1.12) |
| Honduras | 0.3552 (0.2179-0.5269) | 3.3275 (2.0292-4.9751) | 8.37 (8.31-8.44) | 0.165 (0.101-0.245) | 0.265 (0.161-0.396) | 2.53 (2.19-2.87) |
| Hungary | 0.3037 (0.1593-0.4762) | 3.1102 (1.6721-4.8057) | 9.24 (9.5-9.09) | 0.071 (0.037-0.111) | 0.425 (0.228-0.656) | 6.67 (6.45-6.89) |
| Iceland | 0.1119 (0.0661-0.1794) | 1.0948 (0.6442-1.7581) | 8.78 (8.75-8.8) | 0.002 (0.001-0.004) | 0.008 (0.005-0.014) | 4.93 (4.65-5.21) |
| India | 0.2291 (0.1396-0.3414) | 2.0418 (1.2712-2.9873) | 7.91 (8.11-7.75) | 0.251 (0.153-0.373) | 0.438 (0.273-0.641) | 3.09 (2.3-3.89) |
| Indonesia | 0.6039 (0.3617-0.885) | 5.1311 (3.0942-7.4298) | 7.5 (7.55-7.4) | 0.159 (0.095-0.233) | 0.524 (0.316-0.758) | 4.77 (4.4-5.15) |
| Iran | 7.143 (4.1452-11.2156) | 37.8422 (21.3455-62.7296) | 4.3 (4.15-4.59) | 0.093 (0.054-0.146) | 0.312 (0.176-0.518) | 4.33 (4.01-4.66) |
| Iraq | 0.6733 (0.4064-0.9907) | 2.2582 (1.3665-3.3335) | 2.35 (2.36-2.36) | 0.053 (0.032-0.078) | 0.058 (0.035-0.086) | 0.64 (0.46-0.83) |
| Ireland | 0.0127 (0.0075-0.0192) | 0.0322 (0.0155-0.0544) | 1.54 (1.07-1.83) | 0.012 (0.007-0.018) | 0.018 (0.009-0.031) | 0.37 (0.04-0.7) |
| Israel | 1.0217 (0.5747-1.6605) | 1.4133 (0.7352-2.3151) | 0.38 (0.28-0.39) | 0.308 (0.173-0.501) | 0.234 (0.122-0.383) | -1.02 (-1.17--0.87) |
| Italy | 0.0166 (0.0103-0.024) | 0.2029 (0.1241-0.3084) | 11.22 (11.05-11.85) | 0.005 (0.003-0.008) | 0.024 (0.015-0.037) | 6.67 (6.1-7.25) |
| Jamaica | 0.6672 (0.3736-1.0131) | 0.2179 (0.1118-0.3861) | -0.67 (-0.7--0.62) | 0.186 (0.104-0.283) | 0.05 (0.026-0.088) | -6.67 (-8.76--4.53) |
| Japan | 17.9079 (10.0382-28.3781) | 18.524 (9.8193-30.7059) | 0.03 (-0.02-0.08) | 0.057 (0.032-0.09) | 0.044 (0.023-0.072) | -2.44 (-4.02--0.84) |
| Jordan | 1.3292 (0.7035-2.111) | 4.2073 (2.217-6.6687) | 2.17 (2.15-2.16) | 0.052 (0.027-0.082) | 0.132 (0.07-0.21) | 3.52 (3.28-3.76) |
| Kazakhstan | 0.0573 (0.0346-0.0852) | 0.8019 (0.4753-1.1877) | 12.99 (12.74-12.94) | 0.002 (0.001-0.004) | 0.013 (0.008-0.02) | 7.18 (6.5-7.86) |
| Kenya | 0.0367 (0.0222-0.0554) | 0.1658 (0.1002-0.2486) | 3.52 (3.51-3.49) | 0.037 (0.022-0.055) | 0.053 (0.032-0.08) | 2.02 (1.68-2.36) |
| Kiribati | 2.327 (1.3721-3.4671) | 8.4111 (5.0972-12.3411) | 2.61 (2.71-2.56) | 0.261 (0.154-0.389) | 0.279 (0.169-0.41) | 0.86 (0.63-1.08) |
| Kuwait | 0.5483 (0.295-0.8987) | 1.3163 (0.6974-2.2551) | 1.4 (1.36-1.51) | 0.281 (0.151-0.461) | 0.211 (0.112-0.361) | -0.95 (-1.13--0.77) |
| Kyrgyzstan | 2.2319 (1.1496-3.7644) | 20.7197 (11.1773-31.4819) | 8.28 (8.72-7.36) | 0.188 (0.097-0.317) | 1.029 (0.555-1.564) | 6.2 (5.53-6.88) |
| Laos | 0.0262 (0.013-0.0402) | 0.3989 (0.2278-0.6293) | 14.23 (16.52-14.65) | 0.055 (0.027-0.084) | 0.41 (0.234-0.646) | 6.57 (6.1-7.05) |
| Latvia | 0.4184 (0.2561-0.6099) | 1.5181 (0.9086-2.2343) | 2.63 (2.55-2.66) | 0.047 (0.029-0.069) | 0.064 (0.038-0.095) | 1.54 (1.34-1.75) |
| Lebanon | 1.3925 (0.7707-2.1165) | 23.8916 (13.6369-38.23) | 16.16 (16.69-17.06) | 0.205 (0.113-0.311) | 1.82 (1.039-2.913) | 8.47 (7.93-9.01) |
| Lesotho | 0.041 (0.0238-0.0619) | 0.2091 (0.1177-0.3325) | 4.1 (3.95-4.37) | 0.415 (0.242-0.627) | 0.79 (0.445-1.256) | 2.16 (1.76-2.56) |
| Liberia | 0.0252 (0.0148-0.0372) | 0.4596 (0.2761-0.6814) | 17.24 (17.66-17.32) | 0.003 (0.002-0.004) | 0.02 (0.012-0.029) | 7.91 (7.26-8.57) |
| Libya | 0.0026 (0.0016-0.0039) | 0.0052 (0.0031-0.0081) | 1 (0.94-1.08) | 0.071 (0.043-0.107) | 0.061 (0.036-0.096) | -0.41 (-0.72--0.11) |
| Lithuania | 0.0092 (0.0057-0.0134) | 0.205 (0.1269-0.3079) | 21.28 (21.26-21.98) | 0.056 (0.035-0.082) | 0.134 (0.083-0.201) | 3.7 (3.09-4.32) |
| Luxembourg | 1.34 (0.7409-2.0541) | 15.7319 (8.6557-24.8808) | 10.74 (10.68-11.11) | 0.057 (0.031-0.087) | 0.45 (0.248-0.712) | 7.36 (6.87-7.85) |
| Madagascar | 5.0511 (3.0716-7.5582) | 31.4807 (18.392-46.0193) | 5.23 (4.99-5.09) | 0.085 (0.051-0.127) | 0.191 (0.111-0.279) | 3.37 (3.1-3.65) |
| Malawi | 0.0151 (0.0089-0.0224) | 0.2354 (0.1396-0.3456) | 14.59 (14.69-14.43) | 0.002 (0.001-0.003) | 0.01 (0.006-0.015) | 7.35 (6.72-7.98) |
| Malaysia | 0.8098 (0.4359-1.2529) | 12.8445 (7.5691-19.7249) | 14.86 (16.36-14.74) | 0.1 (0.054-0.155) | 0.643 (0.379-0.987) | 7 (6.54-7.46) |
| Maldives | 0.165 (0.1001-0.2429) | 2.2058 (1.2937-3.2793) | 12.37 (11.92-12.5) | 0.294 (0.178-0.433) | 0.307 (0.18-0.457) | 0.07 (-0.03-0.17) |
| Mali | 0.0228 (0.0134-0.0346) | 0.1389 (0.0813-0.2087) | 5.09 (5.07-5.03) | 0.002 (0.001-0.003) | 0.011 (0.007-0.017) | 7.98 (7.32-8.65) |
| Malta | 21.3227 (12.9019-31.9589) | 79.7233 (47.2312-116.3091) | 2.74 (2.66-2.64) | 0.282 (0.17-0.422) | 0.341 (0.202-0.498) | 0.81 (0.69-0.94) |
| Marshall Islands | 0.3368 (0.1761-0.575) | 0.6965 (0.3759-1.1594) | 1.07 (1.13-1.02) | 0.31 (0.162-0.53) | 0.308 (0.166-0.513) | 0.07 (-0.08-0.22) |
| Mauritania | 0.2548 (0.1434-0.3932) | 0.0361 (0.0206-0.0549) | -0.86 (-0.86--0.86) | 0.033 (0.019-0.051) | 0.003 (0.002-0.005) | -8.16 (-10.25--6.03) |
| Mauritius | 0.0857 (0.0517-0.1274) | 0.3984 (0.2407-0.5803) | 3.65 (3.66-3.55) | 0.216 (0.13-0.321) | 0.4 (0.242-0.583) | 2.16 (2.07-2.26) |
| Mexico | 0.0006 (0.0004-0.0009) | 0.0013 (0.0007-0.0019) | 1.17 (0.75-1.11) | 0.054 (0.031-0.08) | 0.07 (0.041-0.106) | 0.65 (0.53-0.77) |
| Moldova | 218.4359 (130.4707-334.5997) | 898.9593 (518.4396-1420.0333) | 3.12 (2.97-3.24) | 0.285 (0.17-0.436) | 0.447 (0.258-0.706) | 1.6 (1.5-1.71) |
| Monaco | 1.1001 (0.6341-1.6673) | 5.0588 (2.7867-7.7585) | 3.6 (3.39-3.65) | 0.163 (0.094-0.247) | 0.559 (0.308-0.858) | 4.66 (4.24-5.07) |
| Mongolia | 0.1797 (0.1027-0.2826) | 2.1686 (1.2733-3.3653) | 11.07 (11.4-10.91) | 0.248 (0.142-0.39) | 1.374 (0.807-2.133) | 6.88 (6.5-7.27) |
| Montenegro | 4.9418 (2.5998-8.1804) | 196.489 (112.135-332.1097) | 38.76 (42.13-39.6) | 0.032 (0.017-0.054) | 0.865 (0.494-1.462) | 12.47 (10.34-14.64) |
| Morocco | 8.7242 (5.0839-12.9359) | 167.7925 (96.3926-258.4585) | 18.23 (17.96-18.98) | 0.266 (0.155-0.394) | 2.265 (1.301-3.489) | 7.29 (6.23-8.36) |
| Mozambique | 4.8297 (2.9027-7.2931) | 18.4835 (11.1662-27.5028) | 2.83 (2.85-2.77) | 0.314 (0.189-0.474) | 0.467 (0.282-0.695) | 1.39 (1.31-1.47) |
| Myanmar | 0.1294 (0.0704-0.2244) | 0.1967 (0.0986-0.3379) | 0.52 (0.4-0.51) | 0.286 (0.156-0.497) | 0.217 (0.109-0.373) | -1.11 (-1.62--0.59) |
| Namibia | 21.0993 (12.5178-33.0343) | 42.908 (25.0123-70.9629) | 1.03 (1-1.15) | 0.231 (0.137-0.362) | 0.216 (0.126-0.358) | -0.24 (-0.29--0.2) |
| Nauru | 0.8982 (0.4594-1.543) | 2.8468 (1.428-4.9975) | 2.17 (2.11-2.24) | 0.144 (0.074-0.248) | 0.147 (0.074-0.258) | 0.21 (0.16-0.26) |
| Nepal | 0.1607 (0.0911-0.2412) | 0.138 (0.0797-0.2116) | -0.14 (-0.13--0.12) | 0.049 (0.028-0.074) | 0.02 (0.011-0.03) | -2.99 (-3.41--2.58) |
| Netherlands | 30.9198 (17.8048-47.338) | 208.1927 (120.2876-309.5207) | 5.73 (5.76-5.54) | 0.147 (0.085-0.226) | 0.661 (0.382-0.982) | 6.89 (6.18-7.61) |
| New Zealand | 3.0891 (1.8874-4.7269) | 67.2354 (37.5809-104.7847) | 20.77 (18.91-21.17) | 0.093 (0.057-0.143) | 1.149 (0.642-1.791) | 9.29 (8.4-10.17) |
| Nicaragua | 0.2615 (0.1363-0.4542) | 0.4505 (0.2286-0.7629) | 0.72 (0.68-0.68) | 0.139 (0.072-0.241) | 0.126 (0.064-0.213) | -0.22 (-0.26--0.19) |
| Niger | 28.675 (17.0803-44.4787) | 55.3626 (32.6991-86.7958) | 0.93 (0.91-0.95) | 0.85 (0.506-1.318) | 0.702 (0.415-1.1) | -0.67 (-0.96--0.38) |
| Nigeria | 3.1263 (1.7984-4.8609) | 1.2479 (0.6615-1.9784) | -0.6 (-0.63--0.59) | 0.16 (0.092-0.249) | 0.027 (0.014-0.043) | -6.06 (-6.28--5.83) |
| Niue | 0.2329 (0.118-0.3968) | 0.5788 (0.2999-1.002) | 1.49 (1.54-1.53) | 0.131 (0.066-0.223) | 0.133 (0.069-0.231) | 0.29 (0.23-0.35) |
| North Korea | 0.0117 (0.007-0.0174) | 0.0222 (0.0134-0.0327) | 0.9 (0.91-0.88) | 0.081 (0.049-0.121) | 0.09 (0.055-0.133) | 0.37 (0.2-0.53) |
| North Macedonia | 3.5328 (1.5064-6.1037) | 13.1081 (6.859-21.815) | 2.71 (3.55-2.57) | 0.326 (0.139-0.564) | 0.808 (0.423-1.345) | 4.31 (3.93-4.68) |
| Northern Mariana Islands | 0.0947 (0.0472-0.1489) | 1.1823 (0.691-1.8225) | 11.48 (13.64-11.24) | 0.101 (0.051-0.159) | 0.665 (0.389-1.026) | 7.02 (5.95-8.09) |
| Norway | 2.1594 (1.3323-3.2103) | 11.3902 (6.6871-17.0186) | 4.27 (4.02-4.3) | 0.111 (0.068-0.165) | 0.385 (0.226-0.576) | 4.41 (4.12-4.69) |
| Oman | 0.0009 (0.0005-0.0014) | 0.0034 (0.002-0.0051) | 2.78 (3-2.64) | 0.042 (0.024-0.064) | 0.072 (0.042-0.109) | 2.29 (2.07-2.51) |
| Pakistan | 2.957 (1.6966-4.4491) | 0.9606 (0.5225-1.5483) | -0.68 (-0.69--0.65) | 0.128 (0.073-0.192) | 0.033 (0.018-0.054) | -5.8 (-6.16--5.43) |
| Palau | 0.1681 (0.0854-0.2905) | 0.2973 (0.1507-0.4943) | 0.77 (0.76-0.7) | 0.172 (0.087-0.297) | 0.166 (0.084-0.277) | 0.17 (0.09-0.25) |
| Palestine | 2.9079 (1.6649-4.3863) | 42.3386 (24.0487-63.1725) | 13.56 (13.44-13.4) | 0.119 (0.068-0.18) | 1.088 (0.618-1.624) | 8.33 (7.58-9.08) |
| Panama | 4.3938 (2.2485-7.4478) | 10.3054 (5.4819-17.74) | 1.35 (1.44-1.38) | 0.166 (0.085-0.282) | 0.171 (0.091-0.294) | 0.12 (-0.04-0.28) |
| Papua New Guinea | 0.0377 (0.0217-0.0563) | 0.1083 (0.0611-0.1667) | 1.87 (1.82-1.96) | 0.011 (0.007-0.017) | 0.018 (0.01-0.028) | 1.75 (1.53-1.97) |
| Paraguay | 0.0551 (0.0296-0.0929) | 0.1496 (0.0785-0.2503) | 1.72 (1.65-1.69) | 0.173 (0.093-0.291) | 0.189 (0.099-0.316) | 0.77 (0.65-0.89) |
| Peru | 0.6751 (0.3503-1.1121) | 1.4095 (0.752-2.2674) | 1.09 (1.15-1.04) | 0.179 (0.093-0.295) | 0.176 (0.094-0.283) | -0.1 (-0.19--0.02) |
| Philippines | 0.5063 (0.2772-0.8327) | 0.3812 (0.2039-0.6598) | -0.25 (-0.26--0.21) | 0.362 (0.198-0.595) | 0.211 (0.113-0.365) | -2.33 (-2.7--1.96) |
| Poland | 0.0621 (0.0321-0.1063) | 0.1576 (0.0863-0.2559) | 1.54 (1.69-1.41) | 0.191 (0.099-0.327) | 0.195 (0.107-0.317) | 0.21 (0.16-0.27) |
| Portugal | 11.6678 (6.7202-17.3308) | 49.0654 (27.8495-74.1729) | 3.21 (3.14-3.28) | 0.21 (0.121-0.311) | 0.526 (0.298-0.795) | 3.24 (2.81-3.68) |
| Puerto Rico | 235.3652 (130.5305-378.6335) | 1256.0041 (721.8935-2103.7233) | 4.34 (4.53-4.56) | 0.449 (0.249-0.722) | 1.253 (0.72-2.099) | 3.43 (3.02-3.84) |
| Qatar | 0.0411 (0.0246-0.0622) | 0.3179 (0.1848-0.4905) | 6.73 (6.51-6.89) | 0.389 (0.232-0.588) | 1.365 (0.794-2.106) | 4.66 (4.28-5.05) |
| Romania | 0.1441 (0.0766-0.2317) | 0.1911 (0.0962-0.3178) | 0.33 (0.26-0.37) | 0.226 (0.12-0.363) | 0.166 (0.084-0.277) | -1.02 (-1.17--0.86) |
| Russia | 1.8138 (0.9746-3.026) | 2.3017 (1.2188-3.7555) | 0.27 (0.25-0.24) | 0.328 (0.176-0.547) | 0.252 (0.133-0.411) | -1.04 (-1.16--0.92) |
| Rwanda | 0.0388 (0.0201-0.064) | 0.1777 (0.0931-0.3011) | 3.58 (3.63-3.7) | 0.181 (0.094-0.298) | 0.171 (0.09-0.29) | -0.16 (-0.33-0.02) |
| Saint Kitts and Nevis | 2.0732 (1.1068-3.3524) | 4.0228 (2.0631-6.5143) | 0.94 (0.86-0.94) | 0.31 (0.166-0.501) | 0.278 (0.143-0.45) | -0.36 (-0.4--0.32) |
| Saint Lucia | 0.4755 (0.2594-0.7769) | 0.9508 (0.498-1.577) | 1 (0.92-1.03) | 0.286 (0.156-0.468) | 0.265 (0.139-0.44) | -0.36 (-0.42--0.3) |
| Saint Vincent and the Grenadines | 0.5607 (0.2962-0.9065) | 0.7854 (0.4146-1.2881) | 0.4 (0.4-0.42) | 0.298 (0.157-0.481) | 0.238 (0.126-0.39) | -0.83 (-0.93--0.72) |
| Samoa | 1.734 (1.0061-2.5855) | 0.3966 (0.2044-0.6613) | -0.77 (-0.8--0.74) | 0.277 (0.161-0.414) | 0.06 (0.031-0.1) | -6.67 (-8.7--4.59) |
| San Marino | 3.4855 (1.9922-5.5496) | 7.9983 (4.4678-12.6815) | 1.29 (1.24-1.29) | 0.262 (0.149-0.416) | 0.214 (0.119-0.339) | -1.41 (-1.66--1.16) |
| São Tomé and Príncipe | 0.0045 (0.0026-0.0067) | 0.0092 (0.0054-0.0136) | 1.04 (1.08-1.03) | 0.076 (0.043-0.113) | 0.073 (0.043-0.108) | -0.15 (-0.22--0.08) |
| Saudi Arabia | 4.1476 (2.344-6.3718) | 25.263 (15.9425-37.2072) | 5.09 (5.8-4.84) | 0.158 (0.089-0.243) | 0.668 (0.422-0.984) | 6.29 (5.87-6.7) |
| Senegal | 0.2141 (0.109-0.3594) | 0.5888 (0.3017-1.001) | 1.75 (1.77-1.79) | 0.121 (0.062-0.203) | 0.134 (0.068-0.227) | 0.31 (0.28-0.34) |
| Serbia | 4.4428 (2.3906-7.1014) | 12.3219 (7.0092-19.49) | 1.77 (1.93-1.74) | 0.138 (0.074-0.221) | 0.18 (0.102-0.285) | 0.94 (0.82-1.06) |
| Seychelles | 1.5934 (0.8396-2.6937) | 2.8902 (1.4887-5.0178) | 0.81 (0.77-0.86) | 0.189 (0.099-0.319) | 0.157 (0.081-0.272) | -0.75 (-0.87--0.64) |
| Sierra Leone | 1.1742 (0.6166-1.9046) | 2.3119 (1.2638-3.9107) | 0.97 (1.05-1.05) | 0.187 (0.098-0.303) | 0.198 (0.108-0.334) | 0.13 (0.06-0.21) |
| Singapore | 0.2399 (0.1416-0.3605) | 4.0912 (2.4163-6.2267) | 16.05 (16.06-16.27) | 0.197 (0.116-0.296) | 1.209 (0.714-1.84) | 6.52 (6.12-6.92) |
| Slovakia | 1.3526 (0.7072-2.2552) | 3.8893 (2.0893-6.4844) | 1.88 (1.95-1.88) | 0.308 (0.161-0.513) | 0.279 (0.15-0.466) | -0.23 (-0.33--0.12) |
| Slovenia | 1.7343 (0.9494-2.8588) | 2.8648 (1.4686-4.7917) | 0.65 (0.55-0.68) | 0.177 (0.097-0.293) | 0.16 (0.082-0.268) | -0.61 (-0.76--0.45) |
| Solomon Islands | 1.3713 (0.7494-2.2548) | 2.6997 (1.3935-4.4868) | 0.97 (0.86-0.99) | 0.259 (0.142-0.427) | 0.209 (0.108-0.347) | -0.62 (-0.74--0.5) |
| Somalia | 1.2097 (0.684-1.8675) | 1.6565 (0.9067-2.6087) | 0.37 (0.33-0.4) | 0.058 (0.033-0.089) | 0.052 (0.029-0.082) | 0.74 (0.07-1.41) |
| South Africa | 0.6103 (0.3344-1.0278) | 1.8144 (0.9444-2.9791) | 1.97 (1.82-1.9) | 0.169 (0.093-0.285) | 0.192 (0.1-0.316) | 0.33 (0.19-0.47) |
| South Korea | 0.362 (0.2132-0.5446) | 1.433 (0.8692-2.0826) | 2.96 (3.08-2.82) | 0.249 (0.147-0.375) | 0.333 (0.202-0.484) | 1.41 (1.23-1.58) |
| South Sudan | 0.6876 (0.3573-1.1494) | 2.1894 (1.1485-3.5351) | 2.18 (2.21-2.08) | 0.147 (0.076-0.246) | 0.207 (0.109-0.334) | 1.35 (1.23-1.46) |
| Spain | 0.5554 (0.2919-0.8835) | 2.7686 (1.567-4.151) | 3.98 (4.37-3.7) | 0.398 (0.209-0.632) | 0.792 (0.448-1.188) | 2.13 (1.91-2.35) |
| Sri Lanka | 0.0057 (0.0034-0.0085) | 0.0173 (0.01-0.0258) | 2.04 (1.94-2.04) | 0.057 (0.034-0.085) | 0.06 (0.035-0.09) | 0.16 (0.11-0.22) |
| Sudan | 3.7994 (2.0054-6.3677) | 8.5164 (4.5384-13.9409) | 1.24 (1.26-1.19) | 0.211 (0.111-0.353) | 0.207 (0.11-0.338) | 0.27 (0.15-0.4) |
| Suriname | 0.0012 (0.0007-0.0017) | 0.0026 (0.0015-0.0038) | 1.17 (1.14-1.24) | 0.012 (0.007-0.018) | 0.012 (0.007-0.018) | 0.24 (0.03-0.45) |
| Sweden | 1.5159 (0.8368-2.3356) | 16.9409 (9.3984-26.5943) | 10.18 (10.23-10.39) | 0.116 (0.064-0.179) | 0.88 (0.488-1.381) | 7.74 (7.36-8.13) |
| Switzerland | 0.0168 (0.0078-0.0272) | 0.0649 (0.0315-0.1066) | 2.86 (3.04-2.92) | 0.305 (0.142-0.493) | 0.478 (0.232-0.786) | 2.17 (1.93-2.4) |
| Syria | 0.0292 (0.0173-0.0447) | 0.1119 (0.0653-0.1713) | 2.83 (2.77-2.83) | 0.226 (0.134-0.347) | 0.297 (0.173-0.454) | 1.19 (0.84-1.55) |
| Taiwan (province of China) | 0.0565 (0.0341-0.084) | 0.1525 (0.0921-0.2272) | 1.7 (1.7-1.7) | 0.51 (0.308-0.758) | 0.975 (0.589-1.452) | 2.13 (2.03-2.24) |
| Tajikistan | 2.3673 (1.2902-3.9237) | 5.0586 (2.7147-8.5337) | 1.14 (1.1-1.17) | 0.226 (0.123-0.375) | 0.213 (0.114-0.36) | 0.06 (-0.05-0.18) |
| Tanzania | 0.021 (0.0123-0.0312) | 0.0943 (0.0563-0.1474) | 3.49 (3.58-3.72) | 0.15 (0.088-0.224) | 0.293 (0.175-0.458) | 2.14 (2.07-2.21) |
| Thailand | 68.9869 (41.8301-102.9161) | 480.4905 (299.5003-720.3024) | 5.96 (6.16-6) | 0.496 (0.301-0.74) | 2.173 (1.355-3.258) | 5.29 (4.64-5.94) |
| The Bahamas | 1.4467 (0.8832-2.1244) | 5.3479 (3.1796-7.8453) | 2.7 (2.6-2.69) | 0.021 (0.013-0.03) | 0.031 (0.018-0.045) | 1.36 (1.08-1.64) |
| The Gambia | 0.2998 (0.1584-0.502) | 0.7859 (0.4291-1.3094) | 1.62 (1.71-1.61) | 0.322 (0.17-0.54) | 0.324 (0.177-0.54) | -0.13 (-0.24--0.02) |
| Timor-Leste | 0.0107 (0.0062-0.0156) | 0.0194 (0.0114-0.029) | 0.81 (0.84-0.86) | 0.112 (0.066-0.165) | 0.145 (0.085-0.216) | 1 (0.91-1.09) |
| Togo | 0.0003 (0.0002-0.0005) | 0.0005 (0.0003-0.0007) | 0.67 (0.5-0.4) | 0.044 (0.026-0.067) | 0.052 (0.031-0.079) | 0.53 (0.39-0.66) |
| Tokelau | 0.0004 (0.0002-0.0006) | 0.0004 (0.0002-0.0005) | 0 (0--0.17) | 0.109 (0.065-0.16) | 0.093 (0.053-0.14) | -0.06 (-0.24-0.13) |
| Tonga | 1.3139 (0.7537-1.9313) | 11.3234 (6.3911-17.2314) | 7.62 (7.48-7.92) | 0.049 (0.028-0.072) | 0.308 (0.174-0.469) | 5.55 (5.08-6.02) |
| Trinidad and Tobago | 1.1926 (0.6419-2.0083) | 1.5055 (0.7868-2.521) | 0.26 (0.23-0.26) | 0.18 (0.097-0.303) | 0.136 (0.071-0.228) | -1.41 (-1.79--1.03) |
| Tunisia | 1.0081 (0.5616-1.6289) | 1.9519 (1.0494-3.1908) | 0.94 (0.87-0.96) | 0.315 (0.176-0.509) | 0.236 (0.127-0.386) | -0.92 (-0.99--0.86) |
| Türkiye | 2.0322 (1.0925-3.3876) | 3.4538 (1.8661-5.5959) | 0.7 (0.71-0.65) | 0.281 (0.151-0.468) | 0.23 (0.124-0.372) | -0.77 (-0.84--0.7) |
| Turkmenistan | 0.0054 (0.0032-0.0082) | 0.0162 (0.0096-0.0248) | 2 (2-2.02) | 0.222 (0.131-0.337) | 0.167 (0.099-0.255) | -1.82 (-2.27--1.37) |
| Tuvalu | 0.0015 (0.0009-0.0023) | 0.0032 (0.0019-0.0049) | 1.13 (1.11-1.13) | 0.093 (0.054-0.141) | 0.078 (0.045-0.116) | -1.02 (-1.19--0.86) |
| Uganda | 2.2494 (1.1972-3.6136) | 4.8004 (2.5409-7.9586) | 1.13 (1.12-1.2) | 0.304 (0.162-0.488) | 0.236 (0.125-0.391) | -0.92 (-1.1--0.74) |
| UK | 0.0002 (0.0001-0.0003) | 0.0003 (0.0002-0.0004) | 0.5 (1-0.33) | 0.08 (0.047-0.119) | 0.115 (0.067-0.174) | 1.34 (1.22-1.45) |
| Ukraine | 0.0061 (0.0036-0.0093) | 0.0196 (0.0116-0.0294) | 2.21 (2.22-2.16) | 0.096 (0.056-0.145) | 0.152 (0.09-0.228) | 1.74 (1.26-2.23) |
| United Arab Emirates | 1.8578 (1.0947-2.8259) | 12.3187 (7.3738-19.2426) | 5.63 (5.74-5.81) | 0.306 (0.18-0.466) | 1.055 (0.632-1.648) | 5.1 (4.7-5.51) |
| Uruguay | 1.0772 (0.5817-1.7001) | 1.4378 (0.8121-2.1917) | 0.33 (0.4-0.29) | 0.23 (0.124-0.363) | 0.14 (0.079-0.213) | -1.17 (-1.43--0.91) |
| United States | 0.8166 (0.428-1.3862) | 0.9682 (0.517-1.5806) | 0.19 (0.21-0.14) | 0.197 (0.103-0.334) | 0.155 (0.083-0.254) | -0.79 (-0.95--0.63) |
| Uzbekistan | 0.112 (0.0617-0.1824) | 0.1837 (0.0993-0.3037) | 0.64 (0.61-0.67) | 0.284 (0.156-0.462) | 0.24 (0.13-0.396) | -0.26 (-0.39--0.13) |
| Vanuatu | 1.588 (0.8893-2.5813) | 2.2901 (1.1824-3.8462) | 0.44 (0.33-0.49) | 0.343 (0.192-0.557) | 0.247 (0.127-0.414) | -1.02 (-1.14--0.89) |
| Venezuela | 1.4071 (0.8125-2.2024) | 36.1041 (21.646-55.8141) | 24.66 (25.64-24.34) | 0.051 (0.029-0.079) | 0.479 (0.287-0.741) | 6.93 (6.05-7.82) |
| Viet Nam | 0.0357 (0.0188-0.0587) | 0.043 (0.0228-0.0696) | 0.2 (0.21-0.19) | 0.319 (0.168-0.524) | 0.235 (0.125-0.381) | -0.99 (-1.02--0.97) |
| Virgin Islands | 0.0399 (0.0241-0.0606) | 0.4067 (0.2452-0.5936) | 9.19 (9.17-8.8) | 0.003 (0.002-0.004) | 0.013 (0.008-0.019) | 6.89 (6.24-7.54) |
| Yemen | 1.6024 (0.8094-2.6479) | 3.8486 (1.9522-6.5462) | 1.4 (1.41-1.47) | 0.25 (0.126-0.413) | 0.215 (0.109-0.365) | -0.38 (-0.53--0.23) |
| Zambia | 0.1783 (0.0959-0.2919) | 0.355 (0.1891-0.5821) | 0.99 (0.97-0.99) | 0.322 (0.173-0.527) | 0.23 (0.122-0.376) | -1.02 (-1.08--0.96) |
| Zimbabwe | 3.5972 (2.0165-5.7372) | 9.015 (4.9713-14.7437) | 1.51 (1.47-1.57) | 0.352 (0.197-0.562) | 0.331 (0.182-0.541) | -0.41 (-0.66--0.16) |

**Table S14. Prevalent cases, prevalence rates, and EAPC trends of HF impairment under the PC cause category across 204 countries and territories from 1990 to 2021 among adults aged ≥55 years.**

| **Location** | **Prevalent Cases** | |  | **Prevalent Rates** | |  |
| --- | --- | --- | --- | --- | --- | --- |
|  | **1990 (95% UI)** | **2021 (95% UI)** | **Relative change, 1990–2021(95% UI)** | **1990, per 100,000 (95% UI)** | **2021, per 100,000 (95% UI)** | **EAPC (95% CI)** |
| Afghanistan | 0.09897 (0.06698-0.13672) | 0.31765 (0.20862-0.44266) | 2.21 (2.11-2.24) | 0.012 (0.008-0.017) | 0.033 (0.022-0.046) | 3.24 (1.63-4.88) |
| Albania | 35.68824 (24.89998-47.21793) | 121.7703 (87.61902-164.52985) | 2.41 (2.52-2.48) | 0.654 (0.456-0.865) | 0.992 (0.714-1.341) | 1.59 (1.2-1.98) |
| Algeria | 122.21446 (87.11058-166.08543) | 157.55551 (130.20158-190.09671) | 0.29 (0.49-0.14) | 1.278 (0.911-1.737) | 1.018 (0.841-1.228) | -0.91 (-1.19--0.63) |
| American Samoa | 1.07335 (0.79309-1.43448) | 1.56557 (1.10552-2.20424) | 0.46 (0.39-0.54) | 0.022 (0.017-0.03) | 0.011 (0.008-0.016) | -2.96 (-3.39--2.54) |
| Andorra | 27.27636 (19.06799-37.31748) | 71.18374 (50.58898-94.5654) | 1.61 (1.65-1.53) | 1.622 (1.134-2.219) | 1.59 (1.13-2.112) | -0.62 (-0.8--0.45) |
| Angola | 0.00022 (0.00016-0.00028) | 0.00004 (0.00003-0.00006) | -0.82 (-0.81--0.79) | 0.003 (0.002-0.003) | 0 (0-0) | -7.86 (-8.57--7.15) |
| Antigua and Barbuda | 0.07631 (0.05794-0.1006) | 0.30183 (0.23061-0.39309) | 2.96 (2.98-2.91) | 0.01 (0.008-0.014) | 0.014 (0.011-0.018) | 0.94 (0.72-1.15) |
| Argentina | 0.68948 (0.51567-0.89153) | 1.44556 (1.09049-1.8899) | 1.1 (1.11-1.12) | 0.039 (0.029-0.05) | 0.03 (0.023-0.039) | -1.6 (-2.08--1.12) |
| Armenia | 0.01587 (0.0118-0.0212) | 0.02486 (0.01832-0.03317) | 0.57 (0.55-0.56) | 0.065 (0.048-0.086) | 0.035 (0.025-0.046) | -2.42 (-2.93--1.91) |
| Australia | 1.87763 (1.37977-2.51272) | 4.82366 (3.25209-7.12692) | 1.57 (1.36-1.84) | 0.012 (0.009-0.016) | 0.012 (0.008-0.017) | -0.33 (-0.56--0.1) |
| Austria | 0.85602 (0.63638-1.1255) | 0.14364 (0.10938-0.18702) | -0.83 (-0.83--0.83) | 0.051 (0.038-0.066) | 0.004 (0.003-0.005) | -7.39 (-8.76--6) |
| Azerbaijan | 0.01303 (0.00969-0.01734) | 0.01906 (0.01408-0.02532) | 0.46 (0.45-0.46) | 0.028 (0.021-0.037) | 0.021 (0.015-0.028) | -2.01 (-2.58--1.45) |
| Bahrain | 0.00136 (0.00064-0.00206) | 0.00623 (0.00295-0.00946) | 3.58 (3.61-3.59) | 0.009 (0.004-0.013) | 0.01 (0.005-0.016) | -0.2 (-0.54-0.13) |
| Bangladesh | 67.78009 (42.62743-97.00637) | 174.94346 (111.4067-253.22242) | 1.58 (1.61-1.61) | 0.456 (0.287-0.653) | 0.832 (0.53-1.204) | 1.89 (1.73-2.05) |
| Barbados | 9.12905 (6.78589-12.34387) | 22.82205 (16.81986-30.57997) | 1.5 (1.48-1.48) | 0.343 (0.255-0.464) | 0.405 (0.298-0.543) | 0.79 (0.66-0.92) |
| Belarus | 0.00097 (0.00072-0.00129) | 0.00158 (0.00113-0.00213) | 0.63 (0.57-0.65) | 0.01 (0.007-0.013) | 0.01 (0.007-0.014) | 0.02 (-0.22-0.26) |
| Belgium | 56.03686 (36.84099-81.0746) | 14.63918 (8.72431-23.67559) | -0.74 (-0.76--0.71) | 0.449 (0.296-0.65) | 0.108 (0.064-0.174) | -6.36 (-7.21--5.51) |
| Belize | 0.03209 (0.02431-0.04203) | 0.08921 (0.06762-0.11904) | 1.78 (1.78-1.83) | 0.009 (0.007-0.012) | 0.012 (0.009-0.015) | 0.61 (0.4-0.83) |
| Benin | 0.09208 (0.07059-0.11963) | 0.11727 (0.08768-0.15294) | 0.27 (0.24-0.28) | 0.598 (0.459-0.777) | 0.234 (0.175-0.305) | -3.46 (-3.91--3.01) |
| Bermuda | 0.05876 (0.04377-0.07663) | 0.3389 (0.25824-0.44729) | 4.77 (4.9-4.84) | 0.004 (0.003-0.005) | 0.007 (0.005-0.009) | 2.57 (1.88-3.26) |
| Bhutan | 272.40764 (164.37353-388.53126) | 562.30561 (378.64532-794.61538) | 1.06 (1.3-1.05) | 0.92 (0.555-1.312) | 1.077 (0.725-1.522) | -0.35 (-0.68--0.02) |
| Bolivia | 7.51139 (4.18972-11.18489) | 10.48322 (7.23694-14.72734) | 0.4 (0.73-0.32) | 0.445 (0.248-0.662) | 0.358 (0.247-0.502) | -0.07 (-0.7-0.57) |
| Bosnia and Herzegovina | 0.02433 (0.01888-0.0312) | 0.09586 (0.074-0.12407) | 2.94 (2.92-2.98) | 0.159 (0.123-0.204) | 0.176 (0.136-0.228) | 0.86 (0.45-1.27) |
| Botswana | 0.21347 (0.16138-0.28093) | 0.82302 (0.60926-1.1065) | 2.86 (2.78-2.94) | 0.035 (0.026-0.046) | 0.049 (0.036-0.066) | -1.5 (-2.37--0.62) |
| Brazil | 0.08257 (0.05396-0.12394) | 0.67423 (0.28284-1.11722) | 7.17 (4.24-8.01) | 0.007 (0.005-0.011) | 0.064 (0.027-0.106) | 9.8 (6.97-12.7) |
| Brunei | 0.71741 (0.55783-0.94076) | 2.02063 (1.51762-2.6233) | 1.82 (1.72-1.79) | 0.018 (0.014-0.024) | 0.024 (0.018-0.031) | 0.83 (0.71-0.95) |
| Bulgaria | 0.05369 (0.04032-0.07202) | 0.0169 (0.01192-0.02262) | -0.69 (-0.7--0.69) | 0.445 (0.334-0.597) | 0.084 (0.059-0.113) | -5.92 (-6.48--5.36) |
| Burkina Faso | 2.70242 (2.05947-3.54239) | 8.21055 (6.24311-10.58866) | 2.04 (2.03-1.99) | 0.046 (0.035-0.06) | 0.042 (0.032-0.054) | -0.27 (-0.37--0.18) |
| Burundi | 0.23998 (0.17505-0.32512) | 0.48108 (0.35614-0.6335) | 1 (1.03-0.95) | 0.044 (0.032-0.06) | 0.041 (0.03-0.053) | -0.48 (-0.72--0.23) |
| Cabo Verde | 0.00516 (0.00373-0.00721) | 0.01961 (0.01413-0.02635) | 2.8 (2.79-2.65) | 0.008 (0.006-0.012) | 0.017 (0.013-0.023) | -0.75 (-1.92-0.42) |
| Cambodia | 67.05932 (42.48776-95.58796) | 346.70753 (266.98672-442.83875) | 4.17 (5.28-3.63) | 1.347 (0.854-1.921) | 2.073 (1.596-2.648) | 1.61 (1.22-2) |
| Cameroon | 0.02183 (0.01566-0.02917) | 0.0089 (0.00663-0.0119) | -0.59 (-0.58--0.59) | 0.149 (0.107-0.199) | 0.021 (0.016-0.028) | -8.25 (-9.15--7.34) |
| Canada | 0.00037 (0.00027-0.00051) | 0.00103 (0.00074-0.00145) | 1.78 (1.74-1.84) | 0.014 (0.011-0.02) | 0.018 (0.013-0.025) | 0.24 (-0.04-0.51) |
| Central African Republic | 0.00485 (0.00375-0.0062) | 0.02077 (0.01573-0.02738) | 3.28 (3.19-3.42) | 0.011 (0.009-0.014) | 0.015 (0.011-0.019) | 0.83 (0.7-0.96) |
| Chad | 1.66607 (1.28168-2.1928) | 1.32988 (1.03216-1.67246) | -0.2 (-0.19--0.24) | 0.566 (0.435-0.745) | 0.251 (0.195-0.316) | -4.14 (-4.85--3.41) |
| Chile | 0.00147 (0.00102-0.00209) | 0.00117 (0.00085-0.00156) | -0.2 (-0.17--0.25) | 0.012 (0.008-0.017) | 0.005 (0.003-0.006) | -4.83 (-7.39--2.19) |
| China | 16.08326 (11.72727-21.30046) | 17.58039 (12.14687-25.45788) | 0.09 (0.04-0.2) | 0.677 (0.494-0.897) | 0.522 (0.361-0.756) | -0.8 (-1.07--0.52) |
| Colombia | 0.11544 (0.08694-0.1489) | 0.17358 (0.13197-0.22528) | 0.5 (0.52-0.51) | 0.265 (0.2-0.342) | 0.155 (0.118-0.202) | -2.71 (-3.07--2.35) |
| Comoros | 0.07966 (0.05698-0.10787) | 0.16844 (0.12272-0.22737) | 1.11 (1.15-1.11) | 1.356 (0.97-1.837) | 1.424 (1.038-1.922) | -0.12 (-0.36-0.12) |
| Congo (Brazzaville) | 3.96446 (2.41301-5.78759) | 6.2199 (3.8815-9.11735) | 0.57 (0.61-0.58) | 0.861 (0.524-1.257) | 0.576 (0.359-0.844) | -1.57 (-1.72--1.41) |
| Cook Islands | 0.08962 (0.06708-0.11812) | 0.52268 (0.38932-0.69257) | 4.83 (4.8-4.86) | 0.03 (0.023-0.04) | 0.064 (0.047-0.084) | 2.46 (2.19-2.73) |
| Costa Rica | 0.04427 (0.03291-0.05934) | 0.08668 (0.06508-0.11512) | 0.96 (0.98-0.94) | 0.032 (0.024-0.043) | 0.025 (0.019-0.033) | -1.77 (-2.39--1.15) |
| Côte d'Ivoire | 16.09054 (12.10404-21.19998) | 54.54809 (40.85886-71.04834) | 2.39 (2.38-2.35) | 0.559 (0.42-0.736) | 0.57 (0.427-0.743) | -0.24 (-0.75-0.28) |
| Croatia | 1.88887 (1.41018-2.51868) | 6.08031 (4.42661-8.10738) | 2.22 (2.14-2.22) | 0.36 (0.269-0.48) | 0.393 (0.286-0.525) | 0.62 (0.48-0.76) |
| Cuba | 20.59543 (15.86389-26.43706) | 83.28767 (65.98275-103.61303) | 3.04 (3.16-2.92) | 1.045 (0.805-1.341) | 1.489 (1.18-1.852) | 1.79 (1.33-2.25) |
| Cyprus | 1.4531 (1.09538-1.86743) | 4.55729 (3.38191-6.08544) | 2.14 (2.09-2.26) | 0.169 (0.127-0.217) | 0.165 (0.122-0.22) | 0.33 (-0.16-0.83) |
| Czechia | 0.00172 (0.00127-0.00235) | 0.0106 (0.00742-0.01497) | 5.16 (4.84-5.37) | 0.008 (0.006-0.01) | 0.02 (0.014-0.028) | 3.6 (3.33-3.86) |
| Denmark | 0.0905 (0.05194-0.13273) | 0.55659 (0.39388-0.75037) | 5.15 (6.58-4.65) | 0.025 (0.014-0.037) | 0.037 (0.026-0.049) | 0.69 (0.46-0.92) |
| Djibouti | 0.30421 (0.23238-0.38856) | 1.29954 (0.96225-1.69329) | 3.27 (3.14-3.36) | 0.107 (0.082-0.137) | 0.136 (0.1-0.177) | 0.12 (-0.16-0.4) |
| Dominica | 0.0013 (0.00097-0.00174) | 0.00316 (0.0023-0.00441) | 1.43 (1.37-1.53) | 0.016 (0.012-0.022) | 0.024 (0.017-0.034) | 1.01 (0.75-1.28) |
| Dominican Republic | 0.19566 (0.09387-0.3048) | 0.53191 (0.25025-0.82117) | 1.72 (1.67-1.69) | 0.113 (0.054-0.176) | 0.135 (0.063-0.208) | 0.72 (0.54-0.91) |
| DR Congo | 0.00157 (0.00118-0.00212) | 0.00259 (0.00181-0.0037) | 0.65 (0.53-0.75) | 0.003 (0.002-0.004) | 0.002 (0.001-0.003) | -1.36 (-1.66--1.05) |
| Ecuador | 0.10789 (0.08318-0.14063) | 0.17611 (0.13313-0.22953) | 0.63 (0.6-0.63) | 0.022 (0.017-0.029) | 0.017 (0.013-0.022) | -1.12 (-1.34--0.89) |
| Egypt | 1559.16103 (1104.02093-2170.21961) | 4976.14883 (3445.3329-7240.80544) | 2.19 (2.12-2.34) | 1.086 (0.769-1.512) | 1.313 (0.909-1.911) | 0.4 (0.23-0.57) |
| El Salvador | 1.78304 (1.33803-2.37182) | 3.90698 (2.88367-5.16615) | 1.19 (1.16-1.18) | 0.31 (0.233-0.412) | 0.213 (0.158-0.282) | -2.18 (-2.66--1.69) |
| Equatorial Guinea | 70.46784 (51.47588-96.00424) | 57.83862 (39.86912-83.48413) | -0.18 (-0.23--0.13) | 1.014 (0.74-1.381) | 0.268 (0.185-0.387) | -4.93 (-5.15--4.7) |
| Eritrea | 29.72481 (20.28845-41.30661) | 49.66434 (34.81263-67.73746) | 0.67 (0.72-0.64) | 0.408 (0.278-0.567) | 0.331 (0.232-0.452) | -0.7 (-0.89--0.51) |
| Estonia | 0.49318 (0.37765-0.65247) | 1.24277 (0.92636-1.61638) | 1.52 (1.45-1.48) | 0.198 (0.152-0.263) | 0.153 (0.114-0.199) | -1.27 (-1.53--1.02) |
| Eswatini | 1.05856 (0.64585-1.58596) | 16.75633 (10.93881-23.84514) | 14.83 (15.94-14.04) | 0.161 (0.098-0.241) | 1.175 (0.767-1.672) | 7.57 (7.07-8.06) |
| Ethiopia | 0.48439 (0.33187-0.65668) | 0.464 (0.3377-0.60641) | -0.04 (0.02--0.08) | 0.1 (0.068-0.135) | 0.059 (0.043-0.077) | -1.57 (-2.03--1.11) |
| Federated States of Micronesia | 10.50907 (7.1374-14.6995) | 2.71051 (1.79485-3.78223) | -0.74 (-0.75--0.74) | 0.464 (0.315-0.649) | 0.114 (0.076-0.159) | -5.4 (-5.81--4.99) |
| Fiji | 0.85644 (0.64932-1.11226) | 1.7024 (1.2071-2.28867) | 0.99 (0.86-1.06) | 0.257 (0.195-0.334) | 0.162 (0.115-0.218) | -1.59 (-1.66--1.51) |
| Finland | 0.18371 (0.13614-0.24791) | 0.56293 (0.42515-0.73553) | 2.06 (2.12-1.97) | 0.076 (0.056-0.102) | 0.076 (0.057-0.099) | -0.61 (-0.94--0.27) |
| France | 1.69912 (1.10229-2.40034) | 1.4998 (0.89915-2.11732) | -0.12 (-0.18--0.12) | 0.153 (0.099-0.216) | 0.101 (0.06-0.142) | -3 (-3.66--2.33) |
| Gabon | 0.20098 (0.13867-0.276) | 0.39247 (0.27012-0.53604) | 0.95 (0.95-0.94) | 0.039 (0.027-0.054) | 0.046 (0.032-0.063) | 1.51 (1.19-1.84) |
| Georgia | 2.37901 (1.62131-3.25979) | 3.8149 (2.63507-5.30522) | 0.6 (0.63-0.63) | 0.686 (0.467-0.939) | 0.485 (0.335-0.675) | -1.54 (-1.68--1.39) |
| Germany | 0.1706 (0.11858-0.23069) | 0.4391 (0.27955-0.6178) | 1.57 (1.36-1.68) | 0.02 (0.014-0.026) | 0.023 (0.015-0.032) | 0.36 (-0.42-1.15) |
| Ghana | 1.22167 (0.92605-1.60413) | 3.17065 (2.38299-4.1122) | 1.6 (1.57-1.56) | 0.078 (0.059-0.102) | 0.061 (0.046-0.079) | -1.57 (-1.86--1.27) |
| Greece | 2.76855 (1.95701-3.69689) | 2.79255 (1.97157-3.81933) | 0.01 (0.01-0.03) | 0.267 (0.189-0.356) | 0.17 (0.12-0.233) | -2.32 (-2.72--1.92) |
| Greenland | 13.18056 (7.97394-19.89946) | 6.70054 (4.04858-9.45641) | -0.49 (-0.49--0.52) | 0.263 (0.159-0.398) | 0.112 (0.067-0.157) | -3.23 (-3.64--2.82) |
| Grenada | 1.11197 (0.7517-1.50878) | 1.48167 (0.95602-2.06894) | 0.33 (0.27-0.37) | 0.152 (0.102-0.206) | 0.135 (0.087-0.189) | -0.34 (-0.65--0.03) |
| Guam | 0.89815 (0.54781-1.29257) | 1.00074 (0.58951-1.41717) | 0.11 (0.08-0.1) | 0.043 (0.026-0.062) | 0.036 (0.021-0.051) | -2.39 (-3.03--1.75) |
| Guatemala | 28.5652 (20.80092-38.86692) | 188.41595 (131.81711-267.87004) | 5.6 (5.34-5.89) | 0.193 (0.141-0.263) | 0.435 (0.304-0.619) | 2.65 (2.19-3.12) |
| Guinea | 0.27989 (0.20675-0.36426) | 2.6551 (2.00014-3.49222) | 8.49 (8.67-8.59) | 0.013 (0.01-0.017) | 0.044 (0.033-0.058) | 4.83 (4.42-5.24) |
| Guinea-Bissau | 1.56119 (1.16134-2.00512) | 4.19357 (3.09478-5.46204) | 1.69 (1.66-1.72) | 0.43 (0.32-0.553) | 0.424 (0.313-0.552) | -0.1 (-0.42-0.23) |
| Guyana | 0.01585 (0.01195-0.02072) | 0.30998 (0.23611-0.40443) | 18.56 (18.76-18.52) | 0.056 (0.043-0.074) | 0.191 (0.145-0.249) | 5.33 (4.6-6.06) |
| Haiti | 15.39387 (11.92014-19.89844) | 25.44358 (19.47831-33.06437) | 0.65 (0.63-0.66) | 0.344 (0.267-0.445) | 0.23 (0.176-0.299) | -1.49 (-1.6--1.37) |
| Honduras | 0.03512 (0.02696-0.04555) | 0.61961 (0.46701-0.81432) | 16.64 (16.32-16.88) | 0.016 (0.013-0.021) | 0.049 (0.037-0.065) | 4.78 (4.31-5.25) |
| Hungary | 1.35203 (0.77274-1.97033) | 2.21685 (1.39548-3.08959) | 0.64 (0.81-0.57) | 0.314 (0.179-0.457) | 0.303 (0.191-0.422) | -0.54 (-0.74--0.34) |
| Iceland | 1.3984 (1.01559-1.92261) | 15.43218 (10.75329-21.44883) | 10.04 (9.59-10.16) | 0.03 (0.022-0.042) | 0.119 (0.083-0.165) | 5.98 (5.41-6.56) |
| India | 0.01503 (0.01165-0.01942) | 0.08236 (0.06253-0.10809) | 4.48 (4.37-4.57) | 0.016 (0.013-0.021) | 0.018 (0.013-0.023) | 1.93 (0.71-3.16) |
| Indonesia | 0.75156 (0.56735-0.96262) | 4.58727 (3.60509-5.86258) | 5.1 (5.35-5.09) | 0.198 (0.15-0.254) | 0.468 (0.368-0.598) | 3.52 (3.13-3.91) |
| Iran | 40.74754 (28.57329-56.90117) | 26.08532 (18.03629-36.66173) | -0.36 (-0.37--0.36) | 0.529 (0.371-0.738) | 0.215 (0.149-0.303) | -5.12 (-6.29--3.94) |
| Iraq | 1.63752 (1.2605-2.08971) | 4.29445 (3.27899-5.65732) | 1.62 (1.6-1.71) | 0.13 (0.1-0.166) | 0.111 (0.084-0.146) | -0.52 (-0.59--0.45) |
| Ireland | 0.25003 (0.18054-0.33399) | 0.48646 (0.28067-0.7717) | 0.95 (0.55-1.31) | 0.23 (0.166-0.307) | 0.279 (0.161-0.443) | -1.61 (-2.36--0.86) |
| Israel | 1.49988 (1.00136-2.11486) | 2.32409 (1.56082-3.25539) | 0.55 (0.56-0.54) | 0.453 (0.302-0.638) | 0.385 (0.258-0.539) | -0.51 (-0.63--0.39) |
| Italy | 0.07027 (0.05492-0.0902) | 0.42531 (0.32363-0.5453) | 5.05 (4.89-5.05) | 0.023 (0.018-0.029) | 0.051 (0.039-0.065) | 3.58 (3.28-3.88) |
| Jamaica | 0.03147 (0.02117-0.04387) | 0.13647 (0.08209-0.21226) | 3.34 (2.88-3.84) | 0.009 (0.006-0.012) | 0.031 (0.019-0.049) | 2.95 (0.96-4.98) |
| Japan | 8.32094 (5.53266-11.54653) | 44.84569 (28.9975-66.329) | 4.39 (4.24-4.74) | 0.026 (0.018-0.037) | 0.106 (0.068-0.156) | 4.6 (2.53-6.72) |
| Jordan | 8.07361 (4.84734-11.63542) | 4.80579 (2.91387-6.9461) | -0.4 (-0.4--0.4) | 0.313 (0.188-0.452) | 0.151 (0.092-0.218) | -3.01 (-3.28--2.75) |
| Kazakhstan | 0.30351 (0.22748-0.39334) | 1.93705 (1.4634-2.57) | 5.38 (5.43-5.53) | 0.013 (0.01-0.017) | 0.032 (0.024-0.043) | 3.8 (3.43-4.17) |
| Kenya | 0.00318 (0.0023-0.00447) | 0.00983 (0.00737-0.01304) | 2.09 (2.2-1.92) | 0.003 (0.002-0.004) | 0.003 (0.002-0.004) | 0.61 (0.38-0.84) |
| Kiribati | 0.03682 (0.02831-0.04732) | 0.22418 (0.16834-0.28917) | 5.09 (4.95-5.11) | 0.004 (0.003-0.005) | 0.007 (0.006-0.01) | 3.48 (2.81-4.16) |
| Kuwait | 0.83421 (0.54825-1.15731) | 2.23325 (1.5446-3.13684) | 1.68 (1.82-1.71) | 0.428 (0.281-0.593) | 0.357 (0.247-0.502) | -0.61 (-0.73--0.49) |
| Kyrgyzstan | 5.41792 (2.98916-7.71717) | 15.40878 (10.02259-22.34344) | 1.84 (2.35-1.9) | 0.456 (0.252-0.65) | 0.766 (0.498-1.11) | 1.48 (0.94-2.03) |
| Laos | 0.03068 (0.0173-0.04424) | 0.06421 (0.04233-0.09089) | 1.09 (1.45-1.05) | 0.064 (0.036-0.093) | 0.066 (0.043-0.093) | -1.15 (-1.62--0.68) |
| Latvia | 0.13279 (0.10084-0.17087) | 1.01991 (0.75434-1.38638) | 6.68 (6.48-7.11) | 0.015 (0.011-0.019) | 0.043 (0.032-0.059) | 4.42 (4-4.83) |
| Lebanon | 1.00362 (0.65433-1.40162) | 2.29888 (1.62875-3.15916) | 1.29 (1.49-1.25) | 0.147 (0.096-0.206) | 0.175 (0.124-0.241) | 0.63 (0.51-0.74) |
| Lesotho | 0.09992 (0.06936-0.13699) | 0.2789 (0.20549-0.36775) | 1.79 (1.96-1.68) | 1.013 (0.703-1.388) | 1.054 (0.777-1.39) | -0.24 (-0.52-0.04) |
| Liberia | 0.14702 (0.11001-0.19497) | 1.25273 (0.95389-1.61138) | 7.52 (7.67-7.26) | 0.017 (0.013-0.022) | 0.054 (0.041-0.069) | 4.5 (4.07-4.93) |
| Libya | 0.00065 (0.00049-0.00086) | 0.00306 (0.00219-0.00419) | 3.71 (3.47-3.87) | 0.018 (0.013-0.024) | 0.036 (0.026-0.05) | 2.24 (2-2.48) |
| Lithuania | 0.00558 (0.00423-0.00709) | 0.10861 (0.07949-0.14239) | 18.46 (17.79-19.08) | 0.034 (0.026-0.043) | 0.071 (0.052-0.093) | 2.83 (2.31-3.34) |
| Luxembourg | 29.64499 (18.90774-43.12001) | 20.91064 (13.67678-30.65578) | -0.29 (-0.28--0.29) | 1.252 (0.799-1.821) | 0.599 (0.391-0.878) | -3.28 (-3.66--2.9) |
| Madagascar | 8.52953 (6.48206-11.16524) | 25.38032 (19.38707-33.1297) | 1.98 (1.99-1.97) | 0.143 (0.108-0.187) | 0.154 (0.117-0.201) | 0.81 (0.48-1.15) |
| Malawi | 0.09873 (0.07465-0.12833) | 0.7738 (0.57954-1.00143) | 6.84 (6.76-6.8) | 0.012 (0.009-0.016) | 0.034 (0.026-0.045) | 4.27 (3.93-4.61) |
| Malaysia | 0.81202 (0.52292-1.14772) | 2.31762 (1.61496-3.21361) | 1.85 (2.09-1.8) | 0.1 (0.065-0.142) | 0.116 (0.081-0.161) | 0.13 (-0.08-0.35) |
| Maldives | 0.01297 (0.00991-0.01671) | 0.33963 (0.24449-0.4514) | 25.19 (23.67-26.01) | 0.023 (0.018-0.03) | 0.047 (0.034-0.063) | 2.55 (2.36-2.74) |
| Mali | 0.14331 (0.10632-0.18705) | 0.40598 (0.29915-0.53919) | 1.83 (1.81-1.88) | 0.012 (0.009-0.015) | 0.033 (0.024-0.044) | 4.47 (4.08-4.85) |
| Malta | 20.28079 (15.22384-26.80272) | 42.49099 (31.68407-56.67848) | 1.1 (1.08-1.11) | 0.268 (0.201-0.354) | 0.182 (0.136-0.243) | -1.17 (-1.27--1.07) |
| Marshall Islands | 1.25196 (0.76532-1.79514) | 1.94431 (1.23318-2.7033) | 0.55 (0.61-0.51) | 1.154 (0.705-1.654) | 0.861 (0.546-1.196) | -1.06 (-1.1--1.02) |
| Mauritania | 0.0244 (0.01604-0.03376) | 0.0481 (0.03344-0.06703) | 0.97 (1.08-0.99) | 0.003 (0.002-0.004) | 0.005 (0.003-0.006) | 1.33 (-0.19-2.87) |
| Mauritius | 0.05617 (0.04239-0.07272) | 0.19407 (0.14869-0.25028) | 2.46 (2.51-2.44) | 0.142 (0.107-0.183) | 0.195 (0.149-0.251) | 1.1 (1-1.21) |
| Mexico | 0.00014 (0.0001-0.00019) | 0.00047 (0.00034-0.00064) | 2.36 (2.4-2.37) | 0.012 (0.009-0.016) | 0.026 (0.019-0.035) | 2.42 (2.22-2.62) |
| Moldova | 168.34505 (126.3314-219.97132) | 450.78432 (325.81946-616.95974) | 1.68 (1.58-1.8) | 0.22 (0.165-0.287) | 0.224 (0.162-0.307) | 0.05 (-0.04-0.14) |
| Monaco | 0.56778 (0.38581-0.78643) | 0.71862 (0.44423-1.02591) | 0.27 (0.15-0.3) | 0.084 (0.057-0.117) | 0.079 (0.049-0.113) | -0.65 (-0.81--0.49) |
| Mongolia | 0.21324 (0.14962-0.29547) | 0.40584 (0.28791-0.55701) | 0.9 (0.92-0.89) | 0.294 (0.207-0.408) | 0.257 (0.182-0.353) | 0.08 (-0.23-0.39) |
| Montenegro | 259.49495 (143.96444-385.23514) | 192.14221 (130.6489-269.8292) | -0.26 (-0.09--0.3) | 1.705 (0.946-2.531) | 0.846 (0.575-1.188) | -2.75 (-3.31--2.2) |
| Morocco | 20.77617 (15.35558-27.42822) | 181.42878 (140.03188-233.60575) | 7.73 (8.12-7.52) | 0.633 (0.468-0.836) | 2.449 (1.89-3.153) | 4.78 (4.01-5.56) |
| Mozambique | 4.1983 (3.11459-5.54224) | 9.53089 (7.0166-12.62267) | 1.27 (1.25-1.28) | 0.273 (0.202-0.36) | 0.241 (0.177-0.319) | -0.41 (-0.44--0.37) |
| Myanmar | 0.78013 (0.53729-1.04172) | 1.00159 (0.70884-1.34455) | 0.28 (0.32-0.29) | 1.728 (1.19-2.307) | 1.106 (0.783-1.485) | -1.56 (-1.68--1.43) |
| Namibia | 19.26719 (14.15819-26.39745) | 28.22866 (19.69958-39.91256) | 0.47 (0.39-0.51) | 0.211 (0.155-0.289) | 0.142 (0.099-0.201) | -1.54 (-1.64--1.45) |
| Nauru | 3.90808 (2.3279-6.01381) | 7.69075 (4.564-11.73266) | 0.97 (0.96-0.95) | 0.627 (0.374-0.965) | 0.398 (0.236-0.607) | -1.58 (-1.65--1.52) |
| Nepal | 0.10765 (0.07273-0.15228) | 0.08287 (0.0558-0.11618) | -0.23 (-0.23--0.24) | 0.033 (0.022-0.047) | 0.012 (0.008-0.016) | -3.82 (-3.99--3.65) |
| Netherlands | 180.23221 (121.71726-246.96632) | 278.64724 (195.06564-381.29015) | 0.55 (0.6-0.54) | 0.859 (0.58-1.177) | 0.884 (0.619-1.21) | 0.82 (0.44-1.21) |
| New Zealand | 24.17272 (17.11215-32.37837) | 33.94201 (24.09806-47.22611) | 0.4 (0.41-0.46) | 0.73 (0.517-0.978) | 0.58 (0.412-0.807) | -0.75 (-1.07--0.44) |
| Nicaragua | 1.19168 (0.6844-1.83724) | 1.7992 (1.04946-2.81872) | 0.51 (0.53-0.53) | 0.631 (0.362-0.973) | 0.503 (0.293-0.788) | -0.73 (-0.76--0.71) |
| Niger | 48.29799 (35.14015-61.73588) | 86.06484 (61.54567-111.31979) | 0.78 (0.75-0.8) | 1.431 (1.041-1.829) | 1.091 (0.78-1.411) | -1.13 (-1.49--0.77) |
| Nigeria | 2.09506 (1.4816-2.94173) | 1.50411 (0.93143-2.16683) | -0.28 (-0.37--0.26) | 0.107 (0.076-0.15) | 0.032 (0.02-0.047) | -4.77 (-5.21--4.34) |
| Niue | 0.83209 (0.46522-1.3286) | 1.8125 (1.08452-2.82146) | 1.18 (1.33-1.12) | 0.467 (0.261-0.746) | 0.417 (0.25-0.649) | -0.27 (-0.3--0.24) |
| North Korea | 0.00184 (0.0014-0.00243) | 0.00563 (0.00419-0.00757) | 2.06 (1.99-2.12) | 0.013 (0.01-0.017) | 0.023 (0.017-0.031) | 1.71 (1.37-2.04) |
| North Macedonia | 2.32299 (0.56354-4.21516) | 5.92565 (3.35684-8.98782) | 1.55 (4.96-1.13) | 0.215 (0.052-0.389) | 0.365 (0.207-0.554) | 0.49 (-0.39-1.38) |
| Northern Mariana Islands | 0.36064 (0.19319-0.53087) | 0.70295 (0.49079-1.00574) | 0.95 (1.54-0.89) | 0.386 (0.207-0.568) | 0.396 (0.276-0.566) | -0.31 (-0.73-0.11) |
| Norway | 8.30834 (6.17944-10.19149) | 9.15667 (6.49551-11.83522) | 0.1 (0.05-0.16) | 0.427 (0.318-0.524) | 0.31 (0.22-0.4) | -1.49 (-1.63--1.35) |
| Oman | 0.00019 (0.00014-0.00025) | 0.00129 (0.00094-0.00175) | 5.79 (5.71-6) | 0.009 (0.007-0.012) | 0.027 (0.02-0.037) | 4.05 (3.64-4.46) |
| Pakistan | 5.31447 (3.83941-7.33436) | 1.30184 (0.84336-1.97596) | -0.76 (-0.78--0.73) | 0.23 (0.166-0.317) | 0.045 (0.029-0.069) | -7.34 (-7.96--6.71) |
| Palau | 0.45532 (0.27241-0.69406) | 0.69135 (0.4047-1.04965) | 0.52 (0.49-0.51) | 0.465 (0.278-0.709) | 0.387 (0.226-0.587) | -0.41 (-0.47--0.35) |
| Palestine | 55.2751 (40.13634-73.91201) | 69.12198 (50.99918-91.86078) | 0.25 (0.27-0.24) | 2.267 (1.646-3.032) | 1.777 (1.311-2.361) | -0.65 (-0.73--0.58) |
| Panama | 17.31843 (10.29435-25.95317) | 34.65554 (21.14473-51.1859) | 1 (1.05-0.97) | 0.656 (0.39-0.983) | 0.574 (0.35-0.847) | -0.42 (-0.63--0.21) |
| Papua New Guinea | 0.59662 (0.41432-0.80577) | 0.60609 (0.40852-0.8504) | 0.02 (-0.01-0.06) | 0.181 (0.126-0.245) | 0.102 (0.069-0.143) | -2.66 (-3.03--2.29) |
| Paraguay | 0.23851 (0.1474-0.34329) | 0.31642 (0.18954-0.47508) | 0.33 (0.29-0.38) | 0.747 (0.462-1.075) | 0.4 (0.24-0.601) | -2.05 (-2.4--1.69) |
| Peru | 3.19834 (1.95613-4.62004) | 5.29166 (3.22712-8.18313) | 0.65 (0.65-0.77) | 0.85 (0.52-1.228) | 0.661 (0.403-1.022) | -1.02 (-1.11--0.93) |
| Philippines | 2.03873 (1.4267-2.73828) | 2.08819 (1.37843-2.94172) | 0.02 (-0.03-0.07) | 1.458 (1.02-1.958) | 1.157 (0.763-1.629) | -0.82 (-0.98--0.66) |
| Poland | 0.25965 (0.15724-0.39159) | 0.48488 (0.31621-0.702) | 0.87 (1.01-0.79) | 0.8 (0.484-1.207) | 0.6 (0.391-0.868) | -1.01 (-1.07--0.95) |
| Portugal | 4.62241 (3.22033-6.30238) | 9.74775 (6.33762-13.35345) | 1.11 (0.97-1.12) | 0.083 (0.058-0.113) | 0.104 (0.068-0.143) | 0.39 (0.12-0.66) |
| Puerto Rico | 451.42341 (278.49213-665.19304) | 453.37699 (292.67378-636.35785) | 0 (0.05--0.04) | 0.86 (0.531-1.268) | 0.452 (0.292-0.635) | -2.4 (-2.6--2.2) |
| Qatar | 0.02569 (0.01894-0.03435) | 0.00369 (0.00244-0.0053) | -0.86 (-0.87--0.85) | 0.243 (0.179-0.325) | 0.016 (0.01-0.023) | -8.26 (-9.61--6.89) |
| Romania | 0.27041 (0.18394-0.38361) | 0.39471 (0.27687-0.54322) | 0.46 (0.51-0.42) | 0.424 (0.288-0.601) | 0.344 (0.241-0.473) | -0.59 (-0.72--0.47) |
| Russia | 2.43225 (1.63988-3.41327) | 3.62506 (2.4851-5.00507) | 0.49 (0.52-0.47) | 0.44 (0.296-0.617) | 0.397 (0.272-0.548) | -0.21 (-0.36--0.05) |
| Rwanda | 0.14268 (0.08752-0.21689) | 0.57656 (0.35941-0.88687) | 3.04 (3.11-3.09) | 0.665 (0.408-1.011) | 0.556 (0.347-0.855) | -0.67 (-0.86--0.48) |
| Saint Kitts and Nevis | 3.00277 (2.06898-4.25788) | 5.93812 (4.00233-8.20993) | 0.98 (0.93-0.93) | 0.449 (0.309-0.637) | 0.41 (0.277-0.567) | -0.21 (-0.34--0.08) |
| Saint Lucia | 0.70551 (0.48356-0.98295) | 1.38852 (0.93394-1.91272) | 0.97 (0.93-0.95) | 0.425 (0.291-0.592) | 0.387 (0.26-0.533) | -0.25 (-0.39--0.12) |
| Saint Vincent and the Grenadines | 0.84559 (0.57208-1.18579) | 1.26645 (0.84177-1.72449) | 0.5 (0.47-0.45) | 0.449 (0.304-0.629) | 0.384 (0.255-0.522) | -0.51 (-0.64--0.37) |
| Samoa | 0.16897 (0.1108-0.23965) | 0.27041 (0.16679-0.39282) | 0.6 (0.51-0.64) | 0.027 (0.018-0.038) | 0.041 (0.025-0.06) | 1.02 (-0.4-2.46) |
| San Marino | 10.46414 (7.0261-13.93764) | 19.87626 (12.99607-27.96137) | 0.9 (0.85-1.01) | 0.785 (0.527-1.046) | 0.531 (0.347-0.747) | -2.16 (-2.45--1.86) |
| São Tomé and Príncipe | 0.00149 (0.00109-0.00198) | 0.0054 (0.00379-0.00751) | 2.62 (2.48-2.79) | 0.025 (0.018-0.033) | 0.043 (0.03-0.06) | 1.68 (1.44-1.91) |
| Saudi Arabia | 132.26673 (87.83861-187.44805) | 54.144 (43.6785-69.22369) | -0.59 (-0.5--0.63) | 5.045 (3.35-7.149) | 1.432 (1.155-1.831) | -3.87 (-4.05--3.68) |
| Senegal | 0.87711 (0.49954-1.3919) | 1.69582 (1.0567-2.55401) | 0.93 (1.12-0.83) | 0.496 (0.283-0.788) | 0.385 (0.24-0.58) | -0.88 (-0.96--0.81) |
| Serbia | 24.50716 (15.01819-35.99427) | 38.48323 (25.35986-54.55776) | 0.57 (0.69-0.52) | 0.763 (0.467-1.12) | 0.562 (0.37-0.797) | -1.27 (-1.45--1.08) |
| Seychelles | 6.72222 (4.13982-10.03445) | 9.77171 (5.82337-15.33558) | 0.45 (0.41-0.53) | 0.796 (0.49-1.188) | 0.529 (0.315-0.83) | -1.67 (-1.8--1.54) |
| Sierra Leone | 5.08727 (3.12023-7.35813) | 7.3973 (4.64099-11.145) | 0.45 (0.49-0.51) | 0.809 (0.496-1.17) | 0.633 (0.397-0.953) | -1.12 (-1.26--0.99) |
| Singapore | 0.00587 (0.00432-0.00805) | 0.05344 (0.03981-0.06994) | 8.1 (8.22-7.69) | 0.005 (0.004-0.007) | 0.016 (0.012-0.021) | 5.06 (3.52-6.63) |
| Slovakia | 1.9789 (1.30731-2.80396) | 5.40076 (3.61001-7.46687) | 1.73 (1.76-1.66) | 0.45 (0.297-0.638) | 0.388 (0.259-0.536) | -0.42 (-0.55--0.29) |
| Slovenia | 8.0098 (4.88282-11.41368) | 9.75064 (5.85656-14.83849) | 0.22 (0.2-0.3) | 0.82 (0.5-1.168) | 0.545 (0.327-0.829) | -1.59 (-1.73--1.46) |
| Solomon Islands | 2.37382 (1.60838-3.27927) | 4.7612 (3.28611-6.49908) | 1.01 (1.04-0.98) | 0.449 (0.304-0.621) | 0.369 (0.255-0.503) | -0.56 (-0.69--0.44) |
| Somalia | 10.66459 (7.44505-14.9009) | 11.77056 (7.39727-17.18874) | 0.1 (-0.01-0.15) | 0.51 (0.356-0.712) | 0.371 (0.233-0.542) | -0.53 (-0.91--0.16) |
| South Africa | 3.46984 (2.15613-5.10572) | 8.40004 (4.95535-12.54425) | 1.42 (1.3-1.46) | 0.964 (0.599-1.418) | 0.891 (0.525-1.33) | -0.4 (-0.54--0.25) |
| South Korea | 0.14683 (0.11303-0.18996) | 0.47634 (0.37005-0.60019) | 2.24 (2.27-2.16) | 0.101 (0.078-0.131) | 0.111 (0.086-0.139) | 0.79 (0.58-1) |
| South Sudan | 3.59057 (2.15485-5.36807) | 6.62155 (4.01145-9.64668) | 0.84 (0.86-0.8) | 0.767 (0.46-1.147) | 0.626 (0.379-0.912) | -1.04 (-1.2--0.88) |
| Spain | 0.38363 (0.23277-0.55824) | 0.92233 (0.63849-1.27927) | 1.4 (1.74-1.29) | 0.275 (0.167-0.4) | 0.264 (0.183-0.366) | -1.42 (-1.87--0.97) |
| Sri Lanka | 0.00038 (0.00028-0.00051) | 0.00141 (0.00104-0.00196) | 2.71 (2.71-2.84) | 0.004 (0.003-0.005) | 0.005 (0.004-0.007) | 0.69 (0.61-0.78) |
| Sudan | 15.62604 (9.85931-22.17544) | 23.73394 (15.22418-33.64751) | 0.52 (0.54-0.52) | 0.866 (0.546-1.229) | 0.576 (0.369-0.816) | -1.28 (-1.38--1.18) |
| Suriname | 0.00084 (0.00063-0.00108) | 0.00136 (0.00103-0.0018) | 0.62 (0.63-0.67) | 0.009 (0.007-0.011) | 0.007 (0.005-0.009) | -1 (-1.34--0.66) |
| Sweden | 5.21695 (3.3425-7.40977) | 9.55695 (6.51529-13.65713) | 0.83 (0.95-0.84) | 0.4 (0.256-0.568) | 0.496 (0.338-0.709) | 1 (0.67-1.33) |
| Switzerland | 0.00387 (0.00156-0.00649) | 0.00587 (0.00235-0.00928) | 0.52 (0.51-0.43) | 0.07 (0.028-0.118) | 0.043 (0.017-0.068) | -2.04 (-3.36--0.7) |
| Syria | 0.00057 (0.00042-0.00075) | 0.01157 (0.00787-0.01735) | 19.3 (17.74-22.13) | 0.004 (0.003-0.006) | 0.031 (0.021-0.046) | 6.63 (5.92-7.35) |
| Taiwan (province of China) | 0.05317 (0.03791-0.06981) | 0.08675 (0.06072-0.1189) | 0.63 (0.6-0.7) | 0.48 (0.342-0.63) | 0.554 (0.388-0.76) | 0.18 (0-0.36) |
| Tajikistan | 9.18948 (5.72934-13.75673) | 13.07031 (8.38009-19.00148) | 0.42 (0.46-0.38) | 0.879 (0.548-1.315) | 0.551 (0.353-0.801) | -1.65 (-1.72--1.58) |
| Tanzania | 0.00988 (0.00742-0.01341) | 0.01832 (0.01341-0.02432) | 0.85 (0.81-0.81) | 0.071 (0.053-0.096) | 0.057 (0.042-0.076) | -1.98 (-2.59--1.37) |
| Thailand | 628.39117 (494.73192-809.37009) | 699.82862 (536.75727-876.86089) | 0.11 (0.08-0.08) | 4.515 (3.555-5.816) | 3.165 (2.428-3.966) | -1.63 (-1.99--1.27) |
| The Bahamas | 1.59351 (1.23185-2.07783) | 4.04143 (3.0572-5.31628) | 1.54 (1.48-1.56) | 0.023 (0.018-0.03) | 0.023 (0.018-0.03) | -0.26 (-0.62-0.1) |
| The Gambia | 1.13214 (0.72184-1.57218) | 2.51211 (1.72296-3.40303) | 1.22 (1.39-1.16) | 1.218 (0.776-1.691) | 1.036 (0.711-1.404) | -0.4 (-0.56--0.24) |
| Timor-Leste | 0.00276 (0.00206-0.00361) | 0.00967 (0.00726-0.01289) | 2.5 (2.52-2.57) | 0.029 (0.022-0.038) | 0.072 (0.054-0.096) | 2.96 (2.63-3.29) |
| Togo | 0.00005 (0.00003-0.00007) | 0.00016 (0.00011-0.00022) | 2.2 (2.67-2.14) | 0.007 (0.005-0.009) | 0.017 (0.012-0.023) | 3.03 (2.72-3.33) |
| Tokelau | 0.00011 (0.00008-0.00015) | 0.00013 (0.0001-0.00018) | 0.18 (0.25-0.2) | 0.029 (0.021-0.041) | 0.034 (0.025-0.046) | 0.53 (0.33-0.74) |
| Tonga | 0.52947 (0.3814-0.71692) | 0.36691 (0.24613-0.49841) | -0.31 (-0.35--0.3) | 0.02 (0.014-0.027) | 0.01 (0.007-0.014) | -2.9 (-3.16--2.63) |
| Trinidad and Tobago | 4.27261 (2.60118-6.16259) | 4.27296 (2.50504-6.17053) | 0 (-0.04-0) | 0.645 (0.392-0.93) | 0.386 (0.226-0.557) | -1.55 (-1.64--1.46) |
| Tunisia | 1.43546 (0.98026-2.00792) | 3.18526 (2.21328-4.35136) | 1.22 (1.26-1.17) | 0.449 (0.306-0.628) | 0.385 (0.268-0.526) | -0.48 (-0.58--0.37) |
| Türkiye | 2.96211 (1.96899-4.25638) | 5.49808 (3.71619-7.58603) | 0.86 (0.89-0.78) | 0.41 (0.272-0.589) | 0.366 (0.247-0.505) | -0.37 (-0.49--0.25) |
| Turkmenistan | 0.00171 (0.00128-0.00226) | 0.00939 (0.00678-0.01259) | 4.49 (4.3-4.57) | 0.07 (0.052-0.092) | 0.097 (0.07-0.13) | -0.24 (-0.99-0.52) |
| Tuvalu | 0.00026 (0.00019-0.00034) | 0.00078 (0.00056-0.00109) | 2 (1.95-2.21) | 0.016 (0.012-0.021) | 0.019 (0.013-0.026) | 0.15 (-0.07-0.38) |
| Uganda | 3.14549 (2.13753-4.43073) | 7.32127 (4.86685-10.0474) | 1.33 (1.28-1.27) | 0.425 (0.289-0.598) | 0.36 (0.239-0.494) | -0.47 (-0.59--0.35) |
| UK | 0.00004 (0.00003-0.00005) | 0.0001 (0.00007-0.00013) | 1.5 (1.33-1.6) | 0.015 (0.011-0.02) | 0.039 (0.028-0.052) | 3.1 (2.77-3.44) |
| Ukraine | 0.00481 (0.00344-0.00655) | 0.00501 (0.00362-0.00676) | 0.04 (0.05-0.03) | 0.075 (0.054-0.102) | 0.039 (0.028-0.052) | -3.27 (-3.66--2.88) |
| United Arab Emirates | 0.47907 (0.35719-0.63639) | 0.7756 (0.57216-1.03553) | 0.62 (0.6-0.63) | 0.079 (0.059-0.105) | 0.066 (0.049-0.089) | -2.21 (-2.94--1.48) |
| Uruguay | 0.06196 (0.04079-0.08588) | 0.19488 (0.12923-0.27538) | 2.15 (2.17-2.21) | 0.013 (0.009-0.018) | 0.019 (0.013-0.027) | 2.04 (1.35-2.73) |
| United States | 3.76501 (2.27261-5.70045) | 3.8767 (2.38644-5.81731) | 0.03 (0.05-0.02) | 0.908 (0.548-1.375) | 0.623 (0.383-0.934) | -1.5 (-1.64--1.36) |
| Uzbekistan | 0.17174 (0.11792-0.23958) | 0.27972 (0.19346-0.38389) | 0.63 (0.64-0.6) | 0.435 (0.299-0.607) | 0.365 (0.252-0.501) | -0.5 (-0.67--0.32) |
| Vanuatu | 2.04549 (1.37166-2.90921) | 3.88211 (2.63487-5.27243) | 0.9 (0.92-0.81) | 0.441 (0.296-0.628) | 0.418 (0.284-0.568) | -0.1 (-0.22-0.03) |
| Venezuela | 45.84458 (32.21386-63.82972) | 190.34332 (139.34277-252.31368) | 3.15 (3.33-2.95) | 1.653 (1.161-2.301) | 2.528 (1.851-3.351) | 0.22 (-0.8-1.25) |
| Viet Nam | 0.05374 (0.03736-0.0738) | 0.08501 (0.05917-0.11673) | 0.58 (0.58-0.58) | 0.48 (0.334-0.659) | 0.465 (0.324-0.638) | -0.2 (-0.27--0.12) |
| Virgin Islands | 0.24301 (0.17949-0.31566) | 1.11983 (0.84653-1.44909) | 3.61 (3.72-3.59) | 0.016 (0.012-0.021) | 0.036 (0.027-0.047) | 3.41 (2.96-3.86) |
| Yemen | 2.75543 (1.81-3.89464) | 6.60955 (4.50023-8.8936) | 1.4 (1.49-1.28) | 0.43 (0.282-0.608) | 0.369 (0.251-0.496) | -0.46 (-0.58--0.34) |
| Zambia | 0.24398 (0.16201-0.34764) | 0.61988 (0.43647-0.8337) | 1.54 (1.69-1.4) | 0.441 (0.293-0.628) | 0.401 (0.282-0.539) | -0.23 (-0.35--0.12) |
| Zimbabwe | 4.16134 (2.72426-5.83619) | 9.5992 (6.41641-13.766) | 1.31 (1.36-1.36) | 0.407 (0.267-0.572) | 0.352 (0.235-0.505) | -0.43 (-0.57--0.3) |

**Table S15. YLDs, YLD rates, and EAPC trends of HF impairment under the PC cause category across 204 countries and territories from 1990 to 2021 among adults aged ≥55.**

| **Location** | **YLDs** | |  | **YLD rates** | |  |
| --- | --- | --- | --- | --- | --- | --- |
|  | **1990 (95% UI)** | **2021 (95% UI)** | **Relative change, 1990–2021(95% UI)** | **1990, per 100,000 (95% UI)** | **2021, per 100,000 (95% UI)** | **EAPC (95% CI)** |
| Afghanistan | 0.009168 (0.005067-0.013958) | 0.029433 (0.016143-0.045144) | 2.21 (2.19-2.23) | 0.0012 (0.0006-0.0018) | 0.003 (0.0017-0.0047) | 3.24 (1.63-4.88) |
| Albania | 3.307134 (1.936027-4.965615) | 11.285745 (6.498787-17.482562) | 2.41 (2.36-2.52) | 0.0606 (0.0355-0.091) | 0.092 (0.053-0.1425) | 1.59 (1.2-1.98) |
| Algeria | 11.333459 (6.446836-17.511089) | 14.595313 (9.643955-20.689861) | 0.29 (0.5-0.18) | 0.1185 (0.0674-0.1832) | 0.0943 (0.0623-0.1337) | -0.91 (-1.2--0.63) |
| American Samoa | 0.099593 (0.060187-0.159585) | 0.145242 (0.085718-0.232153) | 0.46 (0.42-0.45) | 0.0021 (0.0013-0.0033) | 0.001 (0.0006-0.0017) | -2.96 (-3.39--2.54) |
| Andorra | 2.529501 (1.38585-3.807211) | 6.599784 (3.778446-9.842461) | 1.61 (1.73-1.59) | 0.1504 (0.0824-0.2264) | 0.1474 (0.0844-0.2198) | -0.63 (-0.8--0.45) |
| Angola | 0.00002 (0.000012-0.000031) | 0.000004 (0.000002-0.000006) | -0.8 (-0.83--0.81) | 0.0002 (0.0001-0.0004) | 0 (0-0) | -7.87 (-8.57--7.15) |
| Antigua and Barbuda | 0.007068 (0.004208-0.010428) | 0.027987 (0.017022-0.041715) | 2.96 (3.05-3) | 0.001 (0.0006-0.0014) | 0.0013 (0.0008-0.0019) | 0.94 (0.72-1.15) |
| Argentina | 0.063907 (0.038281-0.094518) | 0.133911 (0.078555-0.199636) | 1.1 (1.05-1.11) | 0.0036 (0.0022-0.0053) | 0.0028 (0.0016-0.0041) | -1.6 (-2.08--1.12) |
| Armenia | 0.001472 (0.000882-0.002236) | 0.002306 (0.001382-0.003412) | 0.57 (0.57-0.53) | 0.006 (0.0036-0.0091) | 0.0032 (0.0019-0.0047) | -2.42 (-2.93--1.91) |
| Australia | 0.174228 (0.105263-0.277214) | 0.44774 (0.254563-0.733133) | 1.57 (1.42-1.64) | 0.0011 (0.0007-0.0017) | 0.0011 (0.0006-0.0018) | -0.33 (-0.56--0.1) |
| Austria | 0.079336 (0.046625-0.118408) | 0.013315 (0.008065-0.019536) | -0.83 (-0.83--0.84) | 0.0047 (0.0028-0.007) | 0.0004 (0.0002-0.0006) | -7.39 (-8.76--6) |
| Azerbaijan | 0.001208 (0.000721-0.001833) | 0.001766 (0.001059-0.00257) | 0.46 (0.47-0.4) | 0.0026 (0.0015-0.0039) | 0.0019 (0.0012-0.0028) | -2.02 (-2.58--1.45) |
| Bahrain | 0.000126 (0.000052-0.000211) | 0.000577 (0.000247-0.000959) | 3.58 (3.75-3.55) | 0.0008 (0.0003-0.0013) | 0.001 (0.0004-0.0016) | -0.2 (-0.54-0.13) |
| Bangladesh | 6.278948 (3.315767-9.959714) | 16.193109 (8.642576-25.622855) | 1.58 (1.61-1.57) | 0.0423 (0.0223-0.0671) | 0.077 (0.0411-0.1218) | 1.89 (1.73-2.05) |
| Barbados | 0.846226 (0.489385-1.264963) | 2.116992 (1.216085-3.063121) | 1.5 (1.48-1.42) | 0.0318 (0.0184-0.0475) | 0.0376 (0.0216-0.0544) | 0.79 (0.67-0.92) |
| Belarus | 0.00009 (0.000051-0.000136) | 0.000146 (0.000084-0.000221) | 0.62 (0.65-0.62) | 0.0009 (0.0005-0.0014) | 0.001 (0.0006-0.0015) | 0.02 (-0.23-0.26) |
| Belgium | 5.196398 (2.797847-8.362999) | 1.360115 (0.68942-2.407859) | -0.74 (-0.75--0.71) | 0.0417 (0.0224-0.0671) | 0.01 (0.0051-0.0177) | -6.35 (-7.2--5.5) |
| Belize | 0.002975 (0.001736-0.004419) | 0.008272 (0.00494-0.012215) | 1.78 (1.85-1.76) | 0.0009 (0.0005-0.0013) | 0.0011 (0.0006-0.0016) | 0.61 (0.4-0.82) |
| Benin | 0.00853 (0.005253-0.012605) | 0.010871 (0.006746-0.016326) | 0.27 (0.28-0.3) | 0.0554 (0.0341-0.0819) | 0.0217 (0.0135-0.0326) | -3.46 (-3.92--3.01) |
| Bermuda | 0.005445 (0.003221-0.008031) | 0.031415 (0.019037-0.045917) | 4.77 (4.91-4.72) | 0.0004 (0.0002-0.0005) | 0.0006 (0.0004-0.0009) | 2.57 (1.89-3.26) |
| Bhutan | 25.261207 (13.091458-40.642608) | 52.090434 (28.480246-79.9151) | 1.06 (1.18-0.97) | 0.0853 (0.0442-0.1372) | 0.0998 (0.0546-0.1531) | -0.36 (-0.68--0.03) |
| Bolivia | 0.695638 (0.315249-1.123416) | 0.973053 (0.54208-1.525539) | 0.4 (0.72-0.36) | 0.0412 (0.0187-0.0665) | 0.0332 (0.0185-0.0521) | -0.07 (-0.7-0.57) |
| Bosnia and Herzegovina | 0.002254 (0.001379-0.003364) | 0.008883 (0.005413-0.012984) | 2.94 (2.93-2.86) | 0.0147 (0.009-0.0219) | 0.0163 (0.01-0.0239) | 0.86 (0.45-1.27) |
| Botswana | 0.019786 (0.011875-0.029939) | 0.076293 (0.045708-0.117193) | 2.86 (2.85-2.91) | 0.0032 (0.0019-0.0049) | 0.0046 (0.0027-0.007) | -1.5 (-2.37--0.62) |
| Brazil | 0.007647 (0.004195-0.012549) | 0.062562 (0.022488-0.11483) | 7.18 (4.36-8.15) | 0.0007 (0.0004-0.0011) | 0.006 (0.0021-0.0109) | 9.8 (6.97-12.7) |
| Brunei | 0.066464 (0.040303-0.098492) | 0.187381 (0.112466-0.275927) | 1.82 (1.79-1.8) | 0.0017 (0.001-0.0025) | 0.0022 (0.0013-0.0033) | 0.83 (0.71-0.95) |
| Bulgaria | 0.004975 (0.003-0.007463) | 0.001569 (0.000891-0.002479) | -0.68 (-0.7--0.67) | 0.0413 (0.0249-0.0619) | 0.0078 (0.0044-0.0123) | -5.92 (-6.48--5.36) |
| Burkina Faso | 0.250438 (0.154299-0.372263) | 0.7608 (0.46003-1.119482) | 2.04 (1.98-2.01) | 0.0042 (0.0026-0.0063) | 0.0039 (0.0024-0.0058) | -0.27 (-0.37--0.18) |
| Burundi | 0.022257 (0.01283-0.032728) | 0.044581 (0.026586-0.064906) | 1 (1.07-0.98) | 0.0041 (0.0024-0.0061) | 0.0038 (0.0022-0.0055) | -0.48 (-0.72--0.23) |
| Cabo Verde | 0.000479 (0.000282-0.00072) | 0.001818 (0.001058-0.002825) | 2.8 (2.75-2.92) | 0.0008 (0.0005-0.0012) | 0.0016 (0.0009-0.0025) | -0.75 (-1.92-0.42) |
| Cambodia | 6.206405 (3.24489-9.979224) | 32.138972 (20.469618-47.367968) | 4.18 (5.31-3.75) | 0.1247 (0.0652-0.2005) | 0.1922 (0.1224-0.2832) | 1.61 (1.23-2) |
| Cameroon | 0.002025 (0.001169-0.003067) | 0.000825 (0.000487-0.00124) | -0.59 (-0.58--0.6) | 0.0138 (0.008-0.021) | 0.002 (0.0012-0.0029) | -8.25 (-9.15--7.35) |
| Canada | 0.000034 (0.00002-0.000051) | 0.000096 (0.000056-0.000145) | 1.82 (1.8-1.84) | 0.0013 (0.0008-0.002) | 0.0016 (0.0009-0.0025) | 0.24 (-0.04-0.51) |
| Central African Republic | 0.000449 (0.000272-0.000657) | 0.001925 (0.001142-0.002825) | 3.29 (3.2-3.3) | 0.001 (0.0006-0.0015) | 0.0014 (0.0008-0.002) | 0.83 (0.7-0.96) |
| Chad | 0.154459 (0.093167-0.233614) | 0.123337 (0.076668-0.180956) | -0.2 (-0.18--0.23) | 0.0524 (0.0316-0.0793) | 0.0233 (0.0145-0.0342) | -4.14 (-4.86--3.42) |
| Chile | 0.000136 (0.00008-0.000219) | 0.000108 (0.000064-0.000163) | -0.21 (-0.2--0.26) | 0.0011 (0.0007-0.0018) | 0.0004 (0.0003-0.0006) | -4.83 (-7.4--2.19) |
| China | 1.489849 (0.883113-2.276062) | 1.630551 (0.92006-2.63257) | 0.09 (0.04-0.16) | 0.0628 (0.0372-0.0959) | 0.0484 (0.0273-0.0782) | -0.8 (-1.07--0.52) |
| Colombia | 0.010698 (0.006416-0.016247) | 0.016085 (0.009806-0.023662) | 0.5 (0.53-0.46) | 0.0246 (0.0147-0.0373) | 0.0144 (0.0088-0.0212) | -2.72 (-3.08--2.35) |
| Comoros | 0.007383 (0.004371-0.01137) | 0.015612 (0.009424-0.023423) | 1.11 (1.16-1.06) | 0.1257 (0.0744-0.1936) | 0.132 (0.0797-0.198) | -0.12 (-0.36-0.12) |
| Congo (Brazzaville) | 0.368281 (0.195956-0.598712) | 0.577871 (0.321338-0.950568) | 0.57 (0.64-0.59) | 0.08 (0.0426-0.1301) | 0.0535 (0.0297-0.088) | -1.57 (-1.72--1.41) |
| Cook Islands | 0.008308 (0.004901-0.012307) | 0.048468 (0.029389-0.071082) | 4.83 (5-4.78) | 0.0028 (0.0017-0.0042) | 0.0059 (0.0036-0.0087) | 2.46 (2.19-2.73) |
| Costa Rica | 0.004105 (0.002428-0.006245) | 0.008044 (0.004837-0.012056) | 0.96 (0.99-0.93) | 0.003 (0.0018-0.0045) | 0.0023 (0.0014-0.0035) | -1.77 (-2.39--1.15) |
| Côte d'Ivoire | 1.492324 (0.887133-2.298034) | 5.056661 (3.007649-7.519924) | 2.39 (2.39-2.27) | 0.0518 (0.0308-0.0798) | 0.0529 (0.0314-0.0786) | -0.24 (-0.76-0.28) |
| Croatia | 0.175047 (0.104772-0.262231) | 0.564098 (0.329582-0.835399) | 2.22 (2.15-2.19) | 0.0334 (0.02-0.05) | 0.0365 (0.0213-0.0541) | 0.62 (0.48-0.76) |
| Cuba | 1.908474 (1.148002-2.763571) | 7.721562 (4.992967-11.024877) | 3.05 (3.35-2.99) | 0.0968 (0.0582-0.1402) | 0.138 (0.0893-0.1971) | 1.78 (1.32-2.25) |
| Cyprus | 0.134794 (0.079939-0.201925) | 0.422906 (0.250817-0.644838) | 2.14 (2.14-2.19) | 0.0157 (0.0093-0.0235) | 0.0153 (0.0091-0.0233) | 0.33 (-0.16-0.83) |
| Czechia | 0.00016 (0.000095-0.000239) | 0.000982 (0.000576-0.001504) | 5.14 (5.06-5.29) | 0.0007 (0.0004-0.0011) | 0.0018 (0.0011-0.0028) | 3.6 (3.33-3.86) |
| Denmark | 0.008383 (0.004053-0.013384) | 0.051586 (0.030734-0.076491) | 5.15 (6.58-4.72) | 0.0023 (0.0011-0.0037) | 0.0034 (0.002-0.005) | 0.69 (0.46-0.92) |
| Djibouti | 0.028219 (0.017046-0.041988) | 0.120406 (0.071053-0.179031) | 3.27 (3.17-3.26) | 0.0099 (0.006-0.0148) | 0.0126 (0.0074-0.0187) | 0.12 (-0.16-0.4) |
| Dominica | 0.00012 (0.00007-0.000178) | 0.000293 (0.00017-0.000444) | 1.44 (1.43-1.49) | 0.0015 (0.0009-0.0022) | 0.0022 (0.0013-0.0034) | 1.01 (0.75-1.28) |
| Dominican Republic | 0.018141 (0.007327-0.030767) | 0.049296 (0.019953-0.082256) | 1.72 (1.72-1.67) | 0.0105 (0.0042-0.0178) | 0.0125 (0.0051-0.0208) | 0.72 (0.54-0.91) |
| DR Congo | 0.000145 (0.000091-0.000213) | 0.00024 (0.000139-0.000371) | 0.66 (0.53-0.74) | 0.0003 (0.0002-0.0004) | 0.0002 (0.0001-0.0003) | -1.35 (-1.66--1.04) |
| Ecuador | 0.009994 (0.006125-0.0147) | 0.016326 (0.009902-0.024145) | 0.63 (0.62-0.64) | 0.002 (0.0013-0.003) | 0.0016 (0.001-0.0024) | -1.12 (-1.34--0.89) |
| Egypt | 144.71842 (84.807291-230.125057) | 461.973671 (269.676566-745.463342) | 2.19 (2.18-2.24) | 0.1008 (0.0591-0.1603) | 0.1219 (0.0712-0.1967) | 0.4 (0.23-0.57) |
| El Salvador | 0.16544 (0.09679-0.250032) | 0.362196 (0.21488-0.544264) | 1.19 (1.22-1.18) | 0.0288 (0.0168-0.0435) | 0.0198 (0.0117-0.0297) | -2.18 (-2.67--1.69) |
| Equatorial Guinea | 6.534123 (3.852051-10.114387) | 5.364657 (3.071893-8.652319) | -0.18 (-0.2--0.14) | 0.094 (0.0554-0.1455) | 0.0249 (0.0143-0.0401) | -4.93 (-5.15--4.7) |
| Eritrea | 2.755877 (1.607948-4.262694) | 4.606935 (2.762476-7.043464) | 0.67 (0.72-0.65) | 0.0378 (0.0221-0.0585) | 0.0307 (0.0184-0.047) | -0.7 (-0.89--0.51) |
| Estonia | 0.045714 (0.027187-0.066964) | 0.115217 (0.068221-0.171635) | 1.52 (1.51-1.56) | 0.0184 (0.0109-0.027) | 0.0142 (0.0084-0.0211) | -1.28 (-1.53--1.02) |
| Eswatini | 0.098202 (0.049462-0.162908) | 1.553016 (0.834724-2.521292) | 14.81 (15.88-14.48) | 0.0149 (0.0075-0.0248) | 0.1089 (0.0585-0.1768) | 7.56 (7.07-8.06) |
| Ethiopia | 0.044896 (0.025911-0.070663) | 0.043014 (0.024856-0.065098) | -0.04 (-0.04--0.08) | 0.0092 (0.0053-0.0145) | 0.0055 (0.0032-0.0083) | -1.57 (-2.03--1.11) |
| Federated States of Micronesia | 0.97574 (0.524208-1.515249) | 0.251466 (0.13831-0.385083) | -0.74 (-0.74--0.75) | 0.0431 (0.0232-0.0669) | 0.0106 (0.0058-0.0162) | -5.41 (-5.82--4.99) |
| Fiji | 0.0794 (0.046711-0.117457) | 0.157877 (0.090594-0.236089) | 0.99 (0.94-1.01) | 0.0238 (0.014-0.0352) | 0.015 (0.0086-0.0224) | -1.58 (-1.66--1.51) |
| Finland | 0.017032 (0.010269-0.025928) | 0.052175 (0.030925-0.076647) | 2.06 (2.01-1.96) | 0.007 (0.0042-0.0107) | 0.007 (0.0042-0.0103) | -0.61 (-0.94--0.27) |
| France | 0.157427 (0.083989-0.240221) | 0.139047 (0.067136-0.222894) | -0.12 (-0.2--0.07) | 0.0141 (0.0075-0.0216) | 0.0093 (0.0045-0.0149) | -3 (-3.66--2.33) |
| Gabon | 0.018628 (0.010405-0.028106) | 0.036396 (0.020995-0.054429) | 0.95 (1.02-0.94) | 0.0036 (0.002-0.0055) | 0.0043 (0.0025-0.0064) | 1.51 (1.19-1.84) |
| Georgia | 0.220699 (0.123196-0.33986) | 0.353893 (0.194223-0.545692) | 0.6 (0.58-0.61) | 0.0636 (0.0355-0.0979) | 0.045 (0.0247-0.0694) | -1.54 (-1.68--1.39) |
| Germany | 0.015813 (0.009046-0.024216) | 0.040732 (0.022664-0.063772) | 1.58 (1.51-1.63) | 0.0018 (0.001-0.0028) | 0.0021 (0.0012-0.0033) | 0.36 (-0.42-1.15) |
| Ghana | 0.113261 (0.068464-0.166707) | 0.293929 (0.167691-0.436775) | 1.6 (1.45-1.62) | 0.0072 (0.0044-0.0106) | 0.0056 (0.0032-0.0083) | -1.57 (-1.86--1.27) |
| Greece | 0.256603 (0.148146-0.381806) | 0.259074 (0.14771-0.408564) | 0.01 (0-0.07) | 0.0247 (0.0143-0.0368) | 0.0158 (0.009-0.0249) | -2.32 (-2.72--1.92) |
| Greenland | 1.22198 (0.602061-1.918662) | 0.621927 (0.31738-0.987576) | -0.49 (-0.47--0.49) | 0.0244 (0.012-0.0383) | 0.0104 (0.0053-0.0164) | -3.23 (-3.64--2.82) |
| Grenada | 0.103061 (0.057497-0.156869) | 0.137371 (0.0767-0.21297) | 0.33 (0.33-0.36) | 0.014 (0.0078-0.0214) | 0.0125 (0.007-0.0194) | -0.34 (-0.65--0.03) |
| Guam | 0.083285 (0.041033-0.134461) | 0.092793 (0.045688-0.14321) | 0.11 (0.11-0.07) | 0.004 (0.002-0.0065) | 0.0033 (0.0016-0.0051) | -2.39 (-3.03--1.75) |
| Guatemala | 2.649141 (1.597992-4.134533) | 17.467657 (10.315652-28.015591) | 5.59 (5.46-5.78) | 0.0179 (0.0108-0.028) | 0.0403 (0.0238-0.0647) | 2.65 (2.19-3.12) |
| Guinea | 0.025962 (0.015198-0.038767) | 0.246163 (0.147004-0.363962) | 8.48 (8.67-8.39) | 0.0012 (0.0007-0.0019) | 0.0041 (0.0024-0.006) | 4.83 (4.42-5.24) |
| Guinea-Bissau | 0.144733 (0.088857-0.216225) | 0.389118 (0.231182-0.581082) | 1.69 (1.6-1.69) | 0.0399 (0.0245-0.0596) | 0.0393 (0.0233-0.0587) | -0.1 (-0.42-0.23) |
| Guyana | 0.001469 (0.000902-0.002207) | 0.028738 (0.01767-0.042672) | 18.56 (18.59-18.33) | 0.0052 (0.0032-0.0079) | 0.0177 (0.0109-0.0263) | 5.32 (4.6-6.05) |
| Haiti | 1.426814 (0.890789-2.10183) | 2.359144 (1.437029-3.459426) | 0.65 (0.61-0.65) | 0.0319 (0.0199-0.047) | 0.0213 (0.013-0.0313) | -1.49 (-1.6--1.37) |
| Honduras | 0.003255 (0.002009-0.004832) | 0.057422 (0.035032-0.085694) | 16.64 (16.44-16.73) | 0.0015 (0.0009-0.0022) | 0.0046 (0.0028-0.0068) | 4.78 (4.31-5.25) |
| Hungary | 0.125368 (0.063309-0.200387) | 0.205752 (0.108929-0.317967) | 0.64 (0.72-0.59) | 0.0291 (0.0147-0.0465) | 0.0281 (0.0149-0.0434) | -0.54 (-0.74--0.34) |
| Iceland | 0.129735 (0.076771-0.207657) | 1.431771 (0.845483-2.317114) | 10.04 (10.01-10.16) | 0.0028 (0.0017-0.0045) | 0.011 (0.0065-0.0178) | 5.98 (5.41-6.56) |
| India | 0.001394 (0.000851-0.002117) | 0.007637 (0.004641-0.01139) | 4.48 (4.45-4.38) | 0.0015 (0.0009-0.0023) | 0.0016 (0.001-0.0024) | 1.93 (0.71-3.16) |
| Indonesia | 0.069711 (0.042548-0.101785) | 0.425287 (0.26441-0.626406) | 5.1 (5.21-5.15) | 0.0184 (0.0112-0.0268) | 0.0434 (0.027-0.0639) | 3.52 (3.13-3.91) |
| Iran | 3.780645 (2.212327-5.896349) | 2.420183 (1.403969-3.940697) | -0.36 (-0.37--0.33) | 0.0491 (0.0287-0.0765) | 0.02 (0.0116-0.0325) | -5.12 (-6.28--3.94) |
| Iraq | 0.151752 (0.092579-0.223042) | 0.398316 (0.240532-0.58635) | 1.62 (1.6-1.63) | 0.012 (0.0073-0.0177) | 0.0103 (0.0062-0.0151) | -0.51 (-0.58--0.45) |
| Ireland | 0.02317 (0.01344-0.034899) | 0.045156 (0.021597-0.075539) | 0.95 (0.61-1.16) | 0.0213 (0.0123-0.032) | 0.0259 (0.0124-0.0433) | -1.61 (-2.36--0.86) |
| Israel | 0.138852 (0.080806-0.211805) | 0.215649 (0.124683-0.33546) | 0.55 (0.54-0.58) | 0.0419 (0.0244-0.0639) | 0.0357 (0.0206-0.0555) | -0.5 (-0.63--0.38) |
| Italy | 0.006513 (0.004004-0.009444) | 0.039449 (0.024354-0.059336) | 5.06 (5.08-5.28) | 0.0021 (0.0013-0.003) | 0.0047 (0.0029-0.0071) | 3.58 (3.28-3.88) |
| Jamaica | 0.002918 (0.001601-0.004681) | 0.012664 (0.006525-0.022291) | 3.34 (3.08-3.76) | 0.0008 (0.0004-0.0013) | 0.0029 (0.0015-0.0051) | 2.95 (0.96-4.98) |
| Japan | 0.77169 (0.421087-1.213564) | 4.159321 (2.176157-6.928666) | 4.39 (4.17-4.71) | 0.0024 (0.0013-0.0038) | 0.0098 (0.0051-0.0163) | 4.6 (2.53-6.72) |
| Jordan | 0.748208 (0.386864-1.195484) | 0.446087 (0.227764-0.695029) | -0.4 (-0.41--0.42) | 0.029 (0.015-0.0464) | 0.014 (0.0072-0.0219) | -3.01 (-3.28--2.75) |
| Kazakhstan | 0.028146 (0.016898-0.041816) | 0.179754 (0.106766-0.265953) | 5.39 (5.32-5.36) | 0.0012 (0.0007-0.0018) | 0.003 (0.0018-0.0044) | 3.8 (3.43-4.17) |
| Kenya | 0.000295 (0.000178-0.000453) | 0.000912 (0.000545-0.001378) | 2.09 (2.06-2.04) | 0.0003 (0.0002-0.0005) | 0.0003 (0.0002-0.0004) | 0.61 (0.38-0.84) |
| Kiribati | 0.003414 (0.00203-0.005035) | 0.020791 (0.01267-0.030636) | 5.09 (5.24-5.08) | 0.0004 (0.0002-0.0006) | 0.0007 (0.0004-0.001) | 3.48 (2.81-4.16) |
| Kuwait | 0.077304 (0.043513-0.119974) | 0.207152 (0.122239-0.32318) | 1.68 (1.81-1.69) | 0.0396 (0.0223-0.0615) | 0.0332 (0.0196-0.0517) | -0.6 (-0.72--0.48) |
| Kyrgyzstan | 0.502507 (0.254108-0.83885) | 1.428556 (0.766292-2.285651) | 1.84 (2.02-1.72) | 0.0423 (0.0214-0.0706) | 0.071 (0.0381-0.1136) | 1.48 (0.94-2.03) |
| Laos | 0.002842 (0.001407-0.004466) | 0.005946 (0.003283-0.009341) | 1.09 (1.33-1.09) | 0.006 (0.0029-0.0094) | 0.0061 (0.0034-0.0096) | -1.15 (-1.62--0.68) |
| Latvia | 0.012307 (0.007376-0.017935) | 0.094528 (0.056549-0.140495) | 6.68 (6.67-6.83) | 0.0014 (0.0008-0.002) | 0.004 (0.0024-0.0059) | 4.42 (4-4.83) |
| Lebanon | 0.093045 (0.050175-0.145772) | 0.213291 (0.123591-0.325976) | 1.29 (1.46-1.24) | 0.0137 (0.0074-0.0214) | 0.0163 (0.0094-0.0248) | 0.62 (0.51-0.74) |
| Lesotho | 0.009264 (0.005404-0.014254) | 0.025832 (0.015298-0.038801) | 1.79 (1.83-1.72) | 0.0939 (0.0548-0.1445) | 0.0976 (0.0578-0.1466) | -0.24 (-0.52-0.04) |
| Liberia | 0.013637 (0.007999-0.020333) | 0.116119 (0.069583-0.172) | 7.51 (7.7-7.46) | 0.0016 (0.0009-0.0023) | 0.005 (0.003-0.0074) | 4.5 (4.07-4.93) |
| Libya | 0.000061 (0.000037-0.000091) | 0.000284 (0.000168-0.000417) | 3.66 (3.54-3.58) | 0.0017 (0.001-0.0025) | 0.0034 (0.002-0.0049) | 2.24 (2-2.48) |
| Lithuania | 0.000517 (0.000323-0.000749) | 0.010073 (0.006048-0.014823) | 18.48 (17.72-18.79) | 0.0032 (0.002-0.0046) | 0.0066 (0.0039-0.0097) | 2.83 (2.32-3.34) |
| Luxembourg | 2.751701 (1.447998-4.37381) | 1.940596 (1.011443-3.047844) | -0.29 (-0.3--0.3) | 0.1162 (0.0612-0.1847) | 0.0555 (0.029-0.0872) | -3.28 (-3.67--2.9) |
| Madagascar | 0.790957 (0.475487-1.188197) | 2.352455 (1.388964-3.449435) | 1.97 (1.92-1.9) | 0.0132 (0.008-0.0199) | 0.0142 (0.0084-0.0209) | 0.81 (0.48-1.14) |
| Malawi | 0.009159 (0.005444-0.013619) | 0.071802 (0.042665-0.104746) | 6.84 (6.84-6.69) | 0.0011 (0.0007-0.0017) | 0.0032 (0.0019-0.0047) | 4.27 (3.93-4.61) |
| Malaysia | 0.075265 (0.039897-0.118394) | 0.2148 (0.122175-0.331668) | 1.85 (2.06-1.8) | 0.0093 (0.0049-0.0146) | 0.0107 (0.0061-0.0166) | 0.13 (-0.08-0.34) |
| Maldives | 0.001202 (0.000731-0.00175) | 0.031484 (0.018525-0.046398) | 25.19 (24.34-25.51) | 0.0021 (0.0013-0.0031) | 0.0044 (0.0026-0.0065) | 2.55 (2.36-2.73) |
| Mali | 0.013285 (0.007881-0.020019) | 0.037642 (0.022283-0.056466) | 1.83 (1.83-1.82) | 0.0011 (0.0006-0.0016) | 0.0031 (0.0018-0.0046) | 4.47 (4.08-4.85) |
| Malta | 1.87915 (1.152551-2.776063) | 3.938005 (2.32477-5.738579) | 1.1 (1.02-1.07) | 0.0248 (0.0152-0.0366) | 0.0169 (0.0099-0.0246) | -1.17 (-1.27--1.07) |
| Marshall Islands | 0.11635 (0.063777-0.194532) | 0.18049 (0.098522-0.286808) | 0.55 (0.54-0.47) | 0.1072 (0.0588-0.1793) | 0.0799 (0.0436-0.1269) | -1.06 (-1.1--1.02) |
| Mauritania | 0.002264 (0.001274-0.003531) | 0.004457 (0.002507-0.006864) | 0.97 (0.97-0.94) | 0.0003 (0.0002-0.0005) | 0.0004 (0.0002-0.0007) | 1.33 (-0.19-2.87) |
| Mauritius | 0.005209 (0.003116-0.007755) | 0.017987 (0.011247-0.026399) | 2.45 (2.61-2.4) | 0.0131 (0.0079-0.0195) | 0.0181 (0.0113-0.0265) | 1.1 (1-1.21) |
| Mexico | 0.000013 (0.000007-0.000019) | 0.000043 (0.000026-0.000065) | 2.31 (2.71-2.42) | 0.0011 (0.0006-0.0016) | 0.0024 (0.0014-0.0036) | 2.42 (2.22-2.61) |
| Moldova | 15.616505 (9.368457-23.661988) | 41.833767 (24.339068-65.823601) | 1.68 (1.6-1.78) | 0.0204 (0.0122-0.0309) | 0.0208 (0.0121-0.0327) | 0.05 (-0.04-0.14) |
| Monaco | 0.052615 (0.029824-0.080307) | 0.066612 (0.034111-0.102614) | 0.27 (0.14-0.28) | 0.0078 (0.0044-0.0119) | 0.0074 (0.0038-0.0113) | -0.65 (-0.81--0.49) |
| Mongolia | 0.01978 (0.01107-0.030385) | 0.037618 (0.022659-0.05785) | 0.9 (1.05-0.9) | 0.0273 (0.0153-0.0419) | 0.0238 (0.0144-0.0367) | 0.08 (-0.23-0.39) |
| Montenegro | 23.933114 (11.297542-39.623221) | 17.793359 (10.179329-27.618569) | -0.26 (-0.1--0.3) | 0.1573 (0.0742-0.2604) | 0.0783 (0.0448-0.1216) | -2.74 (-3.29--2.19) |
| Morocco | 1.925836 (1.113921-2.92689) | 16.811562 (10.521534-24.788797) | 7.73 (8.45-7.47) | 0.0587 (0.0339-0.0892) | 0.2269 (0.142-0.3346) | 4.78 (4.01-5.56) |
| Mozambique | 0.38931 (0.230899-0.597481) | 0.883953 (0.532926-1.348822) | 1.27 (1.31-1.26) | 0.0253 (0.015-0.0388) | 0.0223 (0.0135-0.0341) | -0.41 (-0.44--0.37) |
| Myanmar | 0.072396 (0.043841-0.110719) | 0.092913 (0.055534-0.140039) | 0.28 (0.27-0.26) | 0.1603 (0.0971-0.2452) | 0.1026 (0.0613-0.1547) | -1.56 (-1.68--1.43) |
| Namibia | 1.787431 (1.077001-2.786985) | 2.621007 (1.522427-4.305314) | 0.47 (0.41-0.54) | 0.0196 (0.0118-0.0306) | 0.0132 (0.0077-0.0217) | -1.54 (-1.64--1.45) |
| Nauru | 0.362991 (0.189461-0.608665) | 0.714256 (0.371587-1.190877) | 0.97 (0.96-0.96) | 0.0583 (0.0304-0.0977) | 0.0369 (0.0192-0.0616) | -1.58 (-1.65--1.52) |
| Nepal | 0.009979 (0.005555-0.01501) | 0.007687 (0.004278-0.011726) | -0.23 (-0.23--0.22) | 0.0031 (0.0017-0.0046) | 0.0011 (0.0006-0.0017) | -3.82 (-3.99--3.65) |
| Netherlands | 16.709961 (9.418634-25.857426) | 25.816854 (14.571649-39.29016) | 0.54 (0.55-0.52) | 0.0796 (0.0449-0.1232) | 0.0819 (0.0462-0.1247) | 0.82 (0.44-1.21) |
| New Zealand | 2.242377 (1.332568-3.400331) | 3.147618 (1.817505-4.860319) | 0.4 (0.36-0.43) | 0.0677 (0.0402-0.1027) | 0.0538 (0.0311-0.0831) | -0.76 (-1.07--0.44) |
| Nicaragua | 0.110736 (0.058306-0.184841) | 0.167214 (0.085179-0.282008) | 0.51 (0.46-0.53) | 0.0586 (0.0309-0.0979) | 0.0467 (0.0238-0.0788) | -0.74 (-0.76--0.71) |
| Niger | 4.479596 (2.853707-6.676063) | 7.982757 (5.016946-12.185163) | 0.78 (0.76-0.83) | 0.1327 (0.0845-0.1978) | 0.1012 (0.0636-0.1545) | -1.13 (-1.49--0.77) |
| Nigeria | 0.194146 (0.111605-0.297181) | 0.139482 (0.072726-0.220703) | -0.28 (-0.35--0.26) | 0.0099 (0.0057-0.0152) | 0.003 (0.0016-0.0047) | -4.77 (-5.21--4.34) |
| Niue | 0.077224 (0.038558-0.130627) | 0.168308 (0.087715-0.285426) | 1.18 (1.27-1.19) | 0.0434 (0.0217-0.0734) | 0.0387 (0.0202-0.0657) | -0.27 (-0.3--0.24) |
| North Korea | 0.00017 (0.000104-0.00025) | 0.000521 (0.000316-0.000763) | 2.06 (2.04-2.05) | 0.0012 (0.0007-0.0017) | 0.0021 (0.0013-0.0031) | 1.7 (1.37-2.04) |
| North Macedonia | 0.21519 (0.054807-0.413644) | 0.549865 (0.265561-0.953619) | 1.56 (3.85-1.31) | 0.0199 (0.0051-0.0382) | 0.0339 (0.0164-0.0588) | 0.49 (-0.39-1.38) |
| Northern Mariana Islands | 0.033411 (0.014921-0.052884) | 0.065189 (0.036812-0.101753) | 0.95 (1.47-0.92) | 0.0358 (0.016-0.0566) | 0.0367 (0.0207-0.0573) | -0.31 (-0.73-0.11) |
| Norway | 0.769857 (0.479602-1.142624) | 0.84898 (0.492584-1.294367) | 0.1 (0.03-0.13) | 0.0396 (0.0246-0.0587) | 0.0287 (0.0167-0.0438) | -1.49 (-1.62--1.35) |
| Oman | 0.000017 (0.00001-0.000026) | 0.000119 (0.000073-0.000185) | 6 (6.3-6.12) | 0.0008 (0.0005-0.0012) | 0.0025 (0.0015-0.0039) | 4.05 (3.63-4.46) |
| Pakistan | 0.49252 (0.28115-0.73629) | 0.120685 (0.06481-0.199747) | -0.75 (-0.77--0.73) | 0.0213 (0.0121-0.0318) | 0.0042 (0.0023-0.0069) | -7.34 (-7.96--6.71) |
| Palau | 0.042257 (0.022169-0.068653) | 0.064126 (0.034014-0.104593) | 0.52 (0.53-0.52) | 0.0432 (0.0226-0.0701) | 0.0359 (0.019-0.0585) | -0.41 (-0.47--0.35) |
| Palestine | 5.124011 (3.023761-7.667761) | 6.405798 (3.906573-9.362237) | 0.25 (0.29-0.22) | 0.2102 (0.124-0.3145) | 0.1646 (0.1004-0.2406) | -0.65 (-0.73--0.58) |
| Panama | 1.608624 (0.843535-2.593268) | 3.217625 (1.741228-5.258524) | 1 (1.06-1.03) | 0.0609 (0.0319-0.0982) | 0.0533 (0.0288-0.0871) | -0.42 (-0.63--0.21) |
| Papua New Guinea | 0.055288 (0.03167-0.083614) | 0.056226 (0.031787-0.085842) | 0.02 (0-0.03) | 0.0168 (0.0096-0.0254) | 0.0095 (0.0054-0.0145) | -2.66 (-3.03--2.29) |
| Paraguay | 0.022138 (0.012004-0.036189) | 0.029365 (0.016066-0.047266) | 0.33 (0.34-0.31) | 0.0694 (0.0376-0.1134) | 0.0371 (0.0203-0.0598) | -2.05 (-2.41--1.69) |
| Peru | 0.297001 (0.159266-0.492377) | 0.49138 (0.26942-0.814163) | 0.65 (0.69-0.65) | 0.0789 (0.0423-0.1308) | 0.0614 (0.0337-0.1017) | -1.02 (-1.11--0.93) |
| Philippines | 0.189267 (0.113166-0.289116) | 0.193709 (0.112482-0.301599) | 0.02 (-0.01-0.04) | 0.1353 (0.0809-0.2067) | 0.1073 (0.0623-0.167) | -0.82 (-0.98--0.66) |
| Poland | 0.024135 (0.012444-0.040636) | 0.045022 (0.026023-0.071709) | 0.87 (1.09-0.76) | 0.0744 (0.0383-0.1252) | 0.0557 (0.0322-0.0887) | -1.01 (-1.08--0.95) |
| Portugal | 0.428282 (0.242722-0.639854) | 0.903238 (0.504142-1.350878) | 1.11 (1.08-1.11) | 0.0077 (0.0044-0.0115) | 0.0097 (0.0054-0.0145) | 0.38 (0.11-0.65) |
| Puerto Rico | 41.711821 (21.373195-67.17432) | 42.030202 (23.236655-70.16527) | 0.01 (0.09-0.04) | 0.0795 (0.0407-0.128) | 0.0419 (0.0232-0.07) | -2.4 (-2.6--2.2) |
| Qatar | 0.002382 (0.001413-0.00358) | 0.000342 (0.000189-0.000556) | -0.86 (-0.87--0.84) | 0.0225 (0.0134-0.0338) | 0.0015 (0.0008-0.0024) | -8.26 (-9.61--6.89) |
| Romania | 0.025077 (0.014478-0.038291) | 0.036626 (0.021122-0.056588) | 0.46 (0.46-0.48) | 0.0393 (0.0227-0.06) | 0.0319 (0.0184-0.0493) | -0.59 (-0.71--0.47) |
| Russia | 0.225346 (0.129272-0.344638) | 0.336256 (0.198172-0.5293) | 0.49 (0.53-0.54) | 0.0407 (0.0234-0.0623) | 0.0368 (0.0217-0.0579) | -0.2 (-0.36--0.05) |
| Rwanda | 0.013255 (0.006996-0.022083) | 0.053584 (0.029204-0.087624) | 3.04 (3.17-2.97) | 0.0618 (0.0326-0.1029) | 0.0517 (0.0282-0.0845) | -0.67 (-0.86--0.48) |
| Saint Kitts and Nevis | 0.278477 (0.165697-0.43521) | 0.551087 (0.314094-0.851655) | 0.98 (0.9-0.96) | 0.0416 (0.0248-0.0651) | 0.0381 (0.0217-0.0588) | -0.21 (-0.33--0.08) |
| Saint Lucia | 0.065413 (0.037094-0.102216) | 0.128769 (0.074635-0.200667) | 0.97 (1.01-0.96) | 0.0394 (0.0223-0.0616) | 0.0359 (0.0208-0.0559) | -0.25 (-0.39--0.12) |
| Saint Vincent and the Grenadines | 0.07829 (0.04612-0.122881) | 0.117409 (0.067988-0.180294) | 0.5 (0.47-0.47) | 0.0415 (0.0245-0.0652) | 0.0356 (0.0206-0.0546) | -0.5 (-0.64--0.37) |
| Samoa | 0.015658 (0.008713-0.024523) | 0.02509 (0.012687-0.040374) | 0.6 (0.46-0.65) | 0.0025 (0.0014-0.0039) | 0.0038 (0.0019-0.0061) | 1.02 (-0.4-2.46) |
| San Marino | 0.971303 (0.579008-1.501159) | 1.845052 (1.082031-2.919929) | 0.9 (0.87-0.95) | 0.0729 (0.0434-0.1126) | 0.0493 (0.0289-0.0781) | -2.16 (-2.46--1.87) |
| São Tomé and Príncipe | 0.000138 (0.00008-0.000205) | 0.000501 (0.000298-0.000746) | 2.63 (2.72-2.64) | 0.0023 (0.0013-0.0034) | 0.004 (0.0024-0.0059) | 1.68 (1.44-1.91) |
| Saudi Arabia | 12.257238 (6.668678-18.993896) | 5.014468 (3.241334-7.335464) | -0.59 (-0.51--0.61) | 0.4675 (0.2543-0.7244) | 0.1326 (0.0857-0.194) | -3.87 (-4.05--3.68) |
| Senegal | 0.081516 (0.041105-0.134878) | 0.157506 (0.086099-0.255358) | 0.93 (1.09-0.89) | 0.0461 (0.0233-0.0763) | 0.0357 (0.0195-0.058) | -0.89 (-0.96--0.81) |
| Serbia | 2.277042 (1.213979-3.697432) | 3.571334 (2.046971-5.742154) | 0.57 (0.69-0.55) | 0.0709 (0.0378-0.1151) | 0.0522 (0.0299-0.0839) | -1.27 (-1.45--1.09) |
| Seychelles | 0.623706 (0.34039-1.021811) | 0.907932 (0.474571-1.519278) | 0.46 (0.39-0.49) | 0.0738 (0.0403-0.1209) | 0.0492 (0.0257-0.0823) | -1.67 (-1.8--1.54) |
| Sierra Leone | 0.472742 (0.251945-0.770962) | 0.686879 (0.37853-1.130984) | 0.45 (0.5-0.47) | 0.0752 (0.0401-0.1226) | 0.0588 (0.0324-0.0967) | -1.13 (-1.26--0.99) |
| Singapore | 0.000544 (0.000328-0.000809) | 0.004954 (0.00307-0.007419) | 8.11 (8.36-8.17) | 0.0004 (0.0003-0.0007) | 0.0015 (0.0009-0.0022) | 5.06 (3.52-6.63) |
| Slovakia | 0.183468 (0.105241-0.286982) | 0.50097 (0.293099-0.794501) | 1.73 (1.79-1.77) | 0.0417 (0.0239-0.0653) | 0.036 (0.0211-0.0571) | -0.42 (-0.55--0.28) |
| Slovenia | 0.74368 (0.41035-1.2268) | 0.905639 (0.48382-1.530834) | 0.22 (0.18-0.25) | 0.0761 (0.042-0.1255) | 0.0506 (0.027-0.0856) | -1.59 (-1.73--1.46) |
| Solomon Islands | 0.219926 (0.130141-0.343222) | 0.441709 (0.259374-0.671953) | 1.01 (0.99-0.96) | 0.0416 (0.0246-0.065) | 0.0342 (0.0201-0.052) | -0.56 (-0.68--0.44) |
| Somalia | 0.988641 (0.556079-1.529302) | 1.090626 (0.592139-1.722669) | 0.1 (0.06-0.13) | 0.0473 (0.0266-0.0731) | 0.0344 (0.0187-0.0543) | -0.53 (-0.91--0.16) |
| South Africa | 0.32242 (0.176246-0.533921) | 0.780478 (0.416116-1.25761) | 1.42 (1.36-1.36) | 0.0895 (0.0489-0.1483) | 0.0828 (0.0441-0.1334) | -0.39 (-0.54--0.25) |
| South Korea | 0.013617 (0.008293-0.02026) | 0.04418 (0.027171-0.062957) | 2.24 (2.28-2.11) | 0.0094 (0.0057-0.014) | 0.0103 (0.0063-0.0146) | 0.79 (0.58-1) |
| South Sudan | 0.333575 (0.17466-0.571116) | 0.615363 (0.3355-1.023514) | 0.84 (0.92-0.79) | 0.0713 (0.0373-0.122) | 0.0582 (0.0317-0.0968) | -1.04 (-1.2--0.88) |
| Spain | 0.035603 (0.018584-0.058362) | 0.085509 (0.047814-0.127951) | 1.4 (1.57-1.19) | 0.0255 (0.0133-0.0418) | 0.0245 (0.0137-0.0366) | -1.43 (-1.88--0.98) |
| Sri Lanka | 0.000035 (0.000021-0.000053) | 0.000131 (0.000077-0.000201) | 2.74 (2.67-2.79) | 0.0004 (0.0002-0.0005) | 0.0005 (0.0003-0.0007) | 0.69 (0.61-0.78) |
| Sudan | 1.451246 (0.792849-2.287751) | 2.203414 (1.265739-3.555102) | 0.52 (0.6-0.55) | 0.0804 (0.0439-0.1268) | 0.0535 (0.0307-0.0862) | -1.28 (-1.38--1.18) |
| Suriname | 0.000078 (0.000047-0.000114) | 0.000127 (0.000076-0.000186) | 0.63 (0.62-0.63) | 0.0008 (0.0005-0.0012) | 0.0006 (0.0004-0.0009) | -1 (-1.34--0.66) |
| Sweden | 0.483756 (0.253692-0.739322) | 0.885749 (0.504914-1.358685) | 0.83 (0.99-0.84) | 0.0371 (0.0194-0.0567) | 0.046 (0.0262-0.0706) | 1 (0.67-1.33) |
| Switzerland | 0.000358 (0.000137-0.000626) | 0.000545 (0.00019-0.000926) | 0.52 (0.39-0.48) | 0.0065 (0.0025-0.0114) | 0.004 (0.0014-0.0068) | -2.03 (-3.35--0.7) |
| Syria | 0.000053 (0.000031-0.00008) | 0.00107 (0.000634-0.001744) | 19.19 (19.45-20.8) | 0.0004 (0.0002-0.0006) | 0.0028 (0.0017-0.0046) | 6.63 (5.91-7.34) |
| Taiwan (province of China) | 0.004928 (0.002866-0.007534) | 0.008039 (0.004545-0.012282) | 0.63 (0.59-0.63) | 0.0445 (0.0259-0.068) | 0.0514 (0.029-0.0785) | 0.18 (0-0.36) |
| Tajikistan | 0.85391 (0.476578-1.429202) | 1.213494 (0.669947-2.000417) | 0.42 (0.41-0.4) | 0.0816 (0.0456-0.1366) | 0.0512 (0.0283-0.0844) | -1.65 (-1.72--1.58) |
| Tanzania | 0.000916 (0.000536-0.001362) | 0.001698 (0.001024-0.002565) | 0.85 (0.91-0.88) | 0.0066 (0.0038-0.0098) | 0.0053 (0.0032-0.008) | -1.98 (-2.59--1.36) |
| Thailand | 58.22496 (36.193602-83.845611) | 64.838112 (41.334931-92.042667) | 0.11 (0.14-0.1) | 0.4184 (0.2601-0.6025) | 0.2933 (0.187-0.4163) | -1.64 (-1.99--1.28) |
| The Bahamas | 0.147652 (0.090397-0.216739) | 0.374397 (0.224275-0.551399) | 1.54 (1.48-1.54) | 0.0021 (0.0013-0.0031) | 0.0021 (0.0013-0.0032) | -0.26 (-0.62-0.1) |
| The Gambia | 0.105088 (0.059553-0.162901) | 0.233087 (0.137882-0.356201) | 1.22 (1.32-1.19) | 0.113 (0.0641-0.1752) | 0.0961 (0.0569-0.1469) | -0.4 (-0.56--0.24) |
| Timor-Leste | 0.000256 (0.000153-0.000377) | 0.000895 (0.000541-0.001294) | 2.5 (2.54-2.43) | 0.0027 (0.0016-0.004) | 0.0067 (0.004-0.0096) | 2.96 (2.63-3.29) |
| Togo | 0.000005 (0.000003-0.000007) | 0.000014 (0.000008-0.000022) | 1.8 (1.67-2.14) | 0.0006 (0.0004-0.001) | 0.0016 (0.0009-0.0024) | 3.02 (2.72-3.33) |
| Tokelau | 0.00001 (0.000006-0.000014) | 0.000012 (0.000007-0.000018) | 0.2 (0.17-0.29) | 0.0027 (0.0017-0.0039) | 0.0032 (0.0019-0.0047) | 0.53 (0.33-0.74) |
| Tonga | 0.049074 (0.028711-0.073147) | 0.033996 (0.019402-0.052048) | -0.31 (-0.32--0.29) | 0.0018 (0.0011-0.0027) | 0.0009 (0.0005-0.0014) | -2.9 (-3.16--2.63) |
| Trinidad and Tobago | 0.39658 (0.215474-0.626865) | 0.396739 (0.205751-0.6305) | 0 (-0.05-0.01) | 0.0598 (0.0325-0.0946) | 0.0358 (0.0186-0.0569) | -1.55 (-1.64--1.46) |
| Tunisia | 0.133045 (0.078463-0.206069) | 0.295411 (0.170917-0.460433) | 1.22 (1.18-1.23) | 0.0416 (0.0245-0.0644) | 0.0357 (0.0207-0.0557) | -0.47 (-0.58--0.37) |
| Türkiye | 0.27448 (0.15797-0.437278) | 0.510187 (0.295834-0.800596) | 0.86 (0.87-0.83) | 0.038 (0.0218-0.0605) | 0.0339 (0.0197-0.0533) | -0.36 (-0.49--0.24) |
| Turkmenistan | 0.000159 (0.000095-0.000237) | 0.00087 (0.000511-0.001354) | 4.47 (4.38-4.71) | 0.0065 (0.0039-0.0097) | 0.009 (0.0053-0.0139) | -0.24 (-1-0.51) |
| Tuvalu | 0.000024 (0.000014-0.000035) | 0.000072 (0.000042-0.00011) | 2 (2-2.14) | 0.0015 (0.0009-0.0022) | 0.0017 (0.001-0.0026) | 0.15 (-0.07-0.38) |
| Uganda | 0.291301 (0.176528-0.44468) | 0.678734 (0.39736-1.04199) | 1.33 (1.25-1.34) | 0.0393 (0.0238-0.0601) | 0.0333 (0.0195-0.0512) | -0.46 (-0.58--0.34) |
| UK | 0.000003 (0.000002-0.000005) | 0.000009 (0.000006-0.000013) | 2 (2-1.6) | 0.0014 (0.0008-0.002) | 0.0036 (0.0022-0.0053) | 3.1 (2.76-3.44) |
| Ukraine | 0.000446 (0.000253-0.000678) | 0.000465 (0.000272-0.000701) | 0.04 (0.08-0.03) | 0.007 (0.004-0.0106) | 0.0036 (0.0021-0.0054) | -3.27 (-3.66--2.88) |
| United Arab Emirates | 0.044442 (0.026517-0.06816) | 0.07184 (0.044656-0.112286) | 0.62 (0.68-0.65) | 0.0073 (0.0044-0.0112) | 0.0062 (0.0038-0.0096) | -2.22 (-2.94--1.49) |
| Uruguay | 0.005742 (0.003071-0.008835) | 0.018071 (0.009901-0.027835) | 2.15 (2.22-2.15) | 0.0012 (0.0007-0.0019) | 0.0018 (0.001-0.0027) | 2.04 (1.35-2.74) |
| United States | 0.349696 (0.182774-0.581131) | 0.359912 (0.199095-0.590673) | 0.03 (0.09-0.02) | 0.0843 (0.0441-0.1401) | 0.0578 (0.032-0.0949) | -1.5 (-1.64--1.36) |
| Uzbekistan | 0.015925 (0.009102-0.024756) | 0.025937 (0.015211-0.03987) | 0.63 (0.67-0.61) | 0.0403 (0.0231-0.0627) | 0.0338 (0.0198-0.052) | -0.5 (-0.67--0.32) |
| Vanuatu | 0.18973 (0.108347-0.299109) | 0.360055 (0.210178-0.555341) | 0.9 (0.94-0.86) | 0.0409 (0.0234-0.0645) | 0.0388 (0.0226-0.0598) | -0.09 (-0.22-0.03) |
| Venezuela | 4.252671 (2.468336-6.562175) | 17.664277 (10.619583-27.562108) | 3.15 (3.3-3.2) | 0.1533 (0.089-0.2366) | 0.2346 (0.141-0.366) | 0.22 (-0.8-1.25) |
| Viet Nam | 0.004981 (0.00293-0.007693) | 0.00789 (0.004708-0.012003) | 0.58 (0.61-0.56) | 0.0445 (0.0262-0.0687) | 0.0432 (0.0257-0.0656) | -0.19 (-0.27--0.12) |
| Virgin Islands | 0.022536 (0.013445-0.034227) | 0.103822 (0.062779-0.152098) | 3.61 (3.67-3.44) | 0.0015 (0.0009-0.0023) | 0.0033 (0.002-0.0049) | 3.41 (2.96-3.86) |
| Yemen | 0.25555 (0.143745-0.406042) | 0.61321 (0.351704-0.934843) | 1.4 (1.45-1.3) | 0.0399 (0.0224-0.0634) | 0.0342 (0.0196-0.0521) | -0.46 (-0.58--0.33) |
| Zambia | 0.02261 (0.013185-0.035762) | 0.057457 (0.034443-0.088833) | 1.54 (1.61-1.48) | 0.0408 (0.0238-0.0646) | 0.0372 (0.0223-0.0574) | -0.23 (-0.35--0.11) |
| Zimbabwe | 0.385869 (0.217799-0.61419) | 0.889522 (0.51917-1.423975) | 1.31 (1.38-1.32) | 0.0378 (0.0213-0.0601) | 0.0326 (0.019-0.0522) | -0.43 (-0.57--0.3) |

**Table S16. Joinpoint analysis of prevalence rates for HF impairment under CRD cause categories from 1990 to 2021 among adults aged ≥55 years.**

| **Sex** | **Start Obs** | **End Obs** | **Measure** | **val** | **lower** | **upper** | ***P*.Value** |
| --- | --- | --- | --- | --- | --- | --- | --- |
| Both | 1990 | 2021 | AAPC | 0.35 | 0.22 | 0.47 | <0.001 |
| Female | 1990 | 2021 | AAPC | 0.38 | 0.27 | 0.5 | <0.001 |
| Male | 1990 | 2021 | AAPC | 0.34 | 0.23 | 0.46 | <0.001 |
| Both | 1990 | 2001 | APC | 1.43 | 1.32 | 1.54 | <0.001 |
| Both | 2001 | 2005 | APC | -1.43 | -2.22 | -0.63 | 0.001 |
| Both | 2005 | 2016 | APC | -0.17 | -0.29 | -0.05 | 0.009 |
| Both | 2016 | 2021 | APC | 0.55 | 0.18 | 0.92 | 0.006 |
| Female | 1990 | 2001 | APC | 1.2 | 1.09 | 1.3 | <0.001 |
| Female | 2001 | 2005 | APC | -1.46 | -2.19 | -0.73 | <0.001 |
| Female | 2005 | 2014 | APC | -0.21 | -0.36 | -0.05 | 0.011 |
| Female | 2014 | 2021 | APC | 0.95 | 0.75 | 1.15 | <0.001 |
| Male | 1990 | 2001 | APC | 1.62 | 1.56 | 1.69 | <0.001 |
| Male | 2001 | 2004 | APC | -1.63 | -2.58 | -0.66 | 0.002 |
| Male | 2004 | 2019 | APC | -0.3 | -0.34 | -0.25 | <0.001 |
| Male | 2019 | 2021 | APC | 1.13 | 0.04 | 2.23 | 0.043 |

**Table S17. Joinpoint analysis of YLD rates for HF impairment under CRD cause categories from 1990 to 2021 among adults aged ≥55 years.**

| **Sex** | **Start Obs** | **End Obs** | **Measure** | **val** | **lower** | **upper** | ***P*.Value** |
| --- | --- | --- | --- | --- | --- | --- | --- |
| Both | 1990 | 2021 | AAPC | 0.38 | 0.28 | 0.49 | <0.001 |
| Female | 1990 | 2021 | AAPC | 0.39 | 0.27 | 0.5 | <0.001 |
| Male | 1990 | 2021 | AAPC | 0.35 | 0.24 | 0.46 | <0.001 |
| Both | 1990 | 2001 | APC | 1.43 | 1.35 | 1.51 | <0.001 |
| Both | 2001 | 2005 | APC | -1.48 | -2.03 | -0.93 | <0.001 |
| Both | 2005 | 2019 | APC | -0.09 | -0.15 | -0.03 | 0.006 |
| Both | 2019 | 2021 | APC | 1.76 | 0.56 | 2.98 | 0.006 |
| Female | 1990 | 2001 | APC | 1.19 | 1.08 | 1.29 | <0.001 |
| Female | 2001 | 2005 | APC | -1.45 | -2.19 | -0.71 | 0.001 |
| Female | 2005 | 2014 | APC | -0.21 | -0.37 | -0.05 | 0.012 |
| Female | 2014 | 2021 | APC | 0.97 | 0.76 | 1.17 | <0.001 |
| Male | 1990 | 2001 | APC | 1.62 | 1.56 | 1.68 | <0.001 |
| Male | 2001 | 2004 | APC | -1.59 | -2.5 | -0.66 | 0.002 |
| Male | 2004 | 2019 | APC | -0.29 | -0.34 | -0.24 | <0.001 |
| Male | 2019 | 2021 | APC | 1.17 | 0.13 | 2.21 | 0.029 |

**Table S18. Joinpoint analysis of prevalence rates for HF impairment under the COPD cause category from 1990 to 2021 among adults aged ≥55 years.**

| **Sex** | **Start Obs** | **End Obs** | **Measure** | **val** | **lower** | **upper** | ***P*.Value** |
| --- | --- | --- | --- | --- | --- | --- | --- |
| Both | 1990 | 2021 | AAPC | 0.29 | 0.17 | 0.42 | <0.001 |
| Female | 1990 | 2021 | AAPC | 0.32 | 0.21 | 0.44 | <0.001 |
| Male | 1990 | 2021 | AAPC | 0.29 | 0.18 | 0.4 | <0.001 |
| Both | 1990 | 2001 | APC | 1.38 | 1.27 | 1.5 | <0.001 |
| Both | 2001 | 2005 | APC | -1.55 | -2.34 | -0.75 | <0.001 |
| Both | 2005 | 2016 | APC | -0.23 | -0.35 | -0.11 | <0.001 |
| Both | 2016 | 2021 | APC | 0.55 | 0.18 | 0.93 | 0.006 |
| Female | 1990 | 2001 | APC | 1.14 | 1.03 | 1.25 | <0.001 |
| Female | 2001 | 2005 | APC | -1.61 | -2.34 | -0.87 | <0.001 |
| Female | 2005 | 2014 | APC | -0.28 | -0.44 | -0.13 | 0.001 |
| Female | 2014 | 2021 | APC | 0.95 | 0.75 | 1.15 | <0.001 |
| Male | 1990 | 2001 | APC | 1.59 | 1.52 | 1.65 | <0.001 |
| Male | 2001 | 2004 | APC | -1.74 | -2.64 | -0.83 | <0.001 |
| Male | 2004 | 2019 | APC | -0.36 | -0.4 | -0.31 | <0.001 |
| Male | 2019 | 2021 | APC | 1.2 | 0.17 | 2.24 | 0.025 |

**Table S19. Joinpoint analysis of YLD rates for HF impairment under the COPD cause category from 1990 to 2021 among adults aged ≥55 years.**

| **Sex** | **Start Obs** | **End Obs** | **Measure** | **val** | **lower** | **upper** | ***P*.Value** |
| --- | --- | --- | --- | --- | --- | --- | --- |
| Both | 1990 | 2021 | AAPC | 0.33 | 0.23 | 0.44 | <0.001 |
| Female | 1990 | 2021 | AAPC | 0.33 | 0.21 | 0.44 | <0.001 |
| Male | 1990 | 2021 | AAPC | 0.3 | 0.2 | 0.41 | <0.001 |
| Both | 1990 | 2001 | APC | 1.38 | 1.3 | 1.46 | <0.001 |
| Both | 2001 | 2005 | APC | -1.61 | -2.16 | -1.06 | <0.001 |
| Both | 2005 | 2019 | APC | -0.14 | -0.2 | -0.09 | <0.001 |
| Both | 2019 | 2021 | APC | 1.87 | 0.68 | 3.09 | 0.004 |
| Female | 1990 | 2001 | APC | 1.13 | 1.02 | 1.24 | <0.001 |
| Female | 2001 | 2005 | APC | -1.6 | -2.34 | -0.85 | <0.001 |
| Female | 2005 | 2014 | APC | -0.28 | -0.44 | -0.12 | 0.001 |
| Female | 2014 | 2021 | APC | 0.97 | 0.77 | 1.17 | <0.001 |
| Male | 1990 | 2001 | APC | 1.58 | 1.52 | 1.64 | <0.001 |
| Male | 2001 | 2004 | APC | -1.7 | -2.56 | -0.83 | <0.001 |
| Male | 2004 | 2019 | APC | -0.35 | -0.39 | -0.3 | <0.001 |
| Male | 2019 | 2021 | APC | 1.24 | 0.26 | 2.23 | 0.015 |

**Table S20. Joinpoint analysis of prevalence rates for HF impairment under the ILD&PS cause category from 1990 to 2021 among adults aged ≥55 years.**

| **Sex** | **Start Obs** | **End Obs** | **Measure** | **val** | **lower** | **upper** | ***P*.Value** |
| --- | --- | --- | --- | --- | --- | --- | --- |
| Both | 1990 | 2021 | AAPC | 3.54 | 3.38 | 3.7 | <0.001 |
| Female | 1990 | 2021 | AAPC | 3.45 | 3.32 | 3.57 | <0.001 |
| Male | 1990 | 2021 | AAPC | 3.64 | 3.48 | 3.81 | <0.001 |
| Both | 1990 | 1995 | APC | 3.76 | 3.39 | 4.13 | <0.001 |
| Both | 1995 | 1999 | APC | 6.9 | 6.09 | 7.72 | <0.001 |
| Both | 1999 | 2004 | APC | 5.65 | 5.17 | 6.13 | <0.001 |
| Both | 2004 | 2009 | APC | 3.59 | 3.17 | 4 | <0.001 |
| Both | 2009 | 2018 | APC | 2.25 | 2.12 | 2.39 | <0.001 |
| Both | 2018 | 2021 | APC | -0.81 | -1.49 | -0.14 | 0.022 |
| Female | 1990 | 1995 | APC | 3.35 | 3.06 | 3.64 | <0.001 |
| Female | 1995 | 1999 | APC | 7.15 | 6.52 | 7.79 | <0.001 |
| Female | 1999 | 2004 | APC | 5.98 | 5.6 | 6.36 | <0.001 |
| Female | 2004 | 2009 | APC | 3.5 | 3.18 | 3.83 | <0.001 |
| Female | 2009 | 2018 | APC | 1.98 | 1.87 | 2.09 | <0.001 |
| Female | 2018 | 2021 | APC | -0.99 | -1.54 | -0.45 | 0.002 |
| Male | 1990 | 1995 | APC | 4.22 | 3.81 | 4.62 | <0.001 |
| Male | 1995 | 2000 | APC | 6.47 | 5.91 | 7.04 | <0.001 |
| Male | 2000 | 2005 | APC | 5.05 | 4.54 | 5.57 | <0.001 |
| Male | 2005 | 2014 | APC | 3.08 | 2.93 | 3.23 | <0.001 |
| Male | 2014 | 2018 | APC | 1.93 | 1.28 | 2.59 | <0.001 |
| Male | 2018 | 2021 | APC | -0.21 | -0.95 | 0.52 | 0.545 |

**Table S21. Joinpoint analysis of YLD rates for HF impairment under the ILD&PS cause category from 1990 to 2021 among adults aged ≥55 years.**

| **Sex** | **Start Obs** | **End Obs** | **Measure** | **val** | **lower** | **upper** | ***P*.Value** |
| --- | --- | --- | --- | --- | --- | --- | --- |
| Both | 1990 | 2021 | AAPC | 3.49 | 3.35 | 3.64 | <0.001 |
| Female | 1990 | 2021 | AAPC | 3.4 | 3.28 | 3.52 | <0.001 |
| Male | 1990 | 2021 | AAPC | 3.59 | 3.42 | 3.76 | <0.001 |
| Both | 1990 | 1995 | APC | 3.79 | 3.48 | 4.1 | <0.001 |
| Both | 1995 | 2000 | APC | 6.67 | 6.23 | 7.12 | <0.001 |
| Both | 2000 | 2004 | APC | 5.41 | 4.71 | 6.1 | <0.001 |
| Both | 2004 | 2009 | APC | 3.53 | 3.11 | 3.95 | <0.001 |
| Both | 2009 | 2018 | APC | 2.2 | 2.06 | 2.34 | <0.001 |
| Both | 2018 | 2021 | APC | -0.81 | -1.47 | -0.15 | 0.019 |
| Female | 1990 | 1995 | APC | 3.37 | 3.11 | 3.62 | <0.001 |
| Female | 1995 | 2000 | APC | 6.97 | 6.6 | 7.33 | <0.001 |
| Female | 2000 | 2004 | APC | 5.75 | 5.18 | 6.31 | <0.001 |
| Female | 2004 | 2009 | APC | 3.45 | 3.11 | 3.79 | <0.001 |
| Female | 2009 | 2018 | APC | 1.92 | 1.81 | 2.03 | <0.001 |
| Female | 2018 | 2021 | APC | -1 | -1.53 | -0.46 | 0.001 |
| Male | 1990 | 1995 | APC | 4.2 | 3.84 | 4.56 | <0.001 |
| Male | 1995 | 2000 | APC | 6.41 | 5.89 | 6.93 | <0.001 |
| Male | 2000 | 2005 | APC | 4.94 | 4.44 | 5.45 | <0.001 |
| Male | 2005 | 2014 | APC | 3.03 | 2.87 | 3.19 | <0.001 |
| Male | 2014 | 2018 | APC | 1.9 | 1.15 | 2.66 | <0.001 |
| Male | 2018 | 2021 | APC | -0.23 | -1 | 0.54 | 0.526 |

**Table S22. Joinpoint analysis of prevalence rates for HF impairment under the PC cause category from 1990 to 2021 among adults aged ≥55 years.**

| **Sex** | **Start Obs** | **End Obs** | **Measure** | **val** | **lower** | **upper** | ***P*.Value** |
| --- | --- | --- | --- | --- | --- | --- | --- |
| Both | 1990 | 2021 | AAPC | -0.17 | -0.29 | -0.04 | 0.008 |
| Female | 1990 | 2021 | AAPC | 0.95 | 0.79 | 1.11 | <0.001 |
| Male | 1990 | 2021 | AAPC | -0.3 | -0.38 | -0.22 | <0.001 |
| Both | 1990 | 1995 | APC | 0.5 | 0.16 | 0.85 | 0.006 |
| Both | 1995 | 2000 | APC | 2.07 | 1.61 | 2.53 | <0.001 |
| Both | 2000 | 2019 | APC | -1.07 | -1.11 | -1.03 | <0.001 |
| Both | 2019 | 2021 | APC | 1.2 | -0.23 | 2.66 | 0.096 |
| Female | 1990 | 1995 | APC | 1.19 | 0.79 | 1.6 | <0.001 |
| Female | 1995 | 2000 | APC | 4.28 | 3.71 | 4.84 | <0.001 |
| Female | 2000 | 2004 | APC | 0.79 | -0.06 | 1.65 | 0.066 |
| Female | 2004 | 2014 | APC | -0.72 | -0.87 | -0.58 | <0.001 |
| Female | 2014 | 2021 | APC | 0.93 | 0.7 | 1.16 | <0.001 |
| Male | 1990 | 1995 | APC | 0.36 | 0.2 | 0.52 | <0.001 |
| Male | 1995 | 2000 | APC | 1.9 | 1.68 | 2.12 | <0.001 |
| Male | 2000 | 2009 | APC | -1.28 | -1.35 | -1.22 | <0.001 |
| Male | 2009 | 2016 | APC | -0.95 | -1.05 | -0.86 | <0.001 |
| Male | 2016 | 2019 | APC | -1.92 | -2.49 | -1.35 | <0.001 |
| Male | 2019 | 2021 | APC | 1.89 | 1.21 | 2.58 | <0.001 |

**Table S23. Joinpoint analysis of YLD rates for HF impairment under the PC cause category from 1990 to 2021 among adults aged ≥55 years.**

| **Sex** | **Start Obs** | **End Obs** | **Measure** | **val** | **lower** | **upper** | ***P*.Value** |
| --- | --- | --- | --- | --- | --- | --- | --- |
| Both | 1990 | 2021 | AAPC | -0.16 | -0.27 | -0.05 | 0.006 |
| Female | 1990 | 2021 | AAPC | 0.95 | 0.79 | 1.11 | <0.001 |
| Male | 1990 | 2021 | AAPC | -0.29 | -0.37 | -0.21 | <0.001 |
| Both | 1990 | 1995 | APC | 0.5 | 0.21 | 0.8 | 0.002 |
| Both | 1995 | 2000 | APC | 2.07 | 1.66 | 2.48 | <0.001 |
| Both | 2000 | 2019 | APC | -1.07 | -1.11 | -1.03 | <0.001 |
| Both | 2019 | 2021 | APC | 1.34 | 0.01 | 2.69 | 0.048 |
| Female | 1990 | 1995 | APC | 1.19 | 0.8 | 1.57 | <0.001 |
| Female | 1995 | 2000 | APC | 4.28 | 3.73 | 4.83 | <0.001 |
| Female | 2000 | 2004 | APC | 0.8 | -0.04 | 1.65 | 0.062 |
| Female | 2004 | 2014 | APC | -0.73 | -0.88 | -0.58 | <0.001 |
| Female | 2014 | 2021 | APC | 0.95 | 0.72 | 1.18 | <0.001 |
| Male | 1990 | 1995 | APC | 0.36 | 0.22 | 0.5 | <0.001 |
| Male | 1995 | 2000 | APC | 1.89 | 1.69 | 2.09 | <0.001 |
| Male | 2000 | 2009 | APC | -1.28 | -1.35 | -1.22 | <0.001 |
| Male | 2009 | 2016 | APC | -0.95 | -1.05 | -0.85 | <0.001 |
| Male | 2016 | 2019 | APC | -1.94 | -2.53 | -1.34 | <0.001 |
| Male | 2019 | 2021 | APC | 2.03 | 1.38 | 2.68 | <0.001 |

**Table S24. Net drift in prevalence rates of HF impairment under CRD cause categories overall and for COPD, ILD&PS, and PC from 1990 to 2021 among adults aged ≥55 years.**

| **Region** | **Net Drift (%/year)** | | | |
| --- | --- | --- | --- | --- |
|  | **CRDs** | **COPD** | **ILD&PS** | **PC** |
| Global | -0.06 (-0.16-0.04) | -0.13 (-0.23--0.03) | 3.49 (3.42-3.57) | -0.35 (-0.52--0.17) |
| Low SDI | -0.38 (-0.44--0.31) | -0.39 (-0.45--0.32) | 0.58 (0.08-1.08) | -0.46 (-2.25-1.37) |
| Low-middle SDI | -0.1 (-0.14--0.06) | -0.11 (-0.16--0.07) | 1.15 (0.94-1.36) | -0.46 (-1.11-0.19) |
| Middle SDI | -1 (-1.16--0.83) | -1.03 (-1.2--0.87) | 2.85 (2.66-3.03) | -0.05 (-0.52-0.41) |
| High-middle SDI | -0.59 (-0.76--0.43) | -0.64 (-0.8--0.48) | 4.53 (4.3-4.75) | -0.24 (-0.63-0.15) |
| High SDI | 1.51 (1.44-1.59) | 1.38 (1.3-1.46) | 4.42 (4.31-4.53) | -0.59 (-0.84--0.34) |
| Andean Latin America | 1.14 (1.01-1.27) | 0.34 (0.18-0.49) | 3.72 (3.41-4.03) | 1.51 (-0.64-3.72) |
| Australasia | 1.57 (1.46-1.68) | 1.37 (1.25-1.48) | 6.02 (5.38-6.67) | 4.62 (2.37-6.92) |
| Caribbean | 1.12 (0.97-1.27) | 1.07 (0.92-1.23) | 3.77 (2.54-5.01) | -3.49 (-8.36-1.63) |
| Central Asia | -1.55 (-1.72--1.39) | -1.56 (-1.73--1.39) | -1.15 (-2.34-0.05) | -0.55 (-4.33-3.38) |
| Central Europe | 0.23 (0.13-0.33) | 0.2 (0.1-0.3) | 3.3 (2.44-4.17) | -4.2 (-5.75--2.62) |
| Central Latin America | 0.1 (0.04-0.16) | 0.01 (-0.05-0.07) | 3.17 (2.74-3.6) | -3.6 (-4.66--2.53) |
| Central Sub-Saharan Africa | -0.7 (-0.86--0.53) | -0.71 (-0.87--0.54) | -0.28 (-2.48-1.98) | -0.56 (-3.86-2.85) |
| East Asia | -1.91 (-2.12--1.7) | -1.93 (-2.14--1.72) | 3.74 (3.11-4.37) | 0.29 (-0.43-1.02) |
| Eastern Europe | -2.55 (-2.63--2.47) | -2.54 (-2.62--2.46) | -3.43 (-4.18--2.67) | -1.67 (-3.73-0.44) |
| Eastern Sub-Saharan Africa | -1.06 (-1.14--0.98) | -1.07 (-1.14--0.99) | 0.26 (-1.51-2.08) | -1.68 (-3.79-0.48) |
| High-income Asia Pacific | 0.9 (0.82-0.98) | 0.47 (0.4-0.54) | 3.35 (3.13-3.56) | -0.72 (-1.24--0.19) |
| High-income North America | 2.21 (2.03-2.39) | 2.13 (1.95-2.32) | 4.05 (3.84-4.27) | -1.59 (-2.16--1.01) |
| North Africa and Middle East | 0.72 (0.65-0.79) | 0.7 (0.63-0.77) | 2.75 (2.04-3.47) | 1.26 (-0.94-3.51) |
| Oceania | -0.3 (-0.66-0.07) | -0.29 (-0.66-0.07) | -0.33 (-4.98-4.54) | -3.7 (-12.89-6.46) |
| South Asia | -0.17 (-0.21--0.13) | -0.18 (-0.22--0.14) | 0.94 (0.75-1.14) | -0.73 (-1.49-0.03) |
| Southeast Asia | -0.44 (-0.48--0.4) | -0.45 (-0.48--0.41) | 2.06 (1.28-2.84) | -0.96 (-3.85-2.02) |
| Southern Latin America | 1.33 (1.21-1.44) | 1.14 (1.02-1.25) | 3.98 (3.48-4.48) | -0.32 (-1.77-1.15) |
| Southern Sub-Saharan Africa | -0.59 (-0.74--0.44) | -0.59 (-0.74--0.44) | -0.41 (-1.06-0.23) | -0.93 (-3.03-1.21) |
| Tropical Latin America | 0.29 (0.23-0.35) | 0.21 (0.15-0.27) | 4.68 (4.08-5.28) | 1.67 (0.44-2.9) |
| Western Europe | 1.89 (1.8-1.98) | 1.76 (1.67-1.85) | 5.88 (5.71-6.04) | -1.51 (-1.84--1.18) |
| Western Sub-Saharan Africa | -0.98 (-1.04--0.91) | -0.99 (-1.05--0.92) | -0.43 (-1.29-0.44) | -0.25 (-3.3-2.9) |
| **Five Countries** |  | | | |
| United States | 2.12 (1.92-2.32) | 2.06 (1.86-2.26) | 3.67 (3.43-3.9) | -2.11 (-2.72--1.49) |
| China | -1.95 (-2.17--1.73) | -1.97 (-2.18--1.75) | 3.48 (2.8-4.16) | 0.16 (-0.61-0.93) |
| Brazil | 0.27 (0.21-0.33) | 0.19 (0.13-0.25) | 4.71 (4.11-5.32) | 1.73 (0.49-2.99) |
| India | -0.18 (-0.21--0.15) | -0.19 (-0.23--0.16) | 0.91 (0.69-1.13) | -0.78 (-1.59-0.04) |
| Ethiopia | -1.38 (-1.58--1.18) | -1.39 (-1.59--1.19) | -0.1 (-3.22-3.12) | -2.17 (-5.46-1.24) |

**Table S25. Age effects on prevalence rates of HF impairment under CRD cause categories overall and for COPD, ILD&PS, and PC among adults aged ≥55 years.**

| **Region** | **Age** | **Prevalence rate (per 100,000 population)** | | | |
| --- | --- | --- | --- | --- | --- |
|  |  | **CRDs** | **COPD** | **ILD&PS** | **PC** |
| Global | 55-59 | 24.31 (23.24-25.44) | 23.5 (22.45-24.59) | 0.66 (0.64-0.68) | 0.13 (0.13-0.14) |
| Global | 60-64 | 61.41 (59.52-63.36) | 59.87 (58.03-61.77) | 1.27 (1.24-1.3) | 0.25 (0.24-0.26) |
| Global | 65-69 | 196.4 (191.89-201.02) | 191.57 (187.18-196.05) | 4.19 (4.13-4.26) | 0.65 (0.62-0.67) |
| Global | 70-74 | 350.9 (343.38-358.6) | 341.32 (334.02-348.79) | 9.01 (8.88-9.14) | 1 (0.96-1.03) |
| Global | 75-79 | 471.23 (459.06-483.72) | 456.97 (445.21-469.05) | 14.44 (14.18-14.71) | 1.31 (1.26-1.37) |
| Global | 80-84 | 620.54 (603.57-637.99) | 598.84 (582.51-615.62) | 25.06 (24.57-25.55) | 1.61 (1.54-1.68) |
| Global | 85-89 | 820.12 (795.36-845.64) | 785.18 (761.54-809.57) | 48.77 (47.75-49.81) | 1.91 (1.81-2) |
| Global | 90-94 | 917.79 (882.54-954.45) | 873.85 (840.33-908.71) | 71.16 (69.36-73.02) | 1.81 (1.68-1.93) |
| Global | 95+ | 890.84 (835.8-949.5) | 843.07 (790.94-898.64) | 90.17 (86.83-93.64) | 1.52 (1.34-1.72) |
| Low SDI | 55-59 | 42.47 (41.53-43.43) | 41.43 (40.52-42.36) | 0.86 (0.78-0.94) | 0.18 (0.15-0.23) |
| Low SDI | 60-64 | 84.44 (82.98-85.93) | 83 (81.57-84.46) | 1.19 (1.09-1.3) | 0.25 (0.2-0.31) |
| Low SDI | 65-69 | 221.14 (218.04-224.28) | 217.63 (214.6-220.7) | 3.13 (2.92-3.36) | 0.38 (0.31-0.46) |
| Low SDI | 70-74 | 372.16 (367.15-377.24) | 366.31 (361.4-371.29) | 5.3 (4.95-5.66) | 0.55 (0.45-0.66) |
| Low SDI | 75-79 | 508.38 (500.05-516.84) | 500.3 (492.15-508.59) | 7.37 (6.78-8.02) | 0.72 (0.58-0.91) |
| Low SDI | 80-84 | 761.13 (747.73-774.78) | 749.85 (736.71-763.23) | 10.36 (9.45-11.37) | 0.88 (0.69-1.14) |
| Low SDI | 85-89 | 1184.96 (1161.21-1209.2) | 1169.72 (1146.4-1193.5) | 13.6 (12.14-15.22) | 1.12 (0.81-1.53) |
| Low SDI | 90-94 | 1505.24 (1464.21-1547.42) | 1489.94 (1449.62-1531.38) | 12.27 (10.18-14.79) | 1.07 (0.64-1.78) |
| Low SDI | 95+ | 1695.69 (1615.72-1779.61) | 1680.75 (1602.18-1763.17) | 11.23 (7.61-16.57) | 1.02 (0.36-2.86) |
| Low-middle SDI | 55-59 | 26.84 (26.3-27.39) | 26.21 (25.68-26.75) | 0.56 (0.52-0.6) | 0.06 (0.05-0.08) |
| Low-middle SDI | 60-64 | 66.02 (65.07-66.98) | 64.94 (64.01-65.88) | 0.97 (0.92-1.03) | 0.11 (0.09-0.13) |
| Low-middle SDI | 65-69 | 205.44 (203.21-207.68) | 202.18 (200-204.4) | 3.02 (2.9-3.15) | 0.22 (0.19-0.26) |
| Low-middle SDI | 70-74 | 379.37 (375.52-383.27) | 373.16 (369.36-376.99) | 5.86 (5.63-6.09) | 0.36 (0.32-0.42) |
| Low-middle SDI | 75-79 | 545.52 (538.76-552.37) | 536.15 (529.5-542.89) | 8.97 (8.54-9.41) | 0.51 (0.43-0.61) |
| Low-middle SDI | 80-84 | 848.93 (837.77-860.24) | 834.59 (823.62-845.72) | 13.81 (13.1-14.55) | 0.7 (0.58-0.85) |
| Low-middle SDI | 85-89 | 1359.24 (1339.61-1379.17) | 1336.77 (1317.45-1356.37) | 21.66 (20.41-22.98) | 1.02 (0.82-1.26) |
| Low-middle SDI | 90-94 | 1723.67 (1692.38-1755.54) | 1697.93 (1667.12-1729.3) | 23.52 (21.7-25.51) | 1.27 (0.96-1.69) |
| Low-middle SDI | 95+ | 1773.06 (1723.35-1824.21) | 1746.45 (1697.55-1796.76) | 24.19 (21.16-27.64) | 1.41 (0.92-2.17) |
| Middle SDI | 55-59 | 24.74 (23-26.6) | 24.24 (22.54-26.07) | 0.34 (0.32-0.36) | 0.13 (0.12-0.15) |
| Middle SDI | 60-64 | 65.99 (62.85-69.28) | 65.09 (62-68.32) | 0.65 (0.62-0.68) | 0.26 (0.24-0.28) |
| Middle SDI | 65-69 | 219.38 (211.66-227.39) | 216.49 (208.89-224.37) | 2.22 (2.14-2.3) | 0.67 (0.63-0.72) |
| Middle SDI | 70-74 | 392.95 (380.03-406.32) | 387.49 (374.77-400.64) | 4.66 (4.5-4.82) | 0.98 (0.92-1.05) |
| Middle SDI | 75-79 | 535.66 (514.56-557.62) | 527.5 (506.78-549.06) | 7.72 (7.4-8.07) | 1.28 (1.18-1.38) |
| Middle SDI | 80-84 | 697.54 (668.29-728.08) | 685.7 (657.02-715.62) | 13.14 (12.53-13.77) | 1.41 (1.28-1.54) |
| Middle SDI | 85-89 | 894.39 (852.17-938.7) | 876.21 (834.96-919.49) | 25.26 (23.98-26.61) | 1.52 (1.35-1.7) |
| Middle SDI | 90-94 | 984.92 (923.3-1050.65) | 961.65 (901.61-1025.69) | 39.42 (36.97-42.04) | 1.34 (1.11-1.6) |
| Middle SDI | 95+ | 972.62 (871.81-1085.09) | 944.13 (846.26-1053.31) | 60.16 (54.89-65.94) | 1.42 (1.02-1.97) |
| High-middle SDI | 55-59 | 20.54 (19.06-22.14) | 20.07 (18.62-21.63) | 0.3 (0.28-0.32) | 0.14 (0.12-0.16) |
| High-middle SDI | 60-64 | 55.03 (52.35-57.85) | 54.02 (51.4-56.78) | 0.68 (0.65-0.72) | 0.3 (0.28-0.33) |
| High-middle SDI | 65-69 | 185.67 (179-192.59) | 182.28 (175.75-189.05) | 2.53 (2.43-2.63) | 0.86 (0.81-0.92) |
| High-middle SDI | 70-74 | 331.23 (320.18-342.65) | 324.67 (313.87-335.83) | 5.68 (5.47-5.89) | 1.33 (1.25-1.42) |
| High-middle SDI | 75-79 | 442.71 (425.28-460.86) | 433.17 (416.18-450.86) | 9.4 (8.97-9.85) | 1.73 (1.61-1.87) |
| High-middle SDI | 80-84 | 550.96 (528.08-574.84) | 538.27 (515.99-561.5) | 14.91 (14.17-15.7) | 1.85 (1.7-2.01) |
| High-middle SDI | 85-89 | 673.01 (641.92-705.6) | 655.05 (624.91-686.65) | 28.51 (26.93-30.18) | 1.85 (1.67-2.04) |
| High-middle SDI | 90-94 | 726.94 (683.21-773.48) | 705.36 (663.08-750.34) | 44.63 (41.57-47.92) | 1.47 (1.25-1.72) |
| High-middle SDI | 95+ | 663.09 (593.04-741.41) | 640.03 (572.57-715.43) | 63.93 (57.21-71.44) | 1.3 (0.95-1.77) |
| High SDI | 55-59 | 20.74 (19.97-21.54) | 19.11 (18.39-19.85) | 1.43 (1.38-1.5) | 0.17 (0.15-0.18) |
| High SDI | 60-64 | 52.66 (51.29-54.06) | 49.48 (48.2-50.81) | 2.82 (2.73-2.91) | 0.31 (0.28-0.33) |
| High SDI | 65-69 | 166.74 (163.58-169.96) | 156.84 (153.85-159.87) | 9.17 (8.96-9.38) | 0.77 (0.73-0.83) |
| High SDI | 70-74 | 303.35 (297.99-308.8) | 283.09 (278.06-288.2) | 19.91 (19.5-20.34) | 1.19 (1.11-1.26) |
| High SDI | 75-79 | 397.08 (388.48-405.88) | 367.59 (359.6-375.76) | 30.58 (29.77-31.41) | 1.48 (1.38-1.59) |
| High SDI | 80-84 | 539.7 (527.41-552.29) | 493.27 (481.99-504.81) | 52.64 (51.15-54.17) | 1.91 (1.77-2.05) |
| High SDI | 85-89 | 783.93 (764.8-803.54) | 706.37 (689.07-724.11) | 99.76 (96.72-102.9) | 2.28 (2.11-2.47) |
| High SDI | 90-94 | 943.55 (916.04-971.87) | 845.06 (820.32-870.54) | 139.07 (134.07-144.25) | 2.13 (1.93-2.35) |
| High SDI | 95+ | 963.95 (922.58-1007.17) | 858.15 (821.12-896.85) | 163.59 (155.32-172.3) | 1.6 (1.35-1.89) |
| Andean Latin America | 55-59 | 9.36 (8.63-10.16) | 6.01 (5.44-6.65) | 3.09 (2.68-3.56) | 0.17 (0.09-0.32) |
| Andean Latin America | 60-64 | 24.62 (23.27-26.04) | 17.43 (16.3-18.63) | 6.78 (6.1-7.54) | 0.34 (0.21-0.55) |
| Andean Latin America | 65-69 | 91.17 (87.7-94.78) | 61.06 (58.24-64.01) | 28.95 (27-31.05) | 1.01 (0.69-1.46) |
| Andean Latin America | 70-74 | 191.59 (184.97-198.45) | 125.27 (120.02-130.74) | 65.6 (61.57-69.89) | 1.57 (1.08-2.27) |
| Andean Latin America | 75-79 | 309.43 (296.36-323.08) | 198.27 (188.29-208.79) | 112.57 (103.78-122.1) | 2.44 (1.56-3.79) |
| Andean Latin America | 80-84 | 531.56 (508.16-556.03) | 338.29 (320.7-356.85) | 199.74 (183.03-217.97) | 3.2 (1.98-5.17) |
| Andean Latin America | 85-89 | 932.51 (889.13-978.01) | 588.99 (556.89-622.93) | 363.94 (330.89-400.3) | 3.66 (2.11-6.34) |
| Andean Latin America | 90-94 | 1318.89 (1249.75-1391.85) | 822.97 (773.01-876.15) | 542.41 (485.3-606.25) | 4.13 (2.03-8.41) |
| Andean Latin America | 95+ | 1709.44 (1598.15-1828.48) | 1032.41 (955.38-1115.65) | 799.96 (694.1-921.95) | 6.99 (2.76-17.7) |
| Australasia | 55-59 | 22.23 (21.03-23.5) | 21.17 (20-22.41) | 0.88 (0.68-1.15) | 0.08 (0.03-0.2) |
| Australasia | 60-64 | 70.71 (68.3-73.2) | 67.39 (65.04-69.83) | 2.86 (2.42-3.39) | 0.27 (0.16-0.47) |
| Australasia | 65-69 | 280.71 (274.38-287.19) | 264.82 (258.68-271.11) | 14.56 (13.13-16.13) | 1.46 (1.06-2.01) |
| Australasia | 70-74 | 545.8 (534.6-557.24) | 506.35 (495.63-517.3) | 40.89 (37.31-44.82) | 3.6 (2.7-4.8) |
| Australasia | 75-79 | 710.21 (691.89-729.02) | 644.68 (627.55-662.27) | 80.71 (71.39-91.26) | 6.39 (4.45-9.19) |
| Australasia | 80-84 | 867.23 (843.3-891.83) | 775.64 (753.62-798.3) | 131.38 (114.45-150.82) | 9.74 (6.59-14.4) |
| Australasia | 85-89 | 1049.44 (1017.43-1082.46) | 927.44 (898.31-957.5) | 205.98 (176.16-240.85) | 12.68 (8.17-19.68) |
| Australasia | 90-94 | 1128.57 (1085.23-1173.64) | 994.44 (955.13-1035.37) | 252.04 (207.91-305.53) | 13.77 (7.9-24.02) |
| Australasia | 95+ | 1117.08 (1046.65-1192.25) | 981.38 (917.73-1049.45) | 276.71 (205.27-373.03) | 10.28 (3.54-29.88) |
| Caribbean | 55-59 | 9.85 (9.1-10.65) | 9.6 (8.86-10.39) | 0.24 (0.14-0.39) | 0.01 (0-0.16) |
| Caribbean | 60-64 | 26.4 (25.01-27.87) | 25.89 (24.51-27.34) | 0.49 (0.34-0.73) | 0.01 (0-0.14) |
| Caribbean | 65-69 | 91.49 (88-95.12) | 89.54 (86.09-93.13) | 1.87 (1.43-2.45) | 0.06 (0.01-0.41) |
| Caribbean | 70-74 | 178.8 (172.41-185.42) | 174 (167.71-180.53) | 4.97 (3.91-6.32) | 0.05 (0-0.44) |
| Caribbean | 75-79 | 260.34 (249.01-272.19) | 252.65 (241.54-264.28) | 8.3 (6.1-11.29) | 0.09 (0.01-0.81) |
| Caribbean | 80-84 | 375.86 (358.58-393.97) | 364.44 (347.51-382.2) | 12.77 (9.13-17.86) | 0.12 (0.01-1.09) |
| Caribbean | 85-89 | 554.85 (527.45-583.68) | 537.1 (510.31-565.3) | 20.6 (14.25-29.78) | 0.17 (0.02-1.72) |
| Caribbean | 90-94 | 719.65 (678.63-763.15) | 696.02 (655.96-738.53) | 28.76 (18.65-44.35) | 0.16 (0.01-2.69) |
| Caribbean | 95+ | 886.53 (823.94-953.86) | 857.35 (796.27-923.12) | 37.21 (21.47-64.5) | 0.07 (0-2.78) |
| Central Asia | 55-59 | 6.94 (6.4-7.53) | 6.76 (6.23-7.35) | 0.16 (0.09-0.28) | 0.02 (0-0.11) |
| Central Asia | 60-64 | 16.5 (15.52-17.54) | 16.2 (15.23-17.22) | 0.24 (0.15-0.4) | 0.06 (0.02-0.18) |
| Central Asia | 65-69 | 50.12 (47.74-52.61) | 49.22 (46.86-51.7) | 0.75 (0.51-1.11) | 0.15 (0.06-0.34) |
| Central Asia | 70-74 | 77.41 (73.93-81.06) | 76.01 (72.56-79.62) | 1.16 (0.8-1.7) | 0.26 (0.11-0.58) |
| Central Asia | 75-79 | 85.23 (80.7-90.01) | 83.49 (79.01-88.22) | 1.49 (0.96-2.31) | 0.28 (0.1-0.76) |
| Central Asia | 80-84 | 95.54 (90.08-101.34) | 93.43 (88.04-99.15) | 1.88 (1.18-3) | 0.27 (0.09-0.83) |
| Central Asia | 85-89 | 111.25 (103.96-119.06) | 108.56 (101.37-116.25) | 2.63 (1.57-4.42) | 0.28 (0.07-1.15) |
| Central Asia | 90-94 | 111.81 (102.06-122.48) | 109 (99.4-119.52) | 2.73 (1.4-5.33) | 0.1 (0.01-1.78) |
| Central Asia | 95+ | 89.02 (75.82-104.52) | 86.72 (73.72-102.02) | 2.74 (1-7.53) | 0.35 (0.02-8.26) |
| Central Europe | 55-59 | 10.7 (10.32-11.09) | 10.28 (9.91-10.67) | 0.33 (0.27-0.41) | 0.08 (0.05-0.12) |
| Central Europe | 60-64 | 25.55 (24.89-26.22) | 24.77 (24.12-25.43) | 0.62 (0.52-0.74) | 0.13 (0.09-0.19) |
| Central Europe | 65-69 | 77.75 (76.23-79.29) | 75.5 (74-77.02) | 1.96 (1.72-2.22) | 0.25 (0.18-0.35) |
| Central Europe | 70-74 | 129.54 (127.13-131.99) | 125.74 (123.37-128.16) | 3.57 (3.17-4.01) | 0.31 (0.23-0.43) |
| Central Europe | 75-79 | 152.23 (148.79-155.75) | 147.76 (144.37-151.22) | 4.57 (3.91-5.33) | 0.27 (0.18-0.38) |
| Central Europe | 80-84 | 172.65 (168.39-177.03) | 167.75 (163.56-172.06) | 5.14 (4.3-6.14) | 0.25 (0.17-0.38) |
| Central Europe | 85-89 | 194.49 (188.83-200.31) | 189.05 (183.49-194.79) | 6.36 (5.1-7.92) | 0.19 (0.12-0.31) |
| Central Europe | 90-94 | 195.82 (187.58-204.41) | 190.29 (182.21-198.73) | 7.03 (5-9.87) | 0.15 (0.07-0.31) |
| Central Europe | 95+ | 173.68 (159.91-188.63) | 167.98 (154.52-182.6) | 7.85 (4.06-15.18) | 0.18 (0.05-0.62) |
| Central Latin America | 55-59 | 13.51 (13.07-13.97) | 12.61 (12.18-13.05) | 0.81 (0.71-0.93) | 0.06 (0.04-0.11) |
| Central Latin America | 60-64 | 32.85 (32.07-33.64) | 31.17 (30.42-31.94) | 1.56 (1.4-1.75) | 0.11 (0.07-0.17) |
| Central Latin America | 65-69 | 110.17 (108.29-112.08) | 104.7 (102.87-106.56) | 5.19 (4.78-5.63) | 0.27 (0.2-0.39) |
| Central Latin America | 70-74 | 213.37 (210.04-216.76) | 202.53 (199.29-205.82) | 10.76 (9.98-11.6) | 0.36 (0.26-0.5) |
| Central Latin America | 75-79 | 318.54 (312.48-324.72) | 302.05 (296.18-308.04) | 16.92 (15.33-18.67) | 0.46 (0.32-0.68) |
| Central Latin America | 80-84 | 498.73 (488.82-508.84) | 473.49 (463.89-483.29) | 26.31 (23.61-29.32) | 0.54 (0.36-0.8) |
| Central Latin America | 85-89 | 813.19 (796.1-830.65) | 772.19 (755.64-789.1) | 44.22 (39.23-49.85) | 0.63 (0.41-0.96) |
| Central Latin America | 90-94 | 1083.18 (1057.32-1109.68) | 1030.91 (1005.88-1056.57) | 55.96 (48.3-64.83) | 0.47 (0.27-0.82) |
| Central Latin America | 95+ | 1239.35 (1202.72-1277.09) | 1181.61 (1146.19-1218.12) | 59.69 (48.72-73.15) | 0.4 (0.19-0.87) |
| Central Sub-Saharan Africa | 55-59 | 39.61 (37.94-41.35) | 38.74 (37.09-40.46) | 0.62 (0.43-0.89) | 0.23 (0.13-0.42) |
| Central Sub-Saharan Africa | 60-64 | 72.82 (70.28-75.46) | 71.59 (69.07-74.2) | 0.86 (0.62-1.19) | 0.37 (0.22-0.63) |
| Central Sub-Saharan Africa | 65-69 | 169.21 (164.11-174.46) | 166.56 (161.51-171.77) | 2.01 (1.52-2.66) | 0.64 (0.39-1.04) |
| Central Sub-Saharan Africa | 70-74 | 262.59 (254.74-270.69) | 258.27 (250.48-266.3) | 3.4 (2.59-4.45) | 0.94 (0.58-1.53) |
| Central Sub-Saharan Africa | 75-79 | 344.41 (332.04-357.23) | 338.41 (326.16-351.12) | 4.82 (3.46-6.71) | 1.31 (0.73-2.36) |
| Central Sub-Saharan Africa | 80-84 | 489.42 (470.15-509.47) | 481.24 (462.15-501.11) | 6.63 (4.58-9.59) | 1.68 (0.86-3.28) |
| Central Sub-Saharan Africa | 85-89 | 732.14 (698.23-767.7) | 721.88 (688.22-757.19) | 7.85 (4.9-12.57) | 2.14 (0.91-5) |
| Central Sub-Saharan Africa | 90-94 | 910.41 (849.27-975.95) | 900.73 (839.96-965.91) | 7.58 (3.33-17.24) | 2.5 (0.63-9.93) |
| Central Sub-Saharan Africa | 95+ | 1019.71 (892.12-1165.56) | 1012.9 (885.88-1158.14) | 2.72 (0.16-46.38) | 2.49 (0.12-51.37) |
| East Asia | 55-59 | 32.51 (29.85-35.41) | 32.12 (29.5-34.97) | 0.13 (0.12-0.15) | 0.24 (0.22-0.26) |
| East Asia | 60-64 | 93.47 (88.49-98.73) | 92.64 (87.74-97.82) | 0.32 (0.29-0.34) | 0.51 (0.48-0.55) |
| East Asia | 65-69 | 321.63 (309.09-334.66) | 318.98 (306.63-331.83) | 1.18 (1.12-1.25) | 1.46 (1.38-1.55) |
| East Asia | 70-74 | 569.42 (548.78-590.85) | 564.94 (544.59-586.05) | 2.28 (2.16-2.41) | 2.21 (2.09-2.34) |
| East Asia | 75-79 | 763.36 (730.6-797.6) | 757.14 (724.86-790.85) | 3.68 (3.43-3.95) | 2.89 (2.7-3.1) |
| East Asia | 80-84 | 951.15 (907.68-996.7) | 944.05 (901.22-988.92) | 4.08 (3.75-4.43) | 2.99 (2.76-3.24) |
| East Asia | 85-89 | 1134.79 (1075.33-1197.54) | 1126.69 (1068.11-1188.49) | 5.1 (4.59-5.67) | 2.87 (2.58-3.18) |
| East Asia | 90-94 | 1185.62 (1098.52-1279.63) | 1177.44 (1091.66-1269.96) | 6.69 (5.69-7.86) | 1.93 (1.58-2.37) |
| East Asia | 95+ | 1108.08 (950.12-1292.31) | 1099.08 (943.66-1280.09) | 12.81 (9.74-16.83) | 1.97 (1.24-3.11) |
| Eastern Europe | 55-59 | 6.13 (5.91-6.36) | 6.01 (5.79-6.24) | 0.09 (0.07-0.13) | 0.03 (0.02-0.05) |
| Eastern Europe | 60-64 | 13.17 (12.8-13.54) | 12.99 (12.63-13.37) | 0.14 (0.1-0.19) | 0.03 (0.02-0.06) |
| Eastern Europe | 65-69 | 35.64 (34.82-36.47) | 35.19 (34.38-36.02) | 0.34 (0.27-0.43) | 0.1 (0.06-0.15) |
| Eastern Europe | 70-74 | 52.27 (51.14-53.43) | 51.65 (50.52-52.8) | 0.45 (0.36-0.56) | 0.15 (0.1-0.23) |
| Eastern Europe | 75-79 | 56.15 (54.76-57.59) | 55.53 (54.14-56.95) | 0.43 (0.33-0.56) | 0.16 (0.1-0.26) |
| Eastern Europe | 80-84 | 60.36 (58.78-61.99) | 59.73 (58.15-61.35) | 0.42 (0.32-0.56) | 0.17 (0.11-0.29) |
| Eastern Europe | 85-89 | 66.7 (64.71-68.74) | 66.05 (64.07-68.08) | 0.46 (0.33-0.64) | 0.16 (0.09-0.29) |
| Eastern Europe | 90-94 | 66.87 (64.18-69.66) | 66.23 (63.56-69.02) | 0.41 (0.26-0.65) | 0.21 (0.1-0.44) |
| Eastern Europe | 95+ | 56.99 (52.41-61.97) | 56.34 (51.78-61.29) | 0.43 (0.19-1) | 0.43 (0.14-1.3) |
| Eastern Sub-Saharan Africa | 55-59 | 56.31 (55.16-57.49) | 55.14 (54-56.3) | 0.84 (0.7-1) | 0.34 (0.25-0.45) |
| Eastern Sub-Saharan Africa | 60-64 | 96.12 (94.47-97.81) | 94.59 (92.95-96.26) | 1.08 (0.92-1.27) | 0.45 (0.34-0.59) |
| Eastern Sub-Saharan Africa | 65-69 | 210.55 (207.44-213.7) | 207.54 (204.45-210.67) | 2.37 (2.06-2.73) | 0.63 (0.49-0.82) |
| Eastern Sub-Saharan Africa | 70-74 | 316.15 (311.56-320.81) | 311.58 (307.02-316.21) | 3.68 (3.21-4.22) | 0.89 (0.69-1.15) |
| Eastern Sub-Saharan Africa | 75-79 | 400.4 (393.39-407.52) | 394.66 (387.7-401.73) | 4.62 (3.89-5.48) | 1.13 (0.83-1.54) |
| Eastern Sub-Saharan Africa | 80-84 | 531.29 (521.09-541.69) | 524.56 (514.43-534.9) | 5.32 (4.36-6.48) | 1.28 (0.9-1.83) |
| Eastern Sub-Saharan Africa | 85-89 | 714.88 (698.63-731.51) | 707.68 (691.5-724.23) | 5.2 (3.96-6.81) | 1.43 (0.91-2.24) |
| Eastern Sub-Saharan Africa | 90-94 | 833.25 (806.11-861.3) | 826.79 (799.76-854.73) | 4.2 (2.55-6.92) | 1.09 (0.49-2.45) |
| Eastern Sub-Saharan Africa | 95+ | 886.16 (834.38-941.15) | 881.25 (829.66-936.05) | 3.81 (1.27-11.44) | 0.32 (0.02-5.42) |
| High-income Asia Pacific | 55-59 | 8.9 (8.55-9.27) | 6.05 (5.8-6.3) | 2.52 (2.33-2.72) | 0.32 (0.27-0.38) |
| High-income Asia Pacific | 60-64 | 18.64 (18.08-19.21) | 13.85 (13.45-14.27) | 4.21 (3.96-4.48) | 0.48 (0.42-0.56) |
| High-income Asia Pacific | 65-69 | 45.84 (44.77-46.94) | 33.71 (32.93-34.51) | 11.17 (10.65-11.71) | 0.91 (0.8-1.04) |
| High-income Asia Pacific | 70-74 | 75.52 (73.86-77.21) | 55.18 (53.98-56.41) | 19.74 (18.88-20.63) | 1.03 (0.91-1.17) |
| High-income Asia Pacific | 75-79 | 96.22 (93.66-98.86) | 70.24 (68.39-72.13) | 26.07 (24.65-27.57) | 1.04 (0.9-1.21) |
| High-income Asia Pacific | 80-84 | 192.34 (187.17-197.65) | 139.96 (136.27-143.75) | 54.5 (51.44-57.74) | 1.6 (1.38-1.86) |
| High-income Asia Pacific | 85-89 | 448.94 (436.64-461.59) | 327.57 (318.81-336.56) | 130.6 (122.91-138.76) | 2.5 (2.13-2.93) |
| High-income Asia Pacific | 90-94 | 629.73 (610.89-649.15) | 462.29 (448.92-476.05) | 181.64 (169.45-194.71) | 2.57 (2.12-3.11) |
| High-income Asia Pacific | 95+ | 660.24 (636.36-685.01) | 485.81 (469.12-503.1) | 191.08 (174.5-209.23) | 1.94 (1.44-2.61) |
| High-income North America | 55-59 | 28.89 (26.71-31.23) | 27.28 (25.24-29.5) | 1.53 (1.41-1.67) | 0.05 (0.03-0.07) |
| High-income North America | 60-64 | 72.53 (68.69-76.58) | 69.37 (65.73-73.22) | 3 (2.81-3.2) | 0.11 (0.09-0.14) |
| High-income North America | 65-69 | 227.45 (218.64-236.62) | 217.12 (208.77-225.82) | 9.99 (9.55-10.46) | 0.3 (0.25-0.36) |
| High-income North America | 70-74 | 412.25 (397.33-427.72) | 389.98 (375.92-404.56) | 22.36 (21.47-23.29) | 0.55 (0.47-0.65) |
| High-income North America | 75-79 | 545.06 (520.82-570.42) | 511.26 (488.64-534.94) | 35.38 (33.6-37.27) | 0.74 (0.61-0.89) |
| High-income North America | 80-84 | 729.52 (695.33-765.39) | 676.7 (645.12-709.82) | 58.76 (55.63-62.07) | 1.06 (0.88-1.28) |
| High-income North America | 85-89 | 1004.66 (953.75-1058.27) | 923.68 (877.05-972.8) | 96.36 (90.82-102.23) | 1.34 (1.1-1.63) |
| High-income North America | 90-94 | 1182.61 (1109.61-1260.41) | 1082.53 (1015.84-1153.61) | 125.82 (117.25-135.02) | 1.29 (1.03-1.62) |
| High-income North America | 95+ | 1160.75 (1047.47-1286.28) | 1056.21 (952.97-1170.62) | 141.39 (127.27-157.08) | 0.75 (0.51-1.11) |
| North Africa and Middle East | 55-59 | 14.27 (13.92-14.63) | 14.01 (13.66-14.37) | 0.21 (0.17-0.26) | 0.04 (0.02-0.07) |
| North Africa and Middle East | 60-64 | 27.86 (27.31-28.42) | 27.48 (26.93-28.04) | 0.32 (0.27-0.39) | 0.05 (0.03-0.08) |
| North Africa and Middle East | 65-69 | 71.72 (70.57-72.89) | 70.68 (69.54-71.84) | 0.89 (0.77-1.03) | 0.14 (0.1-0.2) |
| North Africa and Middle East | 70-74 | 125.98 (124.06-127.93) | 124.05 (122.14-125.99) | 1.78 (1.55-2.05) | 0.18 (0.12-0.27) |
| North Africa and Middle East | 75-79 | 175.29 (171.99-178.66) | 172.73 (169.45-176.07) | 2.34 (1.94-2.81) | 0.25 (0.15-0.39) |
| North Africa and Middle East | 80-84 | 252.71 (247.57-257.95) | 249.04 (243.95-254.24) | 3.41 (2.77-4.19) | 0.3 (0.18-0.5) |
| North Africa and Middle East | 85-89 | 392.72 (383.76-401.89) | 386.69 (377.81-395.77) | 5.77 (4.55-7.32) | 0.52 (0.29-0.93) |
| North Africa and Middle East | 90-94 | 528.75 (513.1-544.89) | 518.86 (503.4-534.79) | 10.63 (7.92-14.28) | 0.91 (0.43-1.89) |
| North Africa and Middle East | 95+ | 623.06 (596.39-650.93) | 607.61 (581.39-635.02) | 19.9 (13.71-28.91) | 1.34 (0.49-3.67) |
| Oceania | 55-59 | 20.01 (17.3-23.15) | 19.8 (17.1-22.92) | 0.24 (0.04-1.43) | 0.04 (0-2.41) |
| Oceania | 60-64 | 65.63 (59.69-72.17) | 65.19 (59.27-71.71) | 0.6 (0.2-1.74) | 0.05 (0-2.33) |
| Oceania | 65-69 | 254.57 (237.89-272.42) | 252.71 (236.1-270.5) | 1.66 (0.72-3.85) | 0.06 (0-2.86) |
| Oceania | 70-74 | 488.39 (458.15-520.63) | 485.29 (455.15-517.42) | 3.12 (1.39-7.04) | 0.08 (0-4.06) |
| Oceania | 75-79 | 691.47 (638.97-748.28) | 686.88 (634.57-743.5) | 4.24 (1.52-11.86) | 0.11 (0-10.04) |
| Oceania | 80-84 | 1010.53 (928.41-1099.91) | 1004.29 (922.44-1093.39) | 6.82 (2.24-20.73) | 0.19 (0-20.27) |
| Oceania | 85-89 | 1487.79 (1350.85-1638.61) | 1479.7 (1343.15-1630.13) | 8.59 (2.22-33.22) | 0.41 (0-54.06) |
| Oceania | 90-94 | 1734.57 (1514.71-1986.34) | 1725.16 (1506.02-1976.2) | 3.89 (0.2-75.86) | 1.21 (0.01-203.4) |
| Oceania | 95+ | 1620.83 (1257.16-2089.72) | 1603.39 (1242.28-2069.48) | 14.82 (0.55-397.46) | 4.18 (0.02-945.82) |
| South Asia | 55-59 | 28.92 (28.41-29.45) | 28.26 (27.75-28.78) | 0.63 (0.59-0.67) | 0.03 (0.02-0.04) |
| South Asia | 60-64 | 73.5 (72.59-74.42) | 72.25 (71.35-73.16) | 1.17 (1.12-1.23) | 0.07 (0.06-0.09) |
| South Asia | 65-69 | 234.27 (232.11-236.44) | 230.3 (228.17-232.45) | 3.77 (3.64-3.92) | 0.18 (0.15-0.21) |
| South Asia | 70-74 | 439.96 (436.18-443.77) | 432.2 (428.46-435.97) | 7.42 (7.17-7.68) | 0.35 (0.3-0.4) |
| South Asia | 75-79 | 641.39 (634.61-648.25) | 629.56 (622.86-636.34) | 11.42 (10.94-11.93) | 0.52 (0.43-0.62) |
| South Asia | 80-84 | 1023.89 (1012.39-1035.51) | 1005.35 (993.99-1016.83) | 17.98 (17.16-18.84) | 0.73 (0.59-0.89) |
| South Asia | 85-89 | 1685.16 (1664.36-1706.21) | 1655.5 (1634.95-1676.31) | 28.68 (27.21-30.23) | 1.08 (0.86-1.35) |
| South Asia | 90-94 | 2163.57 (2129.79-2197.89) | 2130.55 (2097.13-2164.5) | 30.14 (28.02-32.43) | 1.18 (0.88-1.6) |
| South Asia | 95+ | 2426.63 (2365.37-2489.47) | 2392.41 (2331.82-2454.58) | 30.17 (26.42-34.46) | 0.95 (0.54-1.68) |
| Southeast Asia | 55-59 | 13 (12.75-13.27) | 12.93 (12.67-13.19) | 0.07 (0.05-0.09) | 0.01 (0-0.02) |
| Southeast Asia | 60-64 | 36.23 (35.74-36.73) | 36.11 (35.62-36.6) | 0.12 (0.09-0.15) | 0.01 (0-0.02) |
| Southeast Asia | 65-69 | 125.53 (124.27-126.8) | 125.11 (123.85-126.37) | 0.4 (0.33-0.47) | 0.02 (0.01-0.04) |
| Southeast Asia | 70-74 | 230.72 (228.56-232.9) | 229.83 (227.68-232.01) | 0.87 (0.74-1.03) | 0.03 (0.01-0.07) |
| Southeast Asia | 75-79 | 319.01 (315.42-322.65) | 317.77 (314.18-321.4) | 1.25 (1.02-1.55) | 0.03 (0.01-0.08) |
| Southeast Asia | 80-84 | 457.59 (452.13-463.11) | 455.87 (450.43-461.38) | 1.66 (1.32-2.1) | 0.05 (0.02-0.15) |
| Southeast Asia | 85-89 | 681.34 (672.42-690.38) | 678.3 (669.41-687.31) | 3.31 (2.57-4.27) | 0.13 (0.04-0.36) |
| Southeast Asia | 90-94 | 836.59 (822.79-850.63) | 832.08 (818.34-846.06) | 5.71 (4.2-7.76) | 0.2 (0.06-0.68) |
| Southeast Asia | 95+ | 885.33 (862.82-908.43) | 880.14 (857.72-903.14) | 6.99 (4.41-11.08) | 0.09 (0.01-1.04) |
| Southern Latin America | 55-59 | 11.53 (10.87-12.23) | 10.53 (9.9-11.19) | 0.91 (0.74-1.12) | 0.08 (0.04-0.16) |
| Southern Latin America | 60-64 | 27.08 (25.95-28.26) | 24.85 (23.77-25.98) | 2.06 (1.77-2.41) | 0.12 (0.06-0.22) |
| Southern Latin America | 65-69 | 84.93 (82.28-87.66) | 77.31 (74.79-79.92) | 7.19 (6.44-8.04) | 0.34 (0.21-0.55) |
| Southern Latin America | 70-74 | 153.54 (149.04-158.17) | 137.72 (133.49-142.09) | 15.48 (14.02-17.09) | 0.62 (0.41-0.96) |
| Southern Latin America | 75-79 | 205.36 (197.97-213.03) | 182.22 (175.35-189.35) | 23.77 (20.89-27.05) | 0.75 (0.44-1.26) |
| Southern Latin America | 80-84 | 320.36 (308.32-332.88) | 282.41 (271.32-293.95) | 40.5 (35.28-46.5) | 1.11 (0.65-1.92) |
| Southern Latin America | 85-89 | 578.43 (555.65-602.13) | 507.15 (486.31-528.88) | 79.36 (68.46-92) | 1.83 (1.03-3.23) |
| Southern Latin America | 90-94 | 844.03 (806.07-883.77) | 741.19 (706.51-777.57) | 116.55 (97.85-138.81) | 2.05 (1.03-4.07) |
| Southern Latin America | 95+ | 1101.54 (1037.55-1169.48) | 968.61 (910.35-1030.6) | 148.73 (116.84-189.33) | 2.42 (0.95-6.19) |
| Southern Sub-Saharan Africa | 55-59 | 81.91 (77.72-86.33) | 78.99 (74.92-83.29) | 2.34 (1.97-2.79) | 0.57 (0.39-0.83) |
| Southern Sub-Saharan Africa | 60-64 | 137.84 (131.99-143.95) | 134.02 (128.3-139.99) | 3.06 (2.62-3.58) | 0.74 (0.53-1.03) |
| Southern Sub-Saharan Africa | 65-69 | 304.26 (292.97-315.99) | 296.45 (285.4-307.91) | 6.66 (5.81-7.62) | 1.14 (0.83-1.58) |
| Southern Sub-Saharan Africa | 70-74 | 459.2 (442.26-476.79) | 446.22 (429.68-463.39) | 11.59 (10.18-13.2) | 1.41 (1.01-1.97) |
| Southern Sub-Saharan Africa | 75-79 | 591.62 (565.6-618.85) | 573.93 (548.56-600.47) | 15.67 (13.41-18.3) | 2.04 (1.39-3) |
| Southern Sub-Saharan Africa | 80-84 | 822.83 (784.34-863.21) | 800.11 (762.52-839.56) | 19.74 (16.69-23.35) | 3.03 (2.02-4.55) |
| Southern Sub-Saharan Africa | 85-89 | 1195.34 (1132.55-1261.62) | 1169.64 (1108.01-1234.7) | 22.65 (18.54-27.67) | 3.14 (1.91-5.14) |
| Southern Sub-Saharan Africa | 90-94 | 1472.48 (1367.83-1585.13) | 1449.47 (1346.42-1560.39) | 20.59 (15-28.26) | 2.95 (1.34-6.46) |
| Southern Sub-Saharan Africa | 95+ | 1635.7 (1422.23-1881.21) | 1615.41 (1405.14-1857.15) | 19.02 (9.44-38.32) | 0.9 (0.05-15.27) |
| Tropical Latin America | 55-59 | 13.31 (12.89-13.75) | 12.95 (12.53-13.38) | 0.28 (0.22-0.35) | 0.07 (0.04-0.11) |
| Tropical Latin America | 60-64 | 33.92 (33.16-34.71) | 33.13 (32.37-33.9) | 0.64 (0.54-0.75) | 0.13 (0.09-0.19) |
| Tropical Latin America | 65-69 | 115.05 (113.17-116.95) | 112.14 (110.29-114.03) | 2.53 (2.25-2.83) | 0.35 (0.26-0.48) |
| Tropical Latin America | 70-74 | 222.24 (218.91-225.63) | 215.68 (212.41-219.01) | 6.43 (5.82-7.12) | 0.6 (0.45-0.8) |
| Tropical Latin America | 75-79 | 326.42 (320.38-332.57) | 315.2 (309.3-321.21) | 12.5 (10.93-14.29) | 0.94 (0.65-1.35) |
| Tropical Latin America | 80-84 | 510.64 (500.76-520.72) | 491.12 (481.51-500.93) | 24.48 (21.16-28.32) | 1.61 (1.1-2.37) |
| Tropical Latin America | 85-89 | 821.8 (804.88-839.08) | 787.2 (770.81-803.94) | 49.67 (42.34-58.26) | 2.72 (1.78-4.15) |
| Tropical Latin America | 90-94 | 1089.82 (1064.03-1116.22) | 1041.8 (1016.89-1067.33) | 73 (60.55-87.99) | 4.95 (3.08-7.95) |
| Tropical Latin America | 95+ | 1315.28 (1276.05-1355.72) | 1256.96 (1219.09-1296) | 91.27 (71.69-116.2) | 6.8 (3.78-12.21) |
| Western Europe | 55-59 | 18.61 (17.72-19.55) | 17.43 (16.58-18.32) | 0.98 (0.92-1.04) | 0.15 (0.12-0.17) |
| Western Europe | 60-64 | 48.42 (46.81-50.08) | 45.78 (44.25-47.36) | 2.29 (2.19-2.39) | 0.27 (0.24-0.31) |
| Western Europe | 65-69 | 156.15 (152.37-160.03) | 146.7 (143.12-150.38) | 8.66 (8.39-8.93) | 0.75 (0.68-0.83) |
| Western Europe | 70-74 | 295.49 (288.81-302.31) | 274.07 (267.81-280.48) | 21.65 (21.05-22.26) | 1.22 (1.11-1.34) |
| Western Europe | 75-79 | 397.78 (386.83-409.04) | 365.43 (355.26-375.88) | 35.79 (34.53-37.1) | 1.55 (1.4-1.71) |
| Western Europe | 80-84 | 567.86 (551.46-584.75) | 520.74 (505.56-536.38) | 56.63 (54.43-58.92) | 1.87 (1.69-2.08) |
| Western Europe | 85-89 | 897.72 (870.22-926.1) | 824.29 (798.84-850.54) | 97.53 (93.33-101.93) | 1.98 (1.77-2.21) |
| Western Europe | 90-94 | 1152.41 (1111.33-1195.01) | 1059.15 (1021.21-1098.49) | 134.56 (127.53-141.98) | 1.64 (1.43-1.89) |
| Western Europe | 95+ | 1267.75 (1203.39-1335.55) | 1163.63 (1104.51-1225.91) | 163.2 (150.56-176.9) | 1.25 (0.98-1.6) |
| Western Sub-Saharan Africa | 55-59 | 42.29 (41.36-43.24) | 40.4 (39.49-41.32) | 1.6 (1.41-1.82) | 0.31 (0.22-0.42) |
| Western Sub-Saharan Africa | 60-64 | 71.75 (70.41-73.12) | 69.75 (68.42-71.1) | 1.66 (1.47-1.88) | 0.36 (0.27-0.48) |
| Western Sub-Saharan Africa | 65-69 | 153.01 (150.53-155.53) | 149.2 (146.75-151.68) | 3.4 (3.05-3.79) | 0.42 (0.31-0.56) |
| Western Sub-Saharan Africa | 70-74 | 223.15 (219.57-226.79) | 218.29 (214.75-221.9) | 4.4 (3.94-4.91) | 0.45 (0.33-0.62) |
| Western Sub-Saharan Africa | 75-79 | 280.84 (275.45-286.33) | 275.17 (269.83-280.62) | 5.13 (4.48-5.87) | 0.52 (0.35-0.76) |
| Western Sub-Saharan Africa | 80-84 | 384.75 (376.76-392.9) | 378.61 (370.67-386.72) | 5.43 (4.63-6.36) | 0.58 (0.36-0.92) |
| Western Sub-Saharan Africa | 85-89 | 558.96 (545.76-572.48) | 553.5 (540.32-567) | 4.27 (3.39-5.38) | 0.56 (0.29-1.07) |
| Western Sub-Saharan Africa | 90-94 | 686.73 (665.08-709.08) | 681.69 (660.08-704) | 3.27 (2.14-5) | 0.68 (0.25-1.85) |
| Western Sub-Saharan Africa | 95+ | 773.81 (734.33-815.42) | 768.69 (729.34-810.16) | 3.23 (1.37-7.57) | 0.32 (0.02-6.29) |
| **Five Countries** | | | | | |
| United States | 55-59 | 28.46 (26.12-31.01) | 27 (24.79-29.4) | 1.4 (1.27-1.54) | 0.04 (0.03-0.06) |
| United States | 60-64 | 70.42 (66.32-74.77) | 67.61 (63.72-71.75) | 2.67 (2.48-2.88) | 0.1 (0.08-0.13) |
| United States | 65-69 | 217.88 (208.56-227.62) | 208.82 (199.96-218.07) | 8.77 (8.31-9.25) | 0.27 (0.22-0.33) |
| United States | 70-74 | 389.41 (373.81-405.67) | 370.24 (355.5-385.59) | 19.15 (18.27-20.08) | 0.48 (0.4-0.58) |
| United States | 75-79 | 509.51 (484.45-535.87) | 480.76 (457.29-505.44) | 29.82 (28.08-31.66) | 0.62 (0.51-0.77) |
| United States | 80-84 | 682.51 (647.18-719.77) | 637.18 (604.41-671.73) | 49.6 (46.57-52.82) | 0.89 (0.72-1.1) |
| United States | 85-89 | 944.57 (891.81-1000.46) | 873.87 (825.31-925.27) | 82.34 (76.97-88.08) | 1.12 (0.9-1.4) |
| United States | 90-94 | 1106.88 (1031.58-1187.67) | 1020.83 (951.64-1095.06) | 104.81 (96.74-113.55) | 1.07 (0.83-1.37) |
| United States | 95+ | 1059.76 (945.11-1188.31) | 972.52 (867.32-1090.48) | 113.33 (100.51-127.78) | 0.6 (0.39-0.92) |
| China | 55-59 | 33.09 (30.35-36.07) | 32.7 (30.01-35.63) | 0.13 (0.12-0.14) | 0.24 (0.21-0.26) |
| China | 60-64 | 94.94 (89.83-100.34) | 94.12 (89.08-99.44) | 0.31 (0.28-0.33) | 0.52 (0.48-0.56) |
| China | 65-69 | 326.01 (313.16-339.39) | 323.41 (310.74-336.59) | 1.14 (1.08-1.21) | 1.46 (1.38-1.55) |
| China | 70-74 | 575.96 (554.83-597.9) | 571.57 (550.74-593.19) | 2.18 (2.06-2.3) | 2.2 (2.07-2.34) |
| China | 75-79 | 770.61 (737.14-805.6) | 764.55 (731.58-799.02) | 3.46 (3.22-3.72) | 2.86 (2.66-3.08) |
| China | 80-84 | 958.74 (914.39-1005.25) | 951.91 (908.2-997.73) | 3.7 (3.39-4.04) | 2.93 (2.69-3.18) |
| China | 85-89 | 1143.28 (1082.57-1207.41) | 1135.58 (1075.74-1198.75) | 4.37 (3.91-4.9) | 2.77 (2.48-3.1) |
| China | 90-94 | 1195.92 (1106.5-1292.57) | 1188.26 (1100.17-1283.41) | 5.5 (4.6-6.57) | 1.75 (1.4-2.2) |
| China | 95+ | 1130.47 (964.01-1325.69) | 1122.42 (958.53-1314.32) | 9.5 (6.89-13.12) | 1.47 (0.83-2.62) |
| Brazil | 55-59 | 13.44 (13.01-13.89) | 13.08 (12.65-13.52) | 0.28 (0.22-0.35) | 0.07 (0.04-0.11) |
| Brazil | 60-64 | 34.24 (33.46-35.04) | 33.44 (32.67-34.23) | 0.64 (0.54-0.76) | 0.13 (0.09-0.19) |
| Brazil | 65-69 | 116.09 (114.19-118.03) | 113.18 (111.3-115.09) | 2.54 (2.26-2.85) | 0.35 (0.26-0.47) |
| Brazil | 70-74 | 224.22 (220.83-227.65) | 217.62 (214.29-220.99) | 6.5 (5.87-7.2) | 0.59 (0.44-0.79) |
| Brazil | 75-79 | 329.33 (323.2-335.57) | 318.02 (312.03-324.12) | 12.68 (11.07-14.51) | 0.93 (0.64-1.35) |
| Brazil | 80-84 | 515.22 (505.18-525.45) | 495.5 (485.74-505.46) | 24.98 (21.56-28.94) | 1.61 (1.09-2.39) |
| Brazil | 85-89 | 828.75 (811.57-846.28) | 793.8 (777.17-810.79) | 50.76 (43.19-59.65) | 2.78 (1.81-4.28) |
| Brazil | 90-94 | 1098.29 (1072.13-1125.09) | 1049.85 (1024.57-1075.74) | 74.78 (61.9-90.34) | 5.09 (3.14-8.26) |
| Brazil | 95+ | 1329.5 (1289.49-1370.76) | 1270.27 (1231.65-1310.09) | 93.94 (73.54-119.98) | 6.93 (3.81-12.61) |
| India | 55-59 | 30.1 (29.69-30.51) | 29.4 (29-29.81) | 0.66 (0.62-0.71) | 0.03 (0.02-0.05) |
| India | 60-64 | 76.48 (75.76-77.2) | 75.17 (74.46-75.89) | 1.23 (1.17-1.3) | 0.08 (0.06-0.09) |
| India | 65-69 | 244.59 (242.88-246.31) | 240.45 (238.75-242.15) | 3.95 (3.8-4.11) | 0.18 (0.15-0.22) |
| India | 70-74 | 463.68 (460.65-466.73) | 455.51 (452.51-458.53) | 7.82 (7.53-8.11) | 0.36 (0.3-0.42) |
| India | 75-79 | 684.47 (678.96-690.03) | 671.91 (666.46-677.4) | 12.12 (11.57-12.7) | 0.54 (0.44-0.66) |
| India | 80-84 | 1108.11 (1098.65-1117.66) | 1088.03 (1078.67-1097.47) | 19.49 (18.53-20.49) | 0.77 (0.62-0.96) |
| India | 85-89 | 1844.73 (1827.43-1862.19) | 1812.48 (1795.37-1829.74) | 31.16 (29.45-32.97) | 1.14 (0.89-1.46) |
| India | 90-94 | 2367.48 (2339.48-2395.82) | 2332.04 (2304.31-2360.1) | 32.19 (29.76-34.82) | 1.22 (0.88-1.7) |
| India | 95+ | 2626.47 (2576.39-2677.52) | 2590.2 (2540.63-2640.75) | 31.78 (27.54-36.66) | 1.05 (0.57-1.92) |
| Ethiopia | 55-59 | 57.54 (55.3-59.87) | 56.56 (54.34-58.87) | 0.69 (0.48-1.01) | 0.29 (0.16-0.52) |
| Ethiopia | 60-64 | 97.05 (93.84-100.37) | 95.73 (92.54-99.03) | 0.93 (0.66-1.31) | 0.38 (0.21-0.66) |
| Ethiopia | 65-69 | 212.27 (206.23-218.49) | 209.57 (203.57-215.75) | 2.12 (1.58-2.83) | 0.56 (0.33-0.95) |
| Ethiopia | 70-74 | 318 (309.19-327.05) | 313.85 (305.1-322.84) | 3.33 (2.5-4.44) | 0.84 (0.5-1.41) |
| Ethiopia | 75-79 | 405.3 (392-419.06) | 400.04 (386.83-413.71) | 4.27 (3-6.08) | 1.05 (0.58-1.93) |
| Ethiopia | 80-84 | 546.69 (527.16-566.94) | 540.24 (520.83-560.37) | 5.12 (3.41-7.71) | 1.22 (0.62-2.42) |
| Ethiopia | 85-89 | 739.49 (708.22-772.13) | 732.22 (701.12-764.7) | 5.07 (2.89-8.9) | 1.55 (0.67-3.6) |
| Ethiopia | 90-94 | 853.92 (800.5-910.9) | 847.33 (794.17-904.05) | 4 (1.31-12.17) | 1.16 (0.23-5.75) |
| Ethiopia | 95+ | 891.01 (781.01-1016.5) | 886.91 (777.33-1011.94) | 3.93 (0.2-77.45) | 1.83 (0.09-38.2) |

**Table S26. Local drift in prevalence rates of HF impairment under CRD cause categories overall and for COPD, ILD&PS, and PC from 1990 to 2021 among adults aged ≥55 years.**

| **Region** | **Age** |  | **Local Drift (%/year)** | |  |
| --- | --- | --- | --- | --- | --- |
|  |  | **CRDs** | **COPD** | **ILD&PS** | **PC** |
| Global | 55-59 | -0.63 (-0.91--0.34) | -0.67 (-0.95--0.38) | 1.66 (1.49-1.83) | -0.65 (-1.01--0.29) |
| Global | 60-64 | -0.65 (-0.82--0.48) | -0.69 (-0.87--0.52) | 2.17 (2.05-2.29) | -1.06 (-1.3--0.83) |
| Global | 65-69 | -0.46 (-0.58--0.35) | -0.51 (-0.63--0.4) | 2.8 (2.71-2.89) | -1.21 (-1.38--1.03) |
| Global | 70-74 | -0.31 (-0.41--0.21) | -0.37 (-0.47--0.27) | 3.39 (3.31-3.47) | -1.13 (-1.28--0.98) |
| Global | 75-79 | -0.24 (-0.34--0.14) | -0.31 (-0.41--0.21) | 3.72 (3.64-3.8) | -0.6 (-0.77--0.44) |
| Global | 80-84 | 0.02 (-0.1-0.13) | -0.06 (-0.17-0.06) | 4.02 (3.93-4.11) | 0 (-0.2-0.2) |
| Global | 85-89 | 0.31 (0.15-0.47) | 0.23 (0.07-0.39) | 4.37 (4.24-4.5) | 0.53 (0.24-0.83) |
| Global | 90-94 | 0.63 (0.34-0.92) | 0.54 (0.25-0.83) | 4.54 (4.31-4.77) | 0.76 (0.21-1.31) |
| Global | 95+ | 0.99 (0.3-1.69) | 0.9 (0.21-1.6) | 4.45 (3.92-4.98) | 0.7 (-0.65-2.08) |
| Low SDI | 55-59 | -0.69 (-0.83--0.55) | -0.71 (-0.85--0.57) | 0.4 (-0.19-1) | -0.84 (-2.17-0.5) |
| Low SDI | 60-64 | -0.62 (-0.71--0.53) | -0.64 (-0.73--0.54) | 0.43 (-0.02-0.88) | -0.98 (-1.99-0.04) |
| Low SDI | 65-69 | -0.49 (-0.56--0.42) | -0.5 (-0.57--0.43) | 0.52 (0.16-0.88) | -1.1 (-1.98--0.2) |
| Low SDI | 70-74 | -0.37 (-0.43--0.31) | -0.38 (-0.44--0.32) | 0.62 (0.28-0.96) | -1.04 (-1.93--0.15) |
| Low SDI | 75-79 | -0.33 (-0.39--0.26) | -0.34 (-0.4--0.27) | 0.63 (0.25-1) | -0.95 (-1.94-0.06) |
| Low SDI | 80-84 | -0.34 (-0.42--0.26) | -0.34 (-0.42--0.26) | 0.59 (0.1-1.08) | -0.81 (-2.12-0.51) |
| Low SDI | 85-89 | -0.36 (-0.47--0.25) | -0.37 (-0.48--0.26) | 0.47 (-0.28-1.23) | -0.74 (-2.71-1.27) |
| Low SDI | 90-94 | -0.23 (-0.43--0.03) | -0.23 (-0.43--0.03) | 0.53 (-1.05-2.13) | -0.29 (-4.51-4.13) |
| Low SDI | 95+ | -0.02 (-0.49-0.44) | -0.03 (-0.49-0.43) | 1.01 (-3.19-5.4) | 3.78 (-13.44-24.43) |
| Low-middle SDI | 55-59 | -0.6 (-0.73--0.48) | -0.63 (-0.76--0.5) | 0.69 (0.27-1.11) | -0.94 (-2.25-0.39) |
| Low-middle SDI | 60-64 | -0.49 (-0.57--0.41) | -0.51 (-0.59--0.43) | 0.82 (0.52-1.12) | -0.81 (-1.74-0.12) |
| Low-middle SDI | 65-69 | -0.26 (-0.32--0.21) | -0.28 (-0.34--0.22) | 1.05 (0.82-1.27) | -0.61 (-1.35-0.13) |
| Low-middle SDI | 70-74 | -0.06 (-0.11--0.02) | -0.08 (-0.13--0.03) | 1.2 (1-1.4) | -0.44 (-1.11-0.25) |
| Low-middle SDI | 75-79 | 0.05 (0-0.1) | 0.04 (-0.01-0.08) | 1.25 (1.04-1.46) | -0.41 (-1.12-0.31) |
| Low-middle SDI | 80-84 | 0.07 (0.01-0.12) | 0.05 (0-0.11) | 1.22 (0.97-1.47) | -0.48 (-1.32-0.37) |
| Low-middle SDI | 85-89 | 0.01 (-0.06-0.08) | 0 (-0.08-0.07) | 1.15 (0.8-1.49) | -0.51 (-1.64-0.63) |
| Low-middle SDI | 90-94 | 0.09 (-0.04-0.21) | 0.08 (-0.05-0.2) | 1.31 (0.66-1.95) | -0.22 (-2.17-1.76) |
| Low-middle SDI | 95+ | 0.13 (-0.16-0.41) | 0.11 (-0.17-0.39) | 1.59 (0.03-3.18) | 0.31 (-4.13-4.97) |
| Middle SDI | 55-59 | -1.6 (-2.05--1.14) | -1.65 (-2.1--1.19) | 2.43 (2.04-2.82) | 0.15 (-0.51-0.82) |
| Middle SDI | 60-64 | -1.65 (-1.92--1.38) | -1.69 (-1.96--1.42) | 2.53 (2.26-2.81) | 0.02 (-0.44-0.49) |
| Middle SDI | 65-69 | -1.42 (-1.59--1.24) | -1.45 (-1.63--1.27) | 2.67 (2.45-2.88) | 0.05 (-0.29-0.4) |
| Middle SDI | 70-74 | -1.22 (-1.36--1.07) | -1.25 (-1.4--1.1) | 2.87 (2.68-3.07) | -0.07 (-0.39-0.26) |
| Middle SDI | 75-79 | -1.08 (-1.23--0.93) | -1.11 (-1.26--0.96) | 2.95 (2.75-3.15) | -0.13 (-0.5-0.24) |
| Middle SDI | 80-84 | -0.96 (-1.14--0.77) | -0.99 (-1.17--0.81) | 2.99 (2.75-3.22) | -0.11 (-0.61-0.4) |
| Middle SDI | 85-89 | -0.72 (-0.99--0.46) | -0.76 (-1.02--0.49) | 3.09 (2.76-3.41) | -0.12 (-0.91-0.67) |
| Middle SDI | 90-94 | -0.33 (-0.83-0.16) | -0.37 (-0.86-0.13) | 3.03 (2.48-3.58) | -0.06 (-1.63-1.53) |
| Middle SDI | 95+ | 0.17 (-1.07-1.42) | 0.13 (-1.1-1.38) | 2.92 (1.71-4.15) | -0.1 (-3.77-3.72) |
| High-middle SDI | 55-59 | -0.84 (-1.31--0.37) | -0.91 (-1.38--0.44) | 3.78 (3.32-4.25) | 0.94 (0.26-1.64) |
| High-middle SDI | 60-64 | -1.04 (-1.32--0.77) | -1.1 (-1.37--0.82) | 3.78 (3.46-4.11) | 0.13 (-0.32-0.59) |
| High-middle SDI | 65-69 | -0.85 (-1.03--0.67) | -0.9 (-1.07--0.72) | 4.25 (4.01-4.49) | -0.32 (-0.64-0) |
| High-middle SDI | 70-74 | -0.84 (-0.99--0.69) | -0.89 (-1.03--0.74) | 4.81 (4.59-5.02) | -0.69 (-0.97--0.4) |
| High-middle SDI | 75-79 | -0.99 (-1.14--0.85) | -1.04 (-1.18--0.9) | 4.96 (4.73-5.19) | -0.69 (-1--0.38) |
| High-middle SDI | 80-84 | -0.71 (-0.88--0.54) | -0.76 (-0.93--0.59) | 4.98 (4.71-5.25) | -0.37 (-0.77-0.03) |
| High-middle SDI | 85-89 | -0.31 (-0.56--0.07) | -0.36 (-0.6--0.12) | 4.95 (4.58-5.34) | 0.02 (-0.6-0.65) |
| High-middle SDI | 90-94 | 0.08 (-0.38-0.54) | 0.03 (-0.43-0.49) | 4.5 (3.83-5.17) | -0.1 (-1.35-1.17) |
| High-middle SDI | 95+ | 0.68 (-0.56-1.93) | 0.64 (-0.59-1.88) | 4.08 (2.52-5.67) | -0.45 (-3.59-2.79) |
| High SDI | 55-59 | 1.31 (1.07-1.56) | 1.34 (1.1-1.59) | 1.86 (1.58-2.14) | -2.68 (-3.39--1.95) |
| High SDI | 60-64 | 1.34 (1.19-1.49) | 1.33 (1.18-1.47) | 2.62 (2.43-2.8) | -2.9 (-3.34--2.46) |
| High SDI | 65-69 | 1.26 (1.15-1.36) | 1.18 (1.08-1.28) | 3.53 (3.4-3.67) | -2.65 (-2.95--2.36) |
| High SDI | 70-74 | 1.21 (1.12-1.29) | 1.07 (0.99-1.16) | 4.36 (4.24-4.48) | -1.83 (-2.08--1.58) |
| High SDI | 75-79 | 1.26 (1.18-1.35) | 1.09 (1-1.17) | 4.89 (4.77-5.02) | -0.46 (-0.71--0.21) |
| High SDI | 80-84 | 1.54 (1.44-1.64) | 1.34 (1.25-1.44) | 5.33 (5.19-5.46) | 0.7 (0.42-0.98) |
| High SDI | 85-89 | 1.88 (1.75-2.01) | 1.68 (1.55-1.8) | 5.6 (5.42-5.78) | 1.41 (1.02-1.81) |
| High SDI | 90-94 | 2.07 (1.85-2.28) | 1.87 (1.65-2.08) | 5.59 (5.27-5.91) | 1.62 (0.9-2.35) |
| High SDI | 95+ | 2.1 (1.62-2.58) | 1.91 (1.44-2.4) | 5.26 (4.52-6) | 1.48 (-0.32-3.3) |
| Andean Latin America | 55-59 | 0.71 (0.22-1.21) | -0.25 (-0.88-0.37) | 2.83 (1.98-3.68) | 0.53 (-3.35-4.56) |
| Andean Latin America | 60-64 | 0.69 (0.39-1) | -0.17 (-0.54-0.2) | 2.83 (2.25-3.42) | 0.61 (-2.05-3.35) |
| Andean Latin America | 65-69 | 0.79 (0.58-1) | -0.04 (-0.29-0.2) | 2.99 (2.57-3.42) | 0.66 (-1.39-2.75) |
| Andean Latin America | 70-74 | 0.95 (0.78-1.12) | 0.13 (-0.07-0.33) | 3.3 (2.93-3.67) | 0.89 (-0.96-2.78) |
| Andean Latin America | 75-79 | 1.14 (0.98-1.3) | 0.32 (0.13-0.5) | 3.72 (3.36-4.09) | 1.33 (-0.61-3.31) |
| Andean Latin America | 80-84 | 1.31 (1.14-1.48) | 0.5 (0.31-0.69) | 4.16 (3.75-4.57) | 1.6 (-0.76-4.01) |
| Andean Latin America | 85-89 | 1.46 (1.25-1.67) | 0.68 (0.46-0.91) | 4.51 (3.98-5.05) | 1.85 (-1.53-5.35) |
| Andean Latin America | 90-94 | 1.57 (1.26-1.88) | 0.88 (0.54-1.22) | 4.59 (3.75-5.44) | 1.25 (-3.67-6.41) |
| Andean Latin America | 95+ | 1.65 (1.04-2.26) | 1.03 (0.37-1.69) | 4.51 (2.83-6.21) | 5.62 (-11.94-26.68) |
| Australasia | 55-59 | 1.37 (1.03-1.71) | 1.26 (0.91-1.61) | 5.04 (3.38-6.73) | 3.22 (-2.32-9.08) |
| Australasia | 60-64 | 1.2 (1-1.39) | 1.06 (0.86-1.26) | 5.41 (4.35-6.47) | 3.16 (-0.03-6.44) |
| Australasia | 65-69 | 1.18 (1.06-1.31) | 1.01 (0.88-1.14) | 5.91 (5.16-6.68) | 3.25 (1.2-5.33) |
| Australasia | 70-74 | 1.35 (1.24-1.45) | 1.13 (1.03-1.24) | 6.53 (5.88-7.18) | 3.87 (2.22-5.55) |
| Australasia | 75-79 | 1.61 (1.5-1.72) | 1.36 (1.25-1.47) | 6.96 (6.32-7.59) | 4.82 (3.19-6.47) |
| Australasia | 80-84 | 1.84 (1.72-1.97) | 1.58 (1.45-1.71) | 7.15 (6.42-7.88) | 5.82 (3.76-7.92) |
| Australasia | 85-89 | 1.93 (1.75-2.12) | 1.67 (1.49-1.86) | 6.69 (5.67-7.73) | 6.32 (3.1-9.65) |
| Australasia | 90-94 | 1.86 (1.53-2.19) | 1.65 (1.32-1.98) | 5.39 (3.6-7.22) | 5.5 (-0.45-11.8) |
| Australasia | 95+ | 1.73 (0.96-2.51) | 1.6 (0.81-2.39) | 3.59 (-0.48-7.82) | 5.36 (-12.25-26.51) |
| Caribbean | 55-59 | 0.94 (0.45-1.44) | 0.91 (0.41-1.41) | 3.01 (-0.2-6.32) | -2.43 (-22.47-22.77) |
| Caribbean | 60-64 | 1.02 (0.71-1.33) | 0.97 (0.66-1.29) | 3.57 (1.34-5.85) | -2.51 (-16.35-13.61) |
| Caribbean | 65-69 | 1.14 (0.93-1.36) | 1.09 (0.88-1.31) | 3.89 (2.25-5.56) | -2.96 (-10.41-5.1) |
| Caribbean | 70-74 | 1.17 (1-1.35) | 1.12 (0.94-1.3) | 4.13 (2.76-5.52) | -3.26 (-10.26-4.28) |
| Caribbean | 75-79 | 1.13 (0.96-1.3) | 1.08 (0.91-1.25) | 4.12 (2.78-5.48) | -2.81 (-8.61-3.35) |
| Caribbean | 80-84 | 1.09 (0.9-1.27) | 1.04 (0.85-1.22) | 4.02 (2.53-5.54) | -2.88 (-8.52-3.11) |
| Caribbean | 85-89 | 1.1 (0.87-1.33) | 1.06 (0.83-1.29) | 3.73 (1.82-5.68) | -4.09 (-10.59-2.88) |
| Caribbean | 90-94 | 1.18 (0.8-1.56) | 1.15 (0.77-1.53) | 3.53 (0.25-6.91) | -4.46 (-14.87-7.21) |
| Caribbean | 95+ | 1.28 (0.39-2.17) | 1.27 (0.38-2.18) | 3.28 (-4.31-11.47) | -6.91 (-25.04-15.62) |
| Central Asia | 55-59 | -2.22 (-2.7--1.75) | -2.25 (-2.73--1.77) | -1.03 (-4.2-2.26) | -1.06 (-8.65-7.17) |
| Central Asia | 60-64 | -1.83 (-2.12--1.54) | -1.85 (-2.14--1.55) | -0.93 (-3.12-1.3) | -0.94 (-5.68-4.05) |
| Central Asia | 65-69 | -1.5 (-1.7--1.29) | -1.51 (-1.71--1.3) | -0.86 (-2.52-0.83) | -0.45 (-4.16-3.39) |
| Central Asia | 70-74 | -1.3 (-1.49--1.11) | -1.31 (-1.51--1.12) | -0.67 (-2.27-0.95) | -0.2 (-3.73-3.46) |
| Central Asia | 75-79 | -1.25 (-1.45--1.04) | -1.26 (-1.46--1.05) | -0.79 (-2.42-0.86) | 0.03 (-4.07-4.3) |
| Central Asia | 80-84 | -1.23 (-1.46--1) | -1.24 (-1.47--1) | -0.96 (-2.69-0.8) | -0.7 (-5.34-4.16) |
| Central Asia | 85-89 | -1.35 (-1.65--1.04) | -1.34 (-1.65--1.04) | -1.41 (-3.51-0.73) | -1.01 (-7.76-6.22) |
| Central Asia | 90-94 | -1.63 (-2.1--1.16) | -1.62 (-2.1--1.15) | -1.98 (-5.09-1.22) | -0.41 (-14.29-15.72) |
| Central Asia | 95+ | -2.23 (-3.26--1.18) | -2.21 (-3.26--1.16) | -2.35 (-8.67-4.4) | -0.68 (-20.92-24.73) |
| Central Europe | 55-59 | 0.8 (0.55-1.05) | 0.79 (0.53-1.04) | 2.48 (1.05-3.93) | -4.85 (-8.39--1.19) |
| Central Europe | 60-64 | 0.88 (0.74-1.03) | 0.86 (0.72-1.01) | 3.07 (2.1-4.04) | -4.93 (-6.98--2.84) |
| Central Europe | 65-69 | 0.88 (0.78-0.98) | 0.85 (0.75-0.95) | 3.74 (3.01-4.47) | -4.69 (-6.09--3.28) |
| Central Europe | 70-74 | 0.83 (0.74-0.91) | 0.79 (0.7-0.87) | 4.33 (3.64-5.02) | -4.32 (-5.54--3.09) |
| Central Europe | 75-79 | 0.56 (0.46-0.66) | 0.51 (0.41-0.62) | 4.32 (3.45-5.19) | -3.98 (-5.35--2.6) |
| Central Europe | 80-84 | 0.22 (0.1-0.34) | 0.18 (0.06-0.3) | 4 (2.9-5.11) | -3.96 (-5.54--2.36) |
| Central Europe | 85-89 | -0.28 (-0.44--0.11) | -0.31 (-0.48--0.15) | 3.29 (1.69-4.91) | -4.34 (-6.56--2.08) |
| Central Europe | 90-94 | -0.87 (-1.17--0.58) | -0.9 (-1.2--0.6) | 2.36 (-0.59-5.4) | -4.73 (-8.65--0.65) |
| Central Europe | 95+ | -1.44 (-2.17--0.71) | -1.48 (-2.21--0.74) | 0.52 (-5.54-6.97) | -1.95 (-14.28-12.15) |
| Central Latin America | 55-59 | -0.3 (-0.5--0.1) | -0.47 (-0.68--0.26) | 3.05 (2.2-3.9) | -3.51 (-6.72--0.19) |
| Central Latin America | 60-64 | -0.3 (-0.43--0.18) | -0.45 (-0.58--0.32) | 3.23 (2.6-3.86) | -3.7 (-5.82--1.52) |
| Central Latin America | 65-69 | -0.22 (-0.31--0.13) | -0.34 (-0.43--0.25) | 3.35 (2.85-3.85) | -3.71 (-5.22--2.17) |
| Central Latin America | 70-74 | -0.07 (-0.14-0.01) | -0.17 (-0.25--0.1) | 3.41 (2.96-3.87) | -3.76 (-5.06--2.44) |
| Central Latin America | 75-79 | 0.1 (0.02-0.17) | 0.01 (-0.06-0.08) | 3.3 (2.83-3.77) | -3.66 (-4.92--2.38) |
| Central Latin America | 80-84 | 0.26 (0.18-0.33) | 0.19 (0.12-0.27) | 3.19 (2.64-3.73) | -3.5 (-4.9--2.08) |
| Central Latin America | 85-89 | 0.4 (0.31-0.49) | 0.35 (0.26-0.44) | 3.07 (2.36-3.79) | -3.39 (-5.18--1.56) |
| Central Latin America | 90-94 | 0.5 (0.35-0.64) | 0.46 (0.31-0.61) | 2.88 (1.64-4.14) | -3.41 (-6.41--0.31) |
| Central Latin America | 95+ | 0.57 (0.26-0.89) | 0.55 (0.23-0.87) | 2.82 (-0.1-5.83) | -3.71 (-9.61-2.58) |
| Central Sub-Saharan Africa | 55-59 | -0.83 (-1.08--0.57) | -0.84 (-1.1--0.58) | 0.11 (-1.96-2.23) | -0.34 (-3.78-3.22) |
| Central Sub-Saharan Africa | 60-64 | -0.85 (-1.04--0.67) | -0.87 (-1.05--0.68) | 0.07 (-1.53-1.7) | -0.35 (-2.95-2.33) |
| Central Sub-Saharan Africa | 65-69 | -0.83 (-0.97--0.68) | -0.84 (-0.99--0.69) | 0.11 (-1.24-1.48) | -0.43 (-2.71-1.9) |
| Central Sub-Saharan Africa | 70-74 | -0.76 (-0.9--0.62) | -0.77 (-0.91--0.63) | 0.18 (-1.12-1.51) | -0.59 (-2.88-1.75) |
| Central Sub-Saharan Africa | 75-79 | -0.7 (-0.85--0.54) | -0.71 (-0.86--0.55) | 0.28 (-1.2-1.78) | -0.7 (-3.3-1.96) |
| Central Sub-Saharan Africa | 80-84 | -0.61 (-0.81--0.41) | -0.62 (-0.82--0.42) | 0.31 (-1.74-2.41) | -0.39 (-4.11-3.48) |
| Central Sub-Saharan Africa | 85-89 | -0.54 (-0.85--0.22) | -0.54 (-0.86--0.23) | 0.02 (-3.5-3.67) | -0.69 (-6.59-5.58) |
| Central Sub-Saharan Africa | 90-94 | -0.54 (-1.1-0.03) | -0.55 (-1.12-0.02) | -1.56 (-7.59-4.87) | 0.1 (-13.02-15.19) |
| Central Sub-Saharan Africa | 95+ | -0.63 (-1.82-0.58) | -0.64 (-1.83-0.56) | -2.96 (-20.58-18.57) | -1.59 (-21.32-23.08) |
| East Asia | 55-59 | -1.85 (-2.4--1.31) | -1.9 (-2.44--1.36) | 4.36 (3.69-5.04) | 1.08 (0.48-1.69) |
| East Asia | 60-64 | -2.1 (-2.41--1.78) | -2.13 (-2.44--1.82) | 4.12 (3.64-4.59) | 0.42 (0.02-0.83) |
| East Asia | 65-69 | -2.02 (-2.21--1.83) | -2.04 (-2.24--1.85) | 3.85 (3.48-4.21) | -0.05 (-0.34-0.25) |
| East Asia | 70-74 | -1.84 (-2--1.68) | -1.86 (-2.02--1.7) | 3.68 (3.34-4.01) | -0.16 (-0.44-0.11) |
| East Asia | 75-79 | -1.77 (-1.93--1.61) | -1.78 (-1.94--1.62) | 3.64 (3.25-4.02) | -0.03 (-0.35-0.29) |
| East Asia | 80-84 | -1.82 (-2.01--1.62) | -1.83 (-2.02--1.64) | 3.61 (3.04-4.18) | 0.21 (-0.26-0.68) |
| East Asia | 85-89 | -1.92 (-2.22--1.62) | -1.93 (-2.23--1.64) | 3.65 (2.65-4.67) | 0.5 (-0.38-1.39) |
| East Asia | 90-94 | -2.01 (-2.61--1.4) | -2.01 (-2.61--1.41) | 3.42 (1.35-5.53) | 0.69 (-1.62-3.05) |
| East Asia | 95+ | -1.99 (-3.75--0.2) | -2 (-3.74--0.22) | 3.5 (-1.86-9.15) | 0.51 (-6.14-7.63) |
| Eastern Europe | 55-59 | -3.03 (-3.28--2.78) | -3.04 (-3.29--2.79) | -2.5 (-4.42--0.54) | -4.43 (-8.8-0.16) |
| Eastern Europe | 60-64 | -3.02 (-3.17--2.88) | -3.03 (-3.18--2.89) | -2.13 (-3.39--0.86) | -4.04 (-6.79--1.21) |
| Eastern Europe | 65-69 | -2.81 (-2.91--2.72) | -2.81 (-2.91--2.72) | -2.18 (-3.09--1.25) | -3.69 (-5.52--1.82) |
| Eastern Europe | 70-74 | -2.45 (-2.53--2.36) | -2.45 (-2.53--2.36) | -2.41 (-3.28--1.54) | -2.99 (-4.59--1.36) |
| Eastern Europe | 75-79 | -2.22 (-2.31--2.13) | -2.21 (-2.3--2.12) | -3.13 (-4.06--2.2) | -2.17 (-3.81--0.5) |
| Eastern Europe | 80-84 | -2.11 (-2.21--2.02) | -2.1 (-2.2--2) | -3.96 (-4.95--2.95) | -1.3 (-3.18-0.62) |
| Eastern Europe | 85-89 | -2.29 (-2.42--2.16) | -2.27 (-2.4--2.14) | -4.87 (-6.16--3.57) | -0.86 (-3.58-1.94) |
| Eastern Europe | 90-94 | -2.61 (-2.83--2.39) | -2.59 (-2.81--2.37) | -5.21 (-7.31--3.05) | 0.81 (-4.8-6.75) |
| Eastern Europe | 95+ | -2.95 (-3.48--2.41) | -2.93 (-3.47--2.39) | -4.85 (-9.39--0.08) | 5.07 (-12.52-26.19) |
| Eastern Sub-Saharan Africa | 55-59 | -1.24 (-1.37--1.11) | -1.26 (-1.38--1.13) | 0.07 (-1-1.15) | -1.65 (-3.39-0.13) |
| Eastern Sub-Saharan Africa | 60-64 | -1.27 (-1.36--1.18) | -1.29 (-1.38--1.2) | -0.02 (-0.85-0.81) | -1.65 (-2.97--0.3) |
| Eastern Sub-Saharan Africa | 65-69 | -1.25 (-1.32--1.18) | -1.26 (-1.33--1.19) | -0.05 (-0.75-0.66) | -1.64 (-2.83--0.45) |
| Eastern Sub-Saharan Africa | 70-74 | -1.19 (-1.25--1.12) | -1.2 (-1.26--1.13) | -0.03 (-0.72-0.66) | -1.63 (-2.81--0.43) |
| Eastern Sub-Saharan Africa | 75-79 | -1.11 (-1.18--1.04) | -1.12 (-1.19--1.04) | -0.03 (-0.83-0.78) | -1.52 (-2.86--0.16) |
| Eastern Sub-Saharan Africa | 80-84 | -1.02 (-1.11--0.93) | -1.03 (-1.12--0.94) | -0.1 (-1.22-1.04) | -1.4 (-3.2-0.43) |
| Eastern Sub-Saharan Africa | 85-89 | -0.91 (-1.05--0.78) | -0.92 (-1.05--0.78) | 0.04 (-1.92-2.03) | -1.16 (-4.09-1.85) |
| Eastern Sub-Saharan Africa | 90-94 | -0.79 (-1.03--0.55) | -0.79 (-1.03--0.55) | -0.03 (-4.25-4.37) | -1.56 (-7.44-4.69) |
| Eastern Sub-Saharan Africa | 95+ | -0.67 (-1.24--0.1) | -0.67 (-1.24--0.09) | 3.28 (-13.88-23.87) | -3.24 (-20.78-18.19) |
| High-income Asia Pacific | 55-59 | 0.91 (0.64-1.17) | 1 (0.74-1.27) | 1.29 (0.78-1.81) | -3.07 (-4.29--1.82) |
| High-income Asia Pacific | 60-64 | 0.88 (0.71-1.04) | 0.79 (0.62-0.95) | 1.83 (1.47-2.19) | -3.2 (-3.99--2.4) |
| High-income Asia Pacific | 65-69 | 0.77 (0.65-0.9) | 0.43 (0.31-0.55) | 2.57 (2.3-2.83) | -2.87 (-3.46--2.28) |
| High-income Asia Pacific | 70-74 | 0.9 (0.79-1) | 0.37 (0.27-0.46) | 3.35 (3.11-3.6) | -1.62 (-2.16--1.08) |
| High-income Asia Pacific | 75-79 | 1.08 (0.98-1.18) | 0.46 (0.36-0.55) | 3.96 (3.71-4.21) | -0.17 (-0.74-0.4) |
| High-income Asia Pacific | 80-84 | 1.11 (1.01-1.2) | 0.48 (0.39-0.57) | 4.22 (3.96-4.49) | 0.81 (0.17-1.45) |
| High-income Asia Pacific | 85-89 | 0.98 (0.87-1.09) | 0.41 (0.31-0.5) | 4.22 (3.9-4.54) | 1.22 (0.37-2.08) |
| High-income Asia Pacific | 90-94 | 0.77 (0.6-0.93) | 0.28 (0.13-0.43) | 4.1 (3.54-4.67) | 1.24 (-0.33-2.83) |
| High-income Asia Pacific | 95+ | 0.46 (0.09-0.84) | 0.08 (-0.25-0.41) | 3.66 (2.29-5.06) | 0.59 (-3.2-4.52) |
| High-income North America | 55-59 | 0.25 (-0.26-0.76) | 0.26 (-0.24-0.77) | 0.55 (-0.02-1.12) | -4.89 (-7.52--2.18) |
| High-income North America | 60-64 | 0.43 (0.13-0.74) | 0.41 (0.11-0.72) | 1.46 (1.09-1.83) | -4.48 (-5.87--3.07) |
| High-income North America | 65-69 | 0.75 (0.54-0.96) | 0.69 (0.48-0.9) | 2.53 (2.27-2.8) | -3.78 (-4.62--2.94) |
| High-income North America | 70-74 | 1.2 (1.02-1.37) | 1.11 (0.93-1.28) | 3.47 (3.25-3.7) | -2.92 (-3.54--2.3) |
| High-income North America | 75-79 | 1.84 (1.66-2.02) | 1.73 (1.55-1.91) | 4.34 (4.12-4.57) | -1.83 (-2.38--1.27) |
| High-income North America | 80-84 | 2.73 (2.51-2.95) | 2.61 (2.4-2.83) | 5.25 (5-5.51) | -0.5 (-1.09-0.09) |
| High-income North America | 85-89 | 3.72 (3.4-4.03) | 3.61 (3.29-3.92) | 5.96 (5.6-6.33) | 0.78 (-0.01-1.59) |
| High-income North America | 90-94 | 4.57 (4.01-5.14) | 4.48 (3.92-5.05) | 6.32 (5.69-6.97) | 1.71 (0.31-3.13) |
| High-income North America | 95+ | 5.07 (3.78-6.38) | 5 (3.72-6.31) | 6.2 (4.8-7.62) | 2.17 (-1.36-5.82) |
| North Africa and Middle East | 55-59 | 0.57 (0.42-0.72) | 0.54 (0.38-0.69) | 3.53 (2.25-4.83) | -0.21 (-3.23-2.91) |
| North Africa and Middle East | 60-64 | 0.55 (0.44-0.66) | 0.52 (0.41-0.62) | 3.45 (2.43-4.47) | -0.05 (-2.35-2.3) |
| North Africa and Middle East | 65-69 | 0.6 (0.52-0.69) | 0.58 (0.49-0.66) | 3.29 (2.43-4.15) | 0.31 (-1.58-2.24) |
| North Africa and Middle East | 70-74 | 0.76 (0.68-0.83) | 0.73 (0.65-0.81) | 3.23 (2.4-4.07) | 0.7 (-1.21-2.64) |
| North Africa and Middle East | 75-79 | 0.88 (0.8-0.96) | 0.86 (0.78-0.95) | 3 (2.06-3.94) | 1.11 (-1.06-3.33) |
| North Africa and Middle East | 80-84 | 0.86 (0.76-0.96) | 0.85 (0.75-0.95) | 2.48 (1.37-3.6) | 1.28 (-1.39-4.03) |
| North Africa and Middle East | 85-89 | 0.76 (0.63-0.89) | 0.75 (0.62-0.88) | 2 (0.57-3.45) | 1.32 (-2.19-4.95) |
| North Africa and Middle East | 90-94 | 0.63 (0.42-0.84) | 0.62 (0.4-0.83) | 1.82 (-0.31-4) | 1.92 (-3.85-8.03) |
| North Africa and Middle East | 95+ | 0.65 (0.21-1.09) | 0.63 (0.19-1.08) | 1.75 (-2.1-5.76) | 5.76 (-11.9-26.96) |
| Oceania | 55-59 | -0.24 (-1.11-0.64) | -0.24 (-1.12-0.64) | 0.84 (-8.52-11.15) | -3.44 (-24.5-23.51) |
| Oceania | 60-64 | -0.25 (-0.77-0.27) | -0.26 (-0.78-0.27) | 0.82 (-5.21-7.24) | -3.24 (-20.1-17.18) |
| Oceania | 65-69 | -0.35 (-0.7-0) | -0.35 (-0.71-0) | 0.87 (-3.75-5.71) | -2.81 (-18.34-15.68) |
| Oceania | 70-74 | -0.45 (-0.75--0.16) | -0.46 (-0.75--0.16) | 0.51 (-3.43-4.6) | -2.45 (-17.58-15.47) |
| Oceania | 75-79 | -0.51 (-0.82--0.19) | -0.51 (-0.82--0.19) | 0.35 (-4.14-5.05) | -2.48 (-17.53-15.33) |
| Oceania | 80-84 | -0.37 (-0.77-0.03) | -0.38 (-0.78-0.02) | 0.13 (-5.98-6.64) | -3.24 (-18.25-14.53) |
| Oceania | 85-89 | -0.13 (-0.73-0.49) | -0.13 (-0.74-0.49) | 0.14 (-11.37-13.14) | -4.57 (-19.82-13.59) |
| Oceania | 90-94 | 0.1 (-1.08-1.3) | 0.09 (-1.1-1.28) | -2.76 (-18.01-15.33) | -6.04 (-22.41-13.79) |
| Oceania | 95+ | -0.2 (-2.92-2.6) | -0.11 (-2.89-2.74) | -5.03 (-24.89-20.08) | -6.81 (-27.14-19.19) |
| South Asia | 55-59 | -0.48 (-0.6--0.37) | -0.51 (-0.63--0.4) | 0.89 (0.51-1.28) | -0.88 (-2.63-0.91) |
| South Asia | 60-64 | -0.39 (-0.46--0.32) | -0.41 (-0.48--0.34) | 0.94 (0.67-1.21) | -0.77 (-1.9-0.36) |
| South Asia | 65-69 | -0.23 (-0.28--0.18) | -0.25 (-0.3--0.2) | 1.03 (0.82-1.23) | -0.64 (-1.47-0.19) |
| South Asia | 70-74 | -0.15 (-0.19--0.1) | -0.16 (-0.21--0.12) | 1.03 (0.85-1.21) | -0.59 (-1.3-0.14) |
| South Asia | 75-79 | -0.12 (-0.16--0.08) | -0.13 (-0.18--0.09) | 0.98 (0.79-1.16) | -0.61 (-1.35-0.13) |
| South Asia | 80-84 | -0.14 (-0.19--0.09) | -0.15 (-0.2--0.1) | 0.89 (0.67-1.11) | -0.74 (-1.6-0.13) |
| South Asia | 85-89 | -0.16 (-0.22--0.09) | -0.17 (-0.23--0.1) | 0.81 (0.5-1.12) | -0.9 (-2.06-0.27) |
| South Asia | 90-94 | -0.03 (-0.15-0.08) | -0.04 (-0.15-0.07) | 0.89 (0.29-1.49) | -1.06 (-3.14-1.05) |
| South Asia | 95+ | 0.15 (-0.11-0.42) | 0.15 (-0.12-0.41) | 1.02 (-0.49-2.55) | -0.48 (-6.28-5.68) |
| Southeast Asia | 55-59 | -0.95 (-1.07--0.82) | -0.96 (-1.08--0.83) | 1.46 (-0.3-3.25) | -2.68 (-9.61-4.77) |
| Southeast Asia | 60-64 | -0.99 (-1.06--0.92) | -1 (-1.07--0.93) | 1.7 (0.41-2.99) | -1.1 (-5.88-3.91) |
| Southeast Asia | 65-69 | -0.93 (-0.98--0.88) | -0.94 (-0.99--0.89) | 1.94 (0.96-2.93) | -1.18 (-5.02-2.83) |
| Southeast Asia | 70-74 | -0.76 (-0.8--0.71) | -0.76 (-0.81--0.72) | 2.15 (1.27-3.03) | -0.82 (-4.34-2.83) |
| Southeast Asia | 75-79 | -0.53 (-0.57--0.49) | -0.54 (-0.58--0.49) | 2.12 (1.18-3.07) | -0.57 (-4.38-3.38) |
| Southeast Asia | 80-84 | -0.29 (-0.34--0.24) | -0.29 (-0.34--0.25) | 2.14 (1.02-3.27) | -0.54 (-4.66-3.75) |
| Southeast Asia | 85-89 | -0.05 (-0.12-0.01) | -0.06 (-0.12-0.01) | 2.27 (0.83-3.72) | 0.06 (-4.93-5.31) |
| Southeast Asia | 90-94 | 0.18 (0.07-0.29) | 0.18 (0.07-0.29) | 2.44 (0.16-4.78) | -0.9 (-7.48-6.16) |
| Southeast Asia | 95+ | 0.55 (0.31-0.8) | 0.55 (0.3-0.8) | 2.11 (-2.47-6.92) | -1.5 (-18.93-19.67) |
| Southern Latin America | 55-59 | 1.45 (1.08-1.82) | 1.31 (0.93-1.7) | 3.35 (2.07-4.64) | -1.95 (-6.82-3.18) |
| Southern Latin America | 60-64 | 1.59 (1.35-1.82) | 1.45 (1.2-1.69) | 3.61 (2.74-4.48) | -1.36 (-4.55-1.93) |
| Southern Latin America | 65-69 | 1.61 (1.44-1.78) | 1.44 (1.26-1.61) | 3.96 (3.32-4.61) | -0.82 (-3.09-1.5) |
| Southern Latin America | 70-74 | 1.55 (1.4-1.69) | 1.33 (1.18-1.48) | 4.35 (3.78-4.92) | -0.01 (-1.92-1.94) |
| Southern Latin America | 75-79 | 1.39 (1.25-1.53) | 1.16 (1.02-1.31) | 4.33 (3.76-4.9) | 0.59 (-1.28-2.5) |
| Southern Latin America | 80-84 | 1.19 (1.04-1.34) | 0.97 (0.82-1.13) | 4.14 (3.51-4.78) | 0.65 (-1.36-2.71) |
| Southern Latin America | 85-89 | 1.01 (0.83-1.18) | 0.8 (0.62-0.99) | 3.92 (3.13-4.71) | 0.35 (-2.08-2.84) |
| Southern Latin America | 90-94 | 0.95 (0.68-1.22) | 0.77 (0.49-1.05) | 3.74 (2.45-5.05) | -0.27 (-4-3.61) |
| Southern Latin America | 95+ | 1.12 (0.53-1.72) | 0.97 (0.36-1.57) | 3.89 (0.77-7.11) | -1.41 (-8.27-5.97) |
| Southern Sub-Saharan Africa | 55-59 | -0.74 (-1.08--0.4) | -0.74 (-1.09--0.4) | -0.55 (-1.64-0.56) | -1.82 (-4.16-0.58) |
| Southern Sub-Saharan Africa | 60-64 | -0.6 (-0.84--0.36) | -0.6 (-0.83--0.36) | -0.55 (-1.37-0.27) | -1.56 (-3.31-0.23) |
| Southern Sub-Saharan Africa | 65-69 | -0.45 (-0.63--0.26) | -0.45 (-0.63--0.26) | -0.37 (-1.03-0.29) | -1.12 (-2.63-0.42) |
| Southern Sub-Saharan Africa | 70-74 | -0.39 (-0.56--0.21) | -0.39 (-0.56--0.21) | -0.3 (-0.9-0.3) | -0.9 (-2.38-0.61) |
| Southern Sub-Saharan Africa | 75-79 | -0.41 (-0.59--0.23) | -0.41 (-0.59--0.23) | -0.25 (-0.88-0.4) | -0.75 (-2.33-0.85) |
| Southern Sub-Saharan Africa | 80-84 | -0.51 (-0.72--0.29) | -0.51 (-0.72--0.3) | -0.3 (-1.09-0.5) | -0.58 (-2.51-1.4) |
| Southern Sub-Saharan Africa | 85-89 | -0.67 (-0.95--0.4) | -0.68 (-0.95--0.41) | -0.46 (-1.56-0.67) | -0.77 (-3.46-2) |
| Southern Sub-Saharan Africa | 90-94 | -0.85 (-1.28--0.43) | -0.86 (-1.28--0.43) | -0.56 (-2.54-1.45) | -0.72 (-5.75-4.58) |
| Southern Sub-Saharan Africa | 95+ | -0.97 (-1.88--0.06) | -0.98 (-1.88--0.07) | -0.63 (-5.19-4.14) | -0.49 (-18.4-21.35) |
| Tropical Latin America | 55-59 | 0.56 (0.36-0.75) | 0.49 (0.29-0.68) | 4.46 (3.14-5.8) | 1.24 (-1.71-4.28) |
| Tropical Latin America | 60-64 | 0.42 (0.3-0.54) | 0.35 (0.22-0.47) | 4.62 (3.67-5.58) | 1.51 (-0.61-3.68) |
| Tropical Latin America | 65-69 | 0.34 (0.26-0.43) | 0.27 (0.18-0.35) | 4.88 (4.15-5.62) | 1.87 (0.18-3.59) |
| Tropical Latin America | 70-74 | 0.34 (0.27-0.41) | 0.26 (0.19-0.33) | 4.99 (4.35-5.64) | 2.09 (0.54-3.66) |
| Tropical Latin America | 75-79 | 0.32 (0.25-0.39) | 0.24 (0.17-0.31) | 4.95 (4.31-5.59) | 2.06 (0.5-3.64) |
| Tropical Latin America | 80-84 | 0.26 (0.18-0.33) | 0.18 (0.1-0.26) | 4.75 (4.06-5.45) | 1.9 (0.21-3.62) |
| Tropical Latin America | 85-89 | 0.16 (0.07-0.26) | 0.09 (-0.01-0.18) | 4.55 (3.64-5.46) | 1.68 (-0.42-3.82) |
| Tropical Latin America | 90-94 | 0.09 (-0.07-0.24) | 0.02 (-0.14-0.17) | 4.39 (2.78-6.04) | 1.66 (-1.68-5.12) |
| Tropical Latin America | 95+ | 0.07 (-0.28-0.41) | 0.01 (-0.34-0.35) | 4.11 (0.24-8.13) | 0.28 (-5.7-6.63) |
| Western Europe | 55-59 | 2.75 (2.44-3.06) | 2.72 (2.41-3.03) | 4.76 (4.36-5.17) | -3.12 (-4.31--1.93) |
| Western Europe | 60-64 | 2.73 (2.54-2.92) | 2.68 (2.49-2.87) | 5.22 (4.95-5.5) | -3.54 (-4.24--2.83) |
| Western Europe | 65-69 | 2.52 (2.39-2.65) | 2.43 (2.3-2.56) | 5.8 (5.6-6.01) | -3.62 (-4.07--3.18) |
| Western Europe | 70-74 | 2.27 (2.16-2.38) | 2.12 (2.01-2.24) | 6.38 (6.2-6.55) | -2.95 (-3.29--2.61) |
| Western Europe | 75-79 | 2.01 (1.9-2.11) | 1.82 (1.71-1.93) | 6.77 (6.59-6.95) | -1.45 (-1.78--1.13) |
| Western Europe | 80-84 | 1.68 (1.56-1.79) | 1.48 (1.36-1.59) | 6.8 (6.6-7) | -0.12 (-0.47-0.24) |
| Western Europe | 85-89 | 1.35 (1.21-1.49) | 1.17 (1.03-1.3) | 6.36 (6.09-6.63) | 0.69 (0.2-1.18) |
| Western Europe | 90-94 | 0.97 (0.75-1.2) | 0.83 (0.61-1.05) | 5.31 (4.85-5.77) | 0.66 (-0.24-1.58) |
| Western Europe | 95+ | 0.63 (0.13-1.12) | 0.52 (0.03-1.01) | 4.05 (3.01-5.11) | -0.01 (-2.23-2.26) |
| Western Sub-Saharan Africa | 55-59 | -0.81 (-0.95--0.68) | -0.83 (-0.96--0.69) | -0.47 (-1.2-0.27) | -0.77 (-2.53-1.02) |
| Western Sub-Saharan Africa | 60-64 | -0.88 (-0.98--0.78) | -0.89 (-0.99--0.79) | -0.6 (-1.19-0) | -0.69 (-2.13-0.76) |
| Western Sub-Saharan Africa | 65-69 | -0.93 (-1--0.85) | -0.93 (-1.01--0.85) | -0.65 (-1.18--0.13) | -0.55 (-2-0.91) |
| Western Sub-Saharan Africa | 70-74 | -0.99 (-1.06--0.91) | -1 (-1.07--0.92) | -0.57 (-1.11--0.04) | -0.34 (-1.98-1.32) |
| Western Sub-Saharan Africa | 75-79 | -1.05 (-1.12--0.97) | -1.06 (-1.14--0.98) | -0.46 (-1.09-0.18) | -0.25 (-2.24-1.77) |
| Western Sub-Saharan Africa | 80-84 | -1.08 (-1.17--0.99) | -1.09 (-1.19--1) | -0.23 (-1.14-0.68) | -0.21 (-2.91-2.57) |
| Western Sub-Saharan Africa | 85-89 | -1.06 (-1.19--0.94) | -1.07 (-1.2--0.95) | 0.08 (-1.52-1.71) | -0.85 (-4.8-3.27) |
| Western Sub-Saharan Africa | 90-94 | -0.98 (-1.19--0.78) | -0.99 (-1.2--0.79) | -0.04 (-3.22-3.23) | 1.62 (-11.36-16.51) |
| Western Sub-Saharan Africa | 95+ | -0.87 (-1.28--0.45) | -0.88 (-1.29--0.46) | -0.98 (-7.25-5.71) | -0.1 (-20.02-24.78) |
| **Five Countries** |  |  |  |  |  |
| United States | 55-59 | 0.04 (-0.52-0.6) | 0.05 (-0.5-0.61) | 0.19 (-0.46-0.85) | -5.24 (-8.19--2.2) |
| United States | 60-64 | 0.29 (-0.05-0.63) | 0.27 (-0.06-0.61) | 1.18 (0.75-1.6) | -4.75 (-6.29--3.19) |
| United States | 65-69 | 0.65 (0.42-0.89) | 0.61 (0.38-0.84) | 2.21 (1.91-2.51) | -4.14 (-5.06--3.22) |
| United States | 70-74 | 1.12 (0.93-1.32) | 1.06 (0.86-1.25) | 3.08 (2.82-3.33) | -3.47 (-4.15--2.79) |
| United States | 75-79 | 1.76 (1.56-1.96) | 1.68 (1.49-1.88) | 3.9 (3.65-4.15) | -2.51 (-3.11--1.91) |
| United States | 80-84 | 2.65 (2.41-2.89) | 2.56 (2.33-2.8) | 4.79 (4.51-5.08) | -1.21 (-1.84--0.58) |
| United States | 85-89 | 3.64 (3.3-3.98) | 3.56 (3.21-3.9) | 5.53 (5.14-5.93) | 0.17 (-0.66-1.02) |
| United States | 90-94 | 4.53 (3.91-5.15) | 4.46 (3.85-5.08) | 5.96 (5.27-6.66) | 1.28 (-0.17-2.76) |
| United States | 95+ | 5.01 (3.6-6.44) | 4.97 (3.56-6.4) | 5.84 (4.32-7.38) | 1.65 (-1.91-5.33) |
| China | 55-59 | -1.87 (-2.42--1.32) | -1.92 (-2.46--1.37) | 4.32 (3.63-5.02) | 1.18 (0.55-1.82) |
| China | 60-64 | -2.12 (-2.43--1.81) | -2.15 (-2.47--1.84) | 4.03 (3.53-4.52) | 0.53 (0.1-0.96) |
| China | 65-69 | -2.06 (-2.25--1.86) | -2.08 (-2.27--1.88) | 3.78 (3.41-4.16) | 0.05 (-0.26-0.37) |
| China | 70-74 | -1.88 (-2.04--1.72) | -1.89 (-2.05--1.73) | 3.62 (3.27-3.96) | -0.1 (-0.4-0.19) |
| China | 75-79 | -1.8 (-1.96--1.64) | -1.81 (-1.97--1.65) | 3.5 (3.1-3.9) | -0.04 (-0.38-0.3) |
| China | 80-84 | -1.85 (-2.05--1.65) | -1.86 (-2.06--1.67) | 3.37 (2.78-3.97) | 0.12 (-0.38-0.62) |
| China | 85-89 | -1.96 (-2.27--1.66) | -1.97 (-2.27--1.67) | 3.2 (2.14-4.27) | 0.32 (-0.62-1.28) |
| China | 90-94 | -2.07 (-2.69--1.45) | -2.08 (-2.69--1.46) | 2.81 (0.6-5.08) | 0.42 (-2.14-3.04) |
| China | 95+ | -2.11 (-3.93--0.25) | -2.11 (-3.92--0.27) | 2.63 (-3.19-8.79) | -0.81 (-7.67-6.56) |
| Brazil | 55-59 | 0.56 (0.36-0.75) | 0.49 (0.29-0.69) | 4.53 (3.19-5.88) | 1.34 (-1.66-4.44) |
| Brazil | 60-64 | 0.42 (0.29-0.54) | 0.35 (0.22-0.47) | 4.69 (3.73-5.66) | 1.6 (-0.57-3.81) |
| Brazil | 65-69 | 0.34 (0.25-0.42) | 0.26 (0.17-0.35) | 4.95 (4.21-5.7) | 1.95 (0.23-3.7) |
| Brazil | 70-74 | 0.33 (0.25-0.4) | 0.24 (0.17-0.32) | 5.06 (4.41-5.72) | 2.17 (0.59-3.77) |
| Brazil | 75-79 | 0.31 (0.24-0.38) | 0.23 (0.15-0.3) | 5.01 (4.36-5.66) | 2.19 (0.59-3.81) |
| Brazil | 80-84 | 0.24 (0.17-0.32) | 0.16 (0.09-0.24) | 4.81 (4.11-5.52) | 2.1 (0.36-3.86) |
| Brazil | 85-89 | 0.14 (0.05-0.24) | 0.07 (-0.03-0.16) | 4.59 (3.67-5.51) | 1.84 (-0.31-4.03) |
| Brazil | 90-94 | 0.05 (-0.1-0.21) | -0.02 (-0.18-0.14) | 4.41 (2.76-6.08) | 1.61 (-1.74-5.09) |
| Brazil | 95+ | -0.01 (-0.36-0.35) | -0.07 (-0.42-0.29) | 3.89 (0.02-7.9) | -0.05 (-6.01-6.3) |
| India | 55-59 | -0.42 (-0.5--0.33) | -0.44 (-0.53--0.36) | 0.92 (0.5-1.34) | -0.8 (-2.71-1.15) |
| India | 60-64 | -0.32 (-0.37--0.27) | -0.34 (-0.39--0.29) | 0.97 (0.68-1.26) | -0.69 (-1.92-0.55) |
| India | 65-69 | -0.16 (-0.2--0.13) | -0.18 (-0.22--0.14) | 1.05 (0.83-1.27) | -0.55 (-1.45-0.36) |
| India | 70-74 | -0.08 (-0.11--0.05) | -0.1 (-0.13--0.07) | 1.05 (0.85-1.24) | -0.49 (-1.28-0.3) |
| India | 75-79 | -0.08 (-0.11--0.05) | -0.1 (-0.13--0.07) | 0.97 (0.76-1.17) | -0.57 (-1.38-0.24) |
| India | 80-84 | -0.16 (-0.2--0.13) | -0.18 (-0.21--0.14) | 0.84 (0.6-1.08) | -0.79 (-1.74-0.17) |
| India | 85-89 | -0.24 (-0.29--0.19) | -0.25 (-0.3--0.2) | 0.72 (0.39-1.06) | -1.02 (-2.29-0.28) |
| India | 90-94 | -0.2 (-0.28--0.11) | -0.21 (-0.29--0.12) | 0.75 (0.08-1.41) | -1.08 (-3.46-1.36) |
| India | 95+ | -0.06 (-0.27-0.15) | -0.07 (-0.28-0.14) | 0.85 (-0.86-2.59) | -1.25 (-7.07-4.94) |
| Ethiopia | 55-59 | -1.51 (-1.76--1.26) | -1.54 (-1.79--1.28) | 1.07 (-1.21-3.4) | -2.17 (-5.87-1.68) |
| Ethiopia | 60-64 | -1.59 (-1.76--1.41) | -1.61 (-1.79--1.43) | 0.79 (-0.98-2.6) | -2 (-4.72-0.8) |
| Ethiopia | 65-69 | -1.57 (-1.7--1.43) | -1.58 (-1.72--1.45) | 0.56 (-0.93-2.08) | -2.21 (-4.55-0.19) |
| Ethiopia | 70-74 | -1.5 (-1.63--1.38) | -1.52 (-1.64--1.39) | 0.34 (-1.11-1.82) | -1.99 (-4.26-0.34) |
| Ethiopia | 75-79 | -1.4 (-1.54--1.27) | -1.41 (-1.55--1.28) | 0.09 (-1.58-1.78) | -1.55 (-4.09-1.05) |
| Ethiopia | 80-84 | -1.38 (-1.56--1.2) | -1.39 (-1.57--1.21) | 0.19 (-2.46-2.92) | -1.13 (-4.84-2.73) |
| Ethiopia | 85-89 | -1.34 (-1.64--1.04) | -1.35 (-1.65--1.04) | 0.34 (-5.15-6.14) | -1.42 (-7.62-5.2) |
| Ethiopia | 90-94 | -1.16 (-1.82--0.5) | -1.17 (-1.83--0.51) | -0.63 (-13.53-14.2) | -2.55 (-15.46-12.34) |
| Ethiopia | 95+ | -0.85 (-2.59-0.91) | -0.86 (-2.59-0.91) | -4.35 (-23.48-19.57) | -5.62 (-24.59-18.11) |

**Table S27. Period rate ratios for prevalence rates of HF impairment under CRD cause categories overall and for COPD, ILD&PS, and PC.**

| **Region** | **Period** |  | **Prevalence Rate Ratio** | |  |
| --- | --- | --- | --- | --- | --- |
|  |  | **CRDs** | **COPD** | **ILD&PS** | **PC** |
| Global | 1992-1996 | 1.01 (0.99-1.04) | 1.02 (0.99-1.04) | 0.64 (0.62-0.65) | 1 (0.97-1.04) |
| Global | 1997-2001 | 1.04 (1.01-1.06) | 1.04 (1.02-1.06) | 0.79 (0.78-0.81) | 1.03 (0.99-1.06) |
| Global | 2002-2006 | 1 (1-1) | 1 (1-1) | 1 (1-1) | 1 (1-1) |
| Global | 2007-2011 | 0.99 (0.97-1.01) | 0.99 (0.97-1.01) | 1.19 (1.17-1.21) | 0.97 (0.94-1) |
| Global | 2012-2016 | 0.99 (0.97-1.02) | 0.99 (0.97-1.01) | 1.36 (1.34-1.38) | 0.96 (0.92-0.99) |
| Global | 2017-2021 | 1.02 (0.99-1.04) | 1.01 (0.98-1.03) | 1.48 (1.46-1.51) | 0.93 (0.9-0.97) |
| Low SDI | 1992-1996 | 1.01 (1-1.03) | 1.01 (1-1.03) | 0.95 (0.86-1.03) | 1.03 (0.79-1.33) |
| Low SDI | 1997-2001 | 1.01 (1-1.03) | 1.01 (1-1.03) | 0.97 (0.9-1.05) | 1.03 (0.83-1.27) |
| Low SDI | 2002-2006 | 1 (1-1) | 1 (1-1) | 1 (1-1) | 1 (1-1) |
| Low SDI | 2007-2011 | 0.97 (0.96-0.99) | 0.97 (0.96-0.99) | 1.03 (0.95-1.11) | 0.98 (0.8-1.2) |
| Low SDI | 2012-2016 | 0.95 (0.94-0.96) | 0.95 (0.93-0.96) | 1.07 (0.98-1.16) | 0.94 (0.73-1.21) |
| Low SDI | 2017-2021 | 0.93 (0.91-0.94) | 0.93 (0.91-0.94) | 1.09 (0.99-1.2) | 0.93 (0.67-1.28) |
| Low-middle SDI | 1992-1996 | 0.99 (0.98-1) | 0.99 (0.98-1) | 0.87 (0.83-0.92) | 1.04 (0.88-1.22) |
| Low-middle SDI | 1997-2001 | 0.99 (0.98-1) | 0.99 (0.98-1.01) | 0.92 (0.88-0.96) | 1.03 (0.88-1.19) |
| Low-middle SDI | 2002-2006 | 1 (1-1) | 1 (1-1) | 1 (1-1) | 1 (1-1) |
| Low-middle SDI | 2007-2011 | 0.99 (0.98-1) | 0.99 (0.98-1) | 1.05 (1.01-1.1) | 0.97 (0.84-1.12) |
| Low-middle SDI | 2012-2016 | 0.98 (0.97-0.99) | 0.98 (0.97-0.99) | 1.11 (1.07-1.16) | 0.95 (0.82-1.09) |
| Low-middle SDI | 2017-2021 | 0.97 (0.96-0.98) | 0.96 (0.95-0.97) | 1.15 (1.1-1.21) | 0.93 (0.8-1.09) |
| Middle SDI | 1992-1996 | 1.13 (1.08-1.17) | 1.13 (1.09-1.17) | 0.72 (0.69-0.76) | 0.99 (0.91-1.08) |
| Middle SDI | 1997-2001 | 1.11 (1.07-1.14) | 1.11 (1.07-1.15) | 0.83 (0.79-0.86) | 1.01 (0.93-1.09) |
| Middle SDI | 2002-2006 | 1 (1-1) | 1 (1-1) | 1 (1-1) | 1 (1-1) |
| Middle SDI | 2007-2011 | 0.95 (0.92-0.98) | 0.94 (0.91-0.98) | 1.19 (1.15-1.24) | 1.02 (0.95-1.1) |
| Middle SDI | 2012-2016 | 0.91 (0.88-0.94) | 0.9 (0.87-0.94) | 1.34 (1.29-1.39) | 1.03 (0.95-1.12) |
| Middle SDI | 2017-2021 | 0.9 (0.87-0.94) | 0.9 (0.86-0.93) | 1.4 (1.34-1.45) | 0.96 (0.87-1.05) |
| High-middle SDI | 1992-1996 | 1.07 (1.03-1.11) | 1.07 (1.04-1.11) | 0.56 (0.53-0.59) | 0.94 (0.87-1.01) |
| High-middle SDI | 1997-2001 | 1.11 (1.08-1.15) | 1.12 (1.08-1.15) | 0.71 (0.68-0.74) | 1.04 (0.97-1.11) |
| High-middle SDI | 2002-2006 | 1 (1-1) | 1 (1-1) | 1 (1-1) | 1 (1-1) |
| High-middle SDI | 2007-2011 | 0.94 (0.91-0.97) | 0.94 (0.91-0.97) | 1.3 (1.25-1.35) | 0.97 (0.91-1.04) |
| High-middle SDI | 2012-2016 | 0.93 (0.9-0.96) | 0.93 (0.9-0.96) | 1.48 (1.42-1.54) | 0.96 (0.89-1.03) |
| High-middle SDI | 2017-2021 | 0.98 (0.94-1.01) | 0.97 (0.94-1) | 1.61 (1.54-1.68) | 0.91 (0.84-0.99) |
| High SDI | 1992-1996 | 0.83 (0.82-0.85) | 0.84 (0.83-0.86) | 0.54 (0.52-0.56) | 1.05 (0.99-1.11) |
| High SDI | 1997-2001 | 0.91 (0.9-0.93) | 0.92 (0.9-0.94) | 0.76 (0.74-0.78) | 1.03 (0.98-1.09) |
| High SDI | 2002-2006 | 1 (1-1) | 1 (1-1) | 1 (1-1) | 1 (1-1) |
| High SDI | 2007-2011 | 1.09 (1.07-1.11) | 1.08 (1.07-1.1) | 1.22 (1.19-1.24) | 0.94 (0.89-0.99) |
| High SDI | 2012-2016 | 1.16 (1.14-1.18) | 1.15 (1.13-1.17) | 1.43 (1.4-1.46) | 0.91 (0.87-0.96) |
| High SDI | 2017-2021 | 1.2 (1.18-1.22) | 1.18 (1.15-1.2) | 1.61 (1.58-1.65) | 0.93 (0.88-0.98) |
| Andean Latin America | 1992-1996 | 0.84 (0.81-0.87) | 0.9 (0.86-0.94) | 0.66 (0.6-0.72) | 0.76 (0.47-1.23) |
| Andean Latin America | 1997-2001 | 0.91 (0.87-0.94) | 0.94 (0.91-0.98) | 0.79 (0.73-0.86) | 0.81 (0.53-1.24) |
| Andean Latin America | 2002-2006 | 1 (1-1) | 1 (1-1) | 1 (1-1) | 1 (1-1) |
| Andean Latin America | 2007-2011 | 1.08 (1.05-1.11) | 1.03 (0.99-1.07) | 1.25 (1.17-1.33) | 1.14 (0.8-1.63) |
| Andean Latin America | 2012-2016 | 1.1 (1.07-1.14) | 1 (0.96-1.03) | 1.48 (1.39-1.57) | 1.11 (0.76-1.63) |
| Andean Latin America | 2017-2021 | 1.09 (1.06-1.13) | 0.97 (0.94-1.01) | 1.56 (1.46-1.67) | 1.04 (0.67-1.62) |
| Australasia | 1992-1996 | 0.84 (0.81-0.86) | 0.86 (0.84-0.88) | 0.35 (0.3-0.41) | 0.45 (0.29-0.71) |
| Australasia | 1997-2001 | 0.94 (0.91-0.96) | 0.95 (0.92-0.97) | 0.75 (0.66-0.84) | 0.64 (0.45-0.91) |
| Australasia | 2002-2006 | 1 (1-1) | 1 (1-1) | 1 (1-1) | 1 (1-1) |
| Australasia | 2007-2011 | 1.11 (1.09-1.14) | 1.1 (1.07-1.12) | 1.41 (1.28-1.56) | 1.25 (0.94-1.66) |
| Australasia | 2012-2016 | 1.17 (1.14-1.19) | 1.15 (1.12-1.17) | 1.54 (1.39-1.71) | 1.3 (0.94-1.8) |
| Australasia | 2017-2021 | 1.23 (1.2-1.26) | 1.21 (1.18-1.24) | 1.63 (1.46-1.83) | 1.37 (0.93-2.02) |
| Caribbean | 1992-1996 | 0.78 (0.75-0.81) | 0.78 (0.75-0.81) | 0.61 (0.44-0.86) | 1.24 (0.34-4.49) |
| Caribbean | 1997-2001 | 0.9 (0.87-0.93) | 0.9 (0.87-0.94) | 0.73 (0.54-0.98) | 1.31 (0.4-4.3) |
| Caribbean | 2002-2006 | 1 (1-1) | 1 (1-1) | 1 (1-1) | 1 (1-1) |
| Caribbean | 2007-2011 | 1.04 (1.01-1.08) | 1.04 (1-1.07) | 1.27 (0.99-1.62) | 0.81 (0.23-2.9) |
| Caribbean | 2012-2016 | 1.05 (1.02-1.09) | 1.05 (1.01-1.08) | 1.43 (1.12-1.83) | 0.66 (0.17-2.56) |
| Caribbean | 2017-2021 | 1.03 (1-1.07) | 1.03 (0.99-1.07) | 1.42 (1.1-1.85) | 0.56 (0.14-2.33) |
| Central Asia | 1992-1996 | 1.44 (1.38-1.5) | 1.44 (1.38-1.5) | 1.38 (0.98-1.95) | 1.8 (0.73-4.4) |
| Central Asia | 1997-2001 | 1.25 (1.2-1.31) | 1.25 (1.2-1.31) | 1.29 (0.92-1.81) | 1.43 (0.58-3.48) |
| Central Asia | 2002-2006 | 1 (1-1) | 1 (1-1) | 1 (1-1) | 1 (1-1) |
| Central Asia | 2007-2011 | 0.92 (0.88-0.96) | 0.92 (0.88-0.96) | 1.01 (0.71-1.43) | 1.1 (0.43-2.76) |
| Central Asia | 2012-2016 | 0.94 (0.9-0.98) | 0.94 (0.9-0.98) | 1.13 (0.8-1.59) | 1.47 (0.59-3.68) |
| Central Asia | 2017-2021 | 1 (0.96-1.05) | 1 (0.96-1.05) | 1 (0.7-1.43) | 1.43 (0.52-3.92) |
| Central Europe | 1992-1996 | 0.96 (0.94-0.98) | 0.96 (0.94-0.98) | 0.87 (0.72-1.04) | 1.22 (0.93-1.6) |
| Central Europe | 1997-2001 | 0.95 (0.93-0.96) | 0.94 (0.93-0.96) | 0.9 (0.76-1.06) | 1.18 (0.92-1.51) |
| Central Europe | 2002-2006 | 1 (1-1) | 1 (1-1) | 1 (1-1) | 1 (1-1) |
| Central Europe | 2007-2011 | 0.99 (0.98-1.01) | 0.99 (0.97-1.01) | 1.24 (1.06-1.44) | 0.66 (0.5-0.87) |
| Central Europe | 2012-2016 | 1.01 (0.99-1.03) | 1.01 (0.99-1.03) | 1.53 (1.31-1.79) | 0.49 (0.36-0.68) |
| Central Europe | 2017-2021 | 1 (0.98-1.02) | 0.99 (0.97-1.01) | 1.89 (1.58-2.25) | 0.5 (0.35-0.71) |
| Central Latin America | 1992-1996 | 0.95 (0.93-0.96) | 0.96 (0.94-0.97) | 0.66 (0.59-0.74) | 1.54 (1.15-2.05) |
| Central Latin America | 1997-2001 | 0.98 (0.97-1) | 0.99 (0.97-1) | 0.81 (0.74-0.9) | 1.25 (0.94-1.65) |
| Central Latin America | 2002-2006 | 1 (1-1) | 1 (1-1) | 1 (1-1) | 1 (1-1) |
| Central Latin America | 2007-2011 | 0.99 (0.98-1.01) | 0.99 (0.97-1) | 1.2 (1.11-1.3) | 0.8 (0.61-1.07) |
| Central Latin America | 2012-2016 | 0.98 (0.97-1) | 0.97 (0.96-0.99) | 1.31 (1.21-1.43) | 0.67 (0.5-0.9) |
| Central Latin America | 2017-2021 | 0.98 (0.97-1) | 0.97 (0.96-0.99) | 1.42 (1.3-1.55) | 0.65 (0.48-0.87) |
| Central Sub-Saharan Africa | 1992-1996 | 1.03 (0.99-1.06) | 1.03 (0.99-1.06) | 1.05 (0.73-1.5) | 0.99 (0.55-1.78) |
| Central Sub-Saharan Africa | 1997-2001 | 1.01 (0.98-1.05) | 1.01 (0.98-1.05) | 1.04 (0.76-1.42) | 0.99 (0.58-1.67) |
| Central Sub-Saharan Africa | 2002-2006 | 1 (1-1) | 1 (1-1) | 1 (1-1) | 1 (1-1) |
| Central Sub-Saharan Africa | 2007-2011 | 0.99 (0.96-1.02) | 0.99 (0.96-1.02) | 1.01 (0.75-1.36) | 0.97 (0.59-1.6) |
| Central Sub-Saharan Africa | 2012-2016 | 0.92 (0.89-0.95) | 0.92 (0.89-0.95) | 0.99 (0.7-1.41) | 0.9 (0.51-1.6) |
| Central Sub-Saharan Africa | 2017-2021 | 0.86 (0.82-0.89) | 0.85 (0.82-0.89) | 0.98 (0.64-1.49) | 0.87 (0.44-1.71) |
| East Asia | 1992-1996 | 1.19 (1.14-1.24) | 1.19 (1.14-1.24) | 0.75 (0.68-0.83) | 0.91 (0.83-1.01) |
| East Asia | 1997-2001 | 1.18 (1.13-1.22) | 1.18 (1.14-1.22) | 0.89 (0.82-0.97) | 1 (0.93-1.08) |
| East Asia | 2002-2006 | 1 (1-1) | 1 (1-1) | 1 (1-1) | 1 (1-1) |
| East Asia | 2007-2011 | 0.88 (0.85-0.92) | 0.88 (0.85-0.92) | 1.39 (1.29-1.49) | 1.02 (0.95-1.09) |
| East Asia | 2012-2016 | 0.81 (0.78-0.84) | 0.81 (0.78-0.84) | 1.69 (1.55-1.84) | 1.05 (0.96-1.15) |
| East Asia | 2017-2021 | 0.78 (0.74-0.81) | 0.78 (0.74-0.81) | 1.73 (1.55-1.93) | 0.98 (0.87-1.11) |
| Eastern Europe | 1992-1996 | 1.61 (1.58-1.64) | 1.61 (1.58-1.64) | 2.51 (2.06-3.07) | 0.83 (0.58-1.18) |
| Eastern Europe | 1997-2001 | 1.34 (1.32-1.36) | 1.34 (1.31-1.36) | 1.85 (1.51-2.27) | 0.96 (0.71-1.29) |
| Eastern Europe | 2002-2006 | 1 (1-1) | 1 (1-1) | 1 (1-1) | 1 (1-1) |
| Eastern Europe | 2007-2011 | 0.86 (0.85-0.88) | 0.86 (0.85-0.88) | 0.76 (0.59-0.97) | 0.94 (0.69-1.27) |
| Eastern Europe | 2012-2016 | 0.82 (0.8-0.84) | 0.82 (0.8-0.84) | 0.85 (0.67-1.08) | 0.62 (0.43-0.91) |
| Eastern Europe | 2017-2021 | 0.9 (0.88-0.92) | 0.9 (0.88-0.92) | 1.25 (1-1.57) | 0.6 (0.39-0.93) |
| Eastern Sub-Saharan Africa | 1992-1996 | 1.07 (1.05-1.09) | 1.07 (1.06-1.09) | 0.94 (0.75-1.19) | 1.14 (0.83-1.57) |
| Eastern Sub-Saharan Africa | 1997-2001 | 1.05 (1.03-1.06) | 1.05 (1.03-1.06) | 0.98 (0.82-1.17) | 1.1 (0.83-1.44) |
| Eastern Sub-Saharan Africa | 2002-2006 | 1 (1-1) | 1 (1-1) | 1 (1-1) | 1 (1-1) |
| Eastern Sub-Saharan Africa | 2007-2011 | 0.93 (0.92-0.95) | 0.93 (0.92-0.95) | 1 (0.84-1.19) | 0.88 (0.67-1.16) |
| Eastern Sub-Saharan Africa | 2012-2016 | 0.87 (0.86-0.89) | 0.87 (0.86-0.89) | 1 (0.8-1.26) | 0.81 (0.59-1.13) |
| Eastern Sub-Saharan Africa | 2017-2021 | 0.83 (0.82-0.85) | 0.83 (0.82-0.85) | 1.02 (0.75-1.38) | 0.77 (0.51-1.16) |
| High-income Asia Pacific | 1992-1996 | 0.86 (0.84-0.88) | 0.9 (0.88-0.92) | 0.62 (0.59-0.66) | 1.04 (0.92-1.18) |
| High-income Asia Pacific | 1997-2001 | 0.95 (0.93-0.97) | 0.96 (0.94-0.98) | 0.88 (0.83-0.92) | 1.07 (0.96-1.2) |
| High-income Asia Pacific | 2002-2006 | 1 (1-1) | 1 (1-1) | 1 (1-1) | 1 (1-1) |
| High-income Asia Pacific | 2007-2011 | 1.01 (0.99-1.03) | 0.99 (0.97-1.01) | 1.12 (1.08-1.17) | 0.91 (0.82-1.02) |
| High-income Asia Pacific | 2012-2016 | 1.06 (1.05-1.08) | 1.01 (1-1.03) | 1.37 (1.31-1.42) | 0.9 (0.81-1.01) |
| High-income Asia Pacific | 2017-2021 | 1.09 (1.07-1.11) | 1.03 (1.01-1.04) | 1.48 (1.42-1.54) | 0.91 (0.81-1.03) |
| High-income North America | 1992-1996 | 0.78 (0.75-0.81) | 0.79 (0.75-0.82) | 0.57 (0.54-0.6) | 1.25 (1.1-1.43) |
| High-income North America | 1997-2001 | 0.88 (0.85-0.92) | 0.89 (0.85-0.92) | 0.76 (0.73-0.8) | 1.06 (0.94-1.2) |
| High-income North America | 2002-2006 | 1 (1-1) | 1 (1-1) | 1 (1-1) | 1 (1-1) |
| High-income North America | 2007-2011 | 1.16 (1.12-1.2) | 1.16 (1.12-1.2) | 1.21 (1.17-1.26) | 0.95 (0.84-1.07) |
| High-income North America | 2012-2016 | 1.24 (1.2-1.29) | 1.24 (1.19-1.29) | 1.35 (1.29-1.4) | 0.85 (0.75-0.97) |
| High-income North America | 2017-2021 | 1.32 (1.27-1.38) | 1.31 (1.26-1.36) | 1.57 (1.5-1.64) | 0.83 (0.72-0.95) |
| North Africa and Middle East | 1992-1996 | 0.93 (0.92-0.95) | 0.93 (0.92-0.95) | 0.74 (0.61-0.91) | 0.94 (0.59-1.51) |
| North Africa and Middle East | 1997-2001 | 0.94 (0.92-0.96) | 0.94 (0.92-0.96) | 0.82 (0.68-0.99) | 0.89 (0.57-1.39) |
| North Africa and Middle East | 2002-2006 | 1 (1-1) | 1 (1-1) | 1 (1-1) | 1 (1-1) |
| North Africa and Middle East | 2007-2011 | 1.06 (1.04-1.07) | 1.05 (1.04-1.07) | 1.17 (0.99-1.37) | 1.11 (0.74-1.64) |
| North Africa and Middle East | 2012-2016 | 1.1 (1.08-1.11) | 1.09 (1.08-1.11) | 1.35 (1.15-1.58) | 1.18 (0.78-1.79) |
| North Africa and Middle East | 2017-2021 | 1.08 (1.06-1.1) | 1.08 (1.06-1.1) | 1.38 (1.17-1.63) | 1.21 (0.76-1.94) |
| Oceania | 1992-1996 | 1.05 (0.98-1.14) | 1.05 (0.98-1.14) | 0.93 (0.32-2.71) | 1.34 (0.06-28.62) |
| Oceania | 1997-2001 | 1.03 (0.96-1.11) | 1.03 (0.96-1.11) | 0.9 (0.33-2.42) | 1.18 (0.06-23.18) |
| Oceania | 2002-2006 | 1 (1-1) | 1 (1-1) | 1 (1-1) | 1 (1-1) |
| Oceania | 2007-2011 | 0.99 (0.92-1.06) | 0.99 (0.92-1.06) | 0.93 (0.38-2.27) | 0.82 (0.04-15.88) |
| Oceania | 2012-2016 | 0.99 (0.92-1.06) | 0.99 (0.92-1.06) | 0.89 (0.35-2.29) | 0.66 (0.03-13.17) |
| Oceania | 2017-2021 | 0.98 (0.9-1.06) | 0.98 (0.9-1.06) | 0.84 (0.29-2.42) | 0.53 (0.03-11.17) |
| South Asia | 1992-1996 | 0.99 (0.98-1) | 0.99 (0.98-1) | 0.88 (0.84-0.92) | 1.05 (0.88-1.25) |
| South Asia | 1997-2001 | 1 (0.99-1.01) | 1 (0.99-1.01) | 0.93 (0.89-0.97) | 1.03 (0.88-1.22) |
| South Asia | 2002-2006 | 1 (1-1) | 1 (1-1) | 1 (1-1) | 1 (1-1) |
| South Asia | 2007-2011 | 0.98 (0.97-0.99) | 0.98 (0.97-0.99) | 1.03 (1-1.07) | 0.96 (0.82-1.11) |
| South Asia | 2012-2016 | 0.97 (0.96-0.98) | 0.97 (0.96-0.98) | 1.09 (1.04-1.13) | 0.91 (0.78-1.07) |
| South Asia | 2017-2021 | 0.95 (0.94-0.96) | 0.95 (0.94-0.96) | 1.11 (1.07-1.16) | 0.88 (0.74-1.05) |
| Southeast Asia | 1992-1996 | 1.05 (1.04-1.06) | 1.05 (1.04-1.06) | 0.86 (0.69-1.07) | 1 (0.44-2.32) |
| Southeast Asia | 1997-2001 | 1.02 (1.01-1.03) | 1.02 (1.01-1.03) | 0.9 (0.73-1.1) | 1.14 (0.53-2.43) |
| Southeast Asia | 2002-2006 | 1 (1-1) | 1 (1-1) | 1 (1-1) | 1 (1-1) |
| Southeast Asia | 2007-2011 | 0.97 (0.96-0.98) | 0.97 (0.96-0.98) | 1.11 (0.93-1.33) | 0.85 (0.4-1.8) |
| Southeast Asia | 2012-2016 | 0.95 (0.94-0.96) | 0.95 (0.94-0.96) | 1.25 (1.04-1.49) | 0.79 (0.37-1.68) |
| Southeast Asia | 2017-2021 | 0.95 (0.94-0.96) | 0.95 (0.94-0.95) | 1.4 (1.17-1.69) | 0.92 (0.44-1.96) |
| Southern Latin America | 1992-1996 | 0.79 (0.77-0.82) | 0.8 (0.78-0.83) | 0.63 (0.55-0.73) | 1.12 (0.73-1.71) |
| Southern Latin America | 1997-2001 | 0.89 (0.86-0.92) | 0.89 (0.87-0.92) | 0.8 (0.71-0.91) | 1.03 (0.68-1.55) |
| Southern Latin America | 2002-2006 | 1 (1-1) | 1 (1-1) | 1 (1-1) | 1 (1-1) |
| Southern Latin America | 2007-2011 | 1.06 (1.03-1.09) | 1.05 (1.02-1.08) | 1.23 (1.11-1.37) | 0.92 (0.62-1.37) |
| Southern Latin America | 2012-2016 | 1.09 (1.07-1.12) | 1.07 (1.04-1.1) | 1.45 (1.31-1.61) | 0.96 (0.65-1.41) |
| Southern Latin America | 2017-2021 | 1.1 (1.07-1.13) | 1.06 (1.03-1.09) | 1.67 (1.5-1.86) | 1.07 (0.72-1.57) |
| Southern Sub-Saharan Africa | 1992-1996 | 0.95 (0.91-0.99) | 0.95 (0.91-0.99) | 0.96 (0.84-1.11) | 0.97 (0.67-1.39) |
| Southern Sub-Saharan Africa | 1997-2001 | 0.93 (0.89-0.96) | 0.93 (0.89-0.96) | 0.89 (0.78-1.02) | 0.97 (0.71-1.34) |
| Southern Sub-Saharan Africa | 2002-2006 | 1 (1-1) | 1 (1-1) | 1 (1-1) | 1 (1-1) |
| Southern Sub-Saharan Africa | 2007-2011 | 1 (0.97-1.04) | 1 (0.97-1.04) | 1.01 (0.89-1.14) | 0.96 (0.71-1.31) |
| Southern Sub-Saharan Africa | 2012-2016 | 0.86 (0.83-0.9) | 0.86 (0.83-0.9) | 0.87 (0.76-0.99) | 0.82 (0.57-1.17) |
| Southern Sub-Saharan Africa | 2017-2021 | 0.8 (0.77-0.84) | 0.8 (0.77-0.84) | 0.84 (0.73-0.98) | 0.78 (0.51-1.2) |
| Tropical Latin America | 1992-1996 | 0.94 (0.93-0.96) | 0.95 (0.93-0.97) | 0.54 (0.46-0.64) | 0.66 (0.45-0.95) |
| Tropical Latin America | 1997-2001 | 0.99 (0.98-1.01) | 1 (0.98-1.01) | 0.72 (0.63-0.82) | 0.88 (0.64-1.21) |
| Tropical Latin America | 2002-2006 | 1 (1-1) | 1 (1-1) | 1 (1-1) | 1 (1-1) |
| Tropical Latin America | 2007-2011 | 1 (0.98-1.01) | 0.99 (0.98-1.01) | 1.29 (1.16-1.43) | 1.04 (0.79-1.37) |
| Tropical Latin America | 2012-2016 | 1.02 (1-1.03) | 1.01 (1-1.02) | 1.49 (1.34-1.66) | 1.05 (0.8-1.38) |
| Tropical Latin America | 2017-2021 | 1.03 (1.01-1.04) | 1.02 (1-1.03) | 1.65 (1.47-1.84) | 1.04 (0.79-1.37) |
| Western Europe | 1992-1996 | 0.75 (0.73-0.77) | 0.76 (0.74-0.78) | 0.41 (0.39-0.43) | 1.14 (1.06-1.22) |
| Western Europe | 1997-2001 | 0.86 (0.84-0.88) | 0.87 (0.85-0.89) | 0.62 (0.6-0.64) | 1.1 (1.03-1.17) |
| Western Europe | 2002-2006 | 1 (1-1) | 1 (1-1) | 1 (1-1) | 1 (1-1) |
| Western Europe | 2007-2011 | 1.1 (1.07-1.12) | 1.09 (1.07-1.11) | 1.29 (1.26-1.33) | 0.9 (0.84-0.96) |
| Western Europe | 2012-2016 | 1.17 (1.14-1.19) | 1.15 (1.13-1.18) | 1.52 (1.47-1.56) | 0.82 (0.76-0.88) |
| Western Europe | 2017-2021 | 1.18 (1.15-1.2) | 1.16 (1.14-1.19) | 1.68 (1.63-1.74) | 0.81 (0.75-0.88) |
| Western Sub-Saharan Africa | 1992-1996 | 1.12 (1.1-1.14) | 1.12 (1.1-1.14) | 1.13 (0.98-1.29) | 1.04 (0.68-1.6) |
| Western Sub-Saharan Africa | 1997-2001 | 1.06 (1.05-1.08) | 1.06 (1.05-1.08) | 1.07 (0.95-1.21) | 1.04 (0.73-1.48) |
| Western Sub-Saharan Africa | 2002-2006 | 1 (1-1) | 1 (1-1) | 1 (1-1) | 1 (1-1) |
| Western Sub-Saharan Africa | 2007-2011 | 0.94 (0.93-0.96) | 0.94 (0.93-0.96) | 0.97 (0.86-1.1) | 1.07 (0.76-1.51) |
| Western Sub-Saharan Africa | 2012-2016 | 0.93 (0.91-0.94) | 0.92 (0.91-0.94) | 1.01 (0.87-1.16) | 1.01 (0.66-1.56) |
| Western Sub-Saharan Africa | 2017-2021 | 0.87 (0.86-0.89) | 0.87 (0.86-0.89) | 1.01 (0.85-1.2) | 0.96 (0.55-1.68) |
| **Five Countries** |  |  |  |  |  |
| United States | 1992-1996 | 0.8 (0.76-0.84) | 0.8 (0.76-0.84) | 0.6 (0.57-0.64) | 1.31 (1.14-1.51) |
| United States | 1997-2001 | 0.89 (0.86-0.93) | 0.9 (0.86-0.94) | 0.79 (0.75-0.84) | 1.1 (0.96-1.26) |
| United States | 2002-2006 | 1 (1-1) | 1 (1-1) | 1 (1-1) | 1 (1-1) |
| United States | 2007-2011 | 1.16 (1.11-1.21) | 1.16 (1.11-1.2) | 1.2 (1.14-1.25) | 0.92 (0.81-1.05) |
| United States | 2012-2016 | 1.25 (1.19-1.3) | 1.24 (1.19-1.29) | 1.31 (1.25-1.37) | 0.8 (0.7-0.92) |
| United States | 2017-2021 | 1.32 (1.26-1.38) | 1.31 (1.25-1.37) | 1.52 (1.45-1.6) | 0.77 (0.66-0.89) |
| China | 1992-1996 | 1.19 (1.14-1.24) | 1.19 (1.15-1.25) | 0.78 (0.7-0.87) | 0.94 (0.85-1.04) |
| China | 1997-2001 | 1.18 (1.14-1.23) | 1.18 (1.14-1.23) | 0.91 (0.83-1) | 1.01 (0.94-1.1) |
| China | 2002-2006 | 1 (1-1) | 1 (1-1) | 1 (1-1) | 1 (1-1) |
| China | 2007-2011 | 0.88 (0.85-0.92) | 0.88 (0.85-0.91) | 1.36 (1.26-1.47) | 1.02 (0.94-1.1) |
| China | 2012-2016 | 0.8 (0.77-0.84) | 0.8 (0.77-0.83) | 1.67 (1.52-1.83) | 1.05 (0.95-1.16) |
| China | 2017-2021 | 0.77 (0.74-0.81) | 0.77 (0.74-0.81) | 1.69 (1.51-1.9) | 0.97 (0.85-1.1) |
| Brazil | 1992-1996 | 0.94 (0.93-0.96) | 0.95 (0.94-0.97) | 0.54 (0.45-0.63) | 0.65 (0.44-0.96) |
| Brazil | 1997-2001 | 0.99 (0.98-1.01) | 1 (0.98-1.01) | 0.72 (0.62-0.82) | 0.89 (0.64-1.23) |
| Brazil | 2002-2006 | 1 (1-1) | 1 (1-1) | 1 (1-1) | 1 (1-1) |
| Brazil | 2007-2011 | 1 (0.98-1.01) | 0.99 (0.98-1.01) | 1.29 (1.16-1.43) | 1.05 (0.79-1.39) |
| Brazil | 2012-2016 | 1.02 (1-1.03) | 1.01 (0.99-1.02) | 1.49 (1.34-1.66) | 1.06 (0.81-1.4) |
| Brazil | 2017-2021 | 1.02 (1.01-1.04) | 1.01 (1-1.03) | 1.64 (1.46-1.85) | 1.05 (0.8-1.4) |
| India | 1992-1996 | 0.99 (0.98-1) | 0.99 (0.99-1) | 0.89 (0.84-0.93) | 1.05 (0.87-1.28) |
| India | 1997-2001 | 1 (0.99-1.01) | 1 (1-1.01) | 0.93 (0.89-0.98) | 1.04 (0.87-1.24) |
| India | 2002-2006 | 1 (1-1) | 1 (1-1) | 1 (1-1) | 1 (1-1) |
| India | 2007-2011 | 0.98 (0.97-0.99) | 0.98 (0.97-0.99) | 1.03 (0.99-1.07) | 0.95 (0.81-1.13) |
| India | 2012-2016 | 0.97 (0.96-0.98) | 0.97 (0.96-0.97) | 1.08 (1.04-1.13) | 0.91 (0.76-1.08) |
| India | 2017-2021 | 0.95 (0.95-0.96) | 0.95 (0.95-0.96) | 1.11 (1.06-1.16) | 0.88 (0.73-1.06) |
| Ethiopia | 1992-1996 | 1.1 (1.07-1.14) | 1.1 (1.07-1.14) | 0.93 (0.59-1.46) | 1.13 (0.64-2.02) |
| Ethiopia | 1997-2001 | 1.08 (1.04-1.11) | 1.08 (1.04-1.11) | 1 (0.7-1.44) | 1.1 (0.66-1.85) |
| Ethiopia | 2002-2006 | 1 (1-1) | 1 (1-1) | 1 (1-1) | 1 (1-1) |
| Ethiopia | 2007-2011 | 0.92 (0.89-0.94) | 0.92 (0.89-0.94) | 1.01 (0.72-1.42) | 0.86 (0.52-1.44) |
| Ethiopia | 2012-2016 | 0.84 (0.81-0.87) | 0.84 (0.81-0.87) | 0.96 (0.63-1.48) | 0.74 (0.41-1.32) |
| Ethiopia | 2017-2021 | 0.8 (0.77-0.83) | 0.8 (0.77-0.83) | 0.92 (0.53-1.6) | 0.69 (0.35-1.37) |

**Table S28. Cohort rate ratios for prevalence rates of HF impairment under CRD cause categories overall and for COPD, ILD&PS, and PC.**

| **Region** | **Cohort** |  | **Prevalence Rate Ratio** | |  |
| --- | --- | --- | --- | --- | --- |
|  |  | **CRDs** | **COPD** | **ILD&PS** | **PC** |
| Global | 1897-1901 | 0.86 (0.68-1.09) | 0.89 (0.71-1.12) | 0.15 (0.13-0.18) | 1.09 (0.69-1.72) |
| Global | 1902-1906 | 0.92 (0.84-1.01) | 0.95 (0.87-1.05) | 0.18 (0.17-0.19) | 1.09 (0.91-1.31) |
| Global | 1907-1911 | 0.99 (0.94-1.04) | 1.02 (0.96-1.07) | 0.22 (0.21-0.23) | 1.12 (1.01-1.23) |
| Global | 1912-1916 | 1.02 (0.98-1.07) | 1.05 (1.01-1.09) | 0.28 (0.27-0.29) | 1.18 (1.1-1.26) |
| Global | 1917-1921 | 1.1 (1.07-1.14) | 1.12 (1.09-1.16) | 0.35 (0.35-0.36) | 1.21 (1.14-1.27) |
| Global | 1922-1926 | 1.08 (1.05-1.12) | 1.1 (1.07-1.13) | 0.44 (0.43-0.45) | 1.29 (1.24-1.36) |
| Global | 1927-1931 | 1.07 (1.04-1.1) | 1.08 (1.05-1.11) | 0.53 (0.52-0.54) | 1.29 (1.24-1.35) |
| Global | 1932-1936 | 1.08 (1.05-1.11) | 1.09 (1.06-1.11) | 0.64 (0.63-0.65) | 1.25 (1.2-1.31) |
| Global | 1937-1941 | 1.05 (1.02-1.08) | 1.05 (1.03-1.08) | 0.75 (0.74-0.76) | 1.15 (1.1-1.2) |
| Global | 1942-1946 | 1.03 (1-1.06) | 1.03 (1.01-1.06) | 0.89 (0.87-0.9) | 1.05 (1.01-1.1) |
| Global | 1947-1951 | 1 (1-1) | 1 (1-1) | 1 (1-1) | 1 (1-1) |
| Global | 1952-1956 | 0.95 (0.92-0.99) | 0.95 (0.92-0.99) | 1.03 (1-1.05) | 0.99 (0.94-1.04) |
| Global | 1957-1961 | 0.91 (0.87-0.96) | 0.91 (0.86-0.96) | 1.09 (1.06-1.13) | 0.96 (0.89-1.03) |
| Global | 1962-1966 | 0.91 (0.84-1) | 0.91 (0.83-0.99) | 1.17 (1.12-1.23) | 0.97 (0.87-1.09) |
| Low SDI | 1897-1901 | 1.1 (0.94-1.29) | 1.11 (0.95-1.29) | 0.64 (0.15-2.7) | 0.36 (0-191.84) |
| Low SDI | 1902-1906 | 1.13 (1.05-1.21) | 1.13 (1.06-1.21) | 0.76 (0.45-1.29) | 1.28 (0.3-5.44) |
| Low SDI | 1907-1911 | 1.17 (1.13-1.21) | 1.17 (1.13-1.22) | 0.82 (0.64-1.04) | 1.45 (0.76-2.76) |
| Low SDI | 1912-1916 | 1.15 (1.12-1.18) | 1.15 (1.12-1.18) | 0.82 (0.7-0.96) | 1.38 (0.9-2.11) |
| Low SDI | 1917-1921 | 1.12 (1.1-1.15) | 1.13 (1.1-1.15) | 0.84 (0.74-0.94) | 1.34 (0.97-1.86) |
| Low SDI | 1922-1926 | 1.1 (1.08-1.12) | 1.1 (1.08-1.12) | 0.86 (0.78-0.95) | 1.29 (0.99-1.68) |
| Low SDI | 1927-1931 | 1.08 (1.07-1.1) | 1.09 (1.07-1.11) | 0.89 (0.81-0.97) | 1.25 (0.99-1.59) |
| Low SDI | 1932-1936 | 1.07 (1.05-1.09) | 1.07 (1.06-1.09) | 0.92 (0.84-1) | 1.2 (0.96-1.49) |
| Low SDI | 1937-1941 | 1.05 (1.04-1.07) | 1.06 (1.04-1.07) | 0.95 (0.88-1.03) | 1.12 (0.91-1.38) |
| Low SDI | 1942-1946 | 1.03 (1.01-1.05) | 1.03 (1.02-1.05) | 0.97 (0.9-1.06) | 1.06 (0.86-1.31) |
| Low SDI | 1947-1951 | 1 (1-1) | 1 (1-1) | 1 (1-1) | 1 (1-1) |
| Low SDI | 1952-1956 | 0.96 (0.94-0.98) | 0.96 (0.94-0.97) | 1 (0.91-1.1) | 0.96 (0.75-1.22) |
| Low SDI | 1957-1961 | 0.92 (0.89-0.94) | 0.92 (0.89-0.94) | 1.02 (0.9-1.16) | 0.94 (0.7-1.26) |
| Low SDI | 1962-1966 | 0.9 (0.86-0.93) | 0.89 (0.85-0.93) | 1.06 (0.89-1.26) | 0.9 (0.6-1.35) |
| Low-middle SDI | 1897-1901 | 0.99 (0.9-1.08) | 0.99 (0.9-1.09) | 0.49 (0.29-0.83) | 1.01 (0.22-4.66) |
| Low-middle SDI | 1902-1906 | 0.99 (0.95-1.03) | 0.99 (0.95-1.03) | 0.56 (0.45-0.69) | 1.12 (0.59-2.15) |
| Low-middle SDI | 1907-1911 | 1.03 (1.01-1.06) | 1.04 (1.01-1.06) | 0.63 (0.57-0.71) | 1.22 (0.85-1.77) |
| Low-middle SDI | 1912-1916 | 1.02 (1-1.04) | 1.02 (1-1.04) | 0.66 (0.61-0.72) | 1.19 (0.9-1.58) |
| Low-middle SDI | 1917-1921 | 1.01 (0.99-1.03) | 1.01 (1-1.03) | 0.69 (0.65-0.74) | 1.15 (0.91-1.46) |
| Low-middle SDI | 1922-1926 | 1.02 (1-1.03) | 1.02 (1.01-1.04) | 0.74 (0.7-0.79) | 1.12 (0.92-1.37) |
| Low-middle SDI | 1927-1931 | 1.03 (1.01-1.04) | 1.03 (1.02-1.04) | 0.79 (0.75-0.83) | 1.1 (0.92-1.32) |
| Low-middle SDI | 1932-1936 | 1.03 (1.02-1.04) | 1.03 (1.02-1.04) | 0.84 (0.8-0.88) | 1.08 (0.91-1.28) |
| Low-middle SDI | 1937-1941 | 1.03 (1.02-1.04) | 1.03 (1.02-1.04) | 0.89 (0.85-0.94) | 1.05 (0.89-1.25) |
| Low-middle SDI | 1942-1946 | 1.02 (1.01-1.03) | 1.02 (1.01-1.03) | 0.95 (0.91-0.99) | 1.04 (0.88-1.23) |
| Low-middle SDI | 1947-1951 | 1 (1-1) | 1 (1-1) | 1 (1-1) | 1 (1-1) |
| Low-middle SDI | 1952-1956 | 0.95 (0.94-0.97) | 0.95 (0.94-0.97) | 1.01 (0.95-1.07) | 0.93 (0.76-1.15) |
| Low-middle SDI | 1957-1961 | 0.91 (0.89-0.93) | 0.91 (0.89-0.93) | 1.02 (0.94-1.11) | 0.88 (0.67-1.15) |
| Low-middle SDI | 1962-1966 | 0.9 (0.86-0.94) | 0.9 (0.86-0.93) | 1.08 (0.96-1.22) | 0.85 (0.57-1.27) |
| Middle SDI | 1897-1901 | 1.31 (0.86-2) | 1.34 (0.88-2.03) | 0.24 (0.16-0.36) | 1.04 (0.3-3.68) |
| Middle SDI | 1902-1906 | 1.4 (1.18-1.65) | 1.42 (1.2-1.67) | 0.28 (0.23-0.33) | 1.02 (0.6-1.72) |
| Middle SDI | 1907-1911 | 1.46 (1.33-1.59) | 1.48 (1.35-1.62) | 0.31 (0.28-0.34) | 1.03 (0.8-1.33) |
| Middle SDI | 1912-1916 | 1.48 (1.39-1.57) | 1.49 (1.4-1.59) | 0.36 (0.33-0.39) | 1.01 (0.85-1.19) |
| Middle SDI | 1917-1921 | 1.44 (1.36-1.51) | 1.45 (1.38-1.53) | 0.42 (0.4-0.45) | 1.02 (0.9-1.15) |
| Middle SDI | 1922-1926 | 1.37 (1.31-1.43) | 1.38 (1.32-1.44) | 0.5 (0.47-0.52) | 1.01 (0.92-1.12) |
| Middle SDI | 1927-1931 | 1.3 (1.25-1.35) | 1.31 (1.25-1.36) | 0.57 (0.54-0.6) | 1 (0.92-1.09) |
| Middle SDI | 1932-1936 | 1.23 (1.18-1.29) | 1.24 (1.19-1.29) | 0.66 (0.63-0.69) | 0.99 (0.91-1.08) |
| Middle SDI | 1937-1941 | 1.17 (1.12-1.21) | 1.17 (1.12-1.22) | 0.75 (0.72-0.78) | 0.99 (0.91-1.07) |
| Middle SDI | 1942-1946 | 1.09 (1.05-1.14) | 1.09 (1.05-1.14) | 0.89 (0.85-0.93) | 0.99 (0.91-1.07) |
| Middle SDI | 1947-1951 | 1 (1-1) | 1 (1-1) | 1 (1-1) | 1 (1-1) |
| Middle SDI | 1952-1956 | 0.91 (0.86-0.95) | 0.9 (0.86-0.95) | 1.08 (1.03-1.14) | 1.01 (0.93-1.11) |
| Middle SDI | 1957-1961 | 0.82 (0.75-0.89) | 0.81 (0.75-0.88) | 1.24 (1.15-1.34) | 0.98 (0.86-1.11) |
| Middle SDI | 1962-1966 | 0.81 (0.7-0.93) | 0.8 (0.69-0.92) | 1.4 (1.26-1.56) | 1.04 (0.86-1.26) |
| High-middle SDI | 1897-1901 | 1.08 (0.71-1.64) | 1.1 (0.72-1.67) | 0.12 (0.07-0.19) | 1.34 (0.45-3.95) |
| High-middle SDI | 1902-1906 | 1.15 (0.99-1.35) | 1.18 (1.01-1.37) | 0.14 (0.11-0.17) | 1.2 (0.79-1.83) |
| High-middle SDI | 1907-1911 | 1.21 (1.12-1.32) | 1.24 (1.14-1.34) | 0.16 (0.14-0.18) | 1.09 (0.89-1.34) |
| High-middle SDI | 1912-1916 | 1.22 (1.15-1.29) | 1.24 (1.17-1.32) | 0.19 (0.18-0.21) | 1.09 (0.96-1.25) |
| High-middle SDI | 1917-1921 | 1.33 (1.27-1.4) | 1.36 (1.29-1.42) | 0.25 (0.23-0.27) | 1.15 (1.04-1.28) |
| High-middle SDI | 1922-1926 | 1.25 (1.19-1.3) | 1.26 (1.21-1.32) | 0.31 (0.3-0.33) | 1.18 (1.08-1.28) |
| High-middle SDI | 1927-1931 | 1.14 (1.1-1.19) | 1.16 (1.11-1.2) | 0.39 (0.37-0.42) | 1.1 (1.02-1.19) |
| High-middle SDI | 1932-1936 | 1.15 (1.1-1.19) | 1.15 (1.11-1.2) | 0.53 (0.5-0.55) | 1.09 (1.01-1.18) |
| High-middle SDI | 1937-1941 | 1.06 (1.02-1.1) | 1.06 (1.02-1.11) | 0.64 (0.61-0.67) | 1 (0.93-1.08) |
| High-middle SDI | 1942-1946 | 1.04 (1-1.08) | 1.04 (1-1.09) | 0.83 (0.8-0.87) | 0.99 (0.92-1.08) |
| High-middle SDI | 1947-1951 | 1 (1-1) | 1 (1-1) | 1 (1-1) | 1 (1-1) |
| High-middle SDI | 1952-1956 | 0.92 (0.88-0.98) | 0.92 (0.87-0.97) | 1.09 (1.03-1.15) | 1.04 (0.95-1.14) |
| High-middle SDI | 1957-1961 | 0.87 (0.8-0.95) | 0.86 (0.79-0.94) | 1.35 (1.24-1.47) | 1.12 (0.98-1.28) |
| High-middle SDI | 1962-1966 | 0.89 (0.77-1.03) | 0.88 (0.76-1.02) | 1.72 (1.52-1.96) | 1.29 (1.05-1.58) |
| High SDI | 1897-1901 | 0.44 (0.37-0.52) | 0.47 (0.4-0.56) | 0.1 (0.08-0.13) | 1.07 (0.58-1.95) |
| High SDI | 1902-1906 | 0.48 (0.45-0.52) | 0.51 (0.48-0.55) | 0.12 (0.11-0.13) | 1.11 (0.88-1.42) |
| High SDI | 1907-1911 | 0.53 (0.5-0.55) | 0.56 (0.54-0.58) | 0.15 (0.14-0.16) | 1.18 (1.04-1.35) |
| High SDI | 1912-1916 | 0.59 (0.57-0.6) | 0.62 (0.6-0.64) | 0.2 (0.19-0.21) | 1.3 (1.18-1.44) |
| High SDI | 1917-1921 | 0.67 (0.65-0.69) | 0.69 (0.68-0.71) | 0.27 (0.26-0.28) | 1.37 (1.25-1.49) |
| High SDI | 1922-1926 | 0.73 (0.71-0.75) | 0.75 (0.74-0.77) | 0.35 (0.34-0.36) | 1.55 (1.43-1.67) |
| High SDI | 1927-1931 | 0.79 (0.77-0.8) | 0.8 (0.78-0.82) | 0.46 (0.44-0.47) | 1.65 (1.54-1.77) |
| High SDI | 1932-1936 | 0.83 (0.81-0.85) | 0.84 (0.82-0.86) | 0.59 (0.57-0.6) | 1.64 (1.53-1.76) |
| High SDI | 1937-1941 | 0.86 (0.85-0.88) | 0.87 (0.85-0.89) | 0.73 (0.72-0.75) | 1.47 (1.37-1.58) |
| High SDI | 1942-1946 | 0.93 (0.91-0.95) | 0.93 (0.91-0.95) | 0.88 (0.86-0.9) | 1.2 (1.12-1.29) |
| High SDI | 1947-1951 | 1 (1-1) | 1 (1-1) | 1 (1-1) | 1 (1-1) |
| High SDI | 1952-1956 | 1.07 (1.04-1.1) | 1.08 (1.05-1.11) | 1.08 (1.04-1.11) | 0.9 (0.82-0.99) |
| High SDI | 1957-1961 | 1.14 (1.1-1.19) | 1.15 (1.1-1.2) | 1.12 (1.07-1.18) | 0.82 (0.71-0.94) |
| High SDI | 1962-1966 | 1.19 (1.1-1.28) | 1.2 (1.11-1.29) | 1.19 (1.1-1.29) | 0.73 (0.58-0.93) |
| Andean Latin America | 1897-1901 | 0.52 (0.43-0.64) | 0.75 (0.6-0.93) | 0.15 (0.09-0.25) | 0.12 (0-64.79) |
| Andean Latin America | 1902-1906 | 0.57 (0.51-0.63) | 0.8 (0.71-0.89) | 0.18 (0.14-0.23) | 0.68 (0.14-3.4) |
| Andean Latin America | 1907-1911 | 0.62 (0.58-0.67) | 0.85 (0.78-0.92) | 0.22 (0.18-0.26) | 0.59 (0.19-1.81) |
| Andean Latin America | 1912-1916 | 0.68 (0.64-0.72) | 0.9 (0.84-0.96) | 0.28 (0.24-0.32) | 0.67 (0.31-1.46) |
| Andean Latin America | 1917-1921 | 0.73 (0.69-0.77) | 0.93 (0.87-0.99) | 0.35 (0.31-0.39) | 0.71 (0.38-1.33) |
| Andean Latin America | 1922-1926 | 0.78 (0.75-0.82) | 0.96 (0.91-1.02) | 0.44 (0.4-0.48) | 0.79 (0.46-1.36) |
| Andean Latin America | 1927-1931 | 0.84 (0.81-0.88) | 1 (0.94-1.05) | 0.54 (0.49-0.59) | 0.87 (0.54-1.42) |
| Andean Latin America | 1932-1936 | 0.9 (0.86-0.94) | 1.01 (0.96-1.06) | 0.66 (0.61-0.72) | 0.94 (0.6-1.48) |
| Andean Latin America | 1937-1941 | 0.93 (0.89-0.97) | 1.01 (0.96-1.07) | 0.75 (0.7-0.81) | 0.97 (0.63-1.49) |
| Andean Latin America | 1942-1946 | 0.97 (0.93-1.01) | 1.01 (0.96-1.06) | 0.87 (0.81-0.94) | 0.99 (0.64-1.51) |
| Andean Latin America | 1947-1951 | 1 (1-1) | 1 (1-1) | 1 (1-1) | 1 (1-1) |
| Andean Latin America | 1952-1956 | 1.03 (0.98-1.09) | 0.99 (0.92-1.06) | 1.15 (1.04-1.27) | 1.05 (0.62-1.78) |
| Andean Latin America | 1957-1961 | 1.07 (0.97-1.17) | 0.97 (0.86-1.08) | 1.32 (1.13-1.55) | 1.1 (0.52-2.34) |
| Andean Latin America | 1962-1966 | 1.12 (0.96-1.3) | 0.95 (0.78-1.16) | 1.51 (1.19-1.92) | 1.08 (0.33-3.47) |
| Australasia | 1897-1901 | 0.46 (0.36-0.59) | 0.5 (0.39-0.65) | 0.09 (0.02-0.33) | 0.09 (0-48.92) |
| Australasia | 1902-1906 | 0.5 (0.45-0.55) | 0.54 (0.49-0.61) | 0.08 (0.05-0.15) | 0.15 (0.02-1.03) |
| Australasia | 1907-1911 | 0.53 (0.5-0.57) | 0.58 (0.55-0.62) | 0.09 (0.06-0.12) | 0.15 (0.05-0.42) |
| Australasia | 1912-1916 | 0.58 (0.56-0.6) | 0.63 (0.6-0.66) | 0.11 (0.08-0.13) | 0.19 (0.09-0.37) |
| Australasia | 1917-1921 | 0.64 (0.62-0.66) | 0.68 (0.66-0.71) | 0.15 (0.12-0.18) | 0.27 (0.16-0.45) |
| Australasia | 1922-1926 | 0.71 (0.69-0.73) | 0.75 (0.73-0.77) | 0.21 (0.17-0.24) | 0.38 (0.24-0.59) |
| Australasia | 1927-1931 | 0.79 (0.76-0.81) | 0.82 (0.79-0.84) | 0.3 (0.26-0.34) | 0.51 (0.34-0.77) |
| Australasia | 1932-1936 | 0.85 (0.83-0.88) | 0.88 (0.85-0.9) | 0.42 (0.37-0.48) | 0.64 (0.44-0.94) |
| Australasia | 1937-1941 | 0.91 (0.88-0.93) | 0.92 (0.9-0.94) | 0.58 (0.52-0.65) | 0.76 (0.53-1.08) |
| Australasia | 1942-1946 | 0.95 (0.93-0.97) | 0.96 (0.93-0.98) | 0.77 (0.69-0.86) | 0.87 (0.62-1.23) |
| Australasia | 1947-1951 | 1 (1-1) | 1 (1-1) | 1 (1-1) | 1 (1-1) |
| Australasia | 1952-1956 | 1.07 (1.03-1.1) | 1.06 (1.03-1.1) | 1.24 (1.07-1.45) | 1.16 (0.71-1.89) |
| Australasia | 1957-1961 | 1.16 (1.1-1.23) | 1.15 (1.08-1.22) | 1.6 (1.22-2.09) | 1.44 (0.59-3.52) |
| Australasia | 1962-1966 | 1.28 (1.15-1.42) | 1.26 (1.13-1.4) | 2 (1.25-3.21) | 1.65 (0.31-8.89) |
| Caribbean | 1897-1901 | 0.55 (0.4-0.73) | 0.55 (0.41-0.75) | 0.17 (0.01-2.2) | 15.1 (0.01-21181.83) |
| Caribbean | 1902-1906 | 0.58 (0.52-0.66) | 0.6 (0.52-0.68) | 0.19 (0.06-0.55) | 5.65 (0.11-294.42) |
| Caribbean | 1907-1911 | 0.63 (0.58-0.68) | 0.64 (0.59-0.69) | 0.22 (0.12-0.41) | 5.44 (0.34-87.36) |
| Caribbean | 1912-1916 | 0.67 (0.63-0.72) | 0.68 (0.64-0.73) | 0.26 (0.16-0.43) | 3.31 (0.26-41.79) |
| Caribbean | 1917-1921 | 0.71 (0.67-0.75) | 0.72 (0.68-0.76) | 0.31 (0.2-0.47) | 2.46 (0.22-27.53) |
| Caribbean | 1922-1926 | 0.75 (0.71-0.79) | 0.76 (0.72-0.8) | 0.37 (0.25-0.53) | 2.25 (0.21-23.6) |
| Caribbean | 1927-1931 | 0.79 (0.75-0.82) | 0.79 (0.76-0.83) | 0.45 (0.32-0.63) | 2.06 (0.24-17.98) |
| Caribbean | 1932-1936 | 0.83 (0.79-0.87) | 0.84 (0.8-0.88) | 0.55 (0.4-0.76) | 1.7 (0.19-15.25) |
| Caribbean | 1937-1941 | 0.88 (0.84-0.92) | 0.88 (0.85-0.92) | 0.69 (0.51-0.94) | 1.51 (0.17-13.47) |
| Caribbean | 1942-1946 | 0.94 (0.9-0.98) | 0.94 (0.9-0.98) | 0.82 (0.62-1.1) | 1.2 (0.11-12.73) |
| Caribbean | 1947-1951 | 1 (1-1) | 1 (1-1) | 1 (1-1) | 1 (1-1) |
| Caribbean | 1952-1956 | 1.03 (0.98-1.09) | 1.03 (0.97-1.09) | 1.15 (0.78-1.7) | 1.04 (0.07-15.26) |
| Caribbean | 1957-1961 | 1.06 (0.97-1.16) | 1.06 (0.96-1.16) | 1.34 (0.74-2.43) | 0.91 (0.01-149.81) |
| Caribbean | 1962-1966 | 1.13 (0.97-1.31) | 1.13 (0.97-1.31) | 1.42 (0.57-3.55) | 0.74 (0-941.8) |
| Central Asia | 1897-1901 | 2.41 (1.69-3.42) | 2.41 (1.69-3.43) | 2.03 (0.22-18.6) | 1.34 (0-1435.6) |
| Central Asia | 1902-1906 | 1.99 (1.71-2.31) | 1.99 (1.7-2.32) | 1.82 (0.64-5.18) | 1.04 (0.01-150.86) |
| Central Asia | 1907-1911 | 1.74 (1.57-1.92) | 1.74 (1.57-1.92) | 1.6 (0.79-3.25) | 1.26 (0.13-12.14) |
| Central Asia | 1912-1916 | 1.58 (1.46-1.71) | 1.59 (1.47-1.72) | 1.41 (0.77-2.56) | 1.37 (0.29-6.44) |
| Central Asia | 1917-1921 | 1.44 (1.34-1.54) | 1.44 (1.34-1.55) | 1.23 (0.71-2.14) | 1 (0.25-4.09) |
| Central Asia | 1922-1926 | 1.35 (1.28-1.44) | 1.36 (1.28-1.44) | 1.15 (0.7-1.88) | 1.06 (0.34-3.25) |
| Central Asia | 1927-1931 | 1.32 (1.25-1.39) | 1.33 (1.26-1.4) | 1.14 (0.73-1.76) | 1.06 (0.4-2.83) |
| Central Asia | 1932-1936 | 1.22 (1.15-1.29) | 1.22 (1.16-1.29) | 1.12 (0.72-1.73) | 1.01 (0.38-2.7) |
| Central Asia | 1937-1941 | 1.14 (1.08-1.2) | 1.14 (1.08-1.2) | 1.06 (0.69-1.63) | 1.06 (0.4-2.78) |
| Central Asia | 1942-1946 | 1.04 (0.99-1.1) | 1.04 (0.99-1.11) | 0.98 (0.62-1.54) | 1.02 (0.38-2.75) |
| Central Asia | 1947-1951 | 1 (1-1) | 1 (1-1) | 1 (1-1) | 1 (1-1) |
| Central Asia | 1952-1956 | 0.89 (0.84-0.96) | 0.89 (0.83-0.95) | 0.91 (0.54-1.56) | 0.92 (0.29-2.96) |
| Central Asia | 1957-1961 | 0.74 (0.68-0.82) | 0.74 (0.67-0.82) | 0.88 (0.44-1.78) | 0.8 (0.17-3.76) |
| Central Asia | 1962-1966 | 0.65 (0.56-0.76) | 0.65 (0.55-0.75) | 0.8 (0.29-2.19) | 0.86 (0.07-10.96) |
| Central Europe | 1897-1901 | 1.14 (0.88-1.46) | 1.16 (0.9-1.49) | 0.33 (0.04-2.61) | 3.47 (0.04-331.19) |
| Central Europe | 1902-1906 | 1.02 (0.92-1.13) | 1.03 (0.94-1.14) | 0.24 (0.09-0.64) | 8.2 (2.12-31.73) |
| Central Europe | 1907-1911 | 0.94 (0.89-0.99) | 0.95 (0.9-1) | 0.25 (0.15-0.41) | 6.48 (3.03-13.82) |
| Central Europe | 1912-1916 | 0.86 (0.82-0.89) | 0.87 (0.83-0.9) | 0.26 (0.18-0.37) | 4.69 (2.73-8.07) |
| Central Europe | 1917-1921 | 0.81 (0.78-0.84) | 0.82 (0.79-0.85) | 0.29 (0.22-0.39) | 3.61 (2.25-5.8) |
| Central Europe | 1922-1926 | 0.8 (0.78-0.82) | 0.81 (0.79-0.83) | 0.35 (0.29-0.42) | 3.04 (2.07-4.47) |
| Central Europe | 1927-1931 | 0.83 (0.81-0.85) | 0.84 (0.82-0.86) | 0.44 (0.37-0.52) | 2.49 (1.75-3.56) |
| Central Europe | 1932-1936 | 0.87 (0.85-0.89) | 0.87 (0.85-0.89) | 0.55 (0.47-0.64) | 2.07 (1.46-2.94) |
| Central Europe | 1937-1941 | 0.88 (0.86-0.9) | 0.88 (0.86-0.9) | 0.68 (0.58-0.79) | 1.65 (1.16-2.36) |
| Central Europe | 1942-1946 | 0.92 (0.9-0.94) | 0.92 (0.9-0.94) | 0.83 (0.72-0.96) | 1.3 (0.89-1.91) |
| Central Europe | 1947-1951 | 1 (1-1) | 1 (1-1) | 1 (1-1) | 1 (1-1) |
| Central Europe | 1952-1956 | 1.03 (1-1.06) | 1.03 (1-1.06) | 1.06 (0.89-1.26) | 0.75 (0.46-1.23) |
| Central Europe | 1957-1961 | 1.05 (1.01-1.1) | 1.05 (1.01-1.1) | 1.16 (0.9-1.51) | 0.6 (0.29-1.21) |
| Central Europe | 1962-1966 | 1.07 (0.99-1.15) | 1.06 (0.98-1.15) | 1.29 (0.85-1.95) | 0.49 (0.14-1.68) |
| Central Latin America | 1897-1901 | 0.89 (0.8-0.99) | 0.92 (0.82-1.02) | 0.22 (0.08-0.58) | 6.92 (0.84-57.19) |
| Central Latin America | 1902-1906 | 0.92 (0.87-0.96) | 0.95 (0.9-0.99) | 0.25 (0.17-0.38) | 5.29 (1.84-15.18) |
| Central Latin America | 1907-1911 | 0.94 (0.91-0.97) | 0.97 (0.94-1) | 0.28 (0.22-0.35) | 4.25 (2.26-8.01) |
| Central Latin America | 1912-1916 | 0.97 (0.94-1) | 1 (0.97-1.03) | 0.32 (0.27-0.38) | 3.54 (2.13-5.9) |
| Central Latin America | 1917-1921 | 1 (0.98-1.03) | 1.03 (1.01-1.06) | 0.38 (0.33-0.44) | 3.09 (1.97-4.83) |
| Central Latin America | 1922-1926 | 1.02 (1-1.04) | 1.05 (1.02-1.07) | 0.44 (0.39-0.5) | 2.64 (1.76-3.96) |
| Central Latin America | 1927-1931 | 1.03 (1.01-1.05) | 1.05 (1.03-1.08) | 0.51 (0.46-0.57) | 2.15 (1.46-3.15) |
| Central Latin America | 1932-1936 | 1.04 (1.02-1.06) | 1.06 (1.04-1.08) | 0.6 (0.54-0.66) | 1.77 (1.21-2.59) |
| Central Latin America | 1937-1941 | 1.04 (1.02-1.06) | 1.05 (1.03-1.07) | 0.72 (0.65-0.79) | 1.48 (1.01-2.16) |
| Central Latin America | 1942-1946 | 1.02 (1.01-1.04) | 1.03 (1.01-1.05) | 0.86 (0.78-0.94) | 1.23 (0.83-1.82) |
| Central Latin America | 1947-1951 | 1 (1-1) | 1 (1-1) | 1 (1-1) | 1 (1-1) |
| Central Latin America | 1952-1956 | 0.98 (0.95-1) | 0.97 (0.95-1) | 1.14 (1.02-1.27) | 0.84 (0.51-1.37) |
| Central Latin America | 1957-1961 | 0.97 (0.93-1.01) | 0.95 (0.92-0.99) | 1.33 (1.14-1.56) | 0.7 (0.35-1.4) |
| Central Latin America | 1962-1966 | 0.97 (0.91-1.03) | 0.94 (0.88-1) | 1.53 (1.22-1.93) | 0.62 (0.22-1.77) |
| Central Sub-Saharan Africa | 1897-1901 | 1.42 (0.95-2.12) | 1.43 (0.96-2.14) | 1.92 (0-1761.69) | 2.17 (0-2165.55) |
| Central Sub-Saharan Africa | 1902-1906 | 1.35 (1.12-1.63) | 1.36 (1.12-1.64) | 1.62 (0.21-12.49) | 1 (0.01-112.34) |
| Central Sub-Saharan Africa | 1907-1911 | 1.29 (1.17-1.44) | 1.3 (1.17-1.44) | 1.01 (0.31-3.28) | 1.35 (0.19-9.79) |
| Central Sub-Saharan Africa | 1912-1916 | 1.27 (1.19-1.35) | 1.27 (1.19-1.36) | 0.92 (0.47-1.82) | 1.08 (0.31-3.72) |
| Central Sub-Saharan Africa | 1917-1921 | 1.24 (1.18-1.3) | 1.24 (1.18-1.31) | 0.93 (0.57-1.5) | 1.19 (0.51-2.78) |
| Central Sub-Saharan Africa | 1922-1926 | 1.21 (1.16-1.26) | 1.21 (1.16-1.26) | 0.96 (0.65-1.42) | 1.16 (0.58-2.33) |
| Central Sub-Saharan Africa | 1927-1931 | 1.17 (1.13-1.21) | 1.17 (1.13-1.22) | 0.97 (0.68-1.37) | 1.11 (0.6-2.06) |
| Central Sub-Saharan Africa | 1932-1936 | 1.13 (1.09-1.17) | 1.13 (1.09-1.18) | 0.99 (0.71-1.37) | 1.04 (0.59-1.85) |
| Central Sub-Saharan Africa | 1937-1941 | 1.09 (1.05-1.13) | 1.09 (1.05-1.13) | 0.99 (0.72-1.36) | 1.03 (0.59-1.78) |
| Central Sub-Saharan Africa | 1942-1946 | 1.04 (1-1.08) | 1.04 (1-1.08) | 0.99 (0.72-1.36) | 1.02 (0.59-1.77) |
| Central Sub-Saharan Africa | 1947-1951 | 1 (1-1) | 1 (1-1) | 1 (1-1) | 1 (1-1) |
| Central Sub-Saharan Africa | 1952-1956 | 0.95 (0.91-0.99) | 0.95 (0.91-0.99) | 1 (0.69-1.43) | 0.98 (0.53-1.82) |
| Central Sub-Saharan Africa | 1957-1961 | 0.91 (0.87-0.96) | 0.91 (0.87-0.96) | 1.01 (0.64-1.58) | 0.95 (0.45-2.01) |
| Central Sub-Saharan Africa | 1962-1966 | 0.89 (0.82-0.96) | 0.88 (0.82-0.96) | 1.03 (0.56-1.87) | 0.95 (0.34-2.67) |
| East Asia | 1897-1901 | 2.61 (1.41-4.85) | 2.63 (1.43-4.85) | 0.16 (0.03-1) | 0.93 (0.09-9.66) |
| East Asia | 1902-1906 | 2.42 (1.97-2.98) | 2.43 (1.98-2.99) | 0.21 (0.11-0.41) | 0.88 (0.4-1.94) |
| East Asia | 1907-1911 | 2.2 (1.99-2.43) | 2.21 (2-2.45) | 0.23 (0.17-0.32) | 0.91 (0.68-1.22) |
| East Asia | 1912-1916 | 1.96 (1.83-2.1) | 1.97 (1.84-2.11) | 0.28 (0.24-0.34) | 0.97 (0.83-1.13) |
| East Asia | 1917-1921 | 1.76 (1.67-1.86) | 1.77 (1.68-1.87) | 0.33 (0.29-0.37) | 0.99 (0.9-1.11) |
| East Asia | 1922-1926 | 1.6 (1.53-1.68) | 1.61 (1.53-1.69) | 0.4 (0.36-0.44) | 1.03 (0.94-1.12) |
| East Asia | 1927-1931 | 1.47 (1.41-1.54) | 1.48 (1.41-1.55) | 0.48 (0.44-0.52) | 1.04 (0.97-1.12) |
| East Asia | 1932-1936 | 1.35 (1.29-1.41) | 1.35 (1.3-1.41) | 0.57 (0.53-0.62) | 1.03 (0.96-1.11) |
| East Asia | 1937-1941 | 1.23 (1.18-1.29) | 1.23 (1.18-1.29) | 0.68 (0.63-0.72) | 1.01 (0.94-1.08) |
| East Asia | 1942-1946 | 1.12 (1.08-1.18) | 1.13 (1.08-1.18) | 0.81 (0.76-0.86) | 0.99 (0.93-1.06) |
| East Asia | 1947-1951 | 1 (1-1) | 1 (1-1) | 1 (1-1) | 1 (1-1) |
| East Asia | 1952-1956 | 0.88 (0.83-0.93) | 0.88 (0.83-0.93) | 1.25 (1.16-1.34) | 1.05 (0.97-1.13) |
| East Asia | 1957-1961 | 0.81 (0.73-0.89) | 0.8 (0.73-0.88) | 1.57 (1.4-1.76) | 1.17 (1.05-1.31) |
| East Asia | 1962-1966 | 0.8 (0.67-0.95) | 0.79 (0.67-0.94) | 1.94 (1.63-2.31) | 1.32 (1.11-1.57) |
| Eastern Europe | 1897-1901 | 3.86 (3.21-4.64) | 3.85 (3.19-4.63) | 6.08 (1.2-30.94) | 0.47 (0-257.43) |
| Eastern Europe | 1902-1906 | 3.22 (2.99-3.47) | 3.21 (2.97-3.46) | 5.44 (2.62-11.31) | 1.44 (0.21-9.94) |
| Eastern Europe | 1907-1911 | 2.82 (2.7-2.95) | 2.81 (2.69-2.94) | 4.55 (2.94-7.05) | 2.31 (0.9-5.92) |
| Eastern Europe | 1912-1916 | 2.4 (2.32-2.48) | 2.39 (2.31-2.47) | 3.29 (2.32-4.65) | 2.18 (1.11-4.28) |
| Eastern Europe | 1917-1921 | 2.08 (2.02-2.14) | 2.08 (2.01-2.14) | 2.42 (1.76-3.33) | 2.2 (1.23-3.94) |
| Eastern Europe | 1922-1926 | 1.82 (1.77-1.87) | 1.82 (1.77-1.87) | 1.85 (1.41-2.44) | 2.1 (1.27-3.48) |
| Eastern Europe | 1927-1931 | 1.71 (1.67-1.75) | 1.71 (1.67-1.75) | 1.53 (1.19-1.96) | 2.03 (1.28-3.22) |
| Eastern Europe | 1932-1936 | 1.58 (1.54-1.62) | 1.58 (1.54-1.62) | 1.32 (1.02-1.72) | 1.81 (1.14-2.87) |
| Eastern Europe | 1937-1941 | 1.36 (1.32-1.39) | 1.36 (1.32-1.39) | 1.19 (0.92-1.55) | 1.57 (0.99-2.47) |
| Eastern Europe | 1942-1946 | 1.15 (1.12-1.18) | 1.15 (1.12-1.18) | 1.06 (0.8-1.41) | 1.25 (0.76-2.06) |
| Eastern Europe | 1947-1951 | 1 (1-1) | 1 (1-1) | 1 (1-1) | 1 (1-1) |
| Eastern Europe | 1952-1956 | 0.86 (0.83-0.88) | 0.86 (0.83-0.88) | 0.85 (0.62-1.18) | 0.81 (0.43-1.54) |
| Eastern Europe | 1957-1961 | 0.73 (0.7-0.77) | 0.73 (0.69-0.77) | 0.77 (0.5-1.18) | 0.66 (0.26-1.71) |
| Eastern Europe | 1962-1966 | 0.63 (0.57-0.68) | 0.62 (0.57-0.68) | 0.62 (0.33-1.17) | 0.49 (0.11-2.22) |
| Eastern Sub-Saharan Africa | 1897-1901 | 1.59 (1.31-1.93) | 1.59 (1.31-1.93) | 0.32 (0-175.49) | 3.87 (0-3532.89) |
| Eastern Sub-Saharan Africa | 1902-1906 | 1.56 (1.44-1.69) | 1.56 (1.44-1.69) | 1.03 (0.24-4.34) | 2.19 (0.29-16.81) |
| Eastern Sub-Saharan Africa | 1907-1911 | 1.52 (1.45-1.59) | 1.52 (1.46-1.59) | 0.98 (0.51-1.87) | 1.71 (0.64-4.53) |
| Eastern Sub-Saharan Africa | 1912-1916 | 1.47 (1.43-1.52) | 1.48 (1.43-1.52) | 1.04 (0.72-1.49) | 1.68 (0.92-3.04) |
| Eastern Sub-Saharan Africa | 1917-1921 | 1.41 (1.38-1.44) | 1.41 (1.38-1.45) | 1.01 (0.78-1.31) | 1.56 (1.01-2.42) |
| Eastern Sub-Saharan Africa | 1922-1926 | 1.34 (1.32-1.37) | 1.35 (1.32-1.38) | 1 (0.82-1.23) | 1.5 (1.05-2.15) |
| Eastern Sub-Saharan Africa | 1927-1931 | 1.28 (1.26-1.3) | 1.28 (1.26-1.31) | 1.01 (0.84-1.21) | 1.38 (1-1.91) |
| Eastern Sub-Saharan Africa | 1932-1936 | 1.21 (1.19-1.23) | 1.21 (1.19-1.23) | 1.01 (0.85-1.2) | 1.28 (0.95-1.73) |
| Eastern Sub-Saharan Africa | 1937-1941 | 1.14 (1.12-1.16) | 1.14 (1.12-1.16) | 1 (0.85-1.18) | 1.17 (0.88-1.55) |
| Eastern Sub-Saharan Africa | 1942-1946 | 1.07 (1.05-1.09) | 1.07 (1.05-1.09) | 1 (0.85-1.17) | 1.08 (0.81-1.44) |
| Eastern Sub-Saharan Africa | 1947-1951 | 1 (1-1) | 1 (1-1) | 1 (1-1) | 1 (1-1) |
| Eastern Sub-Saharan Africa | 1952-1956 | 0.94 (0.92-0.96) | 0.94 (0.92-0.96) | 1 (0.83-1.2) | 0.91 (0.66-1.27) |
| Eastern Sub-Saharan Africa | 1957-1961 | 0.88 (0.86-0.9) | 0.88 (0.85-0.9) | 1 (0.8-1.27) | 0.85 (0.57-1.25) |
| Eastern Sub-Saharan Africa | 1962-1966 | 0.84 (0.8-0.87) | 0.83 (0.8-0.87) | 1.03 (0.75-1.4) | 0.77 (0.45-1.33) |
| High-income Asia Pacific | 1897-1901 | 0.72 (0.63-0.81) | 0.89 (0.8-1) | 0.19 (0.12-0.29) | 1.37 (0.37-5.01) |
| High-income Asia Pacific | 1902-1906 | 0.71 (0.67-0.75) | 0.88 (0.83-0.93) | 0.2 (0.17-0.24) | 1.2 (0.71-2.04) |
| High-income Asia Pacific | 1907-1911 | 0.72 (0.69-0.75) | 0.87 (0.84-0.91) | 0.25 (0.22-0.27) | 1.27 (0.95-1.7) |
| High-income Asia Pacific | 1912-1916 | 0.73 (0.71-0.76) | 0.87 (0.84-0.9) | 0.3 (0.28-0.33) | 1.35 (1.08-1.67) |
| High-income Asia Pacific | 1917-1921 | 0.76 (0.74-0.79) | 0.89 (0.86-0.91) | 0.36 (0.34-0.39) | 1.41 (1.17-1.71) |
| High-income Asia Pacific | 1922-1926 | 0.8 (0.78-0.83) | 0.91 (0.89-0.94) | 0.44 (0.42-0.47) | 1.5 (1.28-1.77) |
| High-income Asia Pacific | 1927-1931 | 0.86 (0.84-0.88) | 0.94 (0.92-0.97) | 0.56 (0.53-0.59) | 1.66 (1.43-1.92) |
| High-income Asia Pacific | 1932-1936 | 0.91 (0.88-0.93) | 0.96 (0.93-0.98) | 0.69 (0.66-0.73) | 1.69 (1.47-1.95) |
| High-income Asia Pacific | 1937-1941 | 0.96 (0.93-0.98) | 0.98 (0.95-1) | 0.83 (0.79-0.88) | 1.57 (1.37-1.8) |
| High-income Asia Pacific | 1942-1946 | 0.99 (0.96-1.01) | 0.99 (0.97-1.02) | 0.93 (0.88-0.98) | 1.29 (1.12-1.5) |
| High-income Asia Pacific | 1947-1951 | 1 (1-1) | 1 (1-1) | 1 (1-1) | 1 (1-1) |
| High-income Asia Pacific | 1952-1956 | 1.05 (1.02-1.09) | 1.06 (1.03-1.1) | 1.06 (0.99-1.14) | 0.85 (0.71-1.03) |
| High-income Asia Pacific | 1957-1961 | 1.16 (1.1-1.22) | 1.2 (1.14-1.26) | 1.11 (1-1.23) | 0.83 (0.64-1.07) |
| High-income Asia Pacific | 1962-1966 | 1.18 (1.09-1.28) | 1.23 (1.13-1.33) | 1.16 (0.99-1.35) | 0.71 (0.47-1.07) |
| High-income North America | 1897-1901 | 0.22 (0.15-0.34) | 0.23 (0.15-0.35) | 0.1 (0.06-0.15) | 1.28 (0.39-4.24) |
| High-income North America | 1902-1906 | 0.28 (0.23-0.33) | 0.29 (0.24-0.35) | 0.12 (0.1-0.15) | 1.38 (0.86-2.22) |
| High-income North America | 1907-1911 | 0.36 (0.33-0.4) | 0.38 (0.34-0.42) | 0.16 (0.14-0.18) | 1.53 (1.14-2.04) |
| High-income North America | 1912-1916 | 0.48 (0.45-0.52) | 0.5 (0.46-0.53) | 0.22 (0.21-0.24) | 1.78 (1.42-2.24) |
| High-income North America | 1917-1921 | 0.62 (0.58-0.66) | 0.64 (0.6-0.67) | 0.32 (0.29-0.34) | 2.03 (1.66-2.5) |
| High-income North America | 1922-1926 | 0.74 (0.7-0.78) | 0.75 (0.72-0.79) | 0.43 (0.4-0.45) | 2.08 (1.72-2.52) |
| High-income North America | 1927-1931 | 0.83 (0.79-0.87) | 0.84 (0.8-0.88) | 0.54 (0.51-0.58) | 2.02 (1.68-2.43) |
| High-income North America | 1932-1936 | 0.91 (0.87-0.95) | 0.92 (0.87-0.96) | 0.68 (0.64-0.72) | 1.85 (1.54-2.23) |
| High-income North America | 1937-1941 | 0.96 (0.91-1) | 0.96 (0.92-1.01) | 0.81 (0.77-0.85) | 1.59 (1.32-1.92) |
| High-income North America | 1942-1946 | 0.98 (0.94-1.03) | 0.99 (0.94-1.03) | 0.91 (0.87-0.96) | 1.28 (1.05-1.56) |
| High-income North America | 1947-1951 | 1 (1-1) | 1 (1-1) | 1 (1-1) | 1 (1-1) |
| High-income North America | 1952-1956 | 1.01 (0.95-1.07) | 1.01 (0.96-1.07) | 1.01 (0.95-1.08) | 0.79 (0.6-1.05) |
| High-income North America | 1957-1961 | 1.02 (0.93-1.11) | 1.02 (0.94-1.12) | 0.97 (0.87-1.07) | 0.59 (0.37-0.95) |
| High-income North America | 1962-1966 | 1.02 (0.87-1.19) | 1.03 (0.88-1.2) | 0.94 (0.79-1.12) | 0.46 (0.18-1.14) |
| North Africa and Middle East | 1897-1901 | 0.67 (0.58-0.78) | 0.68 (0.59-0.79) | 0.28 (0.08-0.99) | 0.15 (0-79.53) |
| North Africa and Middle East | 1902-1906 | 0.73 (0.68-0.78) | 0.73 (0.69-0.79) | 0.31 (0.16-0.63) | 0.52 (0.08-3.51) |
| North Africa and Middle East | 1907-1911 | 0.77 (0.73-0.8) | 0.77 (0.74-0.81) | 0.37 (0.23-0.58) | 0.71 (0.23-2.19) |
| North Africa and Middle East | 1912-1916 | 0.78 (0.75-0.8) | 0.78 (0.76-0.81) | 0.39 (0.28-0.55) | 0.74 (0.31-1.73) |
| North Africa and Middle East | 1917-1921 | 0.77 (0.75-0.79) | 0.78 (0.76-0.8) | 0.39 (0.29-0.52) | 0.76 (0.38-1.5) |
| North Africa and Middle East | 1922-1926 | 0.81 (0.8-0.83) | 0.82 (0.8-0.84) | 0.44 (0.35-0.56) | 0.83 (0.47-1.46) |
| North Africa and Middle East | 1927-1931 | 0.88 (0.86-0.89) | 0.88 (0.86-0.9) | 0.53 (0.43-0.65) | 0.91 (0.56-1.48) |
| North Africa and Middle East | 1932-1936 | 0.92 (0.9-0.94) | 0.92 (0.9-0.94) | 0.6 (0.49-0.72) | 0.97 (0.62-1.54) |
| North Africa and Middle East | 1937-1941 | 0.93 (0.91-0.95) | 0.93 (0.92-0.95) | 0.69 (0.58-0.82) | 0.97 (0.63-1.51) |
| North Africa and Middle East | 1942-1946 | 0.96 (0.94-0.98) | 0.96 (0.94-0.98) | 0.82 (0.7-0.97) | 1 (0.65-1.55) |
| North Africa and Middle East | 1947-1951 | 1 (1-1) | 1 (1-1) | 1 (1-1) | 1 (1-1) |
| North Africa and Middle East | 1952-1956 | 1.02 (1-1.04) | 1.02 (1-1.04) | 1.17 (0.96-1.41) | 0.99 (0.6-1.62) |
| North Africa and Middle East | 1957-1961 | 1.04 (1.01-1.07) | 1.04 (1.01-1.07) | 1.37 (1.07-1.75) | 0.95 (0.49-1.82) |
| North Africa and Middle East | 1962-1966 | 1.08 (1.03-1.12) | 1.07 (1.02-1.12) | 1.66 (1.19-2.31) | 0.94 (0.38-2.29) |
| Oceania | 1897-1901 | 1.23 (0.48-3.11) | 1.19 (0.46-3.07) | 2.89 (0-3302.37) | 10.48 (0-48113.26) |
| Oceania | 1902-1906 | 1.08 (0.72-1.6) | 1.08 (0.73-1.61) | 2.34 (0.02-365.56) | 7.77 (0.01-6153.51) |
| Oceania | 1907-1911 | 1.07 (0.88-1.31) | 1.07 (0.88-1.31) | 0.83 (0.01-46.69) | 5.14 (0.01-1838.78) |
| Oceania | 1912-1916 | 1.09 (0.95-1.24) | 1.09 (0.95-1.24) | 0.85 (0.11-6.61) | 3.49 (0.02-779.44) |
| Oceania | 1917-1921 | 1.11 (1.01-1.24) | 1.12 (1.01-1.24) | 0.82 (0.18-3.61) | 2.47 (0.02-395.19) |
| Oceania | 1922-1926 | 1.12 (1.02-1.22) | 1.12 (1.02-1.22) | 0.89 (0.28-2.85) | 1.9 (0.02-220.27) |
| Oceania | 1927-1931 | 1.08 (1-1.17) | 1.08 (1-1.17) | 0.85 (0.28-2.56) | 1.71 (0.02-154.55) |
| Oceania | 1932-1936 | 1.03 (0.95-1.11) | 1.03 (0.95-1.11) | 0.85 (0.3-2.43) | 1.62 (0.02-118.19) |
| Oceania | 1937-1941 | 1.01 (0.93-1.09) | 1.01 (0.93-1.09) | 0.87 (0.32-2.4) | 1.45 (0.02-89.51) |
| Oceania | 1942-1946 | 1 (0.93-1.08) | 1 (0.93-1.08) | 0.93 (0.35-2.51) | 1.22 (0.02-83.71) |
[truncated: 56,086 more chars]
